# **Social Self-Sorting Synthesis of Molecular Knots**

**DOI:** 10.1021/jacs.2c07682

**Published:** 2022-09-06

**Authors:** Zoe Ashbridge, Olivia M. Knapp, Elisabeth Kreidt, David A. Leigh, Lucian Pirvu, Fredrik Schaufelberger

**Affiliations:** † Department of Chemistry, University of Manchester, Manchester M13 9PL, U.K.; ‡ School of Chemistry and Molecular Engineering, East China Normal University, Shanghai 200062, China

## Abstract

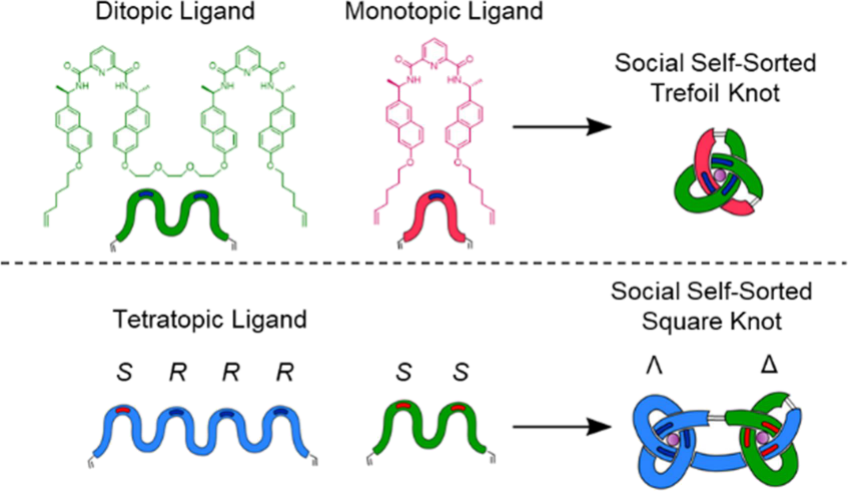

We report the synthesis of molecular prime and composite knots by social self-sorting of 2,6-pyridinedicarboxamide (pdc) ligands of differing topicity and stereochemistry. Upon mixing achiral monotopic and ditopic pdc-ligand strands in a 1:1:1 ratio with Lu(III), a well-defined heteromeric complex featuring one of each ligand strand and the metal ion is selectively formed. Introducing point-chiral centers into the ligands leads to single-sense helical stereochemistry of the resulting coordination complex. Covalent capture of the entangled structure by ring-closing olefin metathesis then gives a socially self-sorted trefoil knot of single topological handedness. In a related manner, a heteromeric molecular granny knot (a six-crossing composite knot featuring two trefoil tangles of the same handedness) was assembled from social self-sorting of ditopic and tetratopic multi-pdc strands. A molecular square knot (a six-crossing composite knot of two trefoil tangles of opposite handedness) was assembled by social self-sorting of a ditopic pdc strand with four (*S*)-centers and a tetratopic strand with two (*S*)- and six (*R*)-centers. Each of the entangled structures was characterized by ^1^H and ^13^C NMR spectroscopy, mass spectrometry, and circular dichroism spectroscopy. The precise control of composition and topological chirality through social self-sorting enables the rapid assembly of well-defined sequences of entanglements for molecular knots.

## Introduction

Knots and entanglements are found at all length scales, from spontaneous random tangling of polymer chains to well-defined specialized climbing and sailing knots.^1^ Self-entanglement of a molecular strand can cause changes in properties such as molecular volume and shape,^2^ strain,^3^ chiral expression,^4^ and photophysical characteristics.^5^ Relatively simple synthetic molecular knot topologies have proven efficacious in areas as diverse as catalysis,^6^ mechanical barrier formation,^7^ dopants for chiral materials,^8^ and nanotherapeutics.^9^

However, accessing different molecular knot scaffolds remains challenging.^10−14^ Single enantiomer^15^ trefoil knots have been synthesized by coordination of three 2,6-pyridinedicarboxamide (pdc) ligands^14^ containing asymmetrically substituted benzyl groups around a lanthanide(III) ion; the point chirality leads to stereoselective assembly of the ligands around the metal center.^14a,15^ This, in turn, directs the topological chirality of the closed-loop knot that results from covalent capture of the complex by ring-closing olefin metathesis^16^ (RCM).

Molecular trefoil knots have been synthesized by the folding and threading of a single tritopic ligand strand^17,18^ (including those containing three pdc units^18^) around a metal ion template, reminiscent of the familiar way that knots are tied in our everyday world. This generates robust trefoil knot precursors (“tangles”^1a,1c^) with unjoined strand ends, so-called overhand knots.^17−20^ Enantiopure overhand knots^18^ can be used for the synthesis^20^ of composite^19−21^ knots, joining together two tangles of either the same (3_1_#3_1_ granny knots) or opposing (3_1_#*3_1_ square knots) handedness.^20^ However, such syntheses require linear synthetic schemes that may be lengthy and result in low overall yields.^19,20^

A useful synthetic strategy for rapidly assembling complexity from simple building blocks is self-sorting.^22^ Self-sorting systems can either be narcissistic^23^—each component preferring to interact with others like themselves—or social,^24^ whereby a compound has greater affinity for components within a system that are different from itself. Narcissistic self-sorting is more common in artificial supramolecular systems, where it tends to yield simpler, high symmetry, homomeric assemblies.^25^

The synthesis of two 12-crossing composite triskelion knots via a Vernier template approach was recently reported.^18b^ By using a coordinative mismatch of lanthanide(III) ions and pdc ligands of varying topicity, entangled assemblies containing the lowest common multiple total binding sites were formed. This allowed for the synthesis of large composite knots from comparatively simple ligand precursors in relatively few synthetic steps.^18b^ However, it also suggests a more general strategy for rapidly accessing complex higher-order entanglements. Here, we report the social self-sorting synthesis of prime and composite molecular knots, exemplified by the entropically driven synthesis of heteromeric trefoil, granny, and square knots.^26^

## Results and Discussion

### Social Self-Sorting Synthesis of a Trefoil Knot Precursor Using Achiral Monotopic and Ditopic Building Blocks

**Figure 1:**
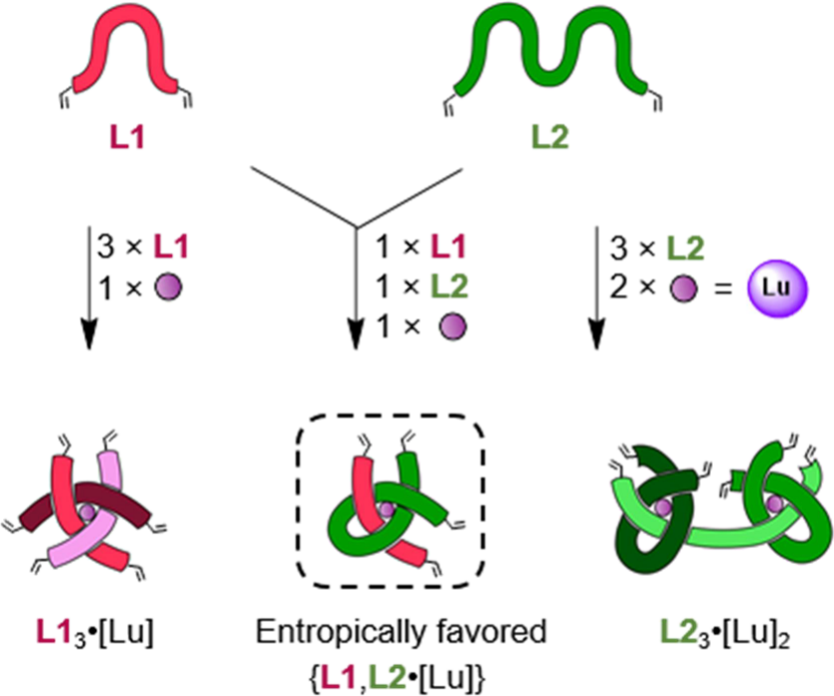
Social self-sorting of ligand strands of different topicity leads to favored heteromeric open knot complexes. Achiral monotopic ligand **L1** and achiral ditopic ligand **L2** form racemic heteromeric trefoil knot precursor {**L1**,**L2**·[Lu]} upon coordination to Lu(III), as this complex requires only two ligand strands to satisfy the lanthanide coordination sphere.

A 1:1:1 mixture of monotopic ligand **L1**, ditopic ligand **L2**, and lutetium(III) ions could potentially form any of three distinct complexes: two narcissistically self-sorted homomeric helicates comprising three identical ligands, or a socially self-sorted heteromeric helicate featuring one of each ligand (Figure 1). In contrast to homomeric complexes **L1**_3_·[Lu] and **L2**_3_·[Lu]_2_, heteromeric complex {**L1**,**L2**·[Lu]} requires only two ligands per metal ion to satisfy the Lu(III) coordination requirements and should therefore be favored.

**Scheme 1:**
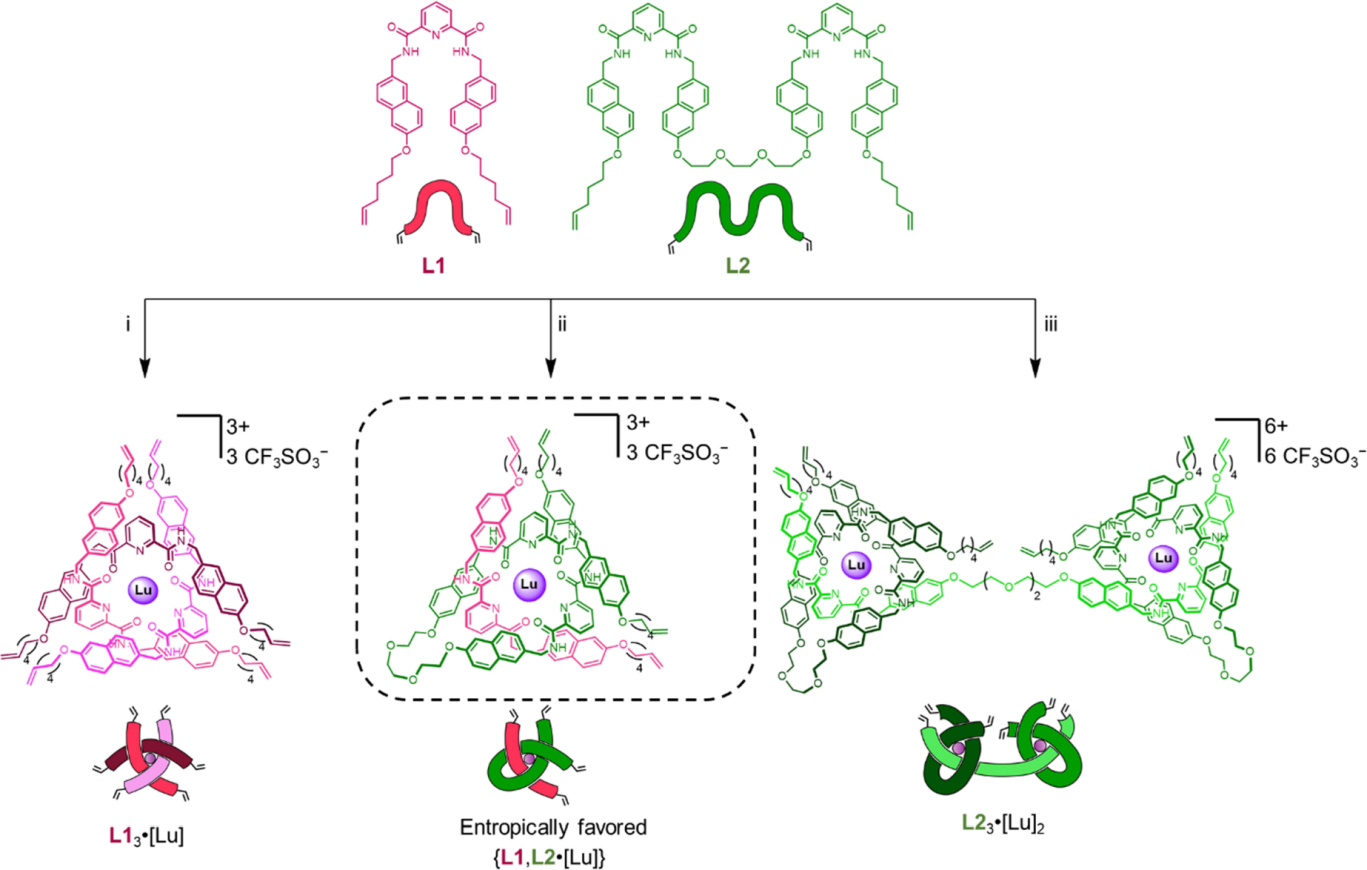
Assembly of Achiral Complexes **L1**_3_·[Lu], **L2**_3_·[Lu]_2_, and Socially Self-Sorted Complex {**L1**,**L2**·[Lu]} Reagents and conditions: (i) 3× **L1**, Lu(CF_3_SO_3_)_3_, MeCN, 80 °C, 2 h, 85%. (ii) **L1**, **L2**, Lu(CF_3_SO_3_)_3_, MeCN, 80 °C, 20 h, 92%. (iii) 3× **L2**, 2× Lu(CF_3_SO_3_)_3_, MeCN, 80 °C, 48 h, 82%.

To assess this concept, first of all, homomeric complexes of each type of ligand coordinated to lutetium(III) were prepared (Scheme 1). Achiral monotopic ligand **L1** and ditopic ligand **L2** were synthesized as described in the Supporting Information (Scheme S2). Complexation of three equivalents of **L1** with one equivalent of lutetium(III) trifluoromethanesulfonate in acetonitrile afforded helicate **L1**_3_·[Lu] upon heating to 80 °C for 2 h (Schemes 1i and S4).^14^ The progress of the coordination process was monitored by electrospray ionization (ESI) mass spectrometry (Figure S77) and ^1^H nuclear magnetic resonance (NMR) spectroscopy (Figure S48). Under similar reaction conditions, ditopic ligand **L2** was complexed to Lu(III) in a 3:2 ratio, generating complex **L2**_3_·[Lu]_2_ over 48 h (Schemes 1iii and S5). The ^1^H NMR spectrum (Figure S2) and ESI mass spectrum (Figure S79) confirmed that an entangled species with 3:2 ligand:metal ratio had been formed.^18b^

**Figure 2:**
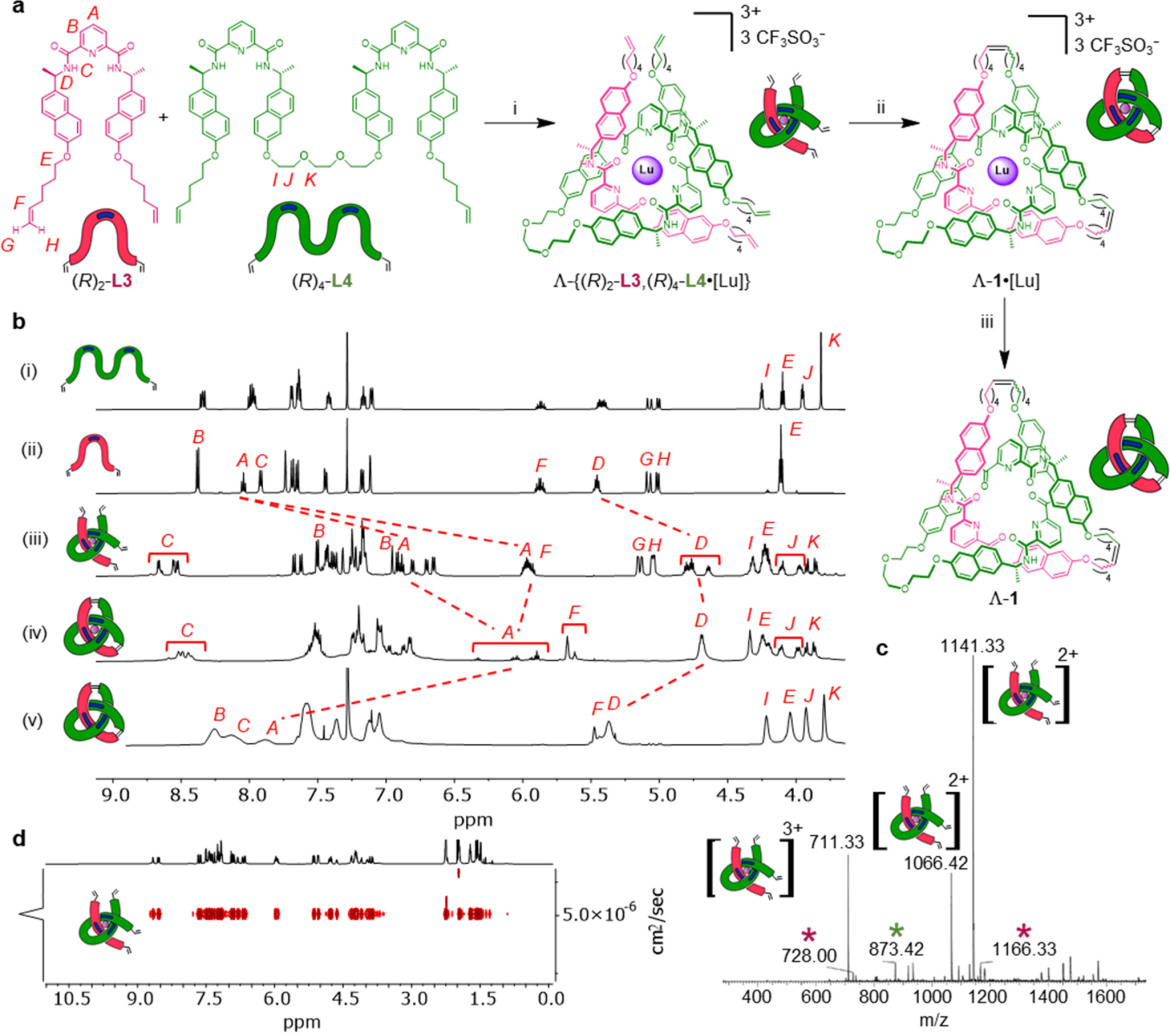
Socially self-sorted assembly of a trefoil knot of single topological handedness. (a) Synthesis of trefoil knot Λ-**1**. Reagents and conditions: (i) Lu(CF_3_SO_3_)_3_, MeCN, 80 °C, 24 h. (ii) Hoveyda–Grubbs second generation catalyst,^16^ CH_2_Cl_2_/CH_3_NO_2_ 1:1 (v/v), 50 °C, 24 h, 53% over two steps. (iii) Et_4_NF, MeCN, r.t., 0.5 h, 35% over three steps. Stereochemistry indicated by dark blue bars (*R*-stereocenters) on the cartoon representations of the ligand strands. (b) Partial ^1^H NMR spectral stack plot of trefoil knot Λ-**1** and precursors (600 MHz, 298 K): (i) ligand (*R*)_4_-**L4** (CDCl_3_), (ii) ligand (*R*)_2_-**L3** (CDCl_3_), (iii) open trefoil knot complex Λ-{(*R*)_2_-**L3**,(*R*)_4_-**L4**·[Lu]} (MeCN-*d*_3_), (iv) metalated knot Λ-**1**·[Lu] (MeCN-*d*_3_), and (v) metal-free knot Λ-**1** (CDCl_3_). Proton assignments refer to atom labels in part (a). For full assignments, see Supporting Information. (c) Low resolution ESI-MS(+) of a crude open trefoil knot complex mixture, showing major signals corresponding to Λ-{(*R*)_2_-**L3**,(*R*)_4_-**L4**·[Lu]}, and small signals corresponding to homomeric complexes Λ-((*R*)_2_-**L3**)_3_·[Lu] (pink *) and (Λ,Λ)-((*R*)_4_-**L4**)_3_·[Lu]_2_ (green *). (d) DOSY ^1^H NMR spectrum of open trefoil knot complex Λ-{(*R*)_2_-**L3**,(*R*)_4_-**L4**·[Lu]} (600 MHz, 298 K, MeCN-*d*_3_), showing a single species is present in solution.

We then investigated the propensity of **L1** and **L2** to self-sort into heteromeric coordination complexes. An acetonitrile solution containing a 1:1:1 mixture of **L1**, **L2** and lutetium(III) trifluoromethanesulfonate was heated at 80 °C (Scheme 1ii). After 20 h, the major ions in the ESI mass spectrum corresponded to the heteromeric complex {**L1**,**L2**·[Lu]}, with no evidence of homomeric complexes **L1**_3_·[Lu] or **L2**_3_·[Lu]_2_ (Figure S81), indicating that the mixture does, indeed, socially self-sort to form the favored complex. Given the similarity in coordination chemistry of different lanthanides, it appears likely that the high selectivity observed originates mainly from entropic effects and, in particular, the number of species in each complex. Next, in order to use social self-sorting to control the crossing sequences more generally using this strategy, directors for entanglement stereochemistry were also introduced (Figure 2).^14,18^

### Social Self-Sorting Synthesis of Trefoil Knots with Chiral Monotopic and Ditopic Building Blocks

To use point chirality to direct tangle stereochemistry within the self-sorted complexes, ditopic ligand (*R*)_4_-**L4** and monotopic ligand (*R*)_2_-**L3** were synthesized as described in the Supporting Information (Scheme S3). Homomeric complexes Λ-((*R*)_2_-**L3**)_3_·[Lu] and (Λ,Λ)-((*R*)_4_-**L4**)_3_·[Lu]_2_ were prepared in an analogous manner to the reactions featuring the achiral building blocks (Scheme S7).^15^ Addition of lutetium(III) trifluoromethanesulfonate (1 equiv) to a 1:1 mixture of (*R*)_2_-**L3** and (*R*)_4_-**L4** in acetonitrile resulted in the formation of heteromeric complex Λ-{(*R*)_2_-**L3**,(*R*)_4_-**L4**·[Lu]} after 24 h at 80 °C (Figure 2a).

The progress of the assembly process was monitored by ^1^H NMR spectroscopy (Figure 2(bi–iii)) and ESI-MS (Figure 2c). The most abundant ions in the ESI mass spectrum (*m*/*z* Λ-{(*R*)_2_-**L3**,(*R*)_4_-**L4**·[Lu]}^3+^ 711.3, Λ-{(*R*)_2_-**L3**,(*R*)_4_-**L4**·[Lu]}-H^2+^ 1066.4, Λ-{(*R*)_2_-**L3**,(*R*)_4_-**L4**·[Lu]}[CF_3_SO_3_]^2+^ 1141.3) correspond to the heteromeric complex, while smaller signals arise from the homomeric circular helicate (*m*/*z* Λ-((*R*)_2_-**L3**)_3_·[Lu]^3+^ 728.0, Λ-((*R*)_2_-**L3**)_3_·[Lu][CF_3_SO_3_]^2+^ 1166.3) and open granny knot complex (*m*/*z* (Λ,Λ)-((*R*)_4_-**L4**)_3_·[Lu]_2_[CF_3_SO_3_]^5+^ 873.4) (Figure 2c). High-resolution mass spectrometry displays isotopic distributions for each complex consistent with the calculated values (Figure S84).

Diffusion-ordered NMR spectroscopy (DOSY) indicates a single species is present in solution (Figure 2d). The CD spectrum showed exciton couplings and signal intensities consistent with previously reported tangled pdc complexes (Figure S121).^14,15,18^ Substantial upfield shifts of protons H_A_ and H_D_ in Λ-{(*R*)_2_-**L3**,(*R*)_4_-**L4**·[Lu]} (Figure 2b(iii)) result from shielding by the naphthalene rings and are consistent with an entangled conformation.^14^ The splitting of the H_A_ signals into different regions (∼7.0 and 6.0 ppm) reflects the difference in the environments of their positions in the coordination complex. The pyridine protons at the open side of the complex (green pyridine sites) are less shielded than the more tightly bound (pink) sites internal to the structure.^4b^ Additional splitting of each set of protons for H_B_, H_C_, and H_D_ into chemically distinct environments also reflects the formation of the low-symmetry coordination complex, Λ-{(*R*)_2_-**L3**,(*R*)_4_-**L4**·[Lu]}.

The entangled complex Λ-{(*R*)_2_-**L3**,(*R*)_4_-**L4**·[Lu]} was covalently captured by RCM using a Hoveyda–Grubbs second generation catalyst to give the closed-loop trefoil knot Λ-**1**·[Lu] (Figure 2a). The ESI mass spectrum of the crude reaction mixture after RCM showed only ions corresponding to the desired knot, Λ-**1**·[Lu] (*m*/*z* Λ-**1**·[Lu]^3+^ 692.7, Λ-**1**·[Lu]-H^2+^ 1038.4, Λ-**1**·[Lu][CF_3_SO_3_]^2+^ 1113.4), with no trace of assemblies derived from narcissistic self-sorting (Figure S3). The DOSY spectrum indicated a single species (Figure S72), and the CD spectrum confirmed that the entanglement stereochemistry is conserved in the closed-loop knot (Figure S109). Purification by size exclusion chromatography removed small amounts of unreacted starting material and larger molecular weight species to give trefoil knot Λ-**1**·[Lu] in 53% yield over two steps (Scheme S8). The modest isolated yield results from the oligomeric and polymeric side products from alkene metathesis and the loss of some of the poorly soluble knot during chromatography.

The ^1^H NMR spectrum of Λ-**1**·[Lu] shows the absence of terminal alkenes (Figure 2b(iv)). The narrower range of chemical shifts of H_A_ with respect to the open complex (Λ-{(*R*)_2_-**L3**,(*R*)_4_-**L4**·[Lu]}) reflects the similarity between the glycol-linked and alkyl chain-linked environments after closure by RCM.

Knot Λ-**1**·[Lu] was readily demetalated by tetraethylammonium fluoride to give wholly organic knot Λ-**1** (Scheme S9). To confirm the absence of alternative entangled species, the reaction was also carried through all three steps from the ligand precursors to Λ-**1** without purification of the intermediates or final product. The matrix-assisted laser desorption/ionization (MALDI) spectrum of the crude reaction mixture after the final step features only ions corresponding to Λ-**1** (*m*/*z* [Λ-**1** + Na]^+^ 1926.3, [Λ-**1** + K]^+^ 1942.0, Figure S6). The absence of homomeric side products after kinetic trapping of the compound by RCM highlights the effectiveness of entropy-driven error correction in the system.

The demetalated knot Λ-**1** was subsequently isolated by size exclusion chromatography in overall 35% yield over three steps (Figure 2a). The ^1^H NMR spectrum of Λ-**1** is broad and typical of other molecular knots that have no single well-defined conformation (Figure 2b(v)).^2^ The metalated knot Λ-**1**·[Lu] could be smoothly regenerated (94% yield) by treatment of Λ-**1** with one equivalent of lutetium(III) trifluoromethanesulfonate in acetonitrile at 80 °C (Scheme S9).

### Social Self-Sorting Synthesis of Composite Knots with Chiral Ditopic and Tetratopic Building Blocks

**Figure 3:**
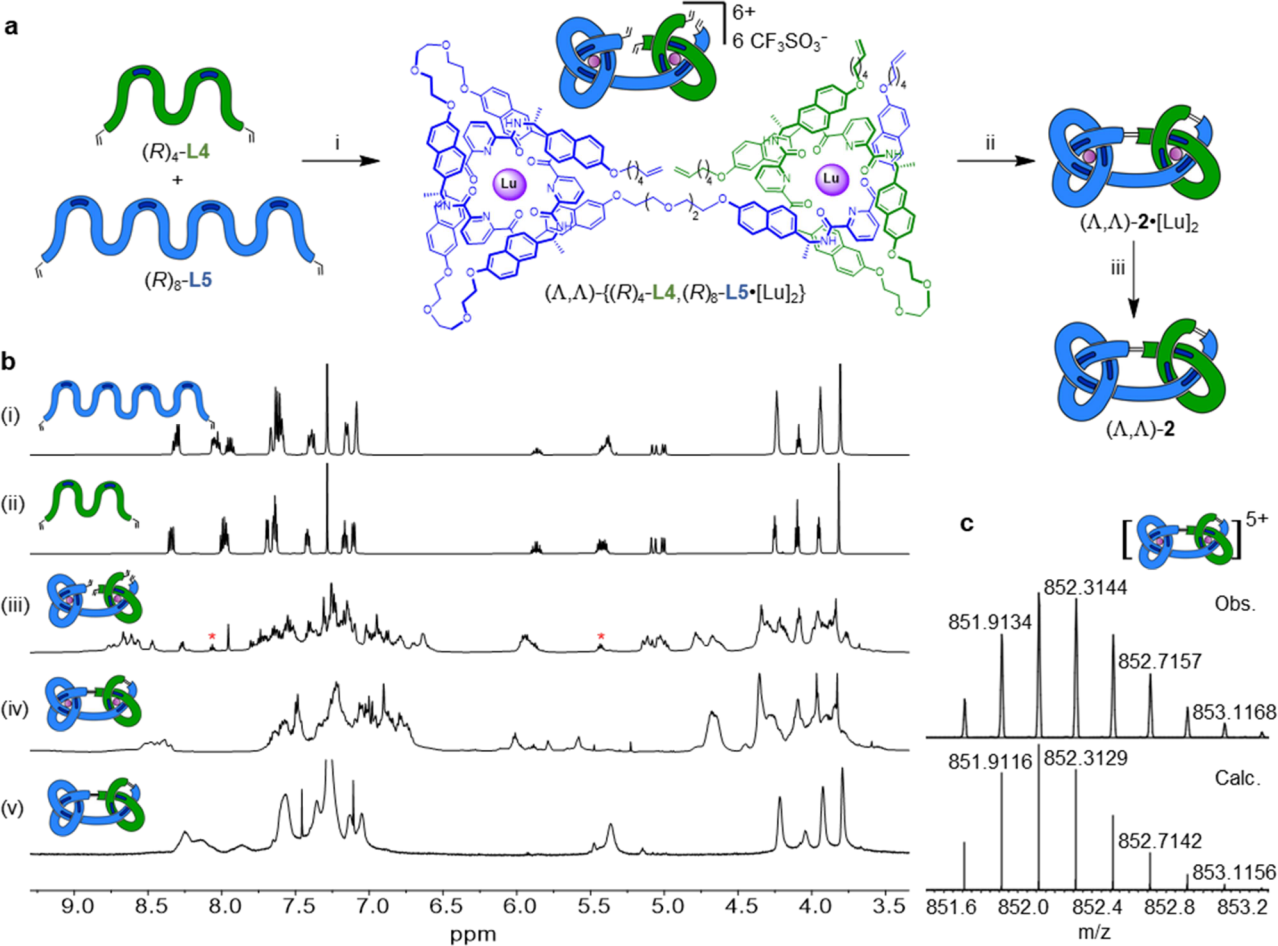
Socially self-sorted assembly of a granny knot of single topological handedness. (a) Synthesis of granny knot (Λ,Λ)-**2**. Reagents and conditions: (i) Lu(CF_3_SO_3_)_3_, MeCN, 80 °C, 72 h. (ii) Hoveyda–Grubbs second generation catalyst, CH_2_Cl_2_/CH_3_NO_2_ 1:1 (v/v), 50 °C, 24 h, 26% over two steps. (iii) Et_4_NF, MeCN, r.t., 0.5 h, 13% over three steps. (b) Partial ^1^H NMR spectral stack plot of granny knot (Λ,Λ)-**2** and precursors (600 MHz, 298 K): (i) ligand (*R*)_8_-**L5** (CDCl_3_), (ii) ligand (*R*)_4_-**L4** (CDCl_3_), (iii) open granny knot complex (Λ,Λ)-{(*R*)_4_-**L4**,(*R*)_8_-**L5**·[Lu]_2_} (MeCN-*d*_3_), (iv) metalated knot (Λ,Λ)-**2**·[Lu]_2_ (MeCN-*d*_3_), and (v) metal-free granny knot (Λ,Λ)-**2** (CDCl_3_). Uncoordinated ligand impurities are indicated *. For full assignments, see Supporting Information. (c) High-resolution ESI-MS(+) of closed-loop granny knot (Λ,Λ)-**2**·[Lu]_2_, comparing the observed spectrum (above) to calculated isotopic distribution of [M – 5(CF_3_SO_3_)]^5+^ (below).

The applicability of the social self-sorting approach to more complex systems was then explored in the formation of a granny knot complex directly from two different chiral ligands. Ditopic ligand (*R*)_4_-**L4** and tetratopic ligand (*R*)_8_-**L5** were treated with lutetium(III) trifluoromethanesulfonate in a 1:1:2 ratio (Figure 3a). After 24 h, the ESI mass spectrum showed several ions corresponding to a heteromeric complex (*m*/*z* (Λ,Λ)-{(*R*)_4_-**L4**,(*R*)_8_-**L5**·[Lu]_2_}^6+^ 694.8, (Λ,Λ)-{(*R*)_4_-**L4**,(*R*)_8_-**L5**·[Lu]_2_}[CF_3_SO_3_]^5+^ 863.4, (Λ,Λ)-{(*R*)_4_-**L4**,(*R*)_8_-**L5**·[Lu]_2_}[CF_3_SO_3_]_2_^4+^ 1116.4, (Λ,Λ)-{(*R*)_4_-**L4**,(*R*)_8_-**L5**·[Lu]_2_}[CF_3_SO_3_]_3_^3+^ 1538.2, Figure S89). Other ions in the spectrum correspond to overhand knot fragments containing a single non-coordinated ligand site, as observed previously for related open Vernier lanthanide complexes.^18b^ The complex consisting of just one tetratopic ligand (*m*/*z* Λ-(*R*)_8_-**L5**·[Lu]^3+^ 901.3, Λ-(*R*)_8_-**L5**·[Lu][CF_3_SO_3_]^2+^ 1425.9) can result from fragmentation of either the heteromeric granny knot or the Vernier template triskelion assembly. However, the complex consisting of two ditopic ligands (*m*/*z* Λ-((*R*)_4_-**L4**)_2_·[Lu]^3+^ 918.0, Λ-((*R*)_4_-**L4**)_2_·[Lu][CF_3_SO_3_]^2+^ 1450.8) can only arise from fragmentation of the heteromeric granny complex. The ^1^H NMR spectrum shows shifts characteristic of strand entanglement [Figure 3(bi–iii)) and additional small signals which correspond to residual unbound ligand even after prolonged reaction times (signals marked *).

The alkene end groups of complex (Λ,Λ)-{(*R*)_4_-**L4**,(*R*)_8_-**L5**·[Lu]_2_} were joined by RCM and the resulting closed-loop knot demetalated by treatment with Et_4_NF (Figure 3a).^14b,15^ As with trefoil knot Λ-**1**, the three steps to granny knot (Λ,Λ)-**2** were also undertaken without isolation of the intermediates to examine the efficacy of the self-sorting. We found no evidence of alternative homomeric products in the mass spectrum of the metalated (Figure S8) or demetalated (Figure S11) crude reaction mixtures, although a trefoil-entangled side product derived from intramolecular closure of fragment Λ-(*R*)_8_-**L5**·[Lu] was observed throughout. The demetalated granny knot was isolated by size exclusion chromatography, yielding (Λ,Λ)-**2** in 13% yield over three steps (Scheme S11). The metalated knot (Λ,Λ)-**2**·[Lu]_2_ could be isolated either after purification by size exclusion chromatography in 26% yield in two steps from (*R*)_4_-**L4** and (*R*)_8_-**L5** (Figure 3a) or in 68% yield by remetalation of (Λ,Λ)-**2** (Scheme S11).

The molecular masses of (Λ,Λ)-**2**·[Lu]_2_ and (Λ,Λ)-**2** were confirmed by HRMS (Figure 3c) and MALDI-TOF (Figure S97), respectively. The ^1^H NMR spectrum of (Λ,Λ)-**2**·[Lu]_2_ (Figure 3b(iv)) is similar to that of previously reported composite knots,^20,21^ and the ^1^H NMR spectrum of the demetalated knot (Λ,Λ)-**2** is broad (Figure 3b(v)).

### Social Self-Sorting of Pre-Coordinated Entangled Strands under Thermodynamic Control

**Figure 4:**
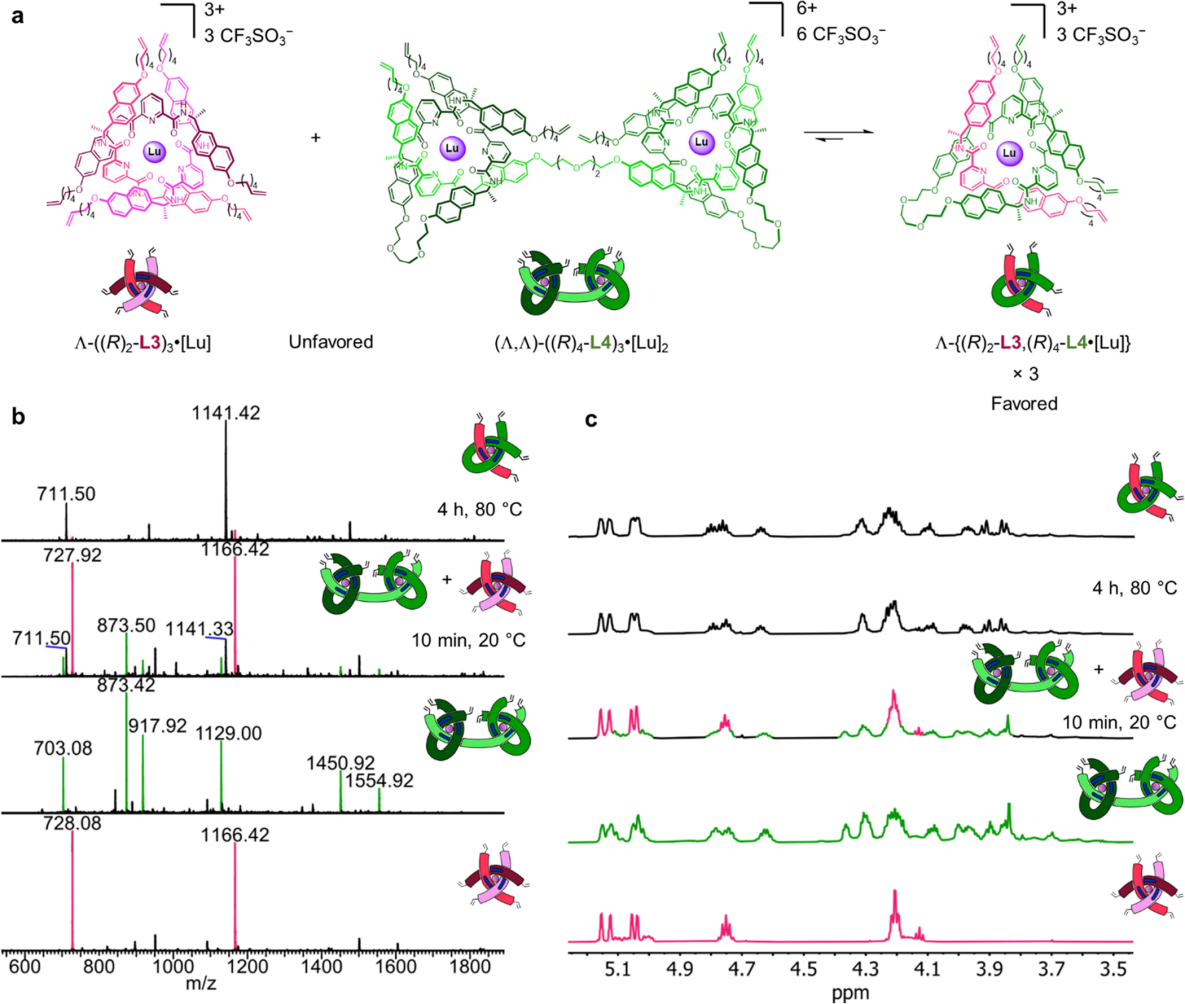
Socially self-sorted rearrangement of complexes to give a heteromeric complex of single entanglement stereochemistry. (a) Synthesis of heteromeric complex Λ-{(*R*)_2_-**L3**,(*R*)_4_-**L4**·[Lu]}. Reagents and conditions: MeCN, 80 °C, 4 h. (b) Comparison of precursor complexes and (qualitative) in situ monitoring of rearrangement by ESI(+) mass spectrometry. (c) Comparison of precursor complexes and (qualitative) in situ monitoring of rearrangement by ^1^H NMR spectroscopy (600 MHz, 298 K, MeCN-*d*_3_), including a reference sample of pristine heteromeric complex Λ-{(*R*)_2_-**L3**,(*R*)_4_-**L4**·[Lu]} (top).

The dynamic conversion of entangled complexes was also investigated (Figure 4a). Equimolar solutions of homomeric helicate Λ-((*R*)_2_-**L3**)_3_·[Lu] and open granny knot complex (Λ,Λ)-((R)_4_-**L4**)_3_·[Lu]_2_ in MeCN were mixed, and the evolution of heteromeric complex Λ-{(*R*)_2_-**L3**,(*R*)_4_-**L4**·[Lu]} was monitored by ^1^H NMR spectroscopy and ESI mass spectrometry. Within 10 min at room temperature, ions corresponding to Λ-{(*R*)_2_-**L3**,(*R*)_4_-**L4**·[Lu]} became apparent by mass spectrometry, becoming the dominant species after 4 h at 80 °C (Figure 4b). Near-complete conversion to the heteromeric socially self-sorted complex was qualitatively confirmed by ^1^H NMR spectroscopy (Figure 4c). The dynamic rearrangement of pre-formed complexes to an entropically favored heteromeric compound was also qualitatively shown by an equimolar mixture of granny knot complex (Λ,Λ)-((*R*)_4_-**L4**)_3_·[Lu]_2_ and triskelion knot complex (Λ_3_,Λ)-((*R*)_8_-**L5**)_3_·[Lu]_4_ (see Section S4.7).

### Control of Entanglement Stereochemistry with Building Block Point Chirality

Monotopic pdc lanthanide helicates containing either (*R*)- or (*S*)-stereogenic centers do not self-sort on the basis of chirality.^15^ However, covalently tethered tritopic ligands containing four (*R*)- and two (*S*)-stereocenters do not self-entangle upon coordination to lanthanide(III) ions because of steric clashes arising from the strand stereochemistry.^18b^ We therefore explored combining stereochemical discrimination and entropically driven social self-sorting in order to prepare a composite knot containing tangles of opposing handedness. Ditopic ligand (*S*)_4_-**L4** and tetratopic ligand (*S*)_2_(*R*)_6_-**L5** were synthesized as previously reported.^18b^ The combination of six stereocenters of (*R*)-chirality and six of (*S*)-chirality across the two ligands is required for forming two trefoil tangles of opposing handedness, a 3_1_#*3_1_ “square knot” (Λ,Δ)-**2**, and a diastereomer of granny knot (Λ,Λ)-**2** (Schemes S10 and S12).

**Figure 5:**
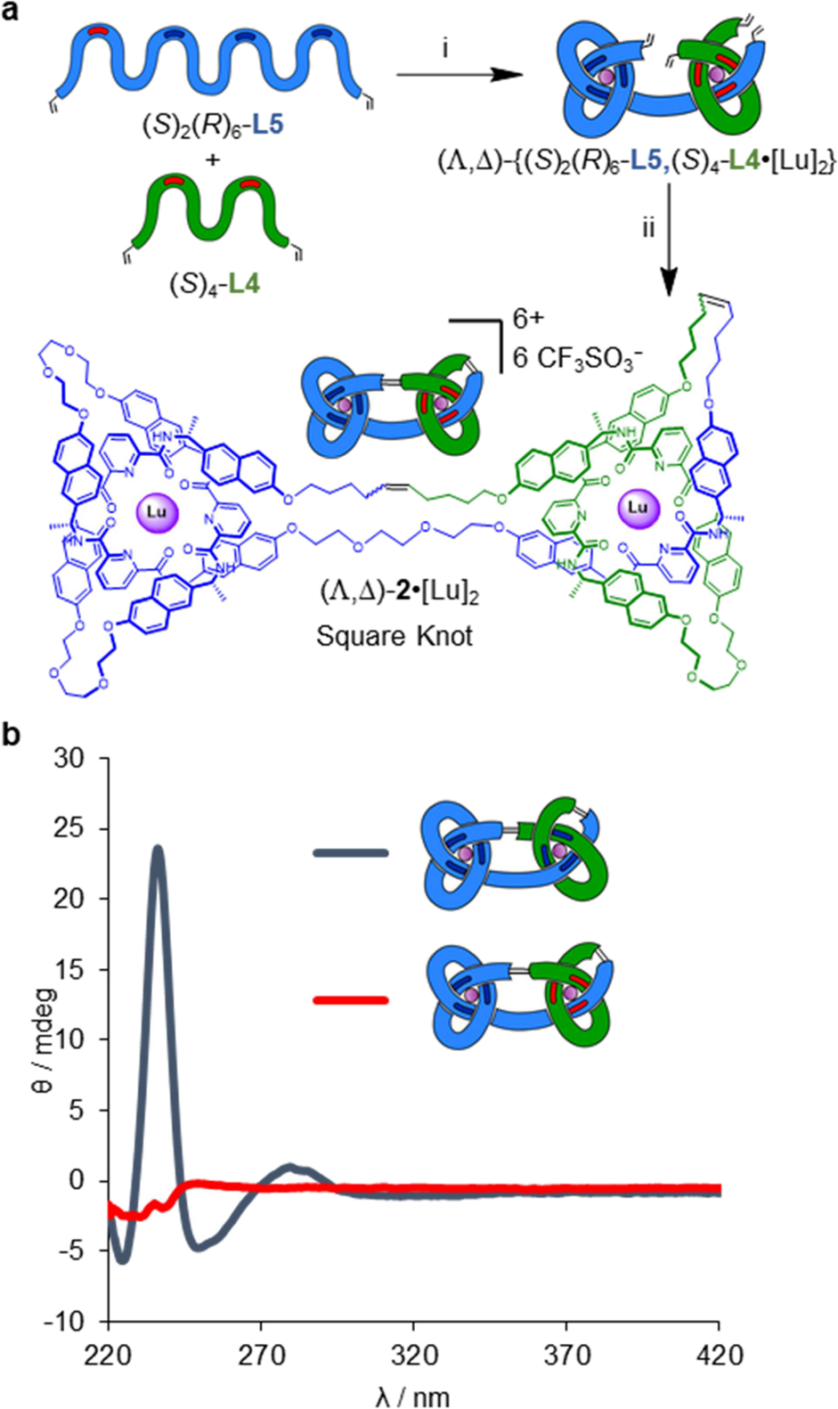
Socially self-sorted assembly of a square knot (*meso*-topological handedness). (a) Synthesis of square knot (Λ,Δ)-**2**·[Lu]_2_. Reagents and conditions: (i) Lu(CF_3_SO_3_)_3_, MeCN, 80 °C, 72 h. (ii) Hoveyda–Grubbs second generation catalyst,^16^ CH_2_Cl_2_/CH_3_NO_2_ 1:1 (v/v), 50 °C, 24 h, 29% over two steps. (b) CD spectral stack plot (5 × 10^–5^ M, MeCN, normalized for absorbance) showing comparison of granny knot (Λ,Λ)-**2**·[Lu]_2_ (blue) and square knot (Λ,Δ)-**2**·[Lu]_2_ (red).

To assemble square knot (Λ,Δ)-**2**, a 1:1:2 mixture of tetratopic ligand (*S*)_2_(*R*)_6_-**L5**, ditopic ligand (*S*)_4_-**L4** and lutetium(III) trifluoromethanesulfonate in acetonitrile afforded open square knot complex (Λ,Δ)-{(*S*)_4_-**L4**,(*S*)_2_(*R*)_6_-**L5**·[Lu]_2_} after heating at 80 °C for 72 h (Figure 5a). Its mass spectrum was similar to that of the diastereomeric granny knot complex (Λ,Λ)-{(*R*)_4_-**L4**,(*R*)_8_-**L5**·[Lu]_2_} (Figure S93). Complex (Λ,Δ)-{(*S*)_4_-**L4**,(*S*)_2_(*R*)_6_-**L5**·[Lu]_2_} is pseudo-achiral, particularly in terms of the environment and stereochemistry of the point-chiral centers around each coordinated Lu(III) ion, and accordingly gives a near baseline CD spectrum (Figure S122). Joining of the terminal alkenes of (Λ,Δ)-{(*S*)_4_-**L4**,(*S*)_2_(*R*)_6_-**L5**·[Lu]_2_} by RCM gave square knot (Λ,Δ)-**2**·[Lu]_2_ (Scheme S12). Subsequent demetalation by Et_4_NF afforded square knot (Λ,Δ)-**2** in 7% yield over three steps (Scheme S13).

Square knot (Λ,Δ)-**2**·[Lu]_2_ and granny knot (Λ,Λ)-**2**·[Lu]_2_ have virtually indistinguishable ^1^H NMR spectra (Figures S60 and S64) but strikingly different CD responses (Figure 5b). The small deviations from the baseline in the CD spectrum of (Λ,Δ)-**2**·[Lu]_2_ are likely the result of the different connectivities of the point-chiral groups on the strand.

## Conclusions

Our findings demonstrate that molecular prime and composite knots can be rapidly assembled by social self-sorting using 2,6-pyridinedicarboxamide-containing strands of different topicity. A 1:1 ratio of monotopic and ditopic pdc ligands coordinates to Lu(III) to selectively generate a heteromeric precursor complex to a trefoil knot. Molecular granny and square knots can be assembled through social self-sorting of chiral ditopic and tetratopic ligand strands. The pdc-Ln(III) social self-sorting is dynamic, with pre-coordinated lanthanide complexes of the ligand strands rapidly rearranging to the entropically preferred self-sorted structures. Social self-sorting of programed pdc-ligand strands is a highly effective new addition to the strategies^10−13,18^ available for the rapid assembly of well-defined sequences of orderly molecular entanglements. The ability to access low-symmetry knots with simpler synthetic strategies provides new avenues to explore the functions and properties associated with molecular entanglements.

## Supporting Information Available

Additional experimental details including synthetic procedures and characterization data (PDF)

## Abbreviations

OTf: trifluoromethanesulfonate
pdc: 2,6-pyridinedicarboxamide
MALDI: matrix-assisted laser desorption/ionization
RCM: ring-closing olefin metathesis
r.t.: room temperature.
