# Supplementary material for: Social Self-Sorting Synthesis of Molecular Knots
Source: J Am Chem Soc. 2022 Sep 6;144(37):17232–40. doi: 10.1021/jacs.2c07682 (PMC9501921; doi:10.1021/jacs.2c07682)
Supplement: Supplementary file 1 — ja2c07682_si_001.pdf [file ja2c07682_si_001.pdf]

Social Self-Sorting Synthesis of Molecular Knots

Zoe Ashbridge[1], Olivia M. Knapp[1], Elisabeth Kreidt[1], David A. Leigh[1,2*], Lucian Pirvu[1], Fredrik Schaufelberger[1]

[1]Department of Chemistry, University of Manchester, Manchester M13 9PL, United Kingdom.

[2]School of Chemistry and Molecular Engineering, East China Normal University, 200062 Shanghai, China.

* Correspondence to: david.leigh@manchester.ac.uk

- Supporting Information -

Table of Contents

S1.	Abbreviations	S3
S2.	General Experimental	S4
S3.	Reaction Schemes	S5
	S3.1 Synthesis of ligands	S5
	S3.2 Synthesis of achiral open complexes	S7
	S3.3 Synthesis of enantiopure Λ-handed open complexes	S10
	S3.4 Synthesis of trefoil knot Λ-**1**	S12
	S3.5 Synthesis of granny knot (Λ,Λ)-**2**	S14
	S3.6 Synthesis of square knot (Λ,Δ)-**2**	S16
	S3.7 In-situ rearrangement of helicate Λ-((R)$_2$-**L3**)$_3$•[Lu] and granny complex (Λ,Λ)-((R)$_4$-**L4**)$_3$•[Lu]$_2$ to form helicate Λ-{(R)$_2$-**L3**,(R)$_4$-**L4**•[Lu]}	S18
	S3.8 In-situ rearrangement of triskelion complex (Λ$_3$,Λ)-((R)$_8$-**L5**)$_3$•[Lu]$_4$ and granny complex (Λ,Λ)-((R)$_4$-**L4**)$_3$•[Lu]$_2$ to form granny complex (Λ,Λ)-{(R)$_4$-**L4**,(R)$_8$-**L5**•[Lu]$_2$}	S18
S4.	Experimental Procedures	S19
	S4.1 Synthesis of ligands	S19
	S4.2 Synthesis of achiral open complexes	S24
	S4.3 Synthesis of enantiopure Λ-handed open complexes	S28
	Synthesis of trefoil knot Λ-**1**	S29
	S4.4 Synthesis of granny knot (Λ,Λ)-**2**	S36
	S4.5 Synthesis of square knot (Λ,Δ)-**2**	S43
	S4.6 In-situ rearrangement of helicate Λ-((R)$_2$-**L3**)$_3$•[Lu] and granny complex (Λ,Λ)-((R)$_4$-**L4**)$_3$•[Lu]$_2$ to form helicate Λ-{(R)$_2$-**L3**,(R)$_4$-**L4**•[Lu]}	S49
	S4.7 In-situ rearrangement of triskelion complex (Λ$_3$,Λ)-((R)$_8$-**L5**)$_3$•[Lu]$_4$ and granny complex (Λ,Λ)-((R)$_4$-**L4**)$_3$•[Lu]$_2$ to form granny complex (Λ,Λ)-{(R)$_4$-**L4**,(R)$_8$-**L5**•[Lu]$_2$}	S57
S5.	NMR Spectra	S66
	S5.1 ^{1}H and ^{13}C NMR spectra	S66
	S5.2 DOSY NMR spectra	S83
S6.	Mass Spectra	S88
	S6.1 LRESI and HRESI spectra	S88
	S6.2 MALDI-TOF spectra	S98
S7.	CD and Absorption Spectra	S99
S8.	Further 2D NMR Spectroscopic Characterisation	S109
S9.	References	S110

S1. Abbreviations

Bu butyl; CD Circular Dichroism; CHCA α-Cyano-4-hydroxycinnamic acid; COSY Correlated Spectroscopy; DEPT Distortionless Enhancement by Polarization Transfer; DMF *N*,*N*-dimethylformamide; DMSO dimethyl sulfoxide; DOSY Diffusion-Ordered Spectroscopy; ESI electrospray ionization; Et ethyl; HMBC Heteronuclear Multiple Bond Correlation; HRMS high-resolution mass spectrometry; HSQC Heteronuclear Single Quantum Coherence; MALDI-TOF Matrix Assisted Laser Desorption/Ionization Time-of-Flight; MeCN acetonitrile; MeOH methanol; NMR Nuclear Magnetic Resonance; OTf triflate; PEG polyethylene glycol; RCM ring closing metathesis; r.t. room temperature; TLC thin layer chromatography.

For chiral knots, the naming convention of Mislow and co-workers is adopted throughout.[1]

S2. General Experimental

Unless stated otherwise, reagents were obtained from commercial sources and used without purification. Reactions were carried out in anhydrous solvents and under an N_2 atmosphere. Anhydrous solvents were obtained by passing the solvent through an activated alumina column on a Phoenix SDS (solvent drying system; JC Meyer Solvent Systems, CA, USA). ^{1}H NMR spectra were recorded on a Bruker Avance III instrument with an Oxford AS600 magnet equipped with a cryoprobe [5mm CPDCH ^{13}C^{1}H/D] (600 MHz). Chemical shifts are reported in parts per million (ppm) from high to low frequency using the residual solvent peak as the internal reference ($CDCl_3$ = 7.26 ppm, MeOD-d_4 = 3.31 ppm, DMSO-d_6 = 2.50 ppm and MeCN-d_3 = 1.94 ppm). All ^{1}H resonances are reported to the nearest 0.01 ppm. The multiplicity of ^{1}H signals are indicated as: s = singlet; d = doublet; t = triplet; q = quartet; m = multiplet; or combinations of thereof. Coupling constants (*J*) are quoted in Hz and reported to the nearest 0.1 Hz. Where appropriate, averages of the signals from peaks displaying multiplicity were used to calculate the value of the coupling constant. ^{13}C NMR spectra were recorded on the same spectrometer with the central resonance of the solvent peak as the internal reference (MeCN-d_3 = 118.26 ppm, $CDCl_3$ = 77.16 ppm, DMSO-d_6 = 39.52 ppm and MeOD-d_4 = 49.00 ppm). All ^{13}C resonances are reported to the nearest 0.01 ppm. DEPT, COSY, HSQC and HMBC experiments were used to aid structural determination and spectral assignment. DOSY experiments were carried out on the same spectrometer. DOSY measurements were performed using the standard Bruker pulse program dstebpgp3s. Smoothed square gradients were used with a total duration of 1.6 ms. The gradient recovery delay was 200 μs, and diffusion time Δ = 10 ms was used for the experiments. Ten gradient increments were acquired, ranging from 5.9–53.2 G/cm in equal steps of gradient squared. Flash column chromatography was carried out using Silica 60 Å (particle size 40–63 μm, Sigma Aldrich, UK) as the stationary phase. Size exclusion chromatography was carried out using Sephadex LH-20 and Bio-Beads SX-1 support beads as the stationary phase. TLC was performed on precoated silica gel plates (0.25 mm thick, 60 F_{254}, Merck, Germany) and visualized using both short and long wave ultraviolet light. Low resolution ESI mass spectrometry was performed with a Thermo Scientific LCQ Fleet Ion Trap Mass Spectrometer or an Agilent Technologies 1200 LC system with either an Agilent 6130 single quadrupole MS detector or an Advion Expression LCMS single quadrupole MS detector. CD and UV/Vis spectroscopy was measured on an Applied Photophysics Ltd Chirascan CD Spectrometer. High-resolution mass spectrometry (HRMS) and MALDI-TOF (matrix assisted laser desorption/ionization time-of-flight) were carried out by staff at the Mass Spectrometry Service, Department of Chemistry, The University of Manchester. Compounds **S1**,[2] $(R)_2$-**S5**,[3] $(R)_2$-**S6**,[4] $(R)_4$-**L4**,[4] $(S)_4$-**L4**,[4] $(R)_8$-**L5**,[4] $(S)_2(R)_6$-**L5**,[4] (Λ,Λ)-($(R)_4$-**L4**$)_3$•$[Lu]_2$[4] and ($Λ_3$,Λ)-($(R)_8$-**L5**$)_3$•$[Lu]_4$[4] were prepared as previously reported.

S3. Reaction Schemes

S3.1 Synthesis of ligands

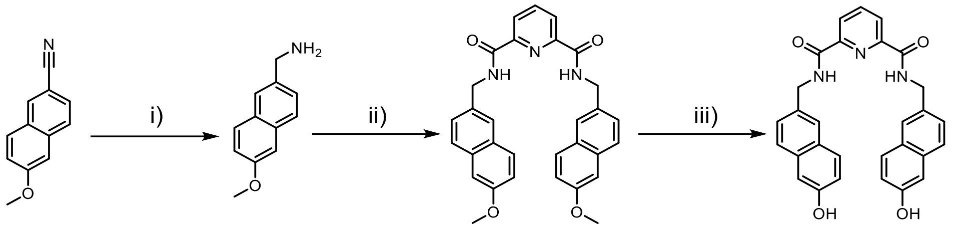

S1 **S2** **S3**

Scheme S1. Synthesis of building block **S3**: i) $LiAlH_4$, THF, 0 °C to r.t., 16 h, 99%; ii) 2,6-pyridinedicarbonyl dichloride, Et_3N, CH_2Cl_2, 0 °C to r.t., 24 h, 72%; iii) BBr_3, CH_2Cl_2, −78 °C to r.t., 3 h, 59%.

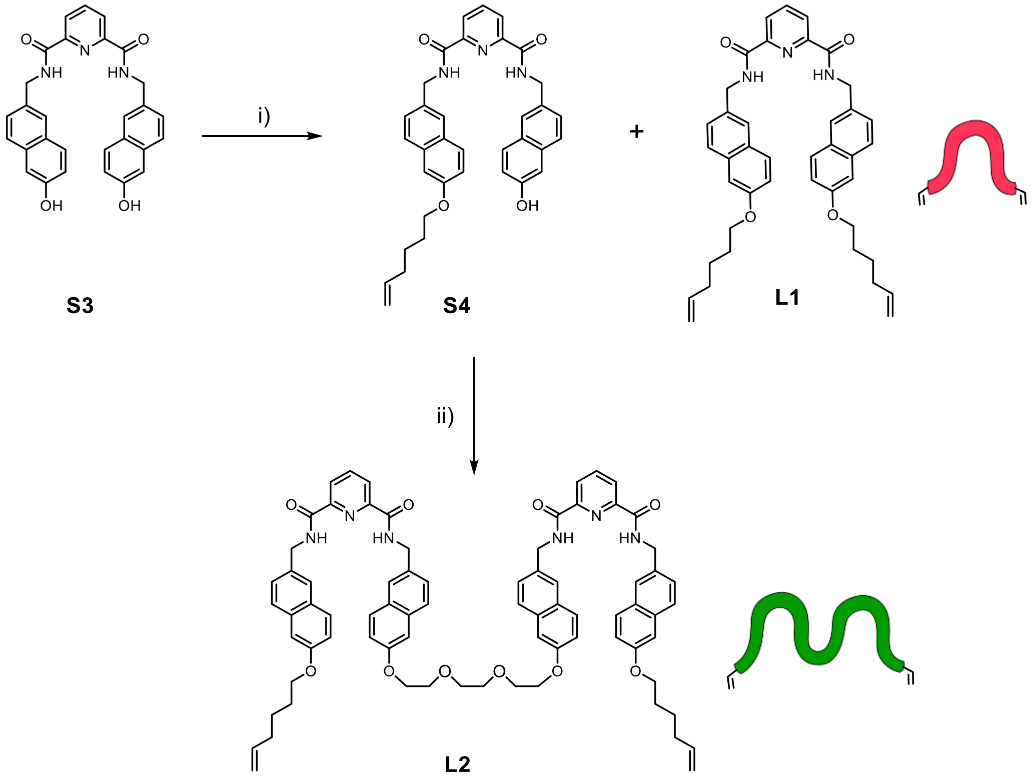

Scheme S2. Synthesis of achiral monotopic ligand **L1** and ditopic ligand **L2**: i) 6-bromohex-1-ene, K_2CO_3, DMF, 80 °C, 16 h, 38% intermediate **S4** and 33% ligand **L1**; ii) 1,2-bis(2-bromoethoxy)ethane, K_2CO_3, DMF, 80 °C, 24 h, 52%.

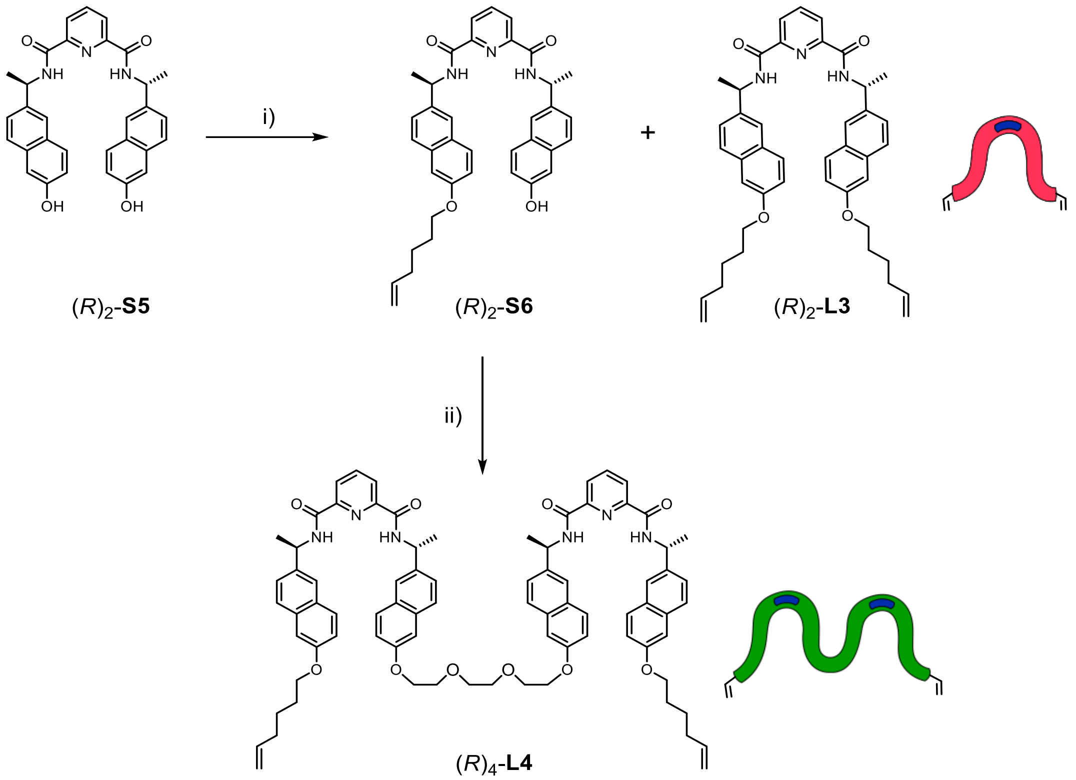

Scheme S3. Synthesis of chiral monotopic ligand (*R*)$_2$-**L3** and ditopic ligand (*R*)$_4$-**L4**: i) 6-bromohex-1-ene, K_2CO_3, DMF, 80 °C, 2 h, 43% intermediate (*R*)$_2$-**S6** and 23% ligand (*R*)$_2$-**L3**; ii) 1,2-bis(2-bromoethoxy)ethane, K_2CO_3, DMF, 80 °C, 24 h, 66%.

S3.2 Synthesis of achiral open complexes

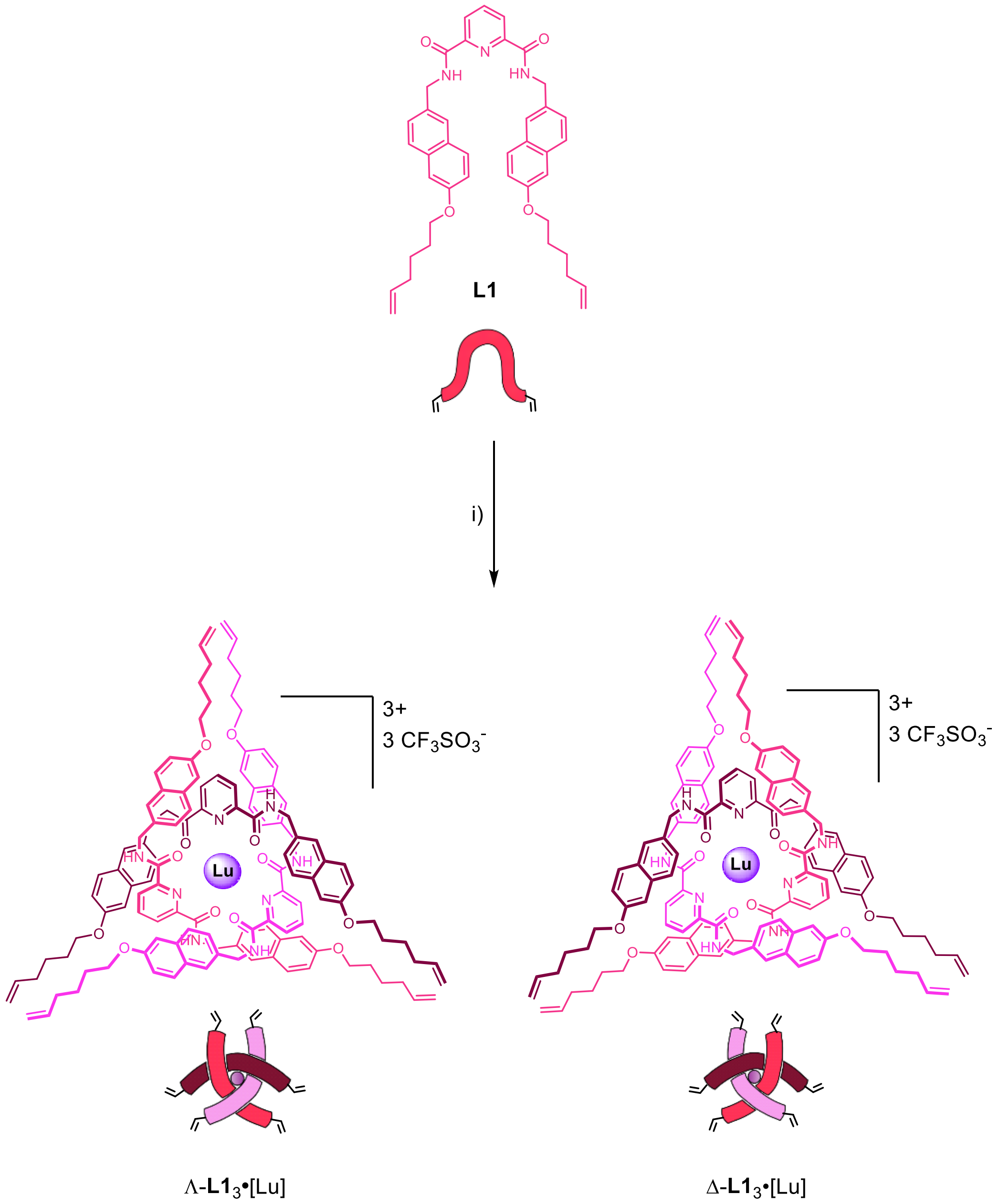

Scheme S4. Complexation of circular helicate mixture of **L1**$_3$•[Lu]: i) Lu(CF_3SO_3)$_3$, MeCN, 80 °C, 2 h, 85%.

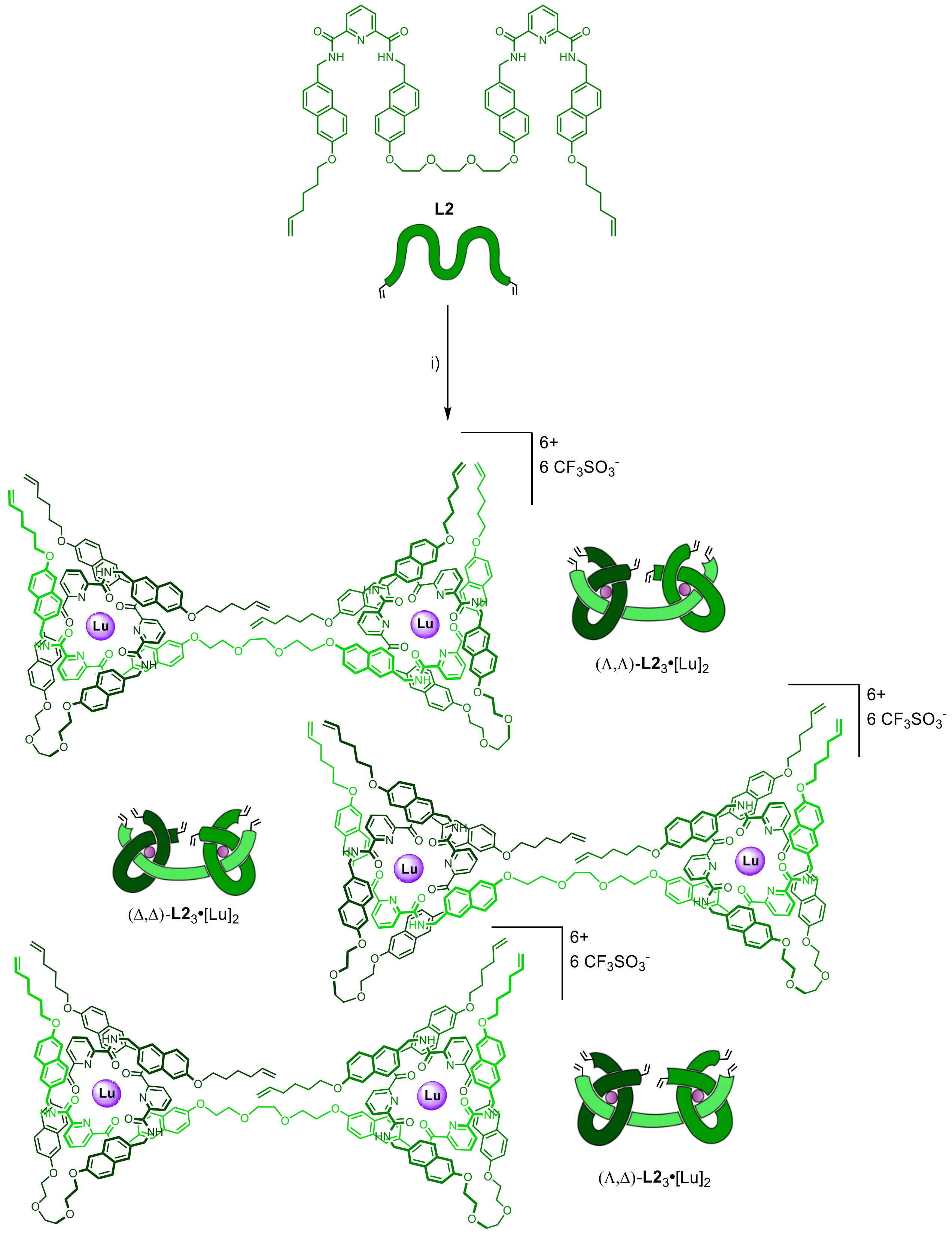

Scheme S5. Complexation of open granny and square knot mixture **L2**$_3$•[Lu]$_2$: i) $Lu(CF_3SO_3)_3$, MeCN, 80 °C, 2 days, 82% isolated crude complex.

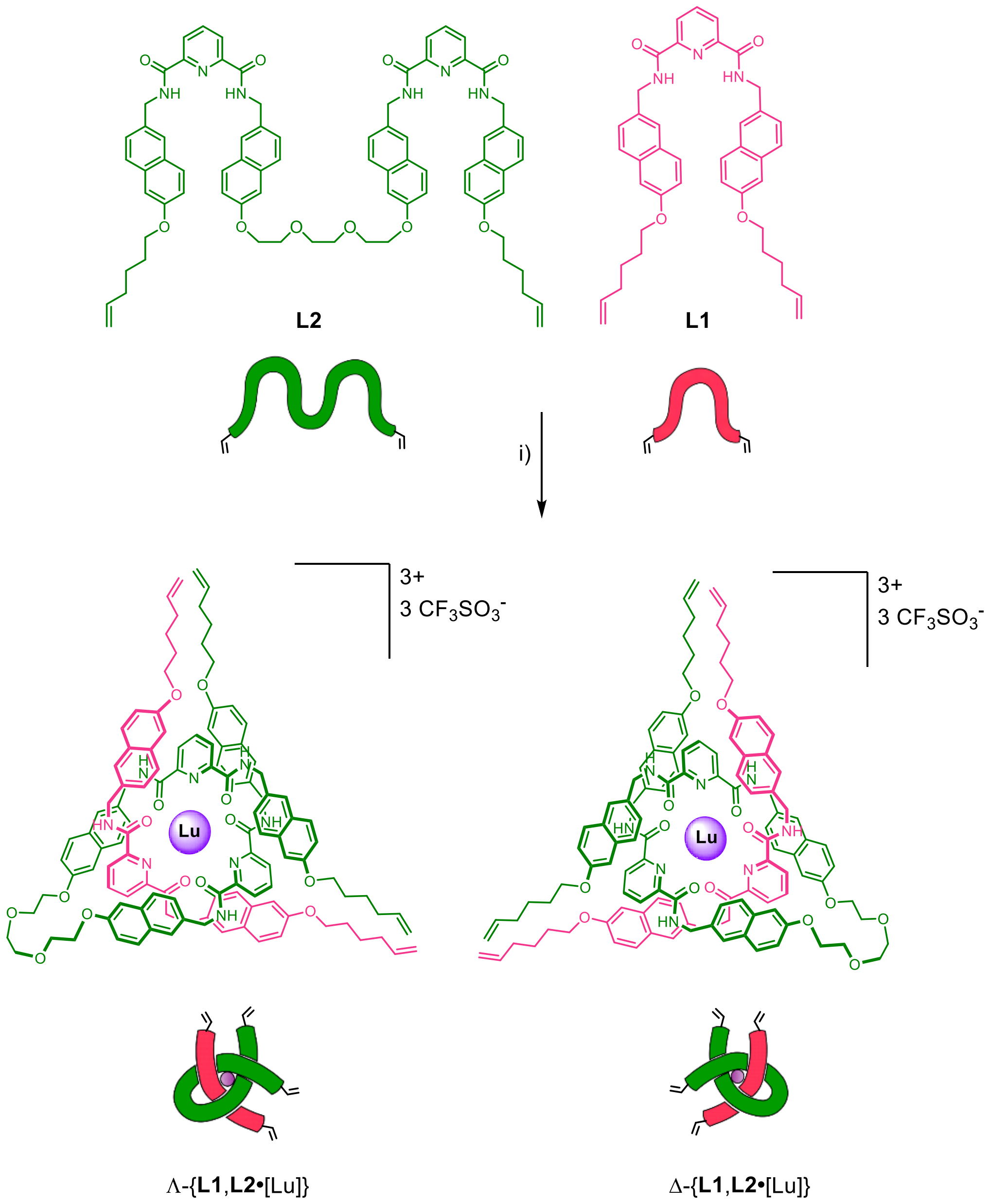

Scheme S6. Complexation of circular helicate mixture {**L1**,**L2•**[Lu]}: i) $Lu(CF_3SO_3)_3$, MeCN, 80 °C, 20 h, 92%.

S3.3 Synthesis of enantiopure Λ-handed open complexes

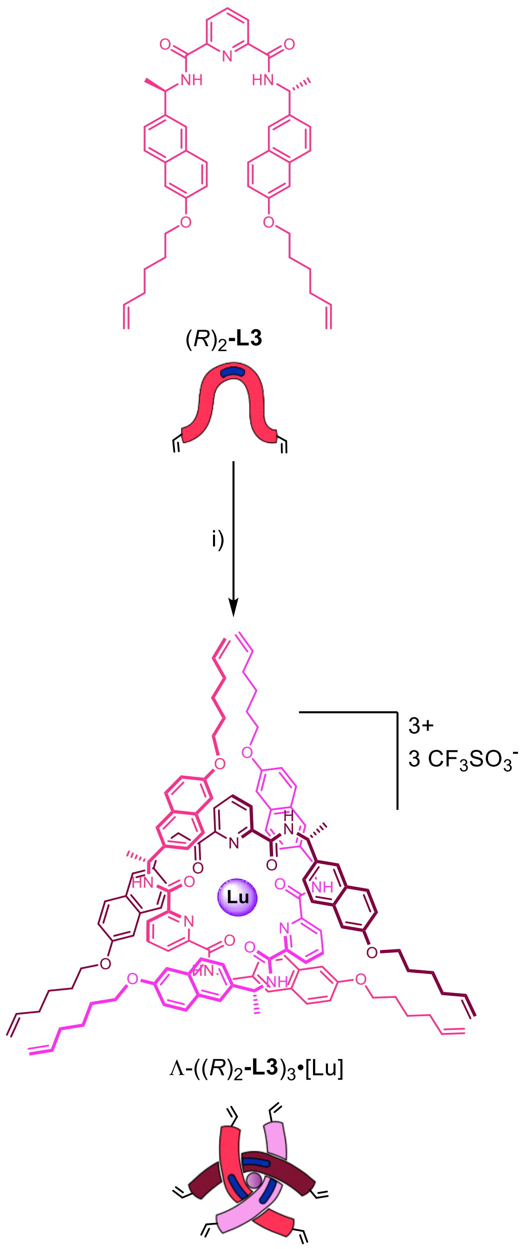

Scheme S7. Complexation of circular helicate Λ-((*R*)$_2$-**L3**)$_3$•[Lu]: i) Lu(CF_3SO_3)$_3$, MeCN-*d*$_3$, r.t., 24 h, 96%.

Homomeric open complexes (Λ,Λ)-((*R*)$_4$-L4)$_3$•[Lu]$_2$ and (Λ$_3$,Λ)-((*R*)$_8$-L5)$_3$•[Lu]$_4$ were prepared as previously reported.[4] Their structures are shown below (Fig. S1).

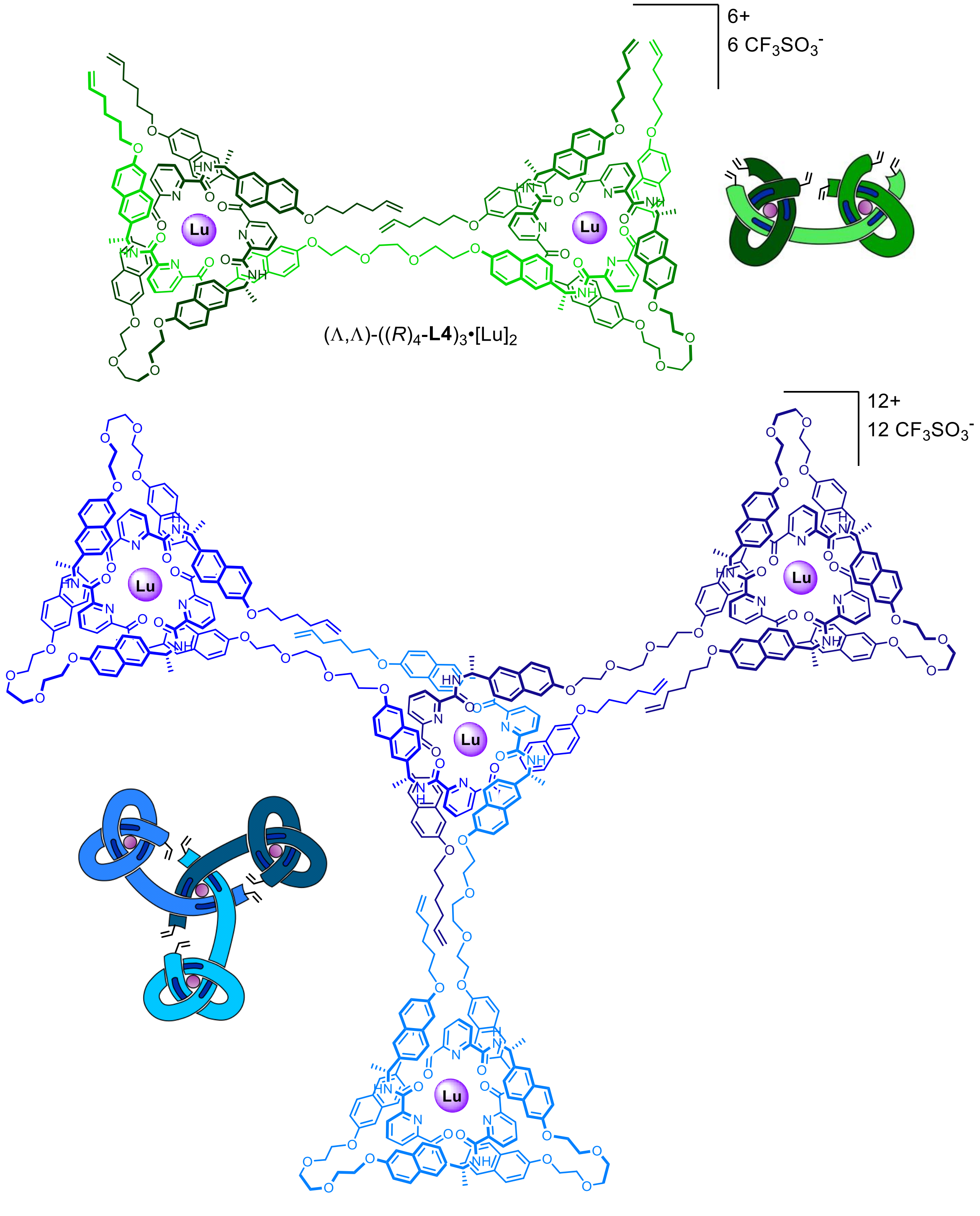

(Λ$_3$,Λ)-((*R*)$_8$-**L5**)$_3$•[Lu]$_4$

Figure S1. Homomeric open complexes (Λ,Λ)-((*R*)$_4$-**L4**)$_3$•[Lu]$_2$ and (Λ$_3$,Λ)-((*R*)$_8$-**L5**)$_3$•[Lu]$_4$.[4]

S3.4 Synthesis of trefoil knot Λ-1

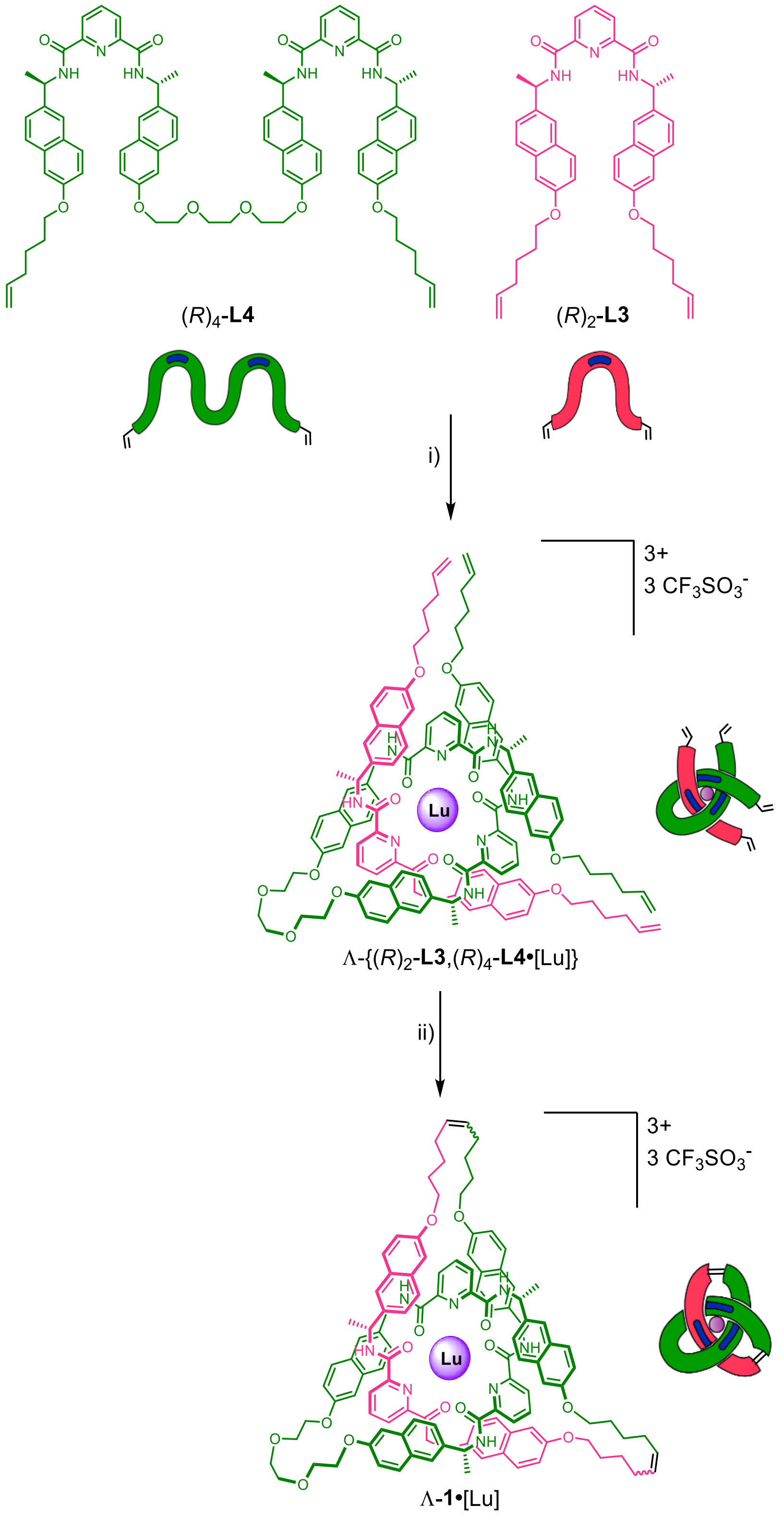

Scheme S8. Synthesis of trefoil knot Λ-**1**•[Lu]: i) $Lu(CF_3SO_3)_3$, MeCN, 80 °C, 24 h; ii) Hoveyda-Grubbs 2nd Generation catalyst, CH_2Cl_2:CH_3NO_2 1:1 (*v*/*v*), 50 °C, 16 h, 53% over two steps.

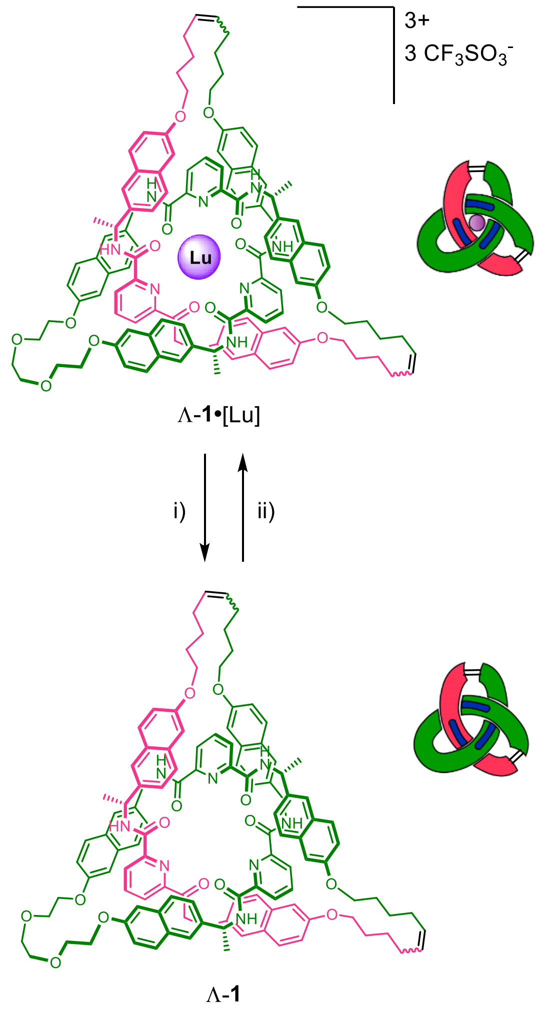

Scheme S9. Demetalation and remetalation of trefoil knot Λ-**1**•[Lu]: i) Et_4NF, MeCN, r.t., 0.5 h, 35% over three steps from ligands (*R*)$_2$-**L3** and (*R*)$_4$-**L4**; ii) $Lu(CF_3SO_3)_3$, MeCN, 80 °C, 24 h, 94%.

S3.5 Synthesis of granny knot (Λ,Λ)-2

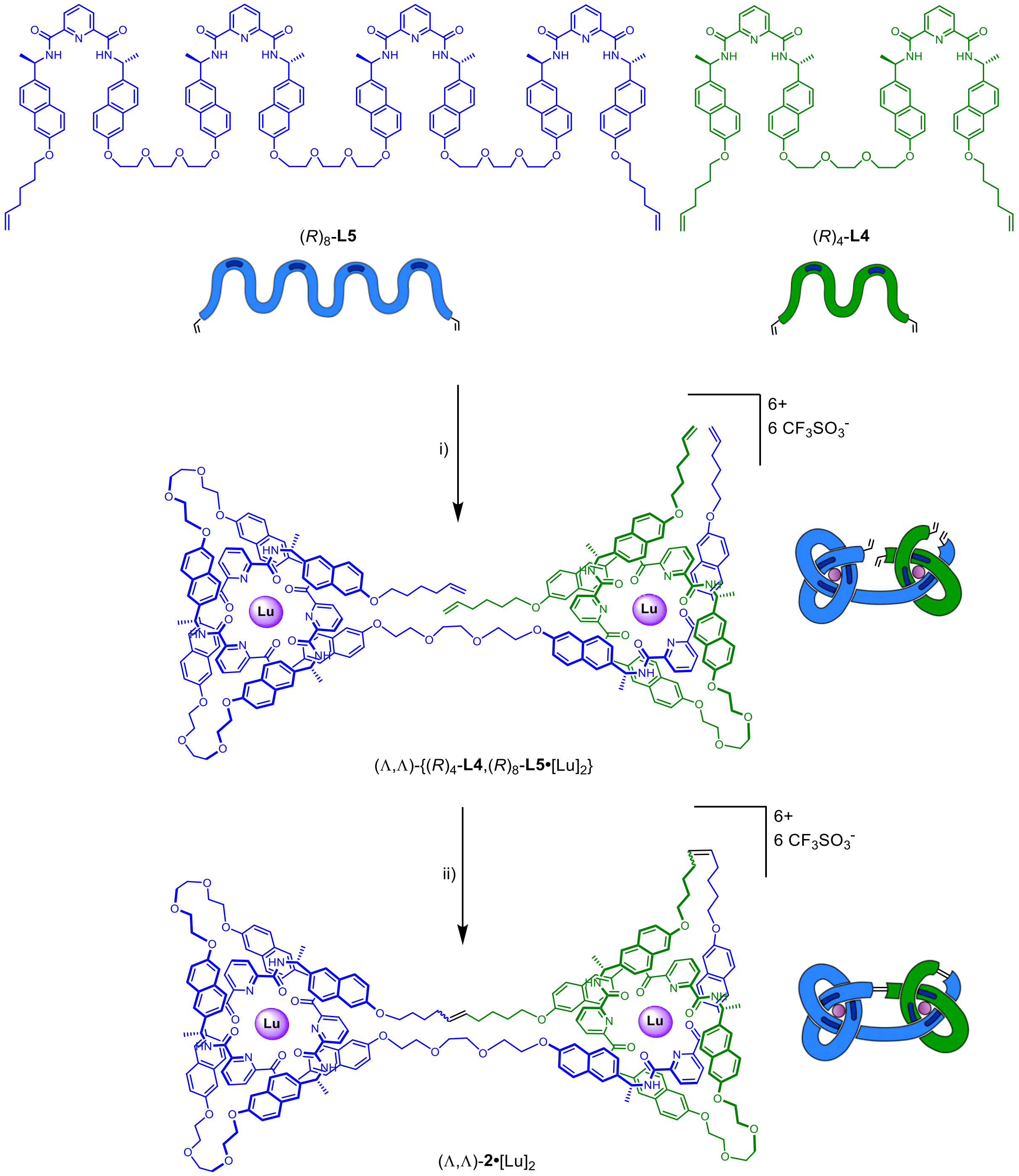

Scheme S10. Synthesis of granny knot (Λ,Λ)-**2**•[Lu]$_2$: i) Lu(CF_3SO_3)$_3$, MeCN, 80 °C, 72 h; ii) Hoveyda-Grubbs 2nd Generation catalyst, CH_2Cl_2:CH_3NO_2 1:1 (*v*/*v*), 50 °C, 24 h, 26% over two steps.

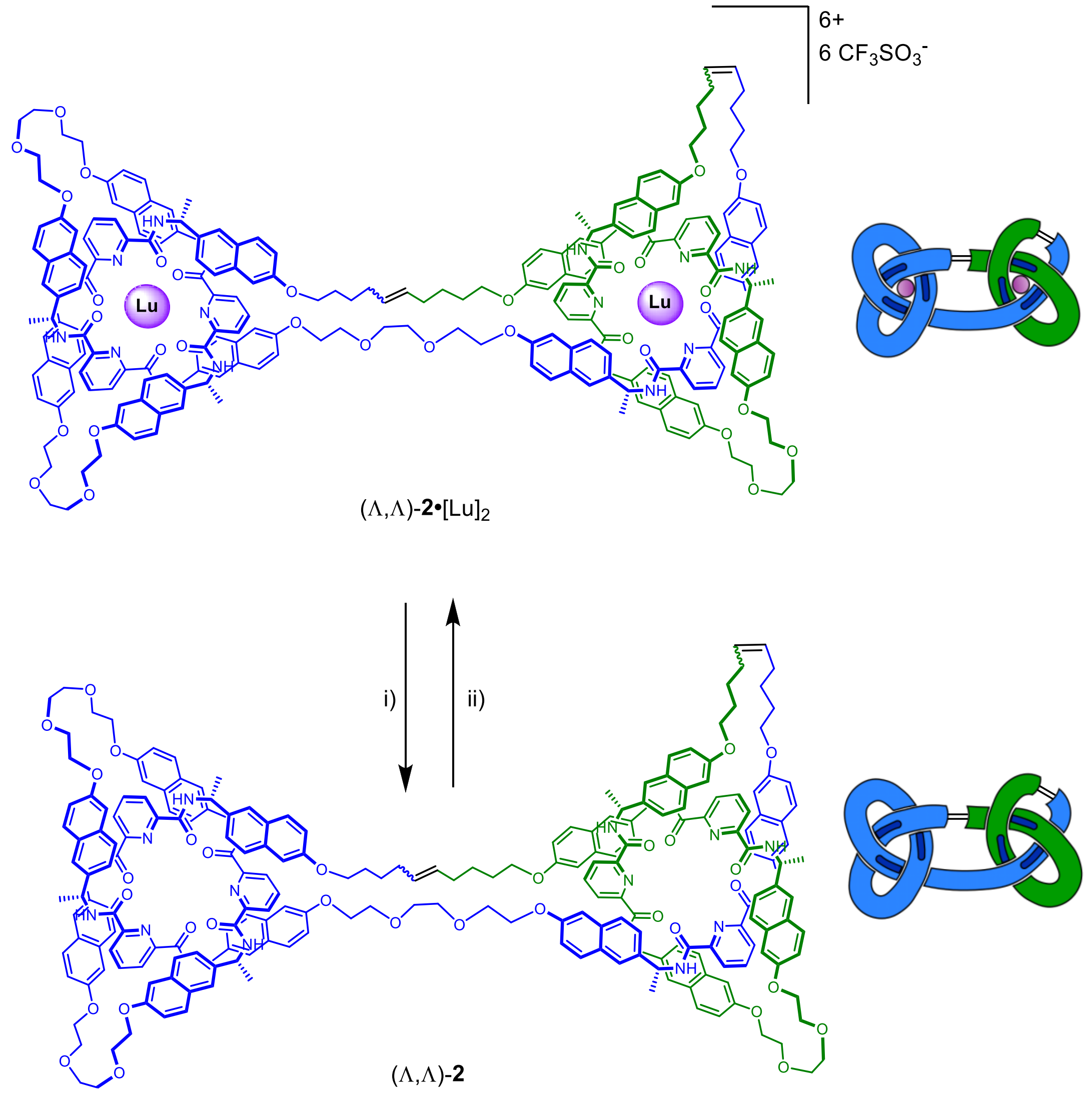

Scheme S11. Demetalation and remetalation of granny knot (Λ,Λ)-**2**: i) Et_4NF, MeCN, r.t., 0.5 h, 13% over three steps from ligands (*R*)$_4$-**L4** and (*R*)$_8$-**L5**; ii) $Lu(CF_3SO_3)_3$, MeCN, 80 °C, 24 h, 68%.

S3.6 Synthesis of square knot (Λ,Δ)-2

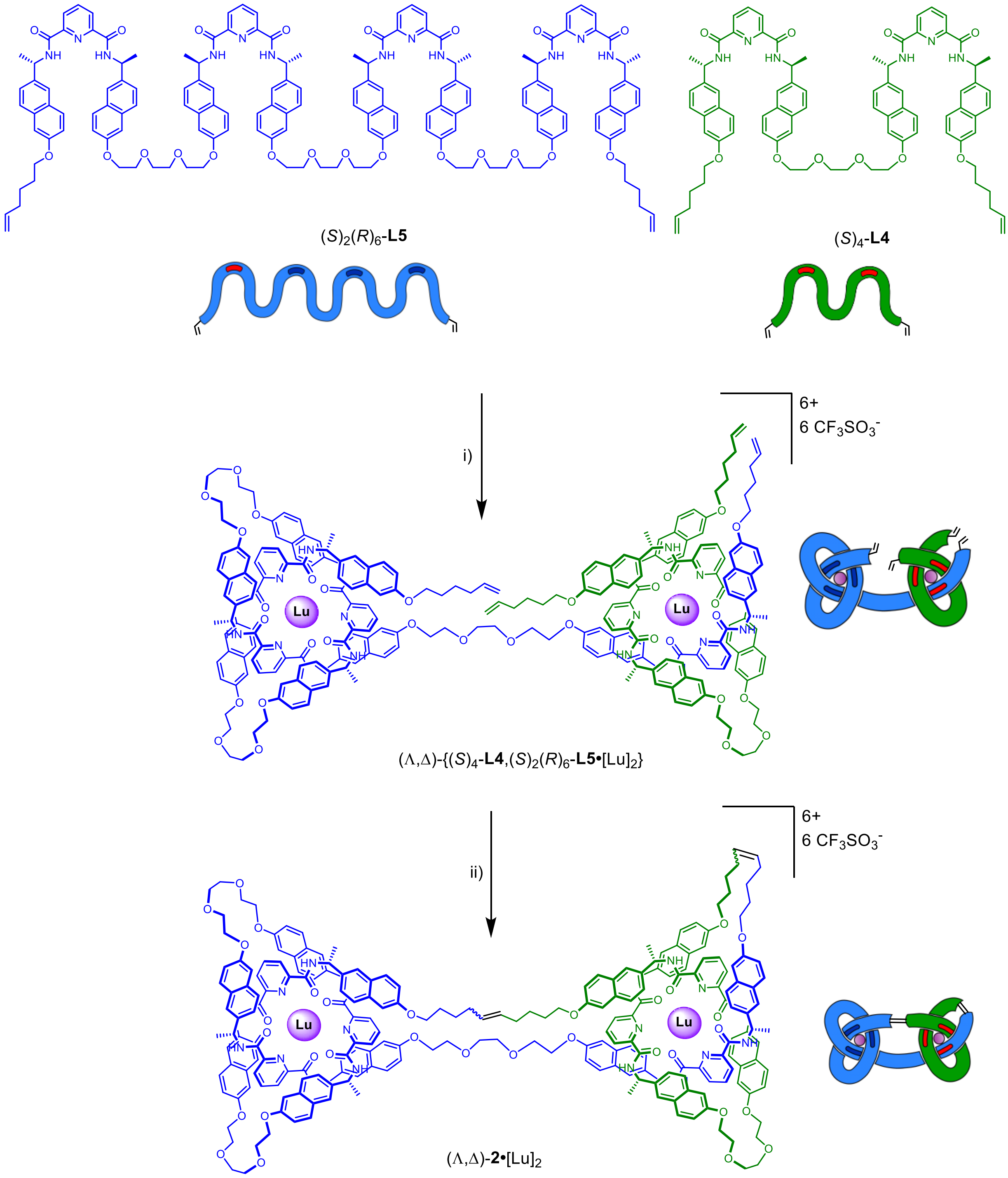

Scheme S12. Synthesis of square knot (Λ,Δ)-**2**•[Lu]$_2$: i) Lu(CF_3SO_3)$_3$, MeCN, 80 °C, 72 h; ii) Hoveyda-Grubbs 2nd Generation catalyst, CH_2Cl_2:CH_3NO_2 1:1 (*v*/*v*), 50 °C, 24 h, 29% over two steps.

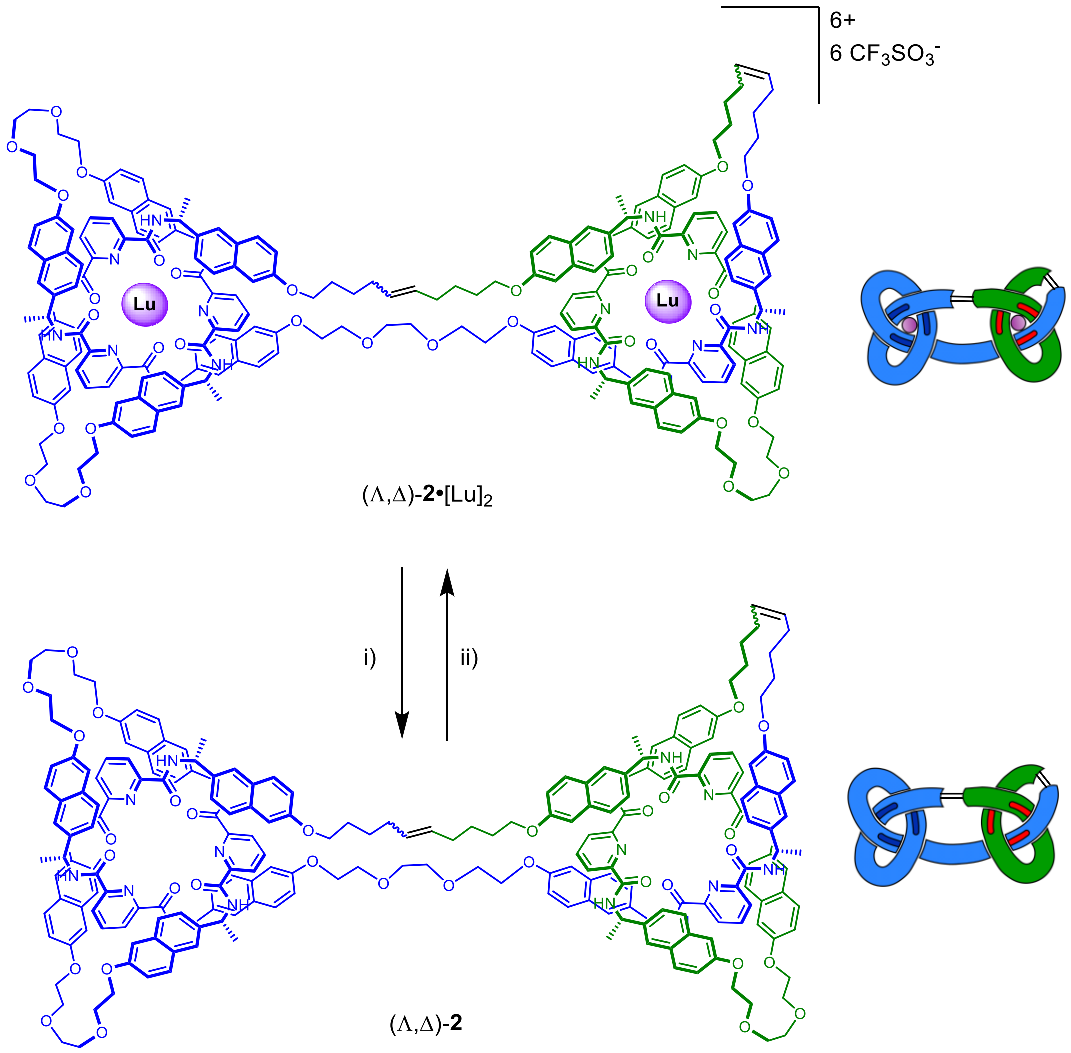

Scheme S13. Demetalation and remetalation of square knot (Λ,Δ)-**2**: i) Et_4NF, MeCN, r.t., 0.5 h, 7% over three steps from ligands (*S*)$_4$-**L4** and (*S*)$_2$(*R*)$_6$-**L5**; ii) $Lu(CF_3SO_3)_3$, MeCN, 80 °C, 40 h, 75%.

S3.7 In-situ rearrangement of helicate Λ-((R)$_2$-L3)$_3$•[Lu] and granny complex (Λ,Λ)-((R)$_4$-L4)$_3$•[Lu]$_2$ to form helicate Λ-{(R)$_2$-L3,(R)$_4$-L4•[Lu]}

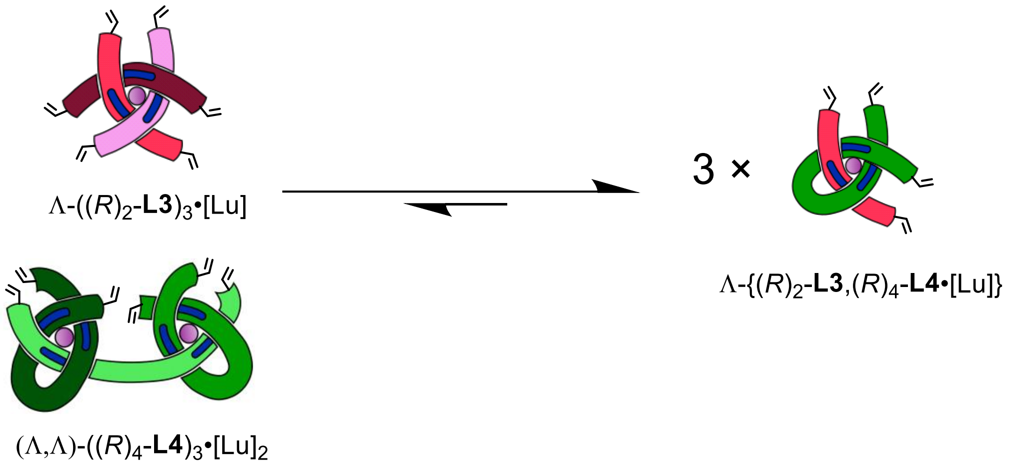

Scheme S14. Rearrangement of circular helicate Λ-((R)$_2$-**L3**)$_3$•[Lu] and open granny knot complex (Λ,Λ)-((R)$_4$-**L4**)$_3$•[Lu]$_2$ to give circular helicate Λ-{(R)$_2$-**L3**,(R)$_4$-**L4**•[Lu]}: MeCN-d_3, 80 °C, 4 h.

S3.8 In-situ rearrangement of triskelion complex (Λ$_3$,Λ)-((R)$_8$-L5)$_3$•[Lu]$_4$ and granny complex (Λ,Λ)-((R)$_4$-L4)$_3$•[Lu]$_2$ to form granny complex (Λ,Λ)-{(R)$_4$-L4,(R)$_8$-L5•[Lu]$_2$}

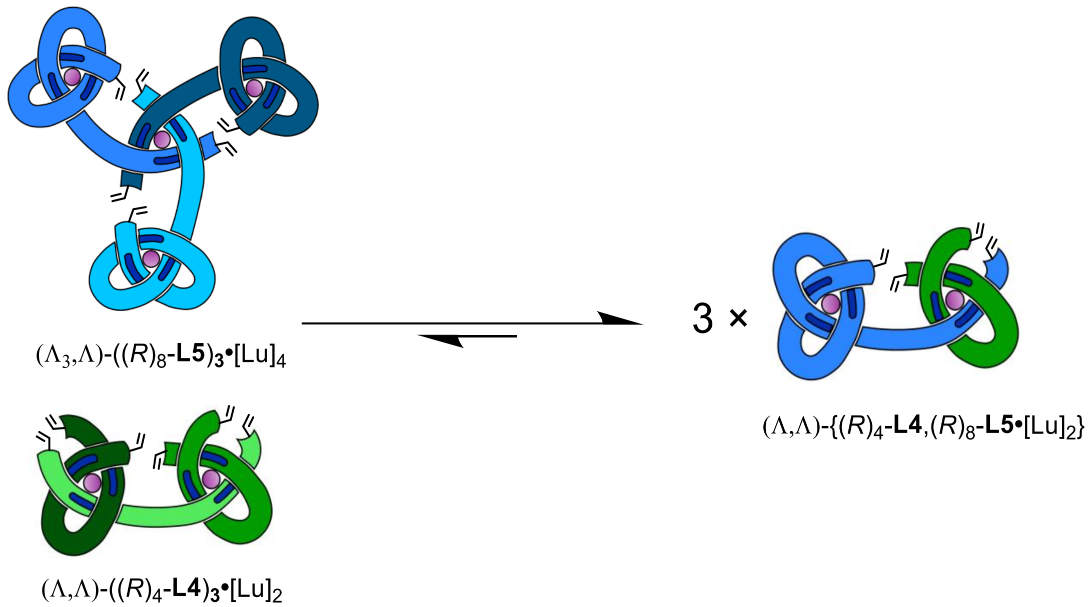

Scheme S15. Rearrangement of open triskelion knot complex (Λ$_3$,Λ)-((R)$_8$-**L5**)$_3$•[Lu]$_4$ and open granny knot complex (Λ,Λ)-((R)$_4$-**L4**)$_3$•[Lu]$_2$ to give open granny knot complex (Λ,Λ)-{(R)$_4$-**L4**,(R)$_8$-**L5**•[Lu]$_2$}: MeCN-d_3, 1 h at r.t. then 16 h at 80 °C.

S4. Experimental Procedures

S4.1 Synthesis of ligands

S2

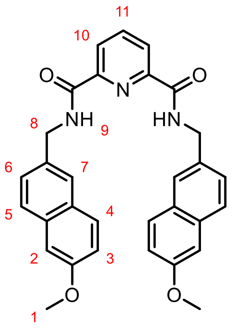

To a solution of **S1** (2.10 g, 11.2 mmol) and Et_3N (2.35 mL, 16.8 mmol) in anhydrous CH_2Cl_2 (105 mL) at 0 °C was added 2,6-pyridinedicarbonyl dichloride (1.14 g, 5.61 mmol) portionwise. After stirring the reaction mixture for 24 hours at room temperature, the reaction was washed with 1 M aq. HCl (2 x 300 mL). After back-extracting the aqueous layer with CH_2Cl_2 (2 × 300 mL), the combined organic phases were then washed with brine (300 mL) before being dried with Na_2SO_4, filtered and concentrated under reduced pressure to obtain an orange solid. MeOH (50 mL) was added to the crude product and the precipitate was filtered under vacuum. The filtrate was recombined with the remaining crude product in the flask, concentrated under reduced pressure and the trituration process with MeOH was repeated to yield the pure compound **S2** as an off-white solid (2.06 g, 72%). **^{1}H NMR** (600 MHz, $CDCl_3$) δ 8.38 (d, J = 7.8 Hz, 2H, H_{10}), 8.09 (t, J = 6.2 Hz, 2H, H_9), 8.02 (t, J = 7.8 Hz, 1H, H_{11}), 7.59 (s, 2H, H_7), 7.58 – 7.54 (m, 4H, $H_{4,5}$), 7.35 (dd, J = 8.4, 1.8 Hz, 2H, H_6), 7.08 (dd, J = 8.9, 2.5 Hz, 2H, H_3), 7.04 (d, J = 2.5 Hz, 2H, H_2), 4.74 (d, J = 6.1 Hz, 4H, H_8), 3.89 (s, 6H, H_1). **^{13}C NMR** (151 MHz, $CDCl_3$) δ 163.56, 157.88, 148.90, 139.16, 134.03, 133.14, 129.28, 128.81, 127.56, 126.52, 126.51, 125.51, 119.30, 105.75, 55.44, 43.73. **HRMS** (ESI$^-$): Calcd. for $C_{31}H_{26}O_4N_3^-$: 504.1929, found 504.1929 [M−H]$^-$.

S3

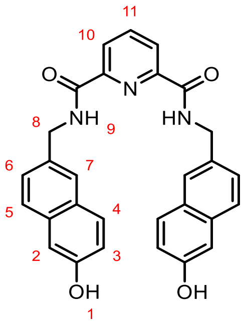

To a solution of **S2** (1.00 g, 1.98 mmol) in anhydrous CH_2Cl_2 (15 mL) at −78 °C was added BBr_3 (1 M solution in CH_2Cl_2, 9.89 mL, 9.89 mmol) dropwise. The mixture was stirred for 30 minutes before being warmed to room temperature and stirred for a further 2 hours. Sat. aq. $NaHCO_3$ (10 mL) was added slowly at 0 °C to quench the reaction. The solution was washed with MeOH (30 mL) and filtered under vacuum to leave an orange solid. The crude product was dried under vacuum before purification by flash column chromatography (CH_2Cl_2 to EtOAc) yielded compound **S3** as a colorless solid (560 mg, 59%). **^{1}H NMR** (600 MHz, MeOD-*d*$_4$) δ 8.30 (d, *J* = 7.8 Hz, 2H, H_{10}), 8.14 (t, *J* = 7.8 Hz, 1H, H_{11}), 7.64 (s, 2H, H_7), 7.61 (d, *J* = 8.8 Hz, 2H, H_4), 7.57 (d, *J* = 8.5 Hz, 2H, H_5), 7.35 (dd, *J* = 8.5, 1.8 Hz, 2H, H_6), 7.05 (d, *J* = 2.5 Hz, 2H, H_2), 7.02 (dd, *J* = 8.8, 2.3 Hz, 2H, H_3), 4.71 (s, 4H, H_8). **^{13}C NMR** (151 MHz, MeOD-*d*$_4$) δ 165.93, 156.45, 150.32, 140.57, 135.64, 134.22, 130.28, 129.65, 127.70, 127.12, 126.94, 125.99, 119.55, 109.82, 44.24. **HRMS** (ESI$^+$): Calcd. for $C_{29}H_{24}O_4N_3^+$: 478.1761, found 478.1766 $[M+H]^+$.

S4

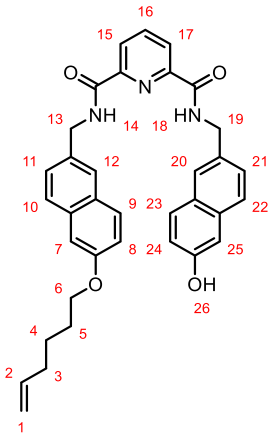

To a solution of **S3** (500 mg, 1.05 mmol) and potassium carbonate (434 mg, 3.14 mmol) in degassed DMF (100 mL) was added 6-bromohex-1-ene (139 µL, 1.05 mmol). The reaction was stirred for 16

hours at 80 °C. The mixture was cooled to room temperature and concentrated under reduced pressure. Purification by flash column chromatography (CH_2Cl_2/EtOAc 3:1) yielded compound **S4** as a colorless solid (223 mg, 38%). **^{1}H NMR** (600 MHz, $CDCl_3$) δ 8.43 (d, J = 7.8 Hz, 2H, $H_{15,17}$), 8.07 (t, J = 7.8 Hz, 1H, H_{16}), 8.01 – 7.97 (m, 2H, $H_{14,18}$), 7.65 (s, 2H, $H_{12,20}$), 7.61 (d, J = 8.5 Hz, 3H, $H_{9,22,23}$), 7.58 (d, J = 8.5 Hz, 1H, H_{10}), 7.36 (dd, J = 8.5, 2.5 Hz, 2H, $H_{11,21}$), 7.11 (dd, J = 8.5, 2.5 Hz, 2H, $H_{8,24}$), 7.09 – 7.02 (m, 2H, $H_{7,25}$), 5.85 (ddt, J = 16.9, 10.2, 6.6 Hz, 1H, H_2), 5.05 (dd, J = 17.1, 1.8 Hz, 1H, $H_{1(trans)}$), 4.99 (dd, J = 10.2, 1.8 Hz, 1H, $H_{1(cis)}$), 4.77 (d, J = 6.2 Hz, 4H, $H_{13,19}$), 4.07 (t, J = 6.5 Hz, 2H, H_6), 2.16 (q, J = 6.8 Hz, 2H, H_3), 1.87 (quint, J = 6.8 Hz, 2H, H_5), 1.62 (quint, J = 7.5 Hz, 2H, H_4). **^{13}C NMR** (151 MHz, $CDCl_3$) δ 163.57, 163.54, 157.37, 153.68, 148.94, 148.92, 139.23, 138.66, 134.11, 134.03, 133.14, 133.00, 129.74, 129.26, 128.78, 127.55, 127.21, 126.65, 126.60, 126.55, 126.50, 125.58, 125.55, 119.61, 118.36, 114.97, 68.01, 43.79, 43.76, 33.61, 28.82, 25.53. Due to the pseudo-symmetry, several carbon signals are not resolved. **HRMS** (ESI$^-$): Calcd. for $C_{35}H_{32}O_4N_3^-$: 558.2398, found 558.2396 [M−H]$^-$.

L1

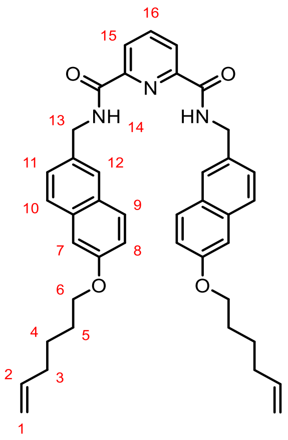

Monotopic ligand **L1** was isolated as a side product from the reaction of 6-bromohex-1-ene and **S3** to form **S4**, as a colorless solid in 33% yield. **^{1}H NMR** (600 MHz, $CDCl_3$) δ 8.41 (d, J = 7.8 Hz, 2H, $H_{15,17}$), 8.08 – 8.00 (m, 3H, $H_{14,16}$), 7.63 (s, 2H, H_{12}), 7.60 (dd, J = 8.8, 1.8 Hz, 4H, $H_{9,10}$), 7.36 (dd, J = 8.5, 1.8 Hz, 2H, H_{11}), 7.10 (dd, J = 8.9, 2.5 Hz, 2H, H_8), 7.06 (d, J = 2.5 Hz, 2H, H_7), 5.84 (ddt, J = 16.9, 10.2, 6.7 Hz, 2H, H_2), 5.05 (dd, J = 17.1, 1.7 Hz, 2H, $H_{1(trans)}$), 4.99 (dd, J = 10.2, 1.5 Hz, 2H, $H_{1(cis)}$), 4.76 (d, J = 6.2 Hz, 4H, H_{13}), 4.06 (t, J = 6.5 Hz, 4H, H_6), 2.16 (q, J = 7.2 Hz, 4H, H_3), 1.86 (quint, J = 6.6 Hz, 4H, H_5), 1.62 (quint, J = 7.5 Hz, 4H, H_4). **^{13}C NMR** (151 MHz, $CDCl_3$) δ 163.57, 157.38, 148.93, 139.17, 138.66, 134.10, 133.04, 129.23, 128.76, 127.54, 126.50, 126.48, 125.54, 119.61, 114.96, 106.53, 67.94, 43.74, 33.61, 28.82, 25.52. Due to the pseudo-symmetry, several carbon signals are not resolved. **HRMS** (ESI$^+$): Calcd. for $C_{41}H_{43}O_4N_3Na^+$: 664.3146, found 664.3144 [M+Na]$^+$.

L2

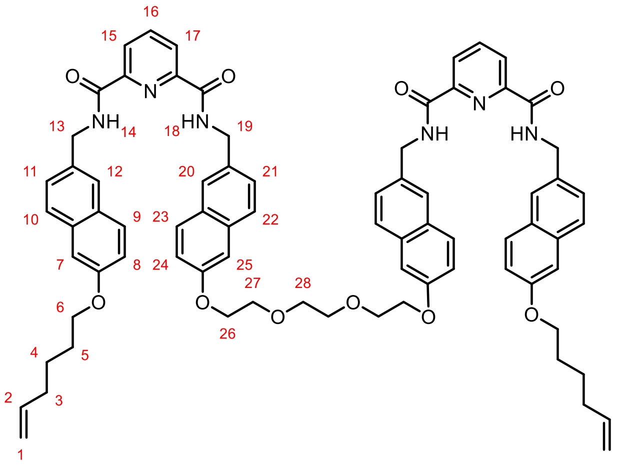

To a solution of **S4** (100 mg, 179 µmol) and potassium carbonate (37.0 mg, 268 µmol) in degassed DMF (9 mL) was added 1,2-bis(2-bromoethoxy)ethane (14.7 µL, 89.3 µmol). The reaction was stirred for 24 hours at 80 °C. The mixture was cooled to room temperature and concentrated under reduced pressure. Purification by flash column chromatography (CH_2Cl_2/EtOAc 2:1) yielded compound **L2** as a colorless solid (57.8 mg, 52%). **^{1}H NMR** (600 MHz, $CDCl_3$) δ 8.40 – 8.29 (m, 8H, $H_{14,15,17,18}$), 7.95 (t, J = 7.8 Hz, 2H, H_{16}), 7.46 – 7.32 (m, 12H, $H_{9,10,12,20,22,23}$), 7.17 (dd, J = 8.4, 1.8 Hz, 2H, $H_{11/21}$), 7.08 (dd, J = 8.4, 1.7 Hz, 2H, $H_{11/21}$), 7.02 (dd, J = 8.9, 2.4 Hz, 2H, $H_{8/24}$), 6.96 (dd, J = 8.9, 2.5 Hz, 2H, $H_{8/24}$), 6.92 (d, J = 2.5 Hz, 4H, $H_{7,25}$), 5.84 (ddt, J = 16.9, 10.1, 6.6 Hz, 2H, H_2), 5.05 (dd, J = 17.1, 1.8 Hz, 2H, $H_{1(trans)}$), 4.98 (dd, J = 10.2, 2.0 Hz, 2H, $H_{1(cis)}$), 4.52 (d, J = 6.0 Hz, 4H, $H_{13/19}$), 4.49 (d, J = 6.1 Hz, 4H, $H_{13/19}$), 4.05 (t, J = 4.7 Hz, 4H, H_{26}), 4.00 (t, J = 6.5 Hz, 4H, H_6), 3.83 (t, J = 5.2 Hz, 4H, H_{27}), 3.74 (s, 4H, H_{28}), 2.15 (q, J = 7.3 Hz, 4H, H_3), 1.85 (quint, J = 6.7 Hz, 4H, H_5), 1.61 (quint, J = 7.7 Hz, 4H, H_4). **^{13}C NMR** (151 MHz, $CDCl_3$) δ 163.81, 163.77, 157.28, 156.97, 148.77, 138.97, 138.65, 133.91, 133.83, 133.06, 132.92, 129.09, 129.07, 128.73, 128.57, 127.42, 127.32, 126.41, 126.36, 126.28, 126.23, 125.30, 125.23, 119.48, 119.41, 114.97, 107.02, 106.38, 71.18, 69.99, 67.90, 67.56, 43.66, 43.62, 33.61, 28.83, 25.52. Due to the pseudo-symmetry, several carbon signals are not resolved. **HRMS** (ESI$^+$): Calcd. for $C_{76}H_{76}O_{10}N_6Na^+$: 1255.5515, found 1255.5511 $[M+Na]^+$.

(*R*)$_2$-**L3**

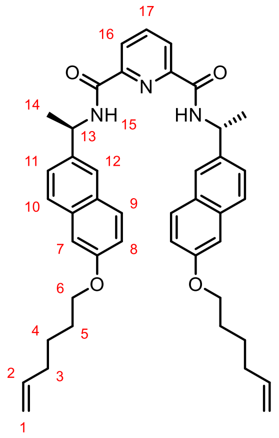

Monotopic ligand (*R*)$_2$-**L3** was isolated as a side product from the reaction of 6-bromohex-1-ene and (*R*)$_2$-**S5** to form compound (*R*)$_2$-**S6**, as a colorless solid in 23% yield. **^{1}H NMR** (600 MHz, $CDCl_3$) δ 8.38 (d, J = 7.7 Hz, 2H, H_{16}), 8.04 (t, J = 7.8 Hz, 1H, H_{17}), 7.92 (d, J = 8.1 Hz, 2H, H_{15}), 7.74 (s, 2H, H_{12}), 7.69 (d, J = 9.0 Hz, 2H, H_9), 7.65 (d, J = 8.5 Hz, 2H, H_{10}), 7.44 (dd, J = 8.4, 1.9 Hz, 2H, H_{11}), 7.18 (dd, J = 8.9, 2.5 Hz, 2H, H_8), 7.12 (d, J = 2.5 Hz, 2H, H_7), 5.91 – 5.84 (m, 2H, H_2), 5.46 (quint, J = 7.1 Hz, 2H, H_{13}), 5.08 (dd, J = 17.1, 1.7 Hz, 2H, $H_{1(trans)}$), 5.01 (dd, J = 10.2, 2.3 Hz, 2H, $H_{1(cis)}$), 4.11 (t, J = 6.5 Hz, 4H, H_6), 2.19 (q, J = 7.2 Hz, 4H, H_3), 1.89 (quint, J = 6.6 Hz, 4H, H_5), 1.67 (d, J = 7.1 Hz, 6H, H_{14}), 1.66 – 1.61 (m, 4H, H_4). **^{13}C NMR** (151 MHz, $CDCl_3$) δ 163.06, 156.89, 150.17, 139.08, 138.52, 137.68, 134.01, 129.31, 128.69, 127.50, 125.16, 124.86, 124.58, 119.57, 114.84, 106.40, 68.76, 49.05, 33.48, 27.66, 25.40, 20.85. Due to the pseudo-symmetry, several carbon signals are not resolved. **HRMS** (ESI^+): Calcd. for $C_{43}H_{47}O_4N_3Na^+$: 692.3459, found 692.3435 $[M+Na]^+$.

S4.2 Synthesis of achiral open complexes

L1$_3$•[Lu]

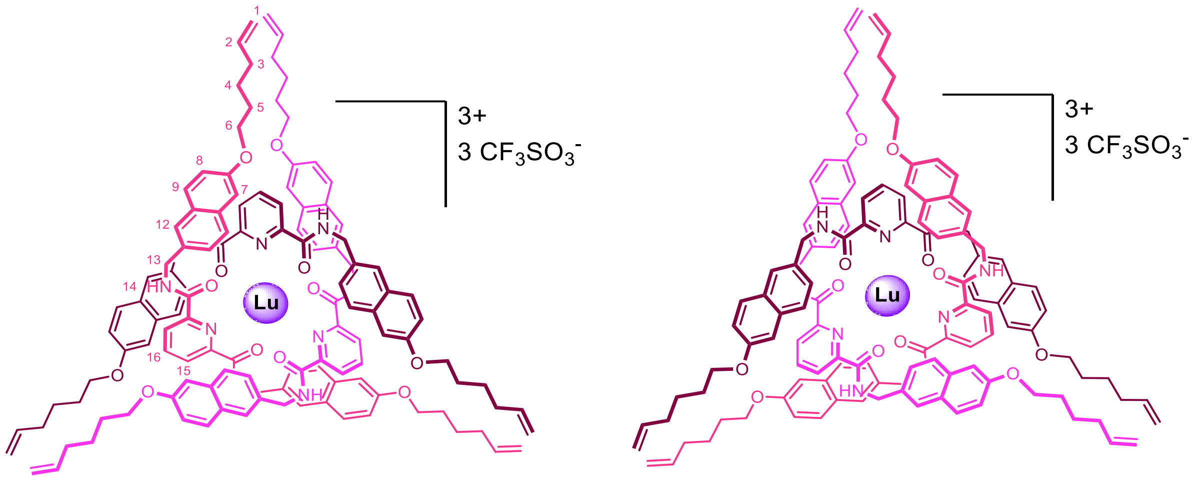

To a solution of **L1** (20.4 mg, 31.8 µmol) in MeCN (4.0 mL) was added $Lu(CF_3SO_3)_3$ (6.60 mg, 10.6 µmol) in MeCN (1.0 mL). The solution was stirred for 2 hours at 80 °C, cooled to room temperature and concentrated under reduced pressure. The resulting solid was washed twice with CH_2Cl_2 to give compound **L1**$_3$•[Lu] as an off-white solid (19.0 mg, 85%). **^{1}H NMR** (600 MHz, MeCN-d_3) δ 9.09 (t, J = 6.0 Hz, 6H, H_{14}), 7.68 (d, J = 8.0 Hz, 6H, H_{15}), 7.49 – 7.43 (m, 15H, $H_{9,10,16}$), 7.21 (s, 6H, H_{12}), 7.18 (d, J = 2.6 Hz, 6H, H_7), 7.14 (dd, J = 8.9, 2.5 Hz, 6H, H_8), 6.79 (dd, J = 8.5, 1.8 Hz, 6H, H_{11}), 5.92 (ddt, J = 17.0, 10.2, 6.7 Hz, 6H, H_2), 5.09 (dq, J = 17.2, 1.8 Hz, 6H, $H_{1(trans)}$), 5.01 (ddd, J = 10.2, 2.3, 1.2 Hz, 6H, $H_{1(cis)}$), 4.27 – 4.21 (m, 12H, H_{13}), 4.16 – 4.12 (m, 12H, H_6), 2.21 – 2.18 (m, 12H, H_3), 1.93 – 1.85 (m, 12H, H_5), 1.65 (quint, J = 7.5 Hz, 12H, H_4). **^{13}C NMR** (151 MHz, MeCN-d_3) δ 167.71, 157.86, 157.85, 145.87, 141.74, 139.38, 134.36, 132.43, 129.51, 128.81, 127.59, 125.63, 125.02, 119.98, 114.82, 107.17, 68.37, 44.69, 33.73, 29.01, 25.73. Due to the pseudo-symmetry, several carbon signals are not resolved. **HRMS** (ESI$^+$): Calcd. for $C_{123}H_{129}O_{12}N_9Lu^{3+}$: 699.6384, found 699.6358 $[M-3(CF_3SO_3)]^{3+}$.

L2$_3$•[Lu]$_2$

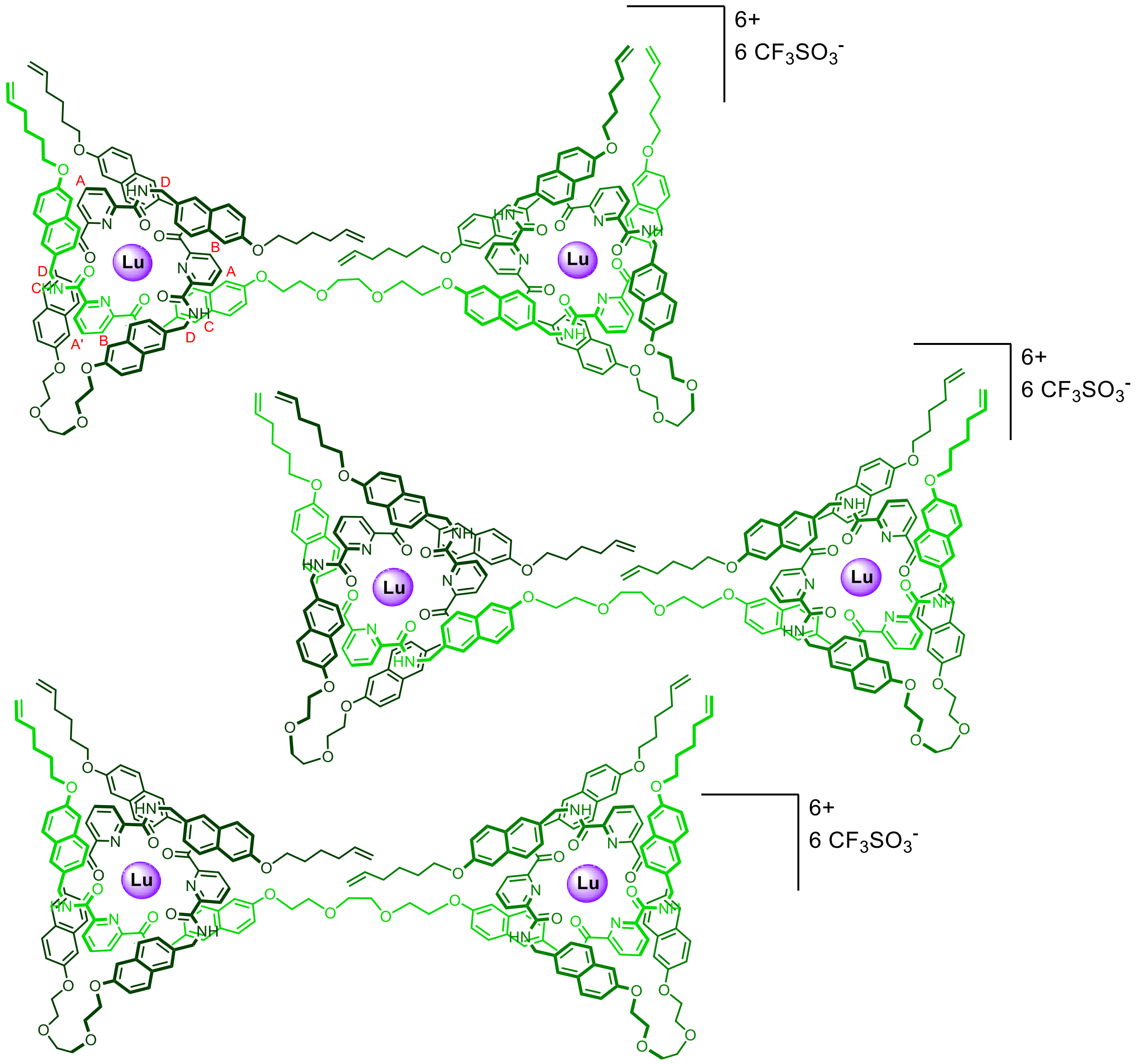

To a solution of **L2** (15.3 mg, 12.4 μmol) in MeCN (3.90 mL) was added a solution of $Lu(CF_3SO_3)_3$ (5.14 mg, 8.27 μmol) in MeCN (0.15 mL). The mixture was stirred for 2 days at 80 °C and monitored by ^{1}H NMR spectroscopy and ESI-MS until no further change was observed. The reaction mixture was cooled to room temperature, concentrated under reduced pressure and washed twice with CH_2Cl_2 to give compound **L2$_3$**•[Lu]$_2$ as a colorless solid (13.7 mg, 82%). The resulting ^{1}H NMR spectrum (Fig. S2) showed peaks consistent with previously reported open composite knot complexes.[4] Selected spectral assignments are shown below (see for example splitting of pyridine proton H_A to two distinct peaks, corresponding to the more shielded 'enclosed' side $H_{A'}$ and the less shielded 'open' side H_A). **HRMS** (ESI$^+$): Calcd. for $C_{229}H_{228}O_{33}N_{18}F_3SLu_2^{5+}$: 839.3023, found 839.3035 $[M-5(CF_3SO_3)]^{5+}$.

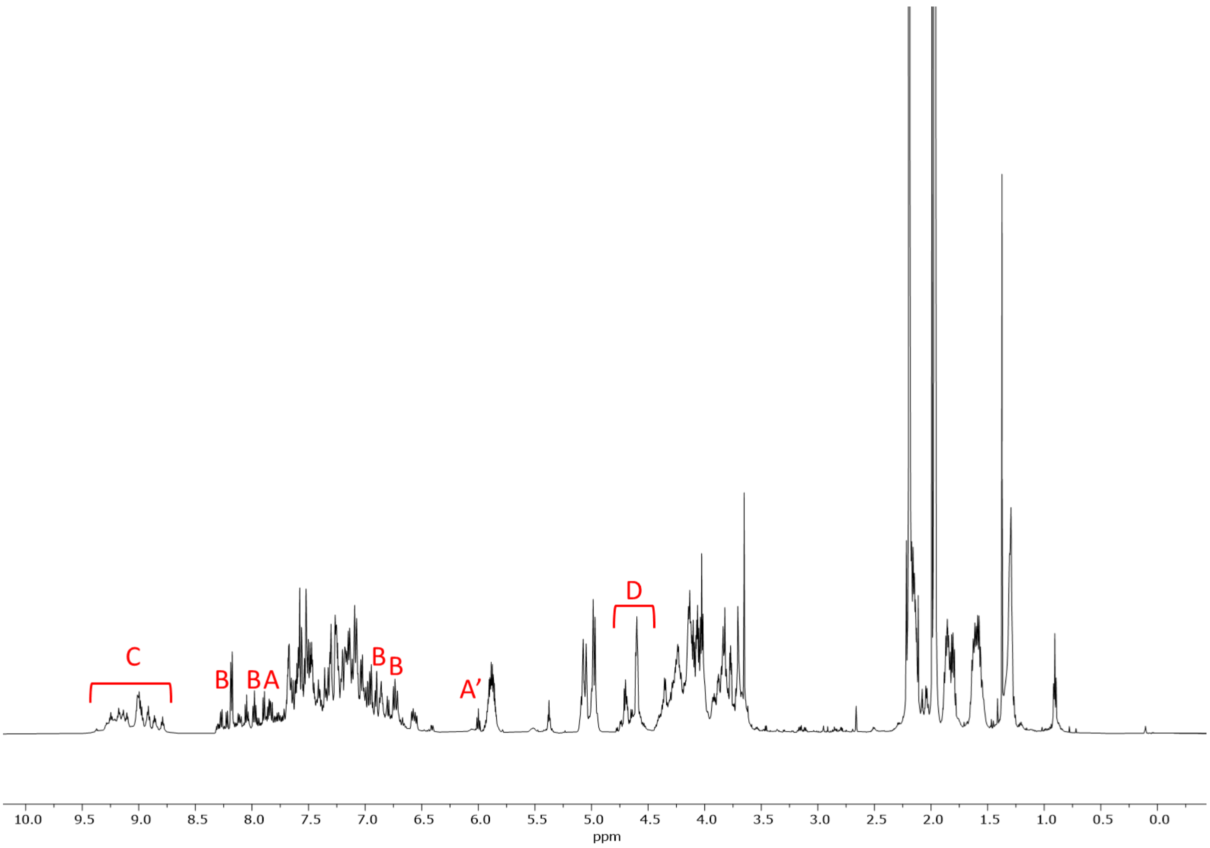

Figure S2. ^{1}H NMR (600 MHz, MeCN-d_3, 298 K) of $\mathbf{L2}_3$•$[Lu]_2$. Selected spectral assignments correspond to the above structure.

{L1,L2•[Lu]}

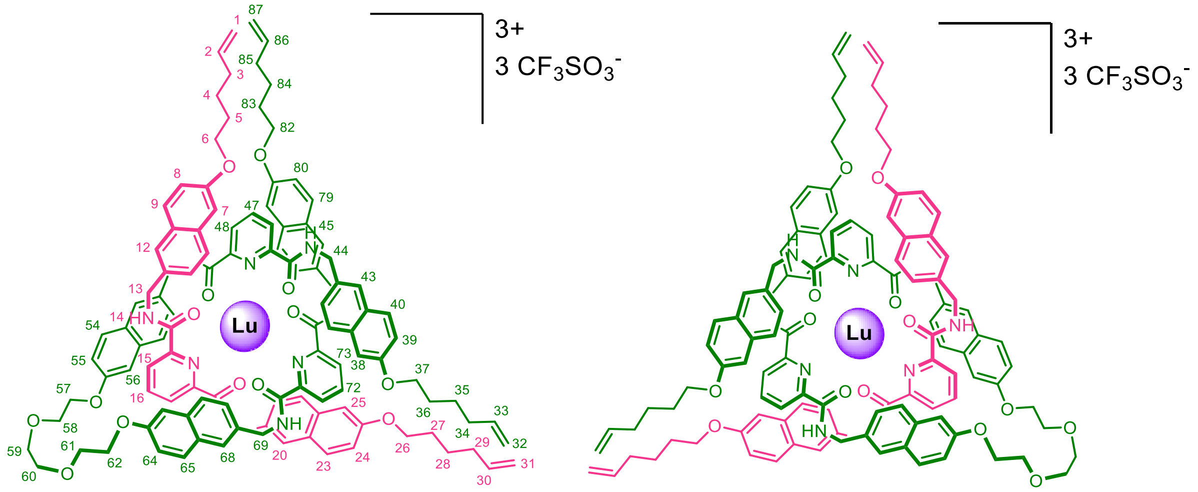

To a solution of **L1** (7.10 mg, 11.1 μmol) and **L2** (13.7 mg, 11.1 μmol) in MeCN (10 mL) was added $Lu(CF_3SO_3)_3$ (6.90 mg, 11.1 μmol) in MeCN (1 mL). The solution was stirred for 20 hours at 80 °C. The mixture was cooled to room temperature, concentrated under reduced pressure and the resulting solid was washed twice with CH_2Cl_2 to give compound {**L1**,**L2•**[Lu]} as an off-white solid (21.0 mg, 92%). **^{1}H NMR** (600 MHz, MeCN-*d*$_3$) δ 9.37 – 8.95 (m, 6H, $H_{14,18,45,49,70,74}$), 7.93 – 7.64 (m, 6H, $H_{9/10/22/23/40/41/53/54/65/66/78/79}$), 7.64 – 7.59 (m, 2H, $H_{15/17/46/48/71/73}$), 7.58 – 7.44 (m, 6H, $H_{9/10/22/23/40/41/53/54/65/66/78/79}$), 7.44 – 7.41 (m, 2H, $H_{15/17/46/48/71/73}$), 7.39 – 7.35 (m, 2H, $H_{15/17/46/48/71/73}$), 7.34 – 7.22 (m, 6H, $H_{7/8/24/25/38/39/55/56/63/64/80/81}$), 7.22 – 7.19 (m, 2H, $H_{16/47/72}$), 7.19 – 7.05 (m, 12H, $H_{7/8/12/20/24/25/38/39/43/51/55/56/63/64/68/76/80/81}$), 6.95 – 6.91 (m, 1H, $H_{16/47/72}$), 6.88 – 6.68 (m, 6H, $H_{11,21,42,52,67,77}$), 5.97 – 5.82 (m, 4H, $H_{2,30,33,86}$), 5.12 – 5.03 (m, 4H, $H_{1,31,32,87(trans)}$), 5.01 – 4.93 (m, 4H, $H_{1,31,32,87(cis)}$), 4.43 – 4.18 (m, 12H, $H_{13,19,44,50,69,75}$), 4.18 – 4.12 (m, 8H, $H_{6,26,37,82}$), 4.11 – 4.06 (m, 4H, $H_{57,62}$), 4.06 – 3.92 (m, 4H, $H_{58,61}$), 3.90 – 3.66 (m, 4H, $H_{59,60}$), 2.22 – 2.12 (m, 8H, $H_{3,29,34,85}$), 1.91 – 1.79 (m, 8H, $H_{5,27,36,83}$), 1.67 – 1.55 (m, 8H, $H_{4,28,35,84}$). **^{13}C NMR** (151 MHz, MeCN-*d*$_3$) δ 168.05, 168.01, 167.84, 167.73, 167.68, 167.30, 157.96, 157.94, 157.90, 157.88, 157.85, 157.44, 145.96, 145.77, 144.63, 142.30, 141.94, 141.66, 139.49, 139.37, 134.40, 134.33, 133.73, 132.76, 132.45, 132.35, 129.65, 129.57, 129.48, 128.85, 128.78, 128.63, 127.71, 127.61, 127.32, 126.25, 125.99, 125.82, 125.68, 125.62, 125.58, 125.28, 125.23, 124.99, 123.97, 123.51, 123.49, 120.10, 119.96, 119.74, 114.81, 114.79, 107.40, 107.29, 107.25, 107.20, 107.18, 107.00, 71.09, 70.97, 69.88, 69.75, 69.70, 69.59, 68.56, 68.38, 68.34, 68.10, 44.91, 44.80, 44.70, 33.72, 29.03, 25.71. Due to the pseudo-symmetry, several carbon signals are not resolved. **HRMS** (ESI$^+$): Calcd. for $C_{117}H_{119}O_{14}N_9Lu^{3+}$: 682.9423, found 682.9426 $[M-3(CF_3SO_3)]^{3+}$.

S4.3 Synthesis of enantiopure Λ-handed open complexes

Λ-((*R*)$_2$-L3)$_3$•[Lu]

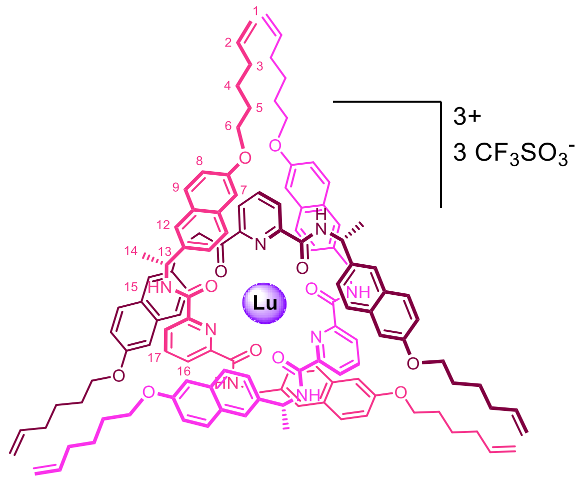

To a solution of (*R*)$_2$-**L3** (63.0 mg, 94.0 µmol) in MeCN (8.4 mL) was added Lu(CF_3SO_3)$_3$ (19.5 mg, 31.3 µmol) in MeCN (1.0 mL), and the solution was stirred at room temperature. After 24 h, the solvent was removed under reduced pressure, and the resulting off-white solid was washed twice with dichloromethane to give Λ-((*R*)$_2$-**L3**)$_3$•[Lu] (65.0 mg, 29.8 µmol) as a colorless solid in 96% yield. **^{1}H NMR** (600 MHz, MeCN-*d*$_3$) δ 8.60 (d, *J* = 6.5 Hz, 6H, H_{15}), 7.46 (d, *J* = 8.5 Hz, 6H, H_9), 7.44 (d, *J* = 9.0 Hz, 6H, H_{10}), 7.39 (d, *J* = 8.0 Hz, 6H, H_{16}), 7.23 (d, *J* = 2.5 Hz, 6H, H_{12}), 7.16 (dd, *J* = 8.9, 2.5 Hz, 6H, H_{11}), 7.13 (s, 6H, H_7), 6.79 (t, *J* = 7.9 Hz, 3H, H_{17}), 6.65 (dd, *J* = 8.5, 1.9 Hz, 6H, H_8), 6.01 – 5.93 (m, 6H, H_2), 5.14 (dq, *J* = 17.2, 1.7 Hz, 6H, H_{1trans}), 5.04 (d, *J* = 10.3 Hz, 6H, H_{1cis}), 4.76 (quint, *J* = 7.0 Hz, 6H, H_{13}), 4.25 – 4.17 (m, 12H, H_6), 2.26 (q, *J* = 7.2 Hz, 12H, H_3), 1.97 – 1.93 (m, 12H, H_5), 1.74 – 1.69 (m, 12H, H_4), 1.53 (d, *J* = 7.1 Hz, 18H, H_{14}). **^{13}C NMR** (151 MHz, MeCN-*d*$_3$) δ 166.86, 157.86, 144.85, 140.82, 139.40, 138.31, 134.19, 129.58, 128.76, 127.67, 124.51, 123.73, 123.69, 120.00, 114.88, 107.25, 68.47, 52.46, 33.79, 29.11, 25.81, 21.72. Due to the pseudo-symmetry, several carbon signals are not resolved. **HRMS** (ESI$^+$): Calcd. for $C_{129}H_{141}N_9O_{12}Lu$ [M−3(CF_3SO_3)]$^{3+}$: 727.6697, found 727.6671.

S4.4 Synthesis of trefoil knot Λ-1

Λ-{(R)$_2$-L3,(R)$_4$-L4•[Lu]}

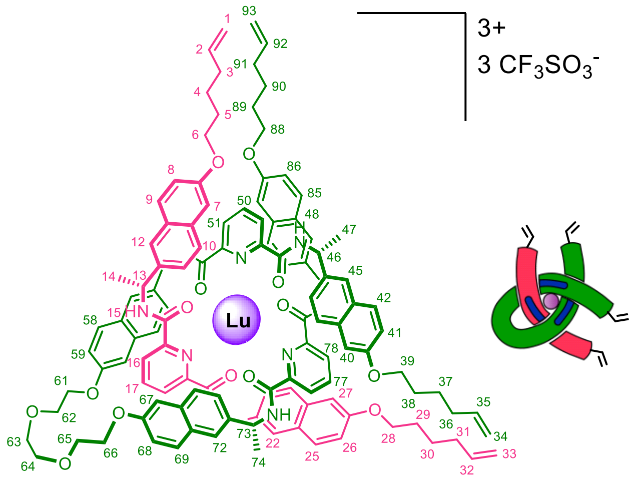

To a solution of (*R*)$_4$-**L4** (72.0 mg, 55.8 μmol) and (*R*)$_2$-**L3** (37.0 mg, 55.8 μmol) in MeCN (50 mL) was added a solution of $Lu(CF_3SO_3)_3$ (35.0 mg, 55.8 μmol) in MeCN (5 mL). The mixture was stirred for 24 hours at 80 °C. The solution was cooled to room temperature, concentrated under reduced pressure and washed twice with CH_2Cl_2 to give the product **Λ-{**(*R*)$_2$-**L3**,(*R*)$_4$-**L4**•[Lu]} (117 mg) as a colorless solid in quantitative yield. **^{1}H NMR** (600 MHz, MeCN-*d*$_3$) δ 8.77 – 8.50 (m, 6H, $H_{15,19,48,52,75,79}$), 7.67 (d, *J* = 9.0 Hz, 2H, $H_{9/10/24/25/42/43/57/58/69/70/84/85}$), 7.62 (d, *J* = 8.5 Hz, 2H, $H_{9/10/24/25/42/43/57/58/69/70/84/85}$), 7.51 (d, *J* = 8.2 Hz, 2H, $H_{16/18/49/51/76/78}$), 7.48 – 7.45 (m, 2H, $H_{9/10/24/25/42/43/57/58/69/70/84/85}$), 7.44 – 7.41 (m, 4H, $H_{16/18/49/51/76/78}$), 7.41 – 7.34 (m, 4H, $H_{9/10/24/25/42/43/57/58/69/70/84/85}$), 7.33 – 7.29 (m, 2H, $H_{9/10/24/25/42/43/57/58/69/70/84/85}$), 7.27 – 7.20 (m, 6H, $H_{7/8/26/27/40/41/59/60/67/68/86/87}$), 7.19 – 7.08 (m, 10H, $H_{7/8/12/22/26/27/40/41/45/55/59/60/67/68/72/82/86/87}$), 6.96 – 6.91 (m, 2H, $H_{12/22/45/55/72/82}$), 6.88 (t, *J* = 8.0 Hz, 2H, $H_{17/50/77}$), 6.83 – 6.79 (m, 1H, $H_{11/23/44/56/71/83}$), 6.77 (t, *J* = 7.8 Hz, 1H, $H_{17/50/77}$), 6.74 – 6.57 (m, 5H, $H_{11/23/44/56/71/83}$), 6.01 – 5.90 (m, 4H, $H_{2,32,35,92}$), 5.17 – 5.09 (m, 4H, $H_{1,33,34,93trans}$), 5.07 – 5.00 (m, 4H, $H_{1,33,34,93cis}$), 4.82 – 4.60 (m, 6H, $H_{13,20,46,53,73,80}$), 4.38 – 4.28 (m, 4H, $H_{61,66}$), 4.27 – 4.16 (m, 8H, $H_{6,28,39,88}$), 4.12 – 4.07 (m, 2H, $H_{62/65}$), 4.01 – 3.94 (m, 2H, $H_{62/65}$), 3.93 – 3.88 (m, 2H, $H_{63/64}$), 3.87 – 3.83 (m, 2H, $H_{63/64}$), 2.28 – 2.21 (m, 8H, $H_{3,31,36,91}$), 1.96 – 1.89 (m, 8H, $H_{5,29,38,89}$), 1.74 – 1.66 (m, 8H, $H_{4,30,37,90}$), 1.62 – 1.46 (m, 18H, $H_{14,21,47,54,74,81}$). **^{13}C NMR** (151 MHz, MeCN-*d*$_3$) δ 167.17, 166.94, 166.87, 166.65, 157.92, 157.85, 157.83, 157.55, 144.93, 144.79, 144.21, 140.93, 140.86, 139.38, 138.94, 138.41, 138.38, 138.33, 134.35, 134.20, 134.13, 129.69, 129.58, 129.49, 128.90, 128.77, 128.72, 127.76, 127.68, 127.62, 124.69, 124.61, 124.54, 123.93, 123.86, 123.71, 123.63, 123.06, 122.66, 120.08, 120.00, 119.97, 114.88, 107.60, 107.32, 107.31, 107.27, 71.17, 69.55, 68.80, 68.49, 68.46, 53.43, 52.64, 52.46, 52.42, 52.30, 52.19, 33.80, 33.78, 29.12, 29.11, 25.81, 25.68, 22.97, 21.76, 21.71. Due to the pseudo-symmetry, several carbon signals are not resolved. **HRMS** (ESI$^+$): Calcd. for $C_{123}H_{131}O_{14}N_9Lu^{3+}$: 710.9736, found 710.9760 $[M-3(CF_3SO_3)]^{3+}$.

Λ-1•[Lu]

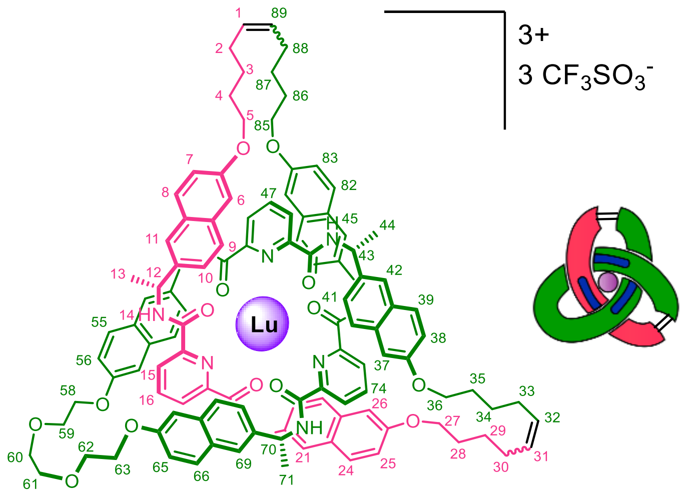

*Synthesis via olefin metathesis of (R)₂-**L3**,(R)₄-**L4**•[Lu]*

To a solution of Λ-{(R)$_2$-**L3**,(R)$_4$-**L4**•[Lu]} (33.2 mg, 15.5 µmol) in degassed CH_3NO_2 (16 mL) was added Hoveyda-Grubbs 2nd generation catalyst (10.0 mg, 15.5 µmol) in degassed CH_2Cl_2 (16 mL). The mixture was stirred for 16 hours at 50 °C and then cooled to room temperature. Ethyl vinyl ether (2 mL) was added and stirred for 1 hour at room temperature. The mixture was concentrated under reduced pressure and the solid was washed twice with CH_2Cl_2. Size exclusion chromatography (Sephadex LH-20, MeOH) yielded the pure compound Λ-**1**•[Lu] as a colorless solid (16.9 mg, 53%). ^{1}**H NMR** (600 MHz, MeCN-d_3) δ 8.59 – 8.40 (m, 6H, $H_{14,18,45,49,72,76}$), 7.56 – 7.47 (m, 12H, $H_{8,9,23,24,39,40,54,55,66,67,81,82}$), 7.27 – 7.22 (m, 6H, $H_{6/7/25/26/37/38/56/57/64/65/83/84}$), 7.22 – 7.19 (m, 6H, $H_{6/7/25/26/37/38/56/57/64/65/83/84}$), 7.08 – 7.05 (m, 4H, $H_{15/17/46/48/73/75}$), 7.05 – 7.03 (m, 4H, $H_{11/21/42/52/69/80}$), 7.03 – 6.99 (m, 2H, $H_{11/21/42/52/69/80}$), 6.98 (d, J = 8.0 Hz, 2H, $H_{15/17/46/48/73/75}$), 6.87 (d, J = 7.9 Hz, 2H, $H_{10/22/41/53/68/79}$), 6.83 (d, J = 7.5 Hz, 4H, $H_{10/22/41/53/68/79}$), 6.36 – 5.87 (m, 3H, $H_{16,47,74}$), 5.69 – 5.60 (m, 4H, $H_{1,31,32,89}$), 4.72 – 4.65 (m, 6H, $H_{12,19,43,50,70,77}$), 4.36 – 4.32 (m, 4H, $H_{58,63}$), 4.27 – 4.20 (m, 8H, $H_{5,27,36,85}$), 4.13 – 4.10 (m, 2H, $H_{59/62}$), 4.00 – 3.97 (m, 2H, $H_{59/62}$), 3.95 – 3.92 (m, 2H, $H_{60/61}$), 3.89 – 3.86 (m, 2H, $H_{60/61}$), 2.34 – 2.29 (m, 8H, $H_{2,30,33,88}$), 2.06 – 1.99 (m, 8H, $H_{4,28,35,86}$), 1.79 – 1.74 (m, 8H, $H_{3,29,34,87}$), 1.59 – 1.55 (m, 18H, $H_{13,20,44,51,71,78}$). ^{13}**C NMR** (151 MHz, MeCN-d_3) δ 167.09, 166.99, 166.88, 158.00, 157.80, 157.60, 144.13, 144.07, 144.05, 139.58, 139.12, 138.92, 138.85, 134.07, 133.98, 133.84, 131.65, 131.62, 130.77, 129.58, 128.85, 128.79, 128.72, 127.70, 127.62, 127.57, 123.83, 123.76, 123.57, 123.54, 123.46, 123.16, 122.75, 122.61, 122.57, 120.08, 120.05, 107.75, 107.43, 107.29, 71.22, 69.57, 69.02, 68.89, 68.80, 53.47, 53.27, 52.92, 32.03, 31.99, 28.20, 28.15, 26.17, 26.10, 23.03, 22.83, 22.74. Due to the pseudo-symmetry, several carbon signals are not resolved. **HRMS** (ESI^{+}): Calcd. for $C_{119}H_{123}O_{14}N_9Lu^{3+}$: 692.2860, found 692.2854 $[M-3(CF_3SO_3)]^{3+}$.

Note: A mass spectrum of Λ-**1**•[Lu] before purification is shown below (Fig. S3), highlighting the absence of alternative knot peaks corresponding to closure of either Λ-((*R*)$_2$-**L3**)$_3$•[Lu] or (Λ,Λ)-((*R*)$_4$-**L4**)$_3$•[Lu]$_2$.

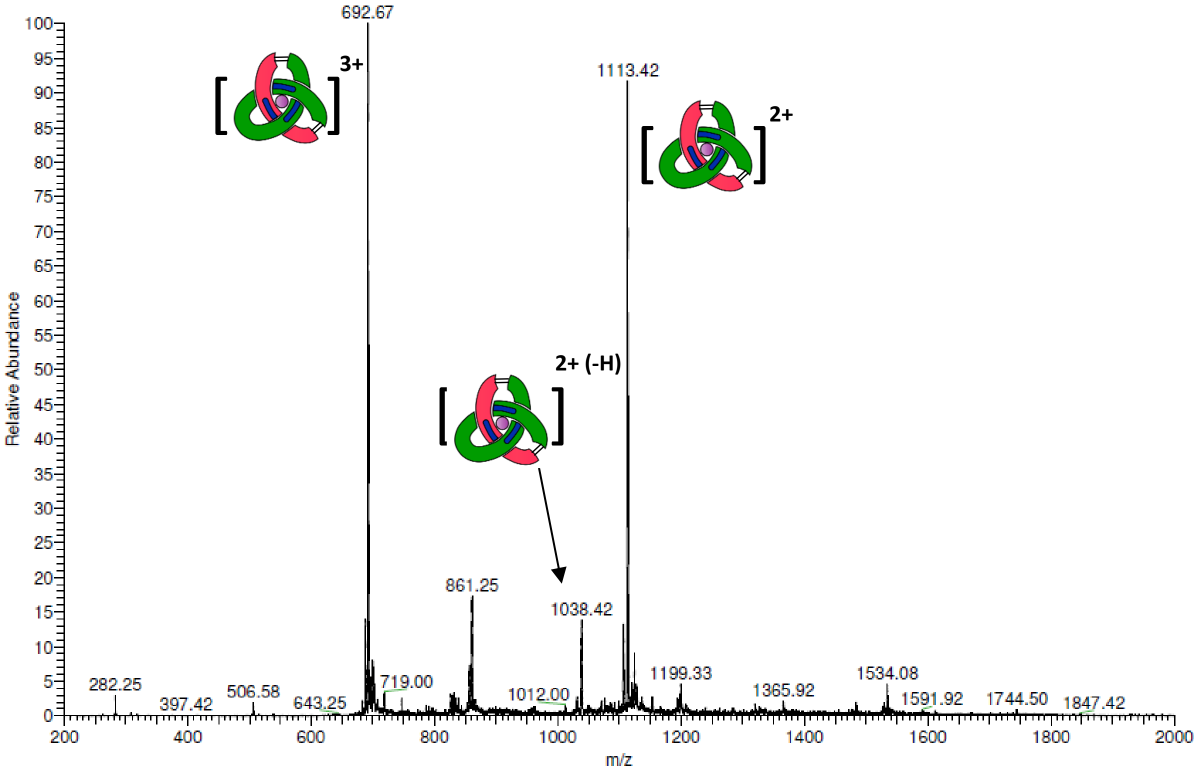

Figure S3. Low resolution ESI-MS(+) of the crude trefoil knot Λ-**1**•[Lu] (all peaks observed as [M−n(CF$_3$SO$_3$)]$^{n+}$ adducts unless otherwise specified).

Synthesis via metalation of organic trefoil knot Λ-**1**

To a solution of Λ-**1** (5.0 mg, 2.6 μmol) in MeCN (2.0 mL) was added Lu(CF$_3$SO$_3$)$_3$ (1.6 mg, 2.6 μmol) in MeCN (0.5 mL). The mixture was stirred for 24 hours at 80 °C, cooled to room temperature, concentrated under reduced pressure and washed with CH$_2$Cl$_2$ to give compound Λ-**1**•[Lu] as a colorless solid (5.1 mg, 94%). Analytical data was identical to that from the above synthesis.

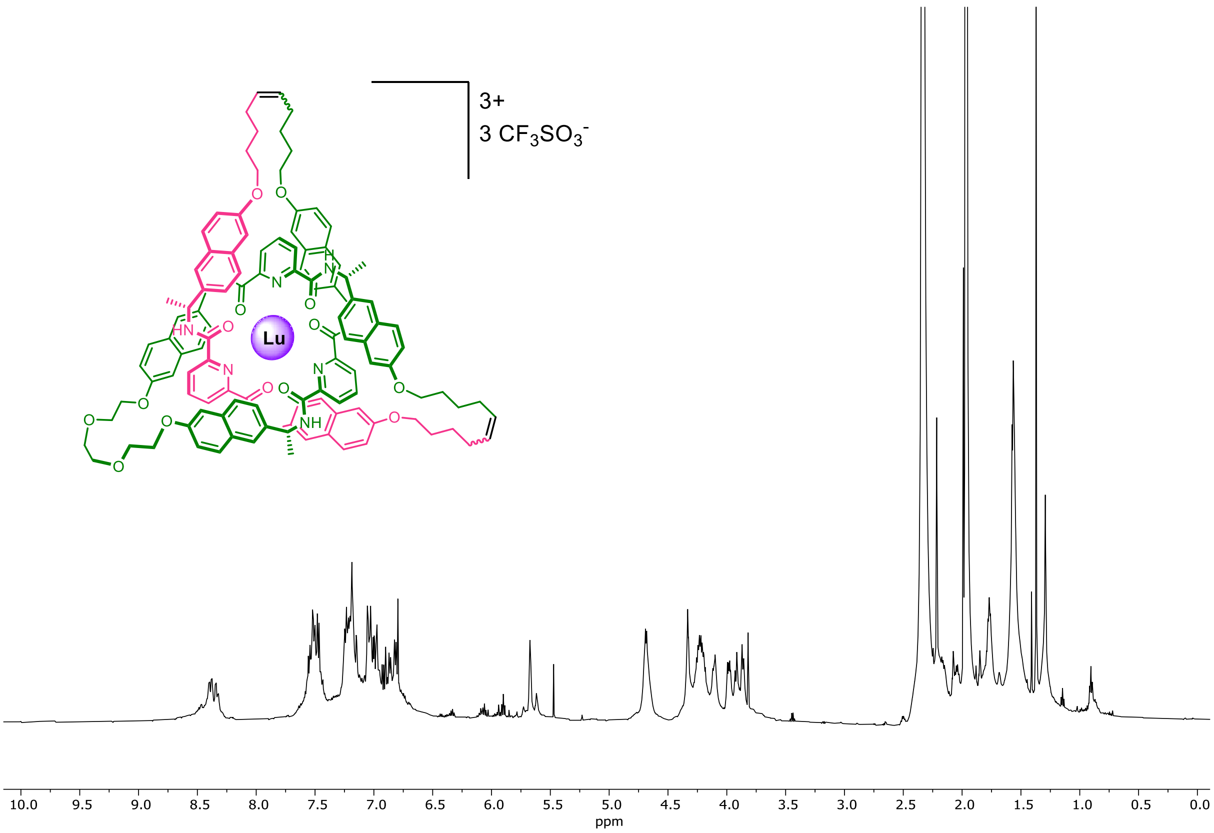

Figure S4. ^{1}H NMR spectrum (600 MHz, MeCN-d_3, 298 K) of remetalated trefoil knot Λ-**1**•[Lu].

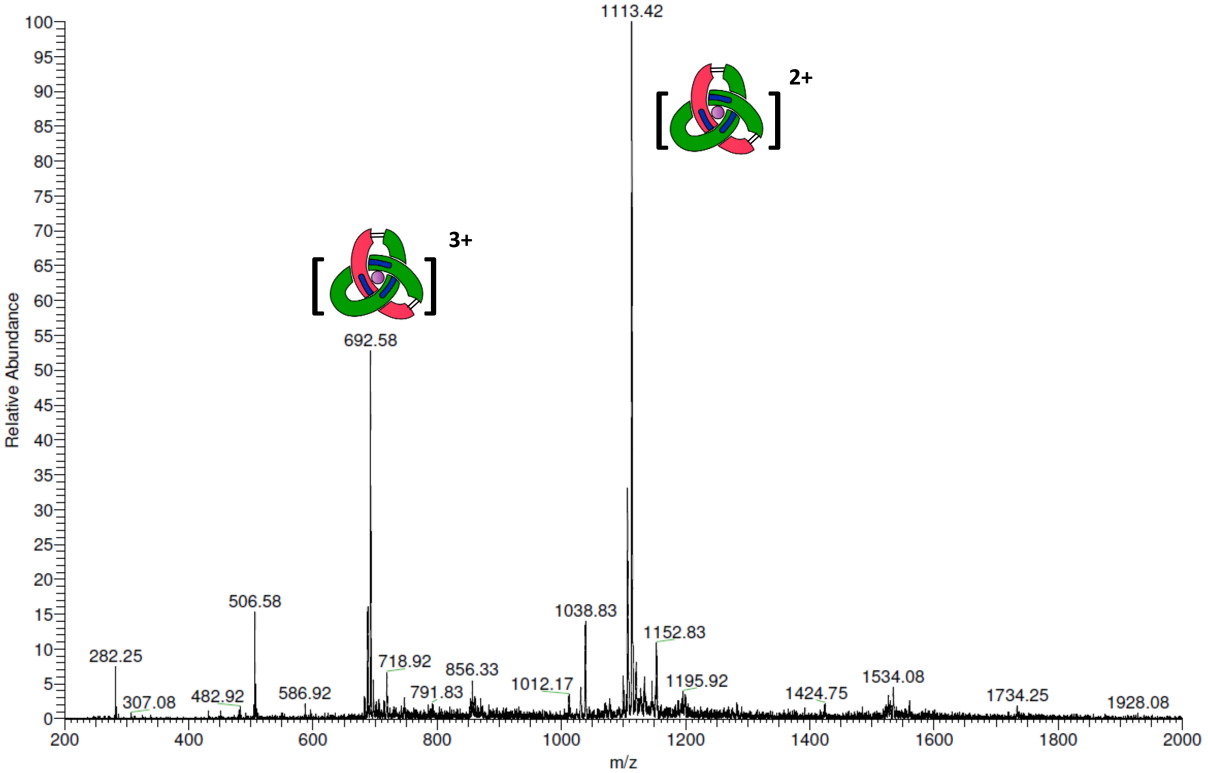

Figure S5. Low resolution ESI-MS(+) of remetalated trefoil knot Λ-**1**•[Lu] (all peaks observed as $[M{-}n(CF_3SO_3)]^{n+}$ adducts).

Λ-1

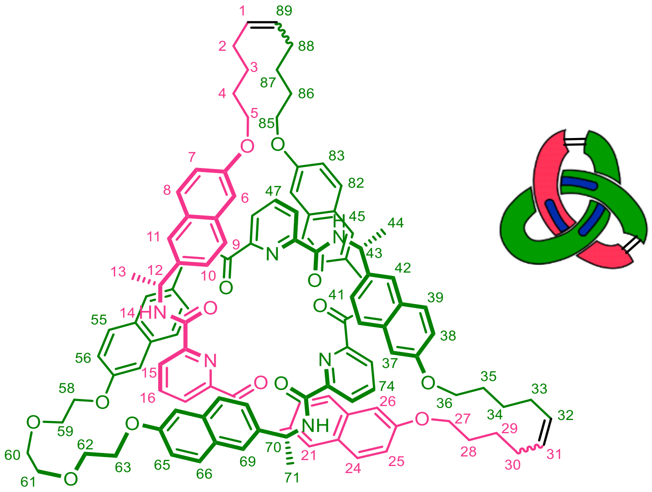

To a solution of Λ-**1**•[Lu] (79.0 mg, 37.9 μmol) in MeCN (40 mL) was added tetraethylammonium fluoride (57.0 mg, 379 μmol). The suspension was stirred for 30 minutes at room temperature and concentrated under reduced pressure. The solid was washed with MeCN (3 × 5 mL) to give the crude product as an off-white solid. The crude solid was purified by size exclusion chromatography (Bio-Beads SX-1, CH_2Cl_2) to give compound Λ-**1** as a colorless solid (29.8 mg, 35% over three steps). **^{1}H NMR** (600 MHz, $CDCl_3$) δ 8.32 – 8.22 (m, 6H, $H_{15,17,46,48,73,75}$), 8.19 – 8.04 (m, 6H, $H_{14,18,45,49,72,76}$), 7.98 – 7.80 (m, 3H, $H_{16,47,74}$), 7.65 – 7.52 (m, 18H, $H_{8,9,11,21,23,24,39,40,42,52,54,55,66,67,69,79,81,82}$), 7.40 – 7.34 (m, 6H, $H_{10,22,41,53,68,80}$), 7.16 – 7.10 (m, 6H, $H_{7,25,38,56,65,83}$), 7.07 – 7.01 (m, 6H, $H_{6,26,37,57,64,84}$), 5.51 – 5.41 (m, 4H, $H_{1,31,32,89}$), 5.40 – 5.32 (m, 6H, $H_{12,19,43,50,70,77}$), 4.24 – 4.19 (m, 4H, $H_{58,63}$), 4.10 – 3.99 (m, 8H, $H_{5,27,36,85}$), 3.94 – 3.91 (m, 4H, $H_{59,62}$), 3.80 (s, 4H, $H_{60,61}$), 2.15 – 2.02 (m, 8H, $H_{2,30,33,88}$), 1.89 – 1.79 (m, 8H, $H_{4,28,35,86}$), 1.58 – 1.54 (m, 8H, $H_{3,29,34,87}$), 1.54 – 1.43 (m, 18H, $H_{13,20,44,51,71,78}$). **^{13}C NMR** (151 MHz, $CDCl_3$) δ 162.71, 157.23, 156.91, 148.69, 138.83, 137.96, 137.74, 133.88, 133.75, 130.38, 129.20, 128.73, 128.57, 127.35, 125.06, 124.42, 119.48, 106.58, 106.36, 70.94, 69.81, 67.86, 67.44, 48.77, 32.28, 28.68, 26.00, 21.96. Due to the pseudo-symmetry, several carbon signals are not resolved. **HRMS** (ESI^+): Calcd. for $C_{119}H_{124}O_{14}N_9K^{2+}$: 970.9447, found 970.9410 $[M+H+K]^{2+}$.

Note: In the synthesis of organic knot Λ-**1**, the reaction mixture was carried through to demetalation in its crude form after closure by RCM in order to determine the composition of the crude organic mixture by MALDI-TOF mass spectrometry (see spectra below). The crude mixture was subsequently purified to obtain organic knot Λ-**1**.

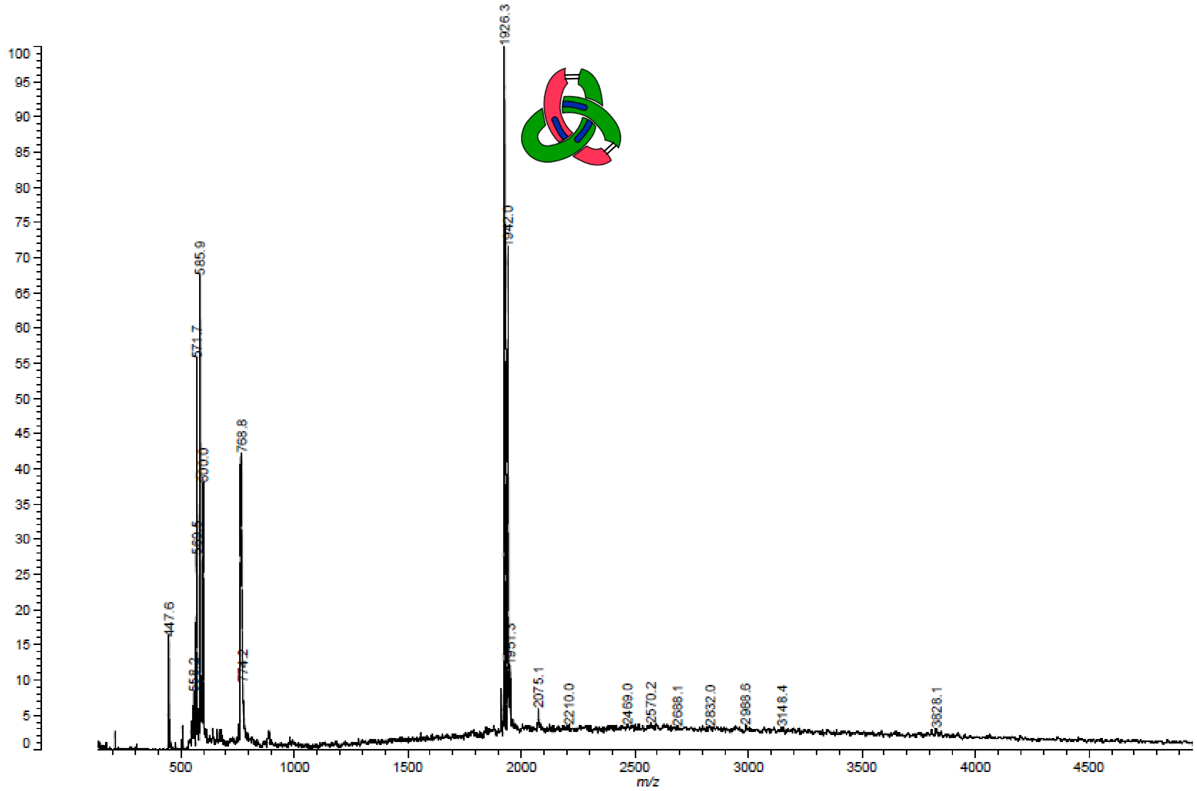

Figure S6. MALDI-TOF (MeOH, CHCA) spectrum of the crude reaction mixture after demetalation, showing organic trefoil knot Λ-**1** $(M+Na)^+$ at 1926.3 m/z. The higher mass region shows the absence of organic homomeric granny knot — the potential product derived from the narcissistic self-sorting pathway, after closure and demetalation of (Λ,Λ)-((*R*)$_4$-**L4**)$_3$•[Lu]$_2$ — at 3807 m/z $(M+Na)^+$.[4]

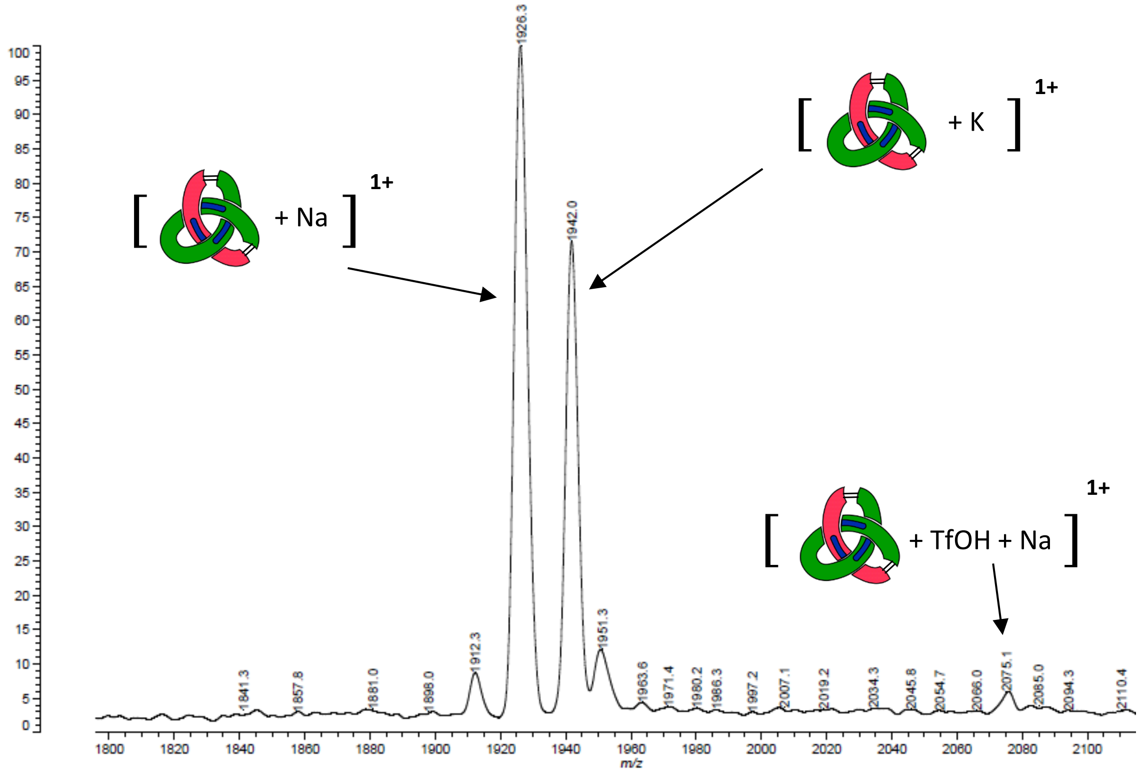

Figure S7. Zoomed-in region 1800-2100 m/z of the above spectrum, showing organic trefoil knot Λ-**1** $(M+Na)^+$ at 1926.3 m/z, $(M+K)^+$ at 1942.0 m/z and $(M+TfOH+Na)^+$ at 2075.1 m/z, and the absence of organic homomeric trefoil knot — the potential product derived from the narcissistic self-sorting pathway, after closure and demetalation of Λ-((*R*)$_2$-**L3**)$_3$•[Lu] — at 1948 m/z $(M+Na)^+$.

S4.4 Synthesis of granny knot (Λ,Λ)-2

(Λ,Λ)-2•[Lu]$_2$

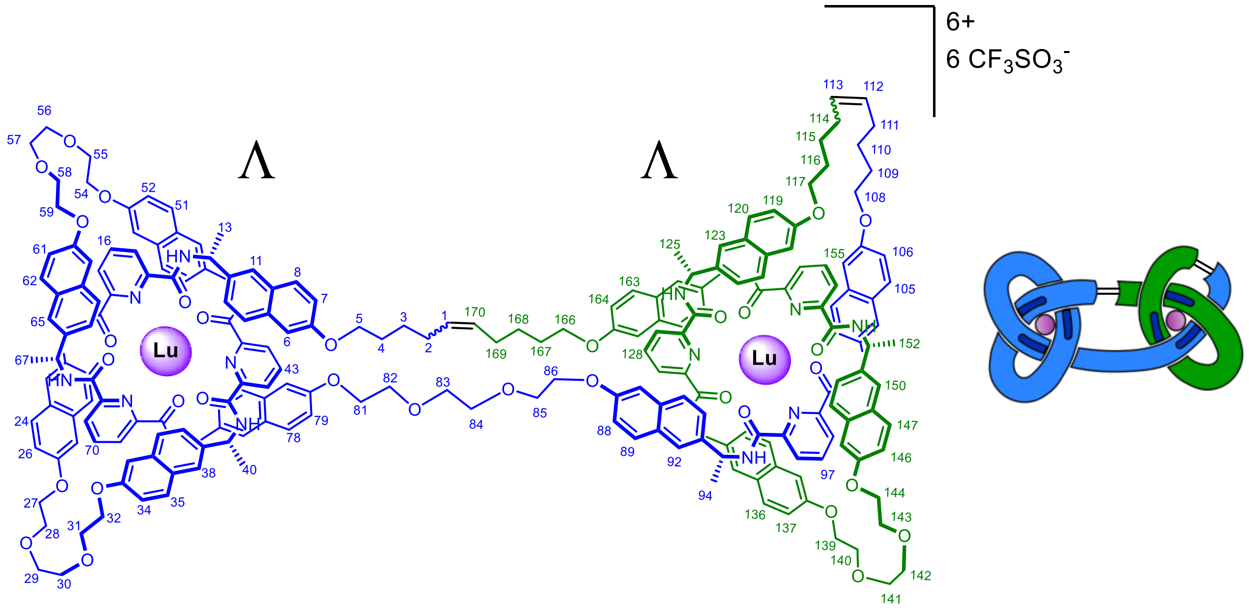

*Synthesis via complexation of ligands (R)$_4$-**L4** and (R)$_8$-**L5** with Lu(CF_3SO_3)$_3$ and subsequent olefin metathesis*

To a solution of (*R*)$_8$-**L5** (25.0 mg, 9.90 µmol) and (*R*)$_4$-**L4** (12.7 mg, 9.90 µmol) in MeCN (8.9 mL) was added a solution of Lu(CF_3SO_3)$_3$ (12.3 mg, 19.8 µmol) in MeCN (1.0 mL). The mixture was stirred for 3 days at 80 °C. The reaction mixture was cooled to room temperature and concentrated under reduced pressure to give the crude compound (Λ,Λ)-{(*R*)$_4$-**L4**,(*R*)$_8$-**L5**•[Lu]$_2$} (38.5 mg) as a white solid. This was carried through without further purification. To a solution of (Λ,Λ)-{(*R*)$_4$-**L4**,(*R*)$_8$-**L5**•[Lu]$_2$} (38.5 mg, 9.24 µmol) in degassed CH_3NO_2 (9.3 mL) was added Hoveyda-Grubbs 2nd generation catalyst (23.2 mg, 36.9 µmol) in degassed CH_2Cl_2 (9.3 mL). The mixture was stirred for 24 hours at 50 °C and cooled to room temperature. Ethyl vinyl ether (2 mL) was added and stirred for 1 hour at room temperature. The mixture was concentrated under reduced pressure and the solid was washed with CH_2Cl_2 (2 mL). Size exclusion chromatography (Sephadex LH-20, MeOH) yielded the pure compound (Λ,Λ)-**2**•[Lu]$_2$ as a colorless solid (10.6 mg, 26%). **^{1}H NMR** (600 MHz, MeCN-*d*$_3$) δ 8.58 – 8.31 (m, 12H, $H_{14,18,41,45,68,72,95,99,126,130,153,157}$), 7.75 – 7.41 (m, 32H, $H_{8,9,22,23,24,35,36,37,49,50,51,62,63,64,77,78,89,90,103,104,105,120,121,122,134,135,136,147,148,149,162,163}$), 7.39 – 7.13 (m, 24H, $H_{6,7,25,26,33,34,52,53,60,61,79,80,87,88,106,107,118,119,137,138,145,146,164,165}$), 7.11 – 6.69 (m, 30H, $H_{10,11,14,17,21,38,41,43,44,48,65,68,71,75,76,91,92,95,98,102,123,126,128,129,133,150,154,156,160,161}$), 6.07 – 5.85 (m, 4H, $H_{16,70,97,155}$), 5.81 – 5.54 (m, 4H, $H_{1,112,113,170}$), 4.77 – 4.57 (m, 12H, $H_{12,19,39,46,66,73,93,100,124,131,151,158}$), 4.42 – 3.71 (m, 56H, $H_{5,27,28,29,30,31,32,54,55,56,57,58,59,61,62,63,64,65,86,108,118,139,140,141,142,143,144,166}$), 2.18 – 2.05 (m, 16H, $H_{2,4,109,111,114,116,167,169}$), 2.01 – 1.98 (m, 8H, $H_{3,110,115,168}$), 1.74 – 1.50 (m, 36H,

$H_{13,20,40,47,67,74,94,103,125,132,152,159}$). **^{13}C NMR** (151 MHz, MeCN-d_3) δ 167.18, 167.08, 167.02, 167.00, 166.92, 166.85, 158.10, 157.69, 157.59, 157.56, 157.53, 157.50, 144.39, 144.32, 144.08, 144.03, 144.01, 143.96, 139.32, 139.30, 139.09, 139.06, 139.03, 133.99, 133.95, 133.82, 133.78, 133.73, 131.58, 131.56, 131.14, 129.86, 129.84, 129.56, 129.53, 129.04, 128.93, 128.90, 128.82, 128.79, 127.76, 127.68, 127.65, 127.61, 127.50, 127.46, 126.40, 125.95, 123.82, 123.51, 123.37, 123.19, 122.97, 122.82, 122.64, 120.20, 120.04, 120.01, 119.99, 119.95, 119.71, 119.67, 119.56, 119.48, 107.80, 107.78, 107.76, 107.71, 107.40, 107.31, 107.23, 107.12, 71.43, 71.37, 71.26, 71.18, 71.16, 71.14, 70.38, 70.29, 70.25, 69.62, 69.57, 69.56, 69.55, 69.52, 69.05, 68.98, 68.89, 68.85, 68.81, 68.52, 68.50, 68.48, 68.42, 68.39, 68.38, 68.34, 68.32, 53.71, 53.69, 53.59, 53.59, 53.39, 53.35, 53.21, 53.05, 32.53, 32.06, 31.97, 31.94, 31.58, 30.46, 30.20, 29.91, 29.74, 29.27, 28.26, 28.16, 26.88, 26.27, 26.25, 26.09, 23.14, 23.11, 23.07, 23.02, 22.99, 22.88, 22.77, 22.74, 22.69, 22.68, 20.44. Due to the pseudo-symmetry, several carbon signals are not resolved. **HRMS** (ESI^+): Calcd. for $C_{231}H_{234}O_{35}N_{18}F_3SLu_2^{5+}$: 851.7109, found 851.7129 $[M-5(CF_3SO_3)]^{5+}$.

Note: A mass spectrum of (Λ,Λ)-**2**•$[Lu]_2$ before purification is shown below, highlighting the absence of alternative knot peaks corresponding to closure of either (Λ,Λ)-((*R*)$_4$-**L4**)$_3$•$[Lu]_2$ or ($Λ_3$,Λ)-((*R*)$_8$-**L5**)$_3$•$[Lu]_4$.

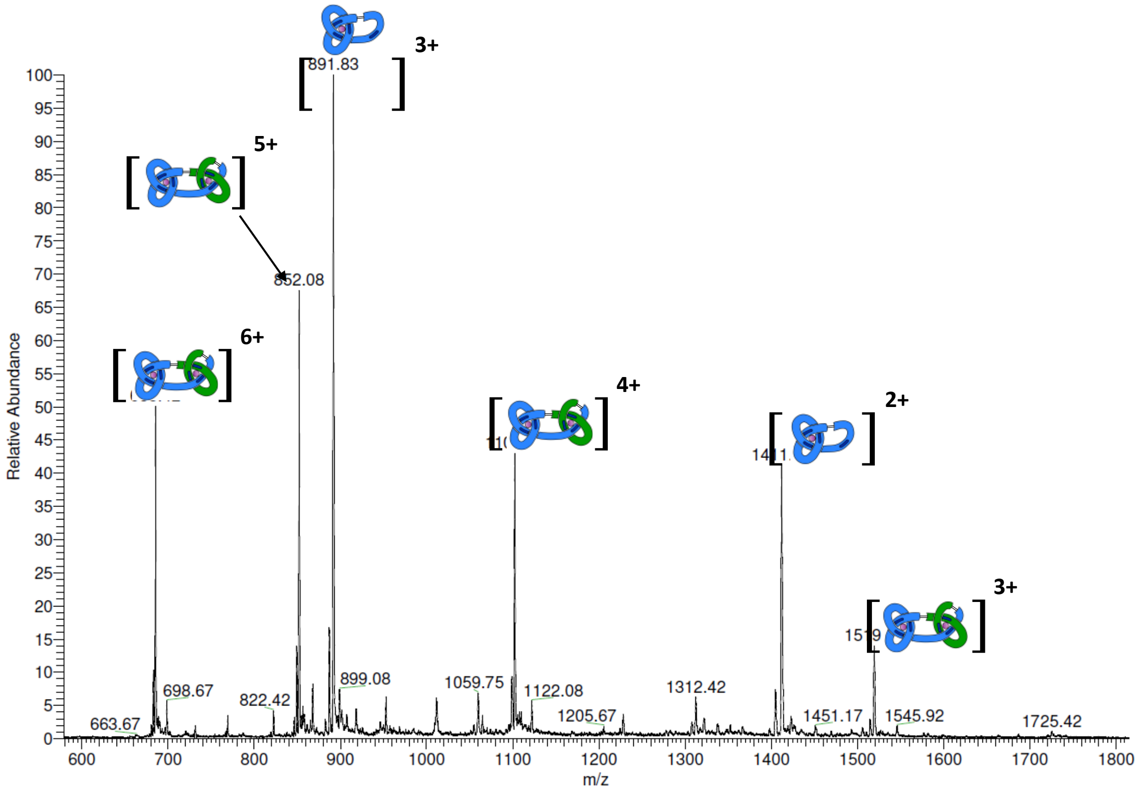

Figure S8. Low resolution ESI-MS(+) of the crude granny knot (Λ,Λ)-**2**•$[Lu]_2$ and side product derived from intramolecular closure of Λ-(*R*)$_8$-**L5**•[Lu] (all peaks observed as $[M-n(CF_3SO_3)]^{n+}$ adducts).

*Synthesis via metalation of organic granny knot (Λ,Λ)-**2***

To a solution of (Λ,Λ)-**2** (2.6 mg, 0.69 µmol) in MeCN (0.4 mL) was added Lu(CF_3SO_3)$_3$ (0.9 mg, 1.38 µmol) in MeCN (0.1 mL). The mixture was stirred for 24 hours at 80 °C, cooled to room temperature, concentrated under reduced pressure and washed with CH_2Cl_2 to give compound (Λ,Λ)-**2**•[Lu]$_2$ as a colorless solid (1.9 mg, 68%). Analytical data was identical to that of the above synthesis.

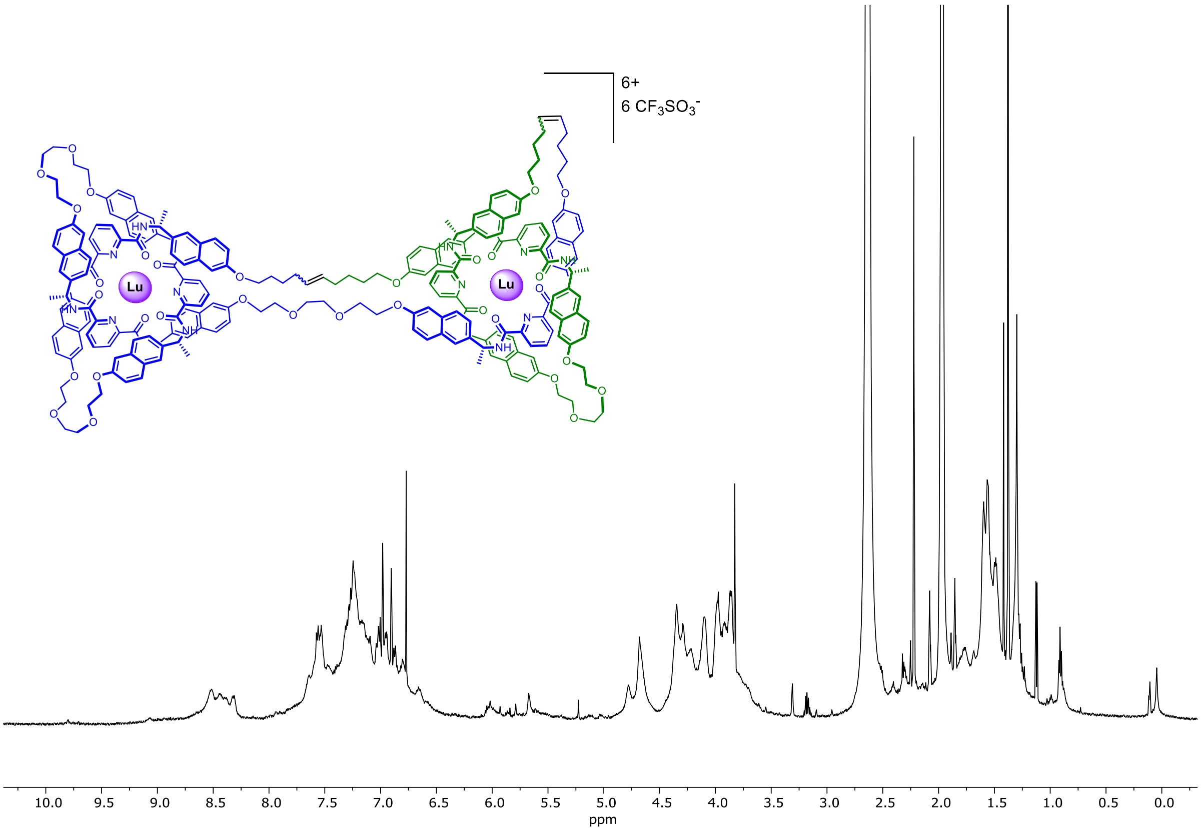

Figure S9. ^{1}H NMR spectrum (600 MHz, MeCN-d_3, 298 K) of remetalated granny knot (Λ,Λ)-**2**•[Lu]$_2$.

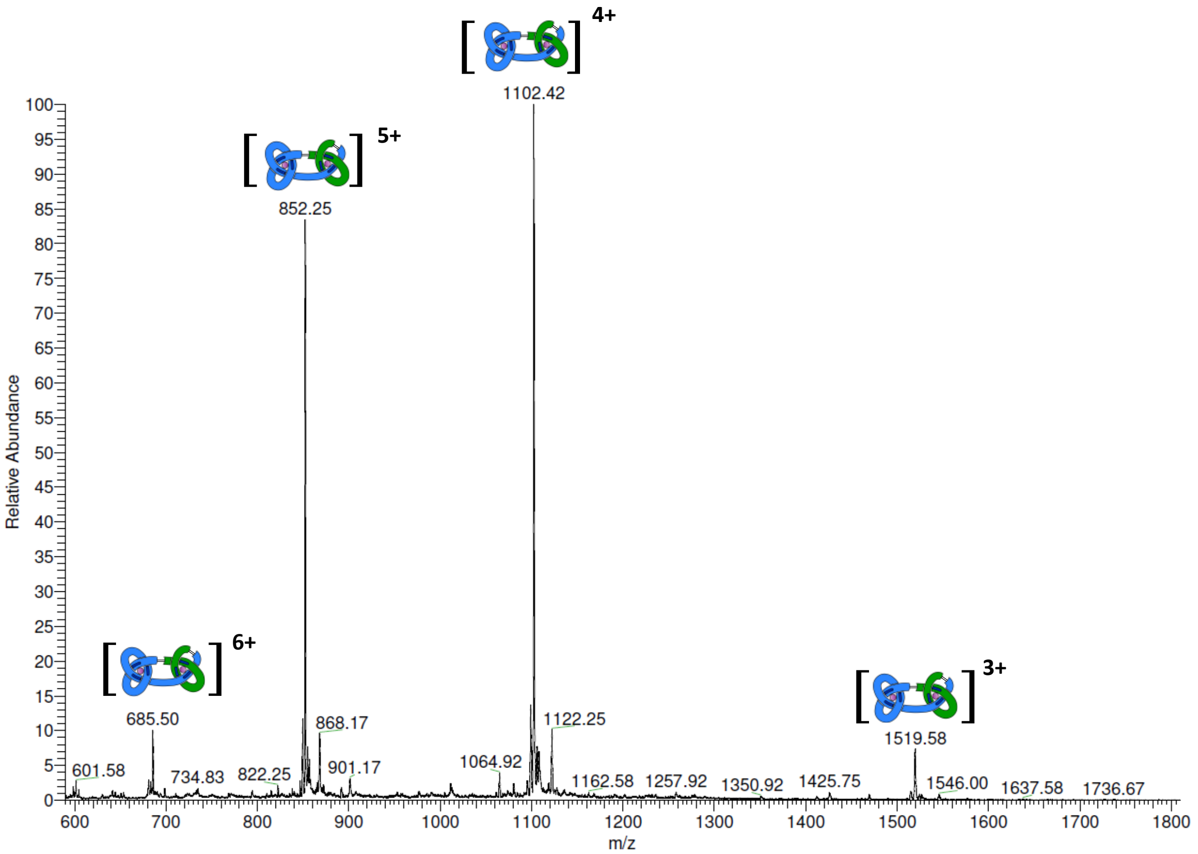

Figure S10. Low resolution ESI-MS(+) of remetalated granny knot (Λ,Λ)-**2**•$[Lu]_2$ (all peaks observed as $[M{-}n(CF_3SO_3)]^{n+}$ adducts).

(Λ,Λ)-2

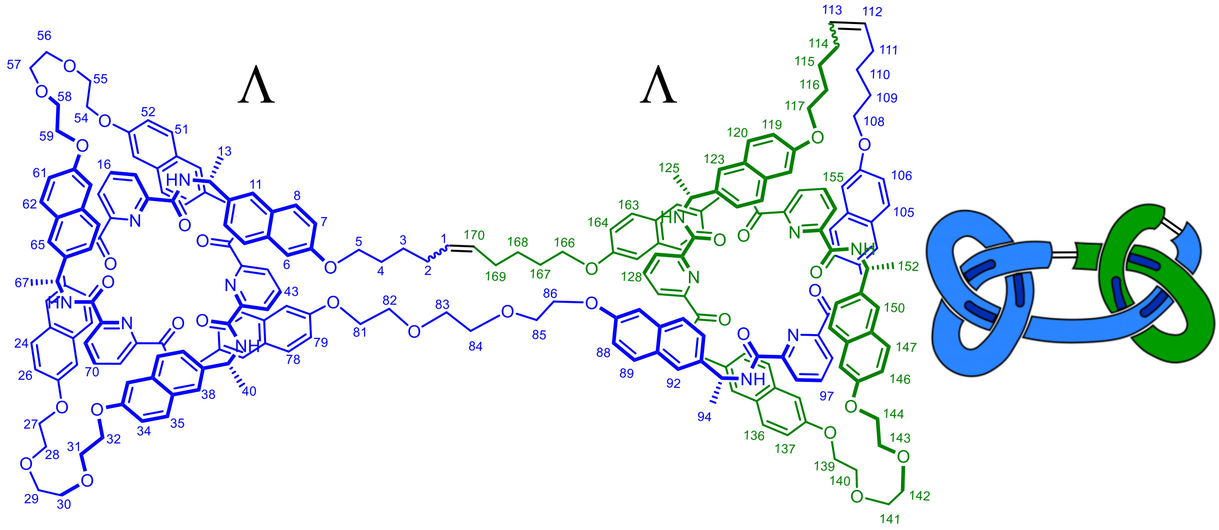

To a solution of (Λ,Λ)-**2**•[Lu]$_2$ (21.4 mg, 5.22 μmol) in MeCN (5 mL) was added tetraethylammonium fluoride (7.80 mg, 52.2 μmol). The suspension was stirred for 30 minutes at room temperature and then filtered under reduced pressure. The solid was washed with MeCN (3 × 2 mL) to give the crude product (Λ,Λ)-**2** as an off-white solid. The crude solid was purified by size exclusion chromatography (Bio-Beads SX-1, CH_2Cl_2) to give compound (Λ,Λ)-**2** as a colorless solid (3.49 mg, 13% over three steps). **^{1}H NMR** (600 MHz, $CDCl_3$) δ 8.33 – 8.20 (m, 12H, $H_{15,17,42,44,69,71,96,98,127,129,154,156}$), 8.20 – 7.99 (m, 12H, $H_{14,18,41,45,68,72,95,99,126,130,153,157}$), 7.98 – 7.79 (m, 6H, $H_{16,43,70,97,128,155}$), 7.68 – 7.51 (m, 36H, $H_{8,9,11,21,23,24,35,36,37,48,50,51,62,63,65,75,77,78,89,90,92,102,104,105,120,121,123,133,135,136,147,148,150,160,162,163}$), 7.40 – 7.33 (m, 12H, $H_{10,22,37,46,64,76,91,103,122,134,149,161}$), 7.17 – 7.10 (m, 12H, $H_{7,25,34,52,61,79,88,106,119,137,146,164}$), 7.09 – 7.02 (m, 12H, $H_{6,26,33,53,60,80,87,107,118,138,145,165}$), 5.52 – 5.42 (m, 4H, $H_{1,112,113,170}$), 5.41 – 5.33 (m, 12H, $H_{12,19,39,46,66,73,93,100,124,131,151,158}$), 4.26 – 4.19 (m, 16H, $H_{27,32,54,59,81,86,139,144}$), 4.09 – 4.01 (m, 8H, $H_{5,108,117,166}$), 3.95 – 3.90 (m, 16H, $H_{28,31,55,58,82,85,140,143}$), 3.79 (s, 16H, $H_{29,30,56,57,83,84,141,142}$), 2.13 – 2.04 (m, 8H, $H_{2,111,114,169}$), 1.90 – 1.81 (m, 8H, $H_{4,109,116,167}$), 1.64 – 1.59 (m, 8H, $H_{3,110,115,168}$), 1.55 – 1.42 (m, 36H, $H_{13,20,40,47,67,74,94,103,125,132,152,159}$). **^{13}C NMR** (151 MHz, $CDCl_3$) δ 161.69, 155.90, 147.66, 137.83, 137.77, 136.94, 132.84, 132.72, 129.34, 128.21, 127.70, 126.37, 124.03, 123.38, 118.43, 105.58, 105.35, 69.91, 68.77, 66.84, 66.43, 47.71, 31.23, 27.65, 24.96, 20.45, 20.32. Due to the pseudo-symmetry, several carbon signals are not resolved. **MALDI-TOF**: Calcd. for $C_{230}H_{234}O_{32}N_{18}Na^+$: 3785.4, found 3785.2 $[M+Na]^+$.

Note: In the synthesis of organic knot (Λ,Λ)-**2**, the reaction mixture was carried through to demetalation in its crude form after closure by RCM in order to determine the composition of the crude organic mixture by MALDI-TOF mass spectrometry (see spectra below). The crude mixture was subsequently purified to obtain organic knot (Λ,Λ)-**2**.

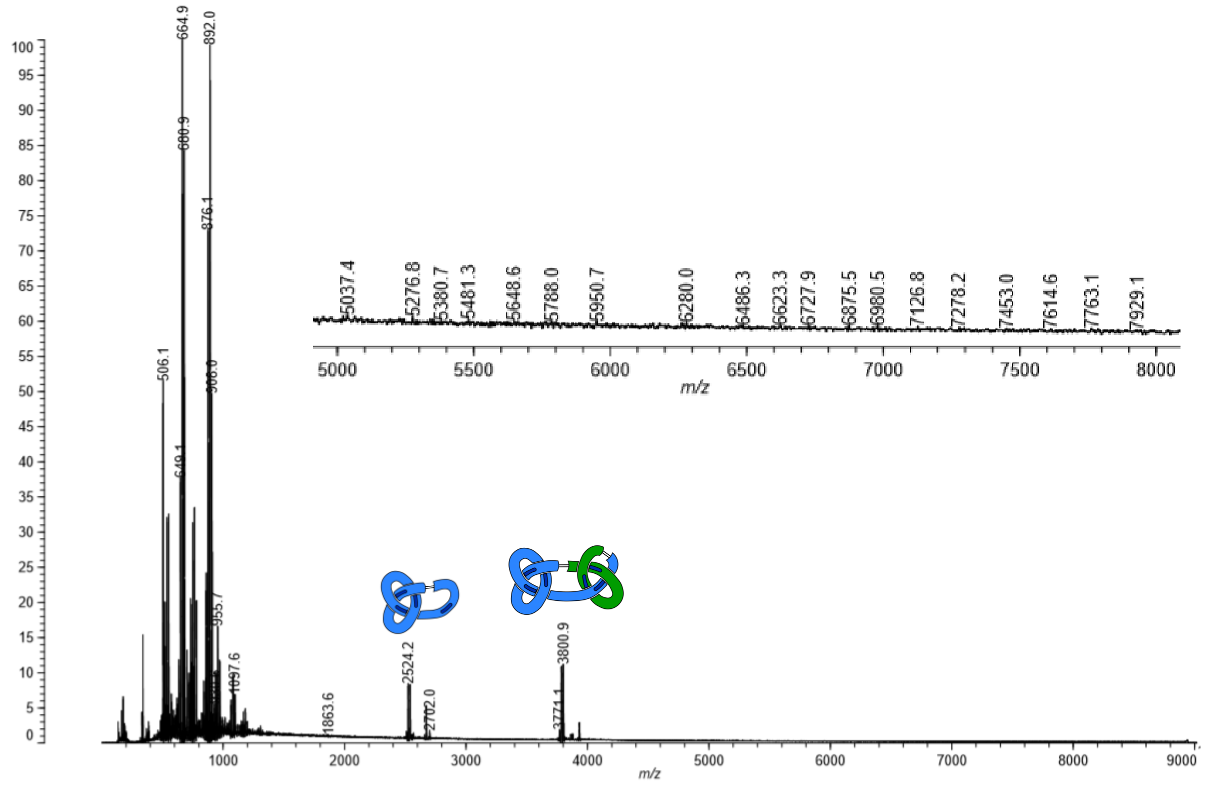

Figure S11. MALDI-TOF (MeOH, CHCA) spectrum of the crude reaction mixture after demetalation, showing organic granny knot (Λ,Λ)-**2** $(M+K)^+$ at 3800.9 m/z, and an organic side product, derived from intramolecular closure of Λ-(R)$_8$-**L5**•[Lu] followed by demetalation (M+Na), at 2524.2 m/z. The higher mass region shows the absence of organic homomeric triskelion knot — the potential product derived from the narcissistic self-sorting pathway, after closure and demetalation of (Λ_3,Λ)-((R)$_8$-**L5**)$_3$•[Lu]$_4$ — at 7535 m/z $(M+Na)^+$.[4]

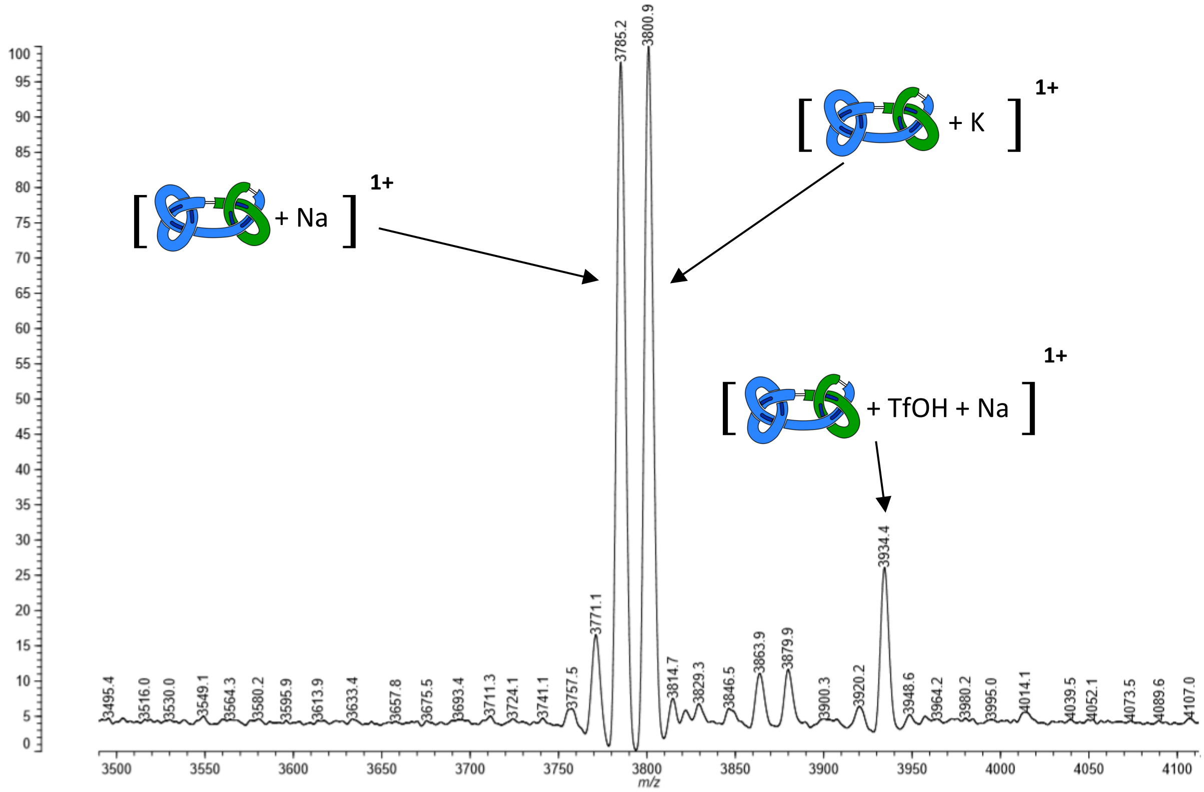

Figure S12. Zoomed-in region 3500-4100 m/z of the above spectrum, showing organic heteromeric granny knot (Λ,Λ)-**2** (M+Na)$^+$ at 3785.2 m/z, (M+K)$^+$ at 3800.9 m/z and (M+TfOH+Na)$^+$ at 3934.4 m/z, and the absence of organic homomeric granny knot — the potential product derived from the narcissistic self-sorting pathway, after closure and demetalation of (Λ,Λ)-((*R*)$_4$-**L4**)$_3$•[Lu]$_2$ — at 3807 m/z (M+Na)$^+$.[4]

S4.5 Synthesis of square knot (Λ,Δ)-2

(Λ,Δ)-2•[Lu]$_2$

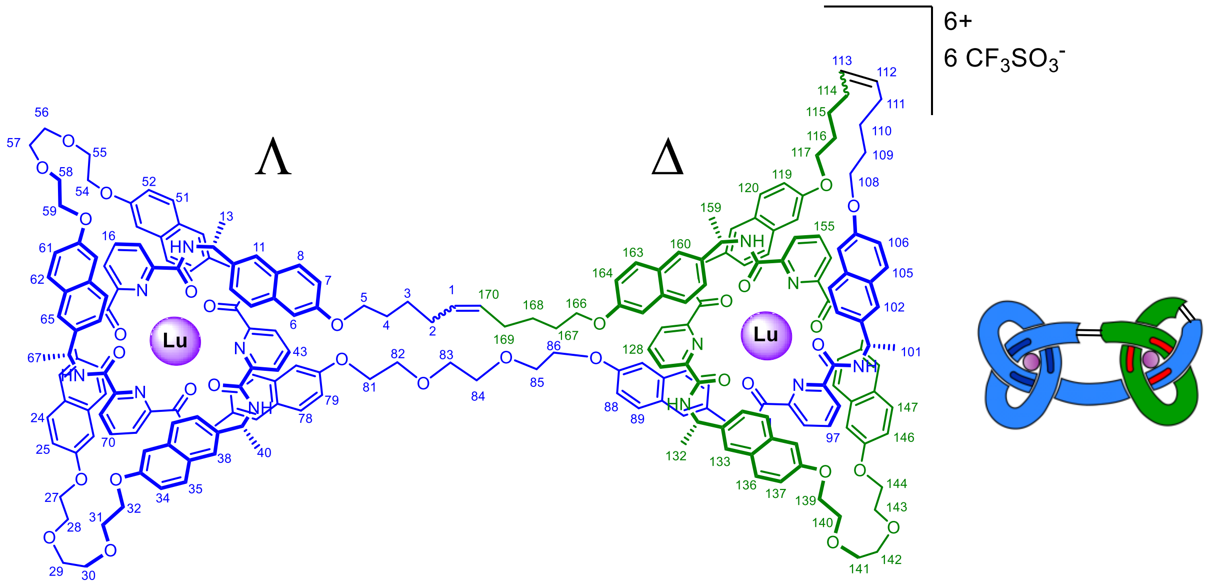

*Synthesis via complexation of ligands (S)$_4$-**L4** and (S)$_2$(R)$_6$-**L5** with Lu(CF_3SO_3)$_3$ and subsequent olefin metathesis*

To a solution of (*S*)$_2$(*R*)$_6$-**L5** (40.0 mg, 15.8 µmol) and (*S*)$_4$-**L4** (20.4 mg, 15.8 µmol) in MeCN (15 mL) was added a solution of Lu(CF_3SO_3)$_3$ (19.7 mg, 31.7 µmol) in MeCN (1 mL). The mixture was stirred for 3 days at 80 °C. The reaction mixture was cooled to room temperature and concentrated under reduced pressure to give the crude compound (Λ,Δ)-**{**(*S*)$_4$-**L4**,(*S*)$_2$(*R*)$_6$-**L5**•[Lu]$_2$} (65.8 mg) as a pale yellow solid. This was carried through without further purification. To a solution of (Λ,Δ)-**{**(*S*)$_4$-**L4**,(*S*)$_2$(*R*)$_6$-**L5**•[Lu]$_2$} (65.8 mg, 15.8 µmol) in degassed CH_3NO_2 (7.5 mL) was added Hoveyda-Grubbs 2nd generation catalyst (39.6 mg, 63.2 µmol) in degassed CH_2Cl_2 (7.5 mL). The mixture was stirred for 24 hours at 50 °C and then cooled to room temperature. Ethyl vinyl ether (2 mL) was added and stirred for 1 hour at room temperature. The mixture was concentrated under reduced pressure and the solid was washed with CH_2Cl_2 (2 mL). Size exclusion chromatography (Sephadex LH-20, MeOH) yielded the pure compound (Λ,Δ)-**2**•[Lu]$_2$ as a colorless solid (18.8 mg, 29%). **^{1}H NMR** (600 MHz, MeCN-*d*$_3$) δ 8.60 – 8.27 (m, 12H, $H_{14,18,41,45,68,72,95,99,126,130,153,157,}$), 7.63 – 7.35 (m, 32H, $H_{8,9,22,23,24,35,36,37,49,50,51,62,63,64,77,78,89,90,103,104,105,120,121,122,134,135,136,147,148,149,162,163}$), 7.32 – 7.08 (m, 24H, $H_{6,7,25,26,33,34,52,53,60,61,79,80,87,88,106,107,118,119,137,138,145,146,164,165}$), 7.06 – 6.70 (m, 30H, $H_{10,11,15,17,21,38,42,43,44,48,65,69,70,75,76,91,92,96,98,102,123,127,129,131,133,150,154,156,160,161}$), 6.09 – 5.86 (m, 4H, $H_{16,70,97,155}$), 5.74 – 5.36 (m, 4H, $H_{1,112,113,170}$), 4.80 – 4.59 (m, 12H, $H_{12,19,39,46,66,73,93,100,124,131,151,158}$), 4.43 – 3.75 (m, 56H, $H_{5,27,28,29,30,31,32,54,55,56,57,58,59,81,82,83,84,85,86,108,117,139,140,141,142,143,144,166}$), 2.33 – 2.01 (m, 16H, $H_{2,4,109,111,114,116,167,169}$), 1.92 – 1.84 (m, 8H, $H_{3,110,115,168}$), 1.62 – 1.48 (m, 36H,

$H_{13,20,40,47,67,74,94,103,125,132,152,159}$). **^{13}C NMR** (151 MHz, MeCN-*d_3*) δ 167.23, 167.20, 167.03, 166.97, 166.88, 166.82, 166.79, 157.83, 157.78, 157.68, 157.65, 157.62, 157.59, 157.57, 157.54, 157.50, 144.34, 144.29, 144.27, 144.23, 144.19, 144.15, 144.06, 139.63, 139.54, 139.48, 139.45, 139.09, 139.08, 139.04, 139.01, 138.99, 138.95, 138.94, 138.92, 138.32, 134.13, 133.95, 133.93, 133.74, 131.63, 131.18, 130.46, 129.71, 129.65, 129.60, 129.50, 129.05, 128.95, 128.92, 128.81, 128.78, 128.77, 127.78, 127.75, 127.72, 127.65, 127.64, 127.62, 127.59, 127.57, 127.54, 126.42, 125.96, 123.79, 123.67, 123.65, 123.62, 123.56, 123.54, 123.50, 123.42, 123.22, 123.21, 123.14, 123.12, 123.05, 123.04, 123.00, 122.92, 122.90, 122.85, 122.67, 122.64, 122.62, 122.59, 107.80, 107.76, 107.69, 107.62, 107.52, 107.46, 107.41, 107.29, 71.32, 71.27, 71.21, 71.10, 71.04, 71.01, 70.05, 69.94, 69.85, 69.60, 69.04, 68.95, 68.87, 68.83, 68.54, 68.33, 53.78, 53.67, 53.39, 53.26, 53.03, 52.85, 52.83, 52.60, 52.56, 52.49, 32.80, 32.06, 31.98, 31.58, 30.20, 30.04, 29.92, 29.74, 29.25, 28.26, 28.15, 27.36, 26.61, 26.19, 26.09, 25.90, 23.13, 23.11, 22.89, 22.85, 22.40, 22.27, 20.45. Due to the high degree of symmetry within the molecule several ^{13}C peaks overlap. **HRMS** (ESI$^-$): Calcd. for $C_{231}H_{234}N_{18}O_{35}F_3SLu_2^{5+}$: 851.7109, found 851.7128 [M-5(CF_3SO_3)]$^{5+}$.

Note: A mass spectrum of (Λ,Δ)-**2**•[Lu]$_2$ before purification is shown below, highlighting the absence of alternative knot peaks corresponding to closure of either (Δ,Δ)-((*S*)$_4$-**L4**)$_3$•[Lu]$_2$ or (Λ$_3$,Δ)-((*S*)$_2$(*R*)$_6$-**L5**)$_3$•[Lu]$_4$.

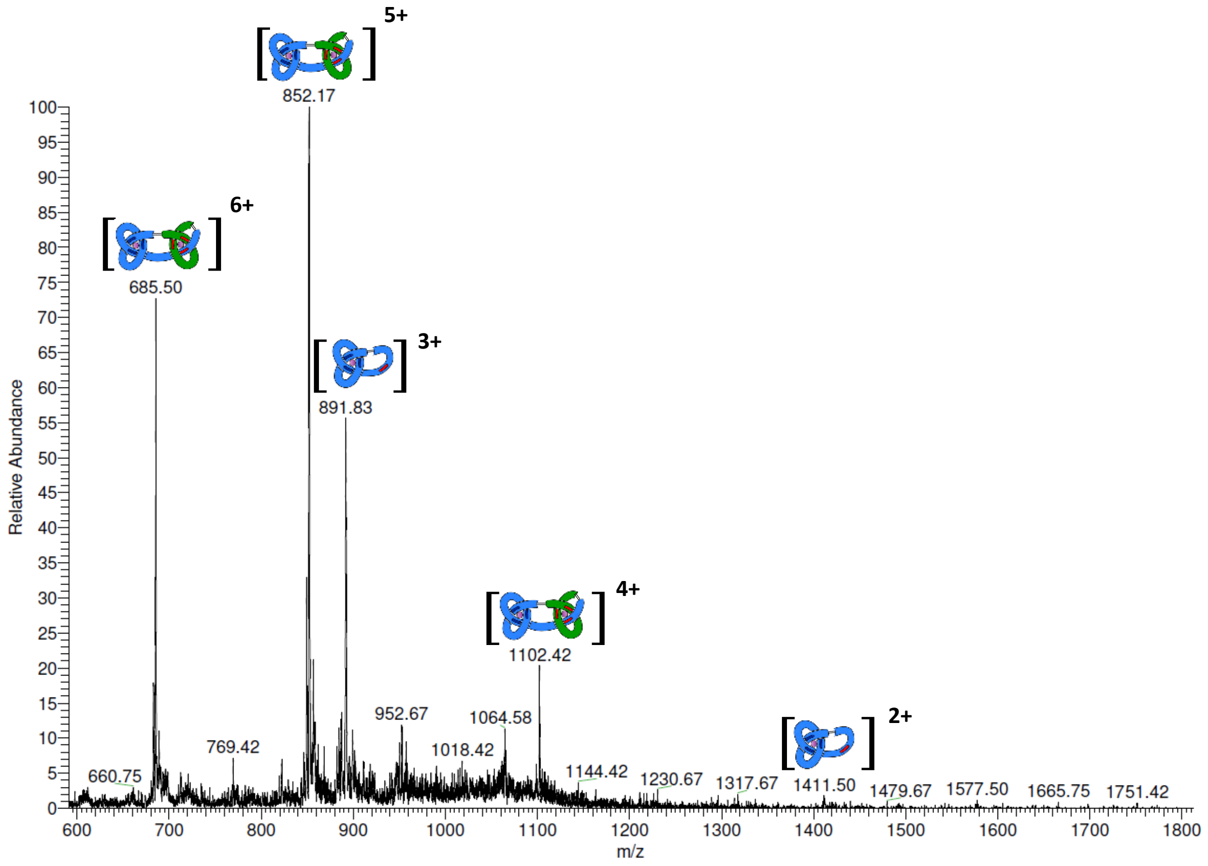

Figure S13. Low resolution ESI-MS(+) of the crude square knot (Λ,Δ)-**2**•[Lu]$_2$ and side product derived from intramolecular closure of Λ-(*S*)$_2$(*R*)$_6$-**L5**•[Lu] (all peaks observed as [M−n(CF_3SO_3)]$^{n+}$ adducts).

*Synthesis via metalation of organic square knot (Λ,Δ)-**2***

To a solution of (Λ,Δ)-**2** (1.1 mg, 0.29 µmol) in MeCN (0.4 mL) was added Lu(CF_3SO_3)$_3$ (0.4 mg, 0.59 µmol) in MeCN (0.1 mL). The mixture was stirred for 40 hours at 80 °C, cooled to room temperature, concentrated under reduced pressure and washed with CH_2Cl_2, giving (Λ,Δ)-**2**•[Lu]$_2$ as a colorless solid (1.2 mg, 75%). Analytical data was identical to that of the above synthesis.

(Λ,Δ)-2

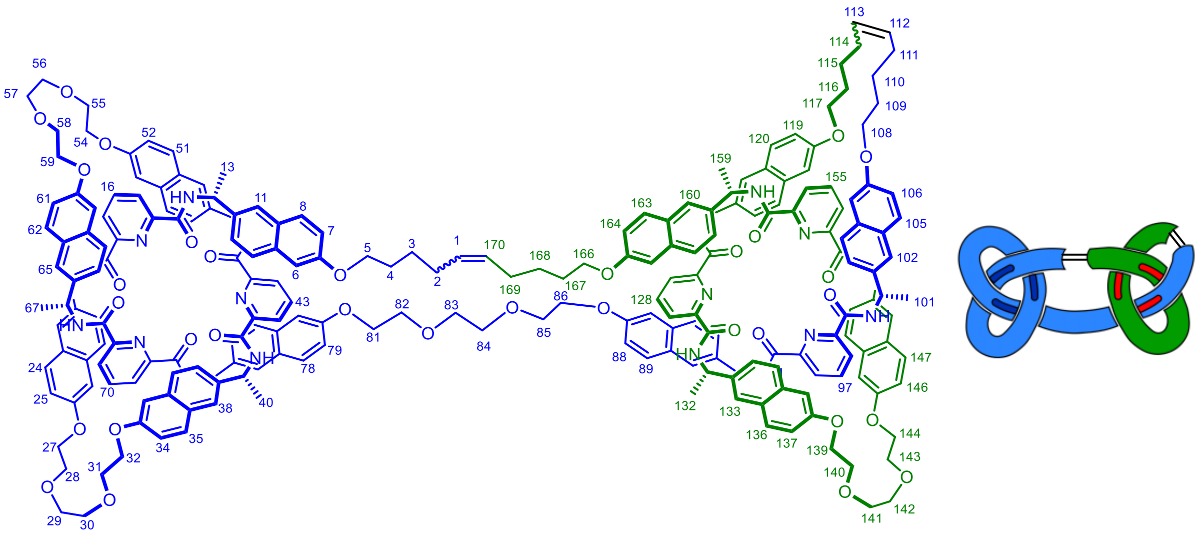

To a solution of (Λ,Δ)-**2**•[Lu]$_2$ (41.6 mg, 10.1 µmol) in MeCN (9.7 mL) was added tetraethylammonium fluoride (15.1 mg, 101 µmol). The suspension was stirred for 30 minutes at room temperature and then filtered under reduced pressure. The solid was washed with MeCN (3 × 2 mL) to give the crude product (Λ,Δ)-**2** as an off-white solid. The crude solid was purified by size exclusion chromatography (Bio-Beads SX-1, CH_2Cl_2) to give (Λ,Δ)-**2** as a colorless solid (2.2 mg, 7%). Analytical data was identical to that of granny knot (Λ,Λ)-**2**, except the CD spectrum which showed a near-zero response, as expected for a topologically achiral square knot. **MALDI-TOF**: Calcd. for $C_{230}H_{234}O_{32}N_{18}Na^+$: 3785.4, found 3783.6 [M+Na]$^+$.

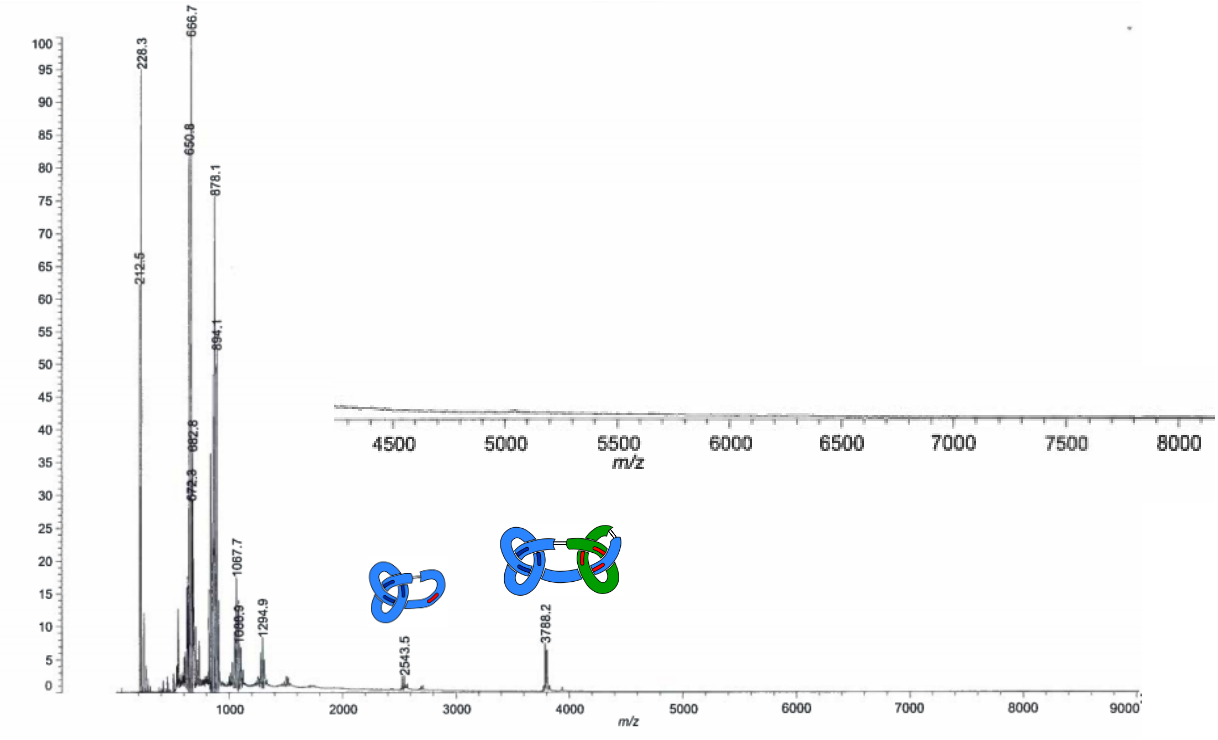

Figure S14. MALDI-TOF (MeOH, CHCA) spectrum of the crude reaction mixture after demetalation, showing organic square knot (Λ,Δ)-**2** $(M+Na)^+$ at 3788.2 m/z, and an organic side product, derived from intramolecular closure of Λ-(S)$_2$(R)$_6$-**L5**•[Lu] followed by demetalation (M+K), at 2543.5 m/z. The higher mass region shows the absence of organic homomeric triskelion knot — the potential product derived from the narcissistic self-sorting pathway, after closure and demetalation of ($Λ_3$,Δ)-((S)$_2$(R)$_6$-**L5**)$_3$•[Lu]$_4$ — at 7535 m/z $(M+Na)^+$.[4]

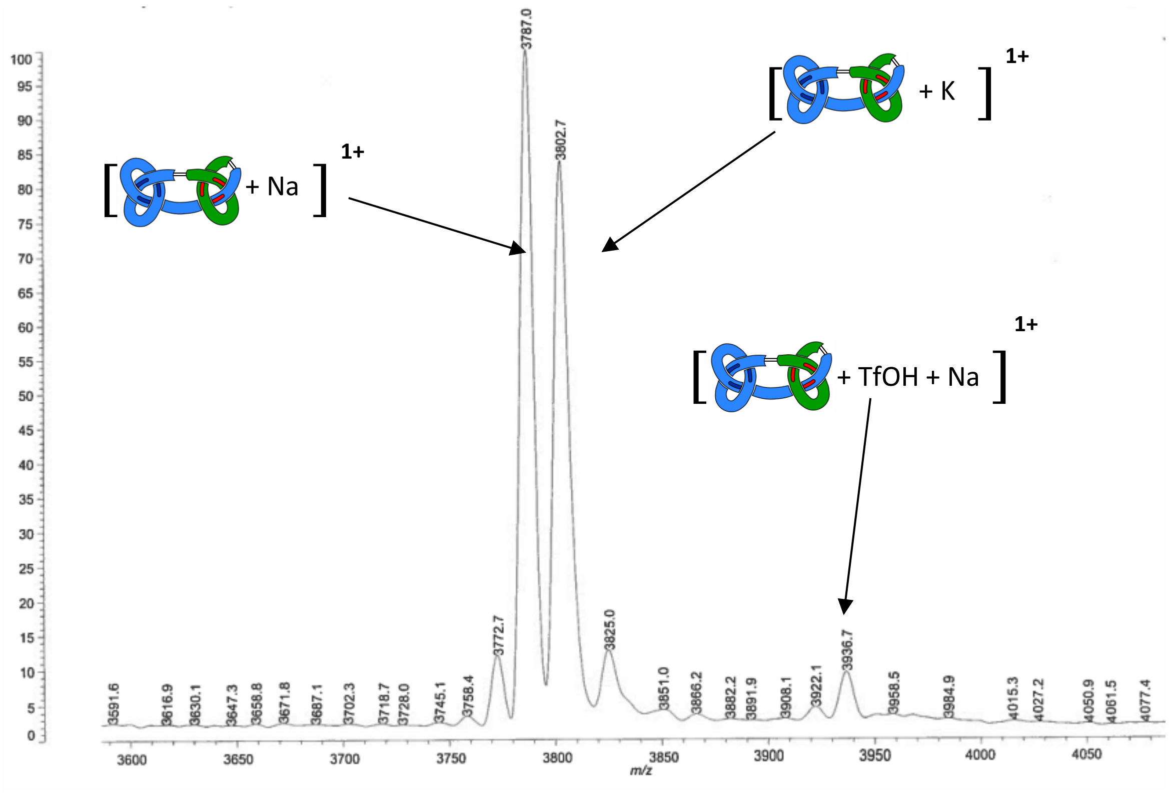

Figure S15. Zoomed-in region 3500-4100 m/z of the above spectrum, showing organic square knot (Λ,Λ)-**2** $(M+Na)^+$ at 3787.0 m/z, $(M+K)^+$ at 3802.7 m/z and $(M+TfOH+Na)^+$ at 3936.7 m/z, and the absence of organic homomeric granny knot — the potential product derived from the narcissistic self-sorting pathway, after closure and demetalation of (Δ,Δ)-$((S)_4$-**L4**$)_3$•$[Lu]_2$ — at 3807 m/z $(M+Na)^+$.[4]

S4.6 In-situ rearrangement of helicate Λ-((*R*)$_2$-L3)$_3$•[Lu] and granny complex (Λ,Λ)-((*R*)$_4$-L4)$_3$•[Lu]$_2$ to form helicate Λ-{(*R*)$_2$-L3,(*R*)$_4$-L4•[Lu]}

Λ-((*R*)$_2$-L3)$_3$•[Lu]

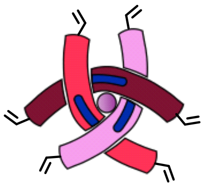

To a solution of (*R*)$_2$-**L3** (0.43 mg, 0.63 µmol) in MeCN-*d*$_3$ (0.4 mL) was added $Lu(CF_3SO_3)_3$ (0.13 mg, 0.21 µmol) in MeCN-*d*$_3$ (0.1 mL). The solution was heated at 80 °C and complexation was monitored by ^{1}H NMR spectroscopy and ESI-MS. After 24 hours, the crude solution of helicate Λ-((*R*)$_2$-**L3**)$_3$•[Lu] (0.46 mg, 0.21 µmol) was cooled to room temperature and used in the subsequent rearrangement experiment without any change to reaction concentration.

(Λ,Λ)-((*R*)$_4$-L4)$_3$•[Lu]$_2$

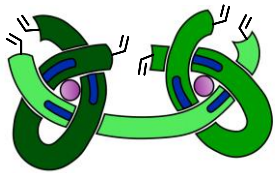

To a solution of (*R*)$_4$-**L4** (0.83 mg, 0.64 µmol) in MeCN-*d*$_3$ (0.4 mL) was added $Lu(CF_3SO_3)_3$ (0.26 mg, 0.43 µmol) in MeCN-*d*$_3$ (0.1 mL). The solution was heated at 80 °C and complexation was monitored by ^{1}H NMR spectroscopy and ESI-MS. After 3 days, the crude solution of open granny complex (Λ,Λ)-((*R*)$_4$-**L4**)$_3$•[Lu]$_2$ (0.90 mg, 0.21 µmol) was cooled to room temperature and used in the subsequent rearrangement experiments without any change to reaction concentration.[4]

Rearrangement experiment

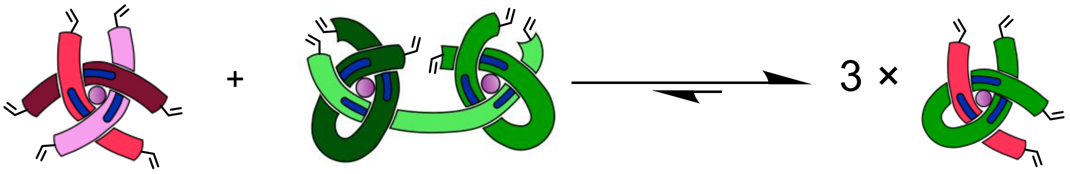

A 0.42 mM solution of (Λ,Λ)-((*R*)$_2$-**L3**)$_3$•[Lu] (0.2 mL) was added to a 0.42 mM solution of Λ-((*R*)$_4$-**L4**)$_3$•[Lu]$_2$ (0.2 mL). The mixture was left unstirred for 10 minutes at room temperature and then stirred for 4 hours at 80 °C. The rearrangement reaction was monitored by ^{1}H NMR spectroscopy and ESI-MS until no further change was observed.

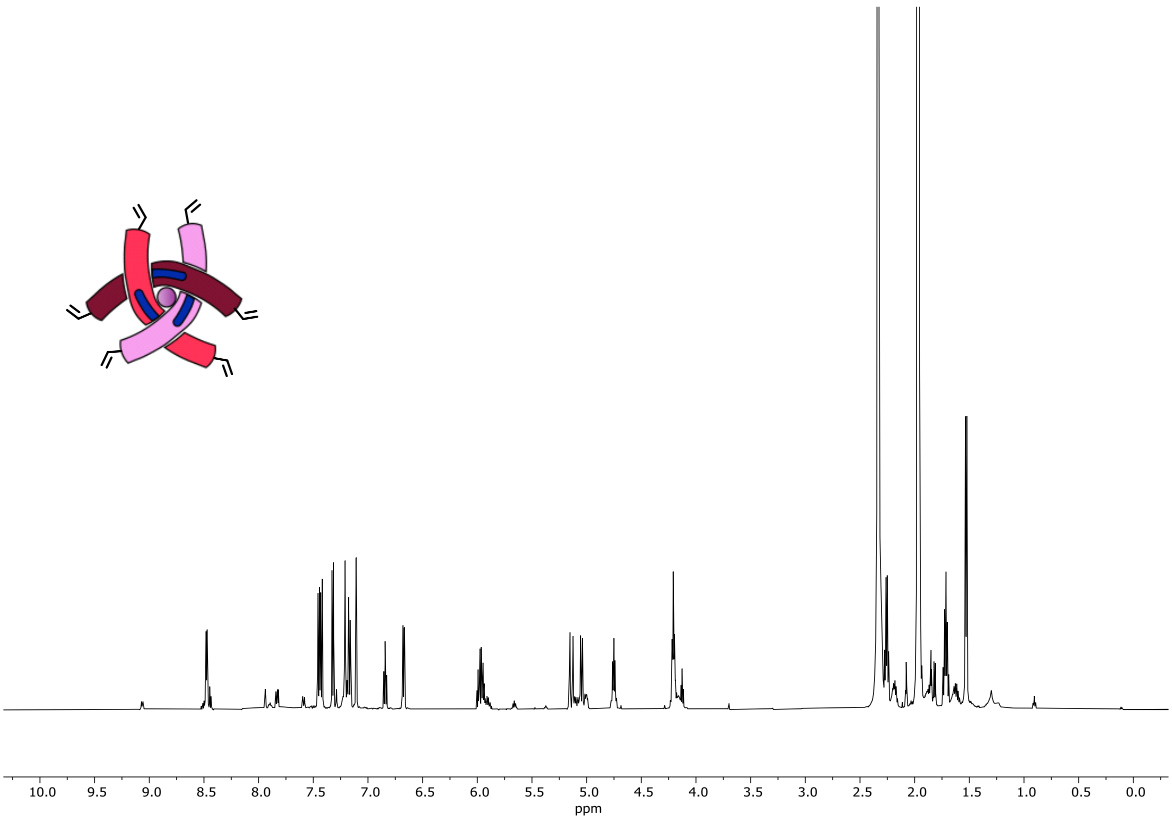

Figure S16. ^{1}H NMR spectrum (600 MHz, MeCN-d_3, 298 K) of crude reaction mixture of helicate Λ-((*R*)$_2$-**L3**)$_3$•[Lu] after 24 h at 80 °C.

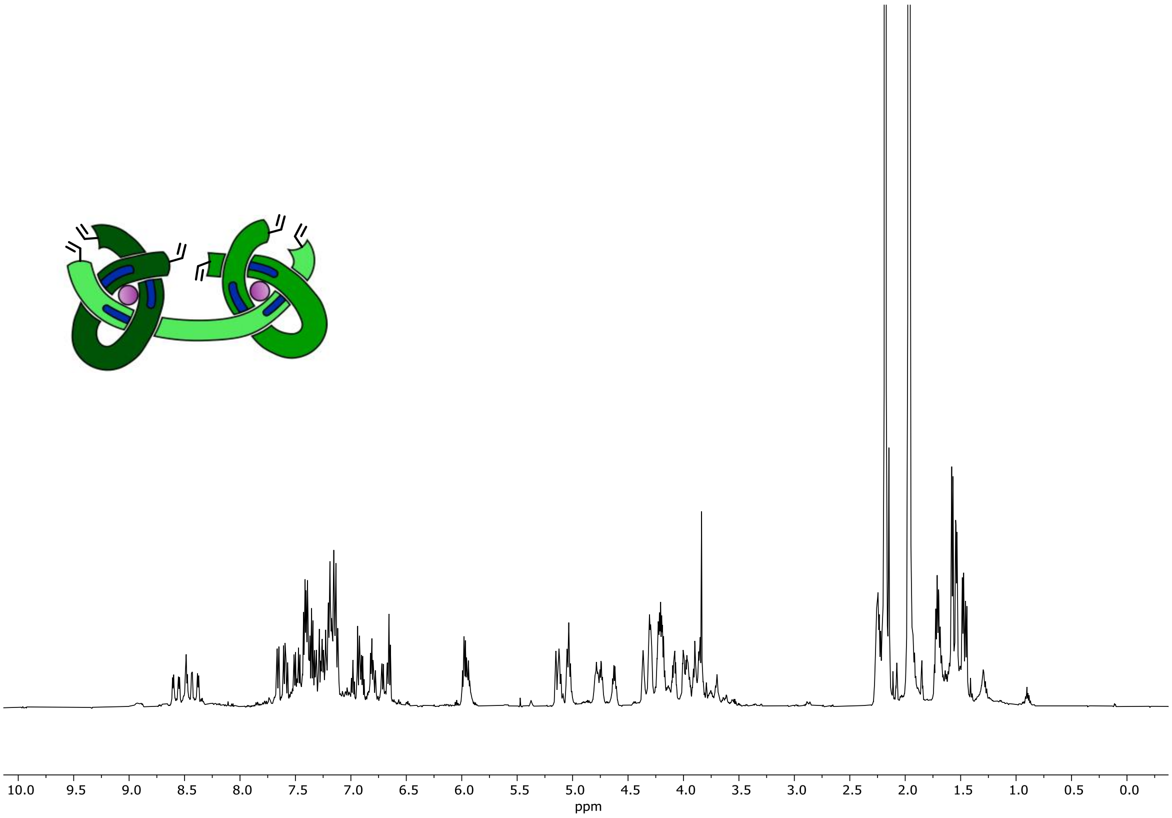

Figure S17. ^{1}H NMR spectrum (600 MHz, MeCN-d_3, 298 K) of crude reaction mixture of granny knot complex (Λ,Λ)-((*R*)$_4$-**L4**)$_3$•[Lu]$_2$ after 3 days at 80 °C.

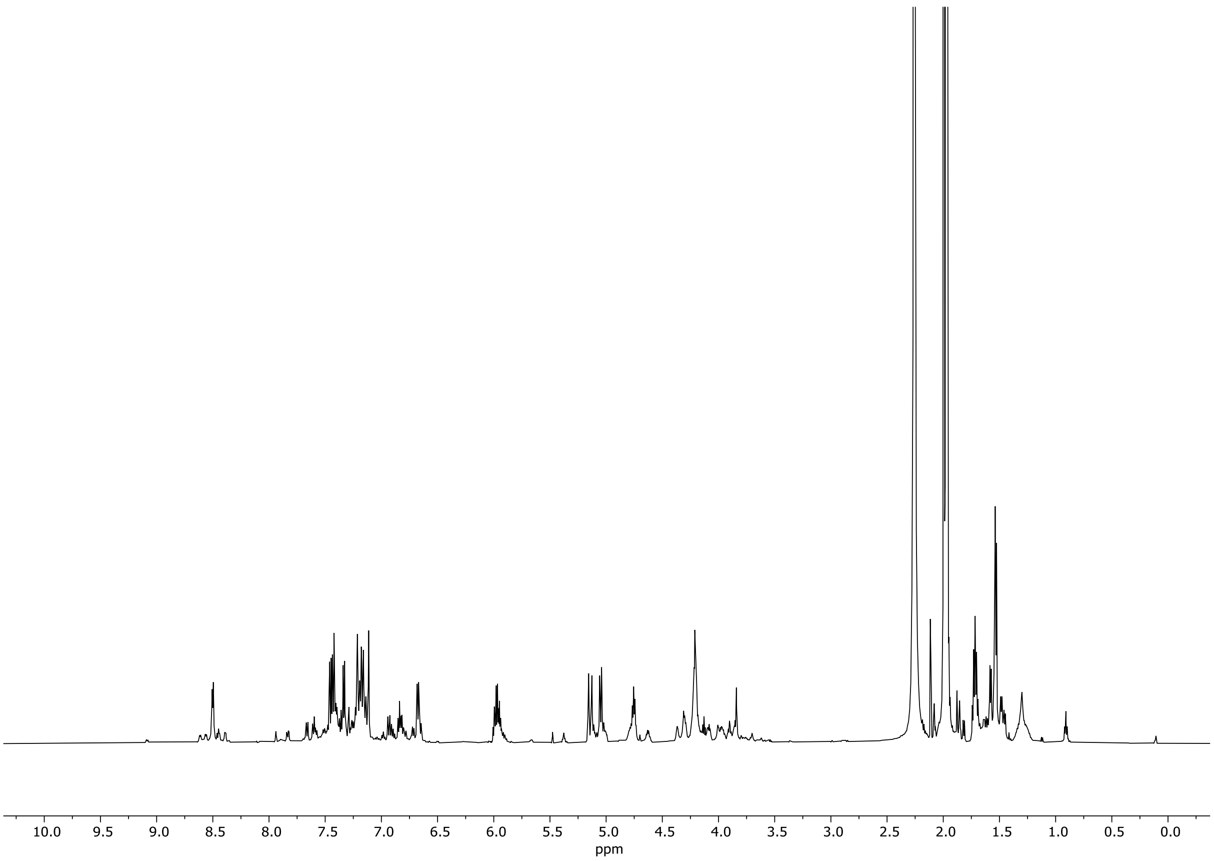

Figure S18. ^{1}H NMR spectrum (600 MHz, MeCN-d_3, 298 K) of complex mixture after 10 mins at r.t.

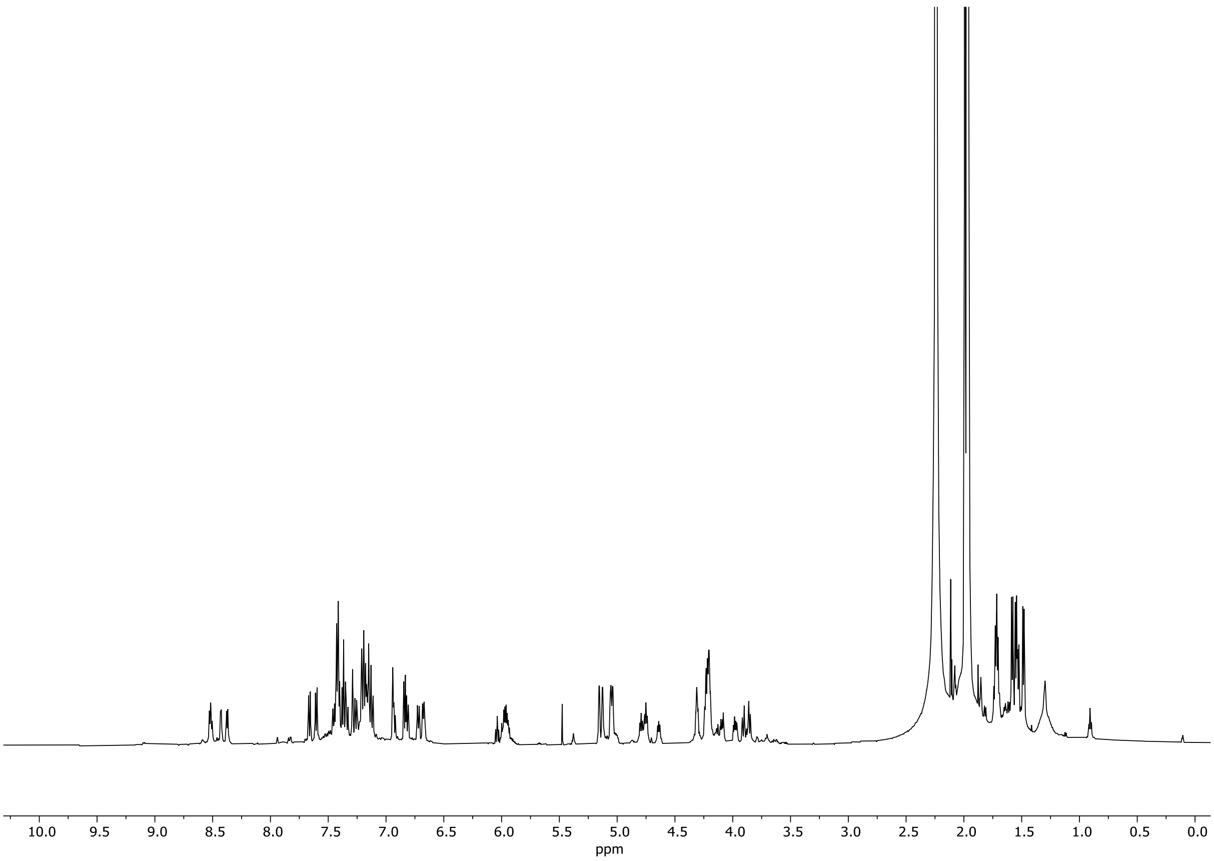

Figure S19. ^{1}H NMR spectrum (600 MHz, MeCN-d_3, 298 K) of complex mixture after 4 h at 80 °C.

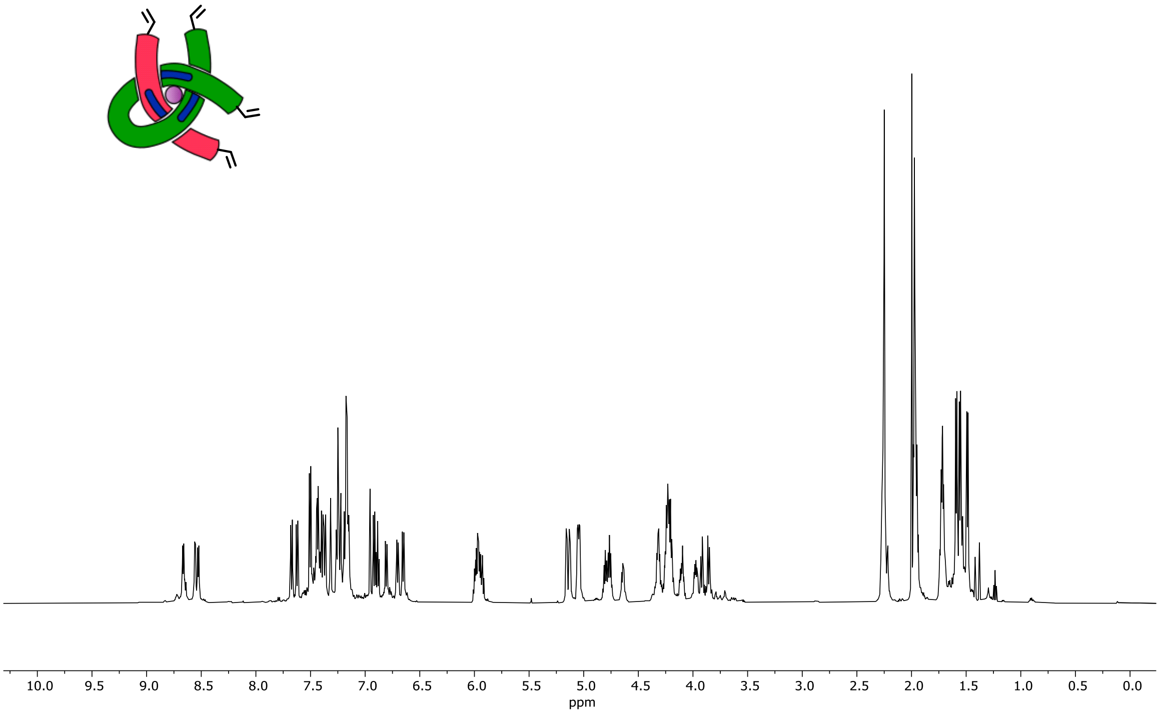

Figure S20. ^{1}H NMR spectrum (600 MHz, MeCN-d_3, 298 K) of reference open helicate Λ-{(*R*)$_2$-**L3**,(*R*)$_4$-**L4**•[Lu]} from 1:1 ratio of ligands (*R*)$_2$-**L3** and (*R*)$_4$-**L4** after 8 h at 80 °C.

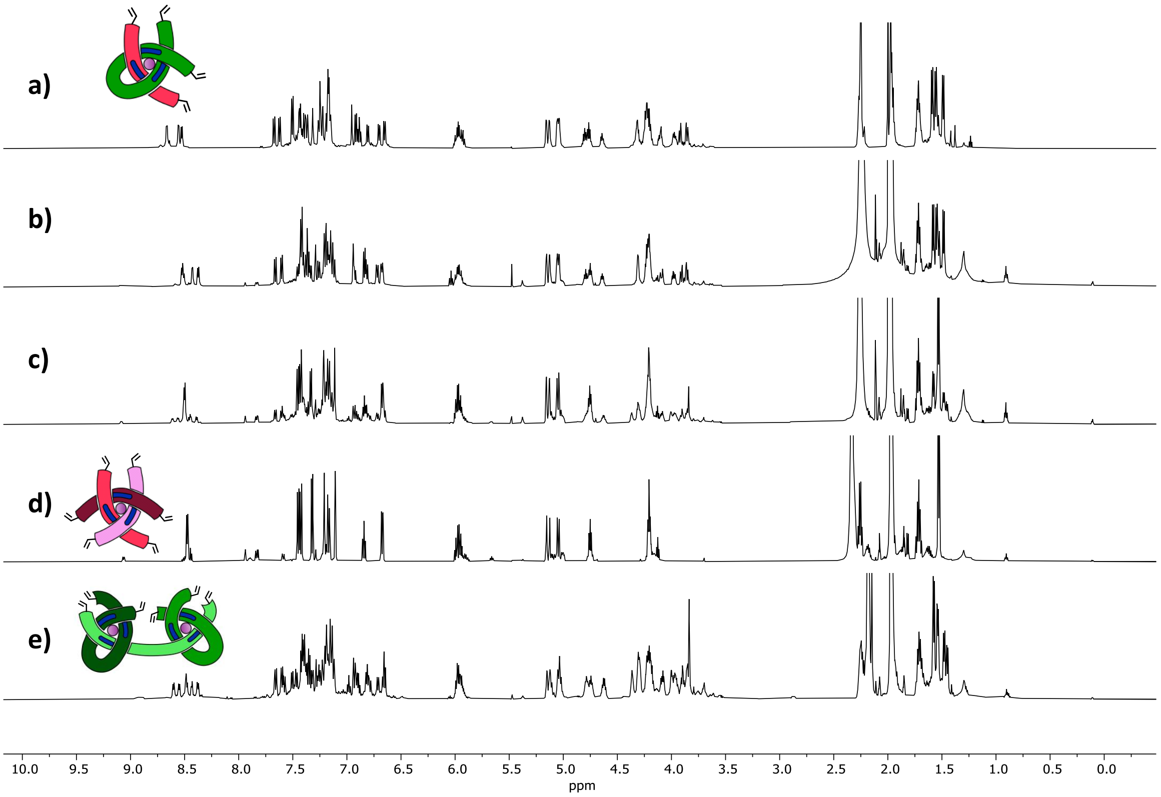

Figure S21. ^{1}H NMR spectral stack plot (600 MHz, MeCN-d_3, 298 K) showing rearrangement of homomeric complexes Λ-((*R*)$_2$-**L3**)$_3$•[Lu] and (Λ,Λ)-((*R*)$_4$-**L4**)$_3$•[Lu]$_2$ into heteromeric complex Λ-{(*R*)$_2$-**L3**,(*R*)$_4$-**L4**•[Lu]}: a) reference helicate Λ-{(*R*)$_2$-**L3**,(*R*)$_4$-**L4**•[Lu]}; b) reaction mixture after 4 h at 80 °C; c) reaction mixture after 10 mins at r.t.; d) open helicate Λ-((*R*)$_2$-**L3**)$_3$•[Lu]; e) open granny knot (Λ,Λ)-((*R*)$_4$-**L4**)$_3$•[Lu]$_2$.

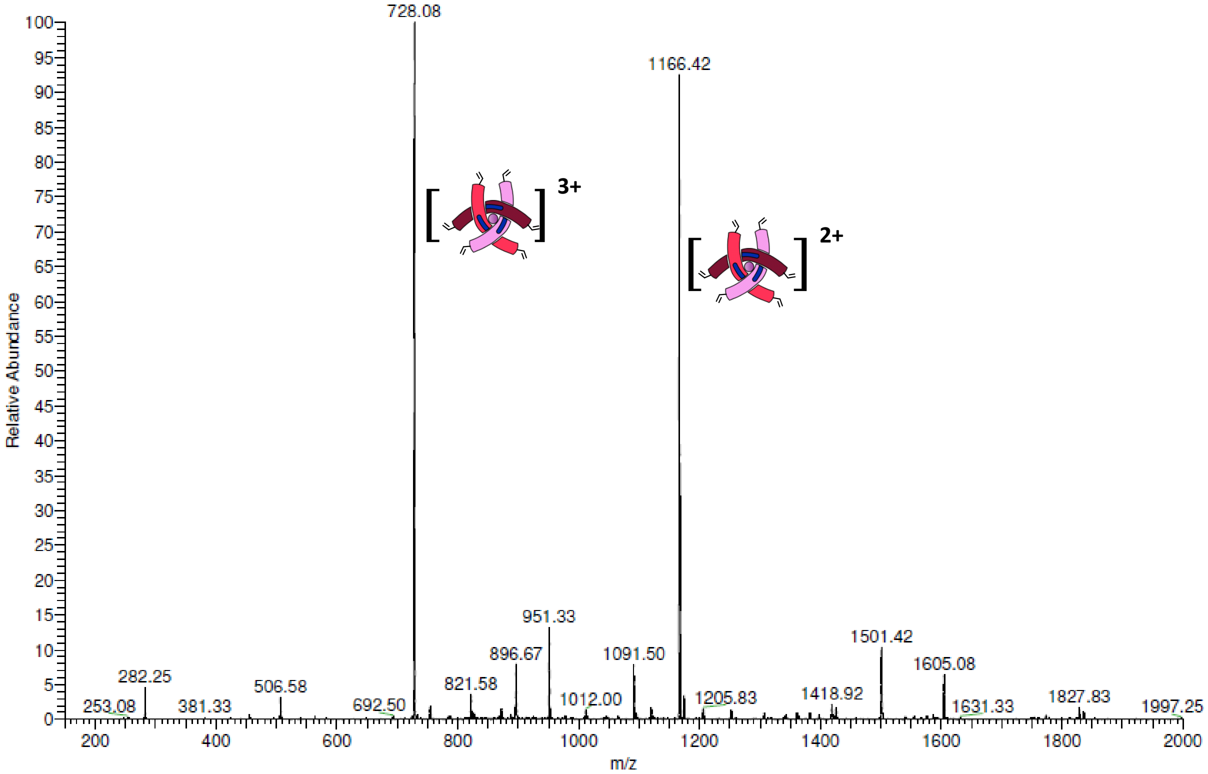

Figure S22. Low resolution ESI-MS(+) of open helicate Λ-((*R*)$_2$-**L3**)$_3$•[Lu] (all peaks observed as $[M{-}n(CF_3SO_3)]^{n+}$ adducts) after 24 h at 80 °C.

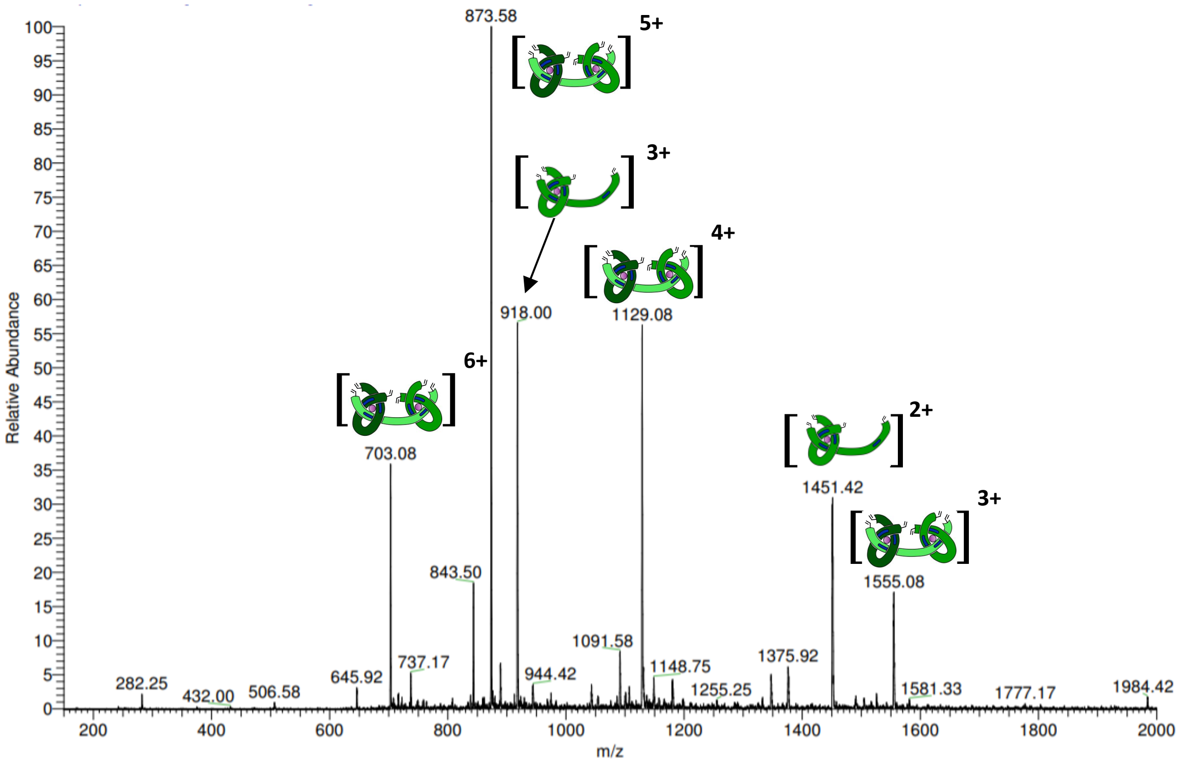

Figure S23. Low resolution ESI-MS(+) of open granny knot (Λ,Λ)-((*R*)$_4$-**L4**)$_3$•[Lu]$_2$ (all peaks observed as $[M{-}n(CF_3SO_3)]^{n+}$ adducts) after 3 days at 80 °C.

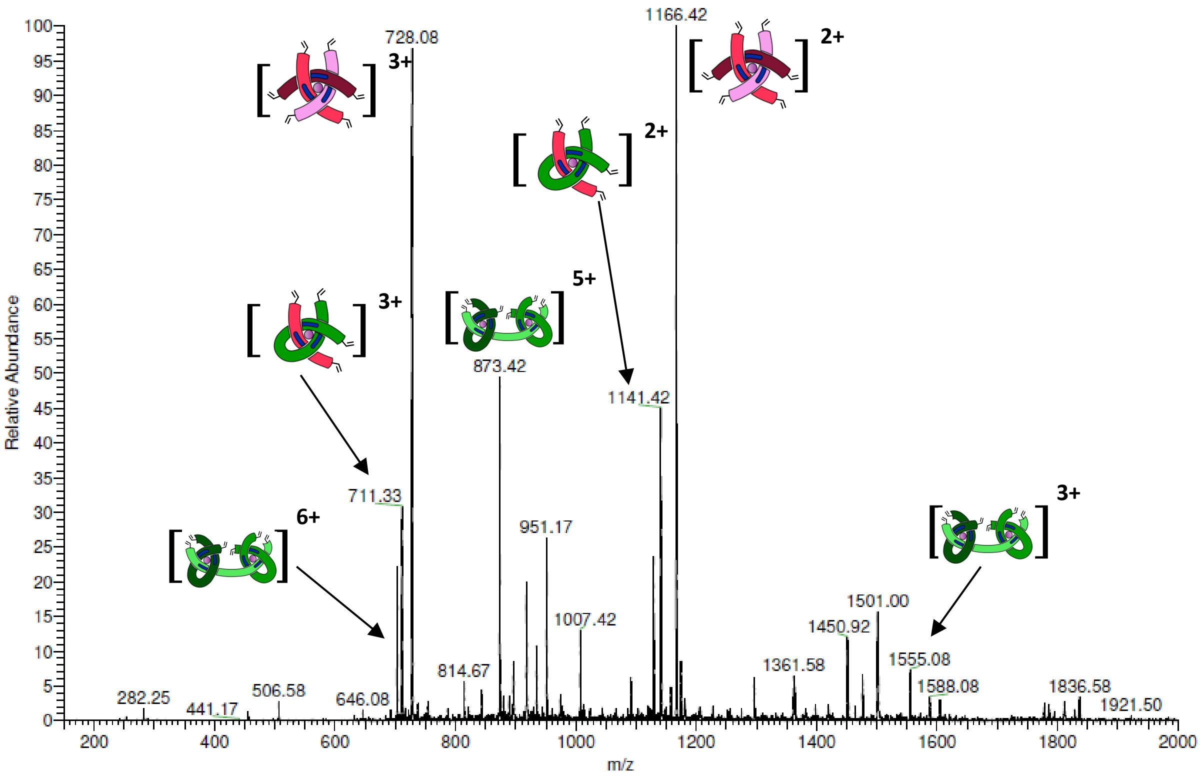

Figure S24. Low resolution ESI-MS(+) of complex mixture (all peaks observed as $[M{-}n(CF_3SO_3)]^{n+}$ adducts) after 10 mins at r.t.

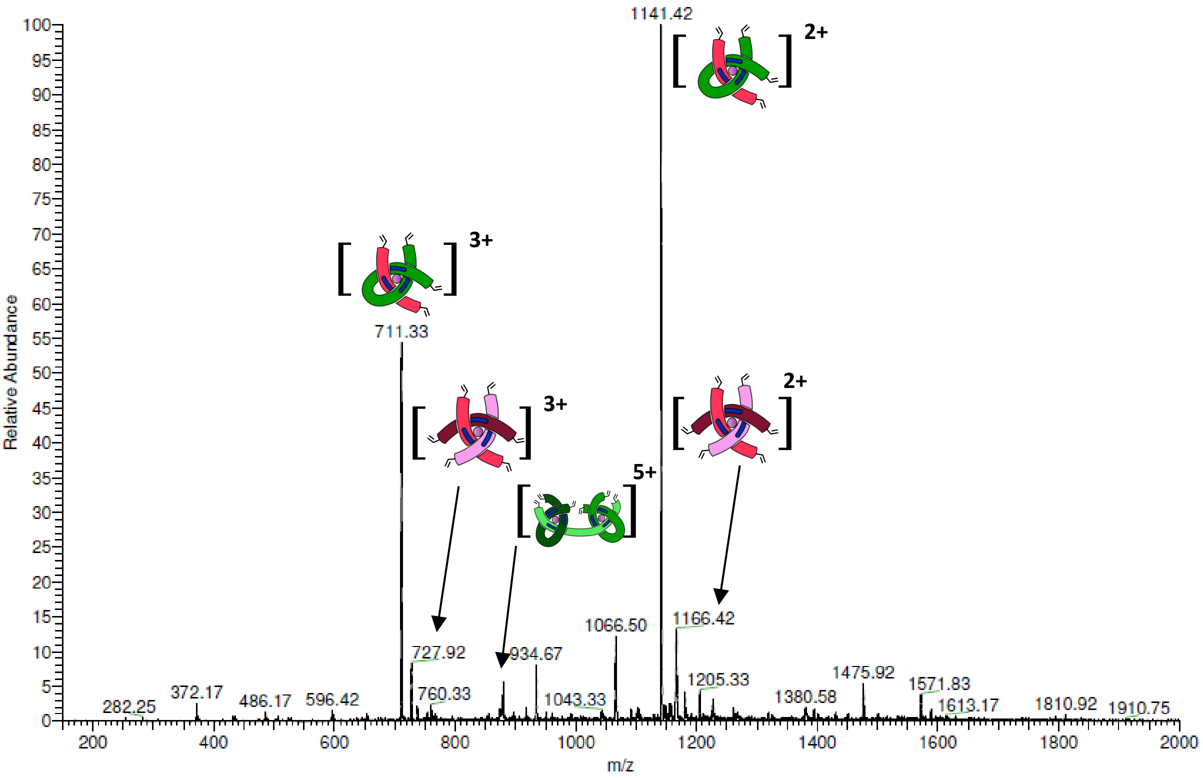

Figure S25. Low resolution ESI-MS(+) of complex mixture (all peaks observed as $[M{-}n(CF_3SO_3)]^{n+}$ adducts) after 4 h at 80 °C.

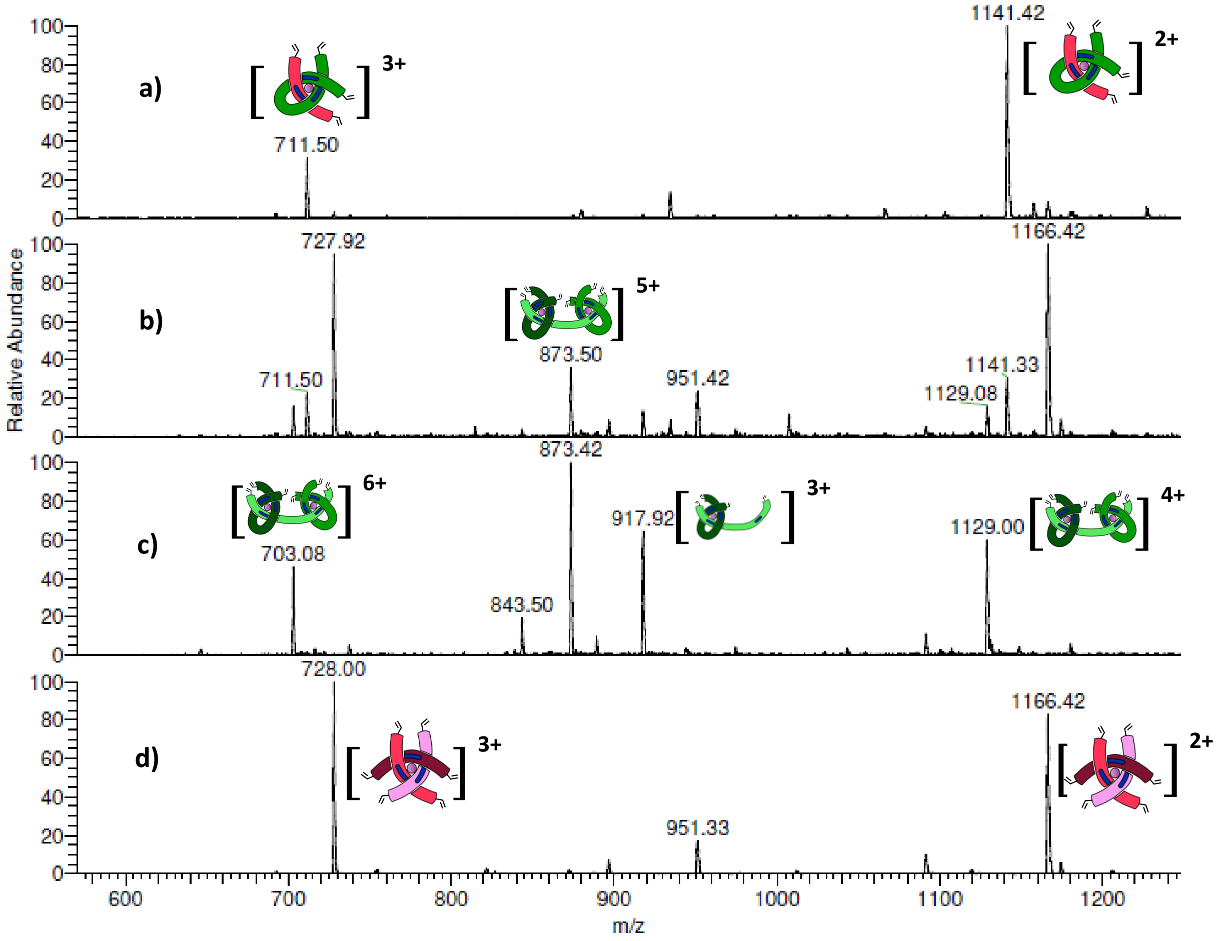

Figure S26. Partial (600-1200 m/z) mass spectral stack plot (+) showing rearrangement of homomeric complexes Λ-((*R*)$_2$-**L3**)$_3$•[Lu] and (Λ,Λ)-((*R*)$_4$-**L4**)$_3$•[Lu]$_2$ into heteromeric complex Λ-{(*R*)$_2$-**L3**,(*R*)$_4$-**L4**•[Lu]}: a) reaction mixture after 4 h at 80 °C; b) reaction mixture after 10 min at r.t; c) open granny knot (Λ,Λ)-((*R*)$_4$-**L4**)$_3$•[Lu]$_2$; d) open helicate Λ-((*R*)$_2$-**L3**)$_3$•[Lu].

S4.7 In-situ rearrangement of triskelion complex (Λ$_3$,Λ)-((R)$_8$-L5)$_3$•[Lu]$_4$ and granny complex (Λ,Λ)-((R)$_4$-L4)$_3$•[Lu]$_2$ to form granny complex (Λ,Λ)-{(R)$_4$-L4,(R)$_8$-L5•[Lu]$_2$}

(Λ$_3$,Λ)-((R)$_8$-L5)$_3$•[Lu]$_4$

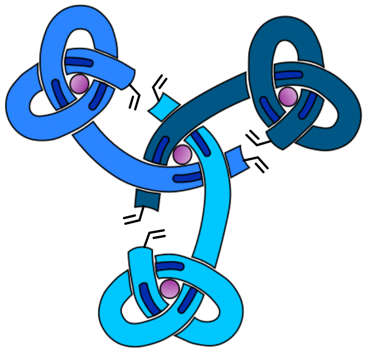

To a solution of (R)$_8$-**L5** (1.59 mg, 0.63 µmol) in MeCN-d_3 (0.4 mL) was added $Lu(CF_3SO_3)_3$ (0.52 mg, 0.84 µmol) in MeCN-d_3 (0.1 mL). The solution was stirred at 80 °C and complexation was monitored by ^{1}H NMR spectroscopy and ESI-MS. After 5 days, the crude solution of (Λ$_3$,Λ)-((R)$_8$-**L5**)$_3$•[Lu]$_4$ (1.74 mg, 0.21 µmol) was cooled to room temperature and used in the subsequent rearrangement experiment without any change to reaction concentration.[4]

Rearrangement experiment

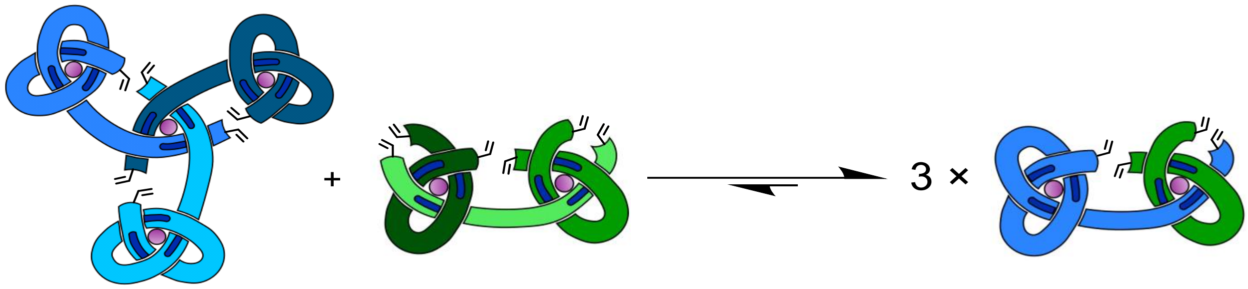

A 0.42 mM solution of the (Λ$_3$,Λ)-((R)$_8$-**L5**)$_3$•[Lu]$_4$ complex prepared above (0.2 mL) was added to a 0.42 mM solution of (Λ,Λ)-((R)$_4$-**L4**)$_3$•[Lu]$_2$ (see section S4.6) (0.2 mL). The mixture was left unstirred for 1 hour at room temperature and then stirred for a further 16 hours at 80 °C. The rearrangement reaction was monitored by ^{1}H NMR spectroscopy and ESI-MS until no further change was observed.

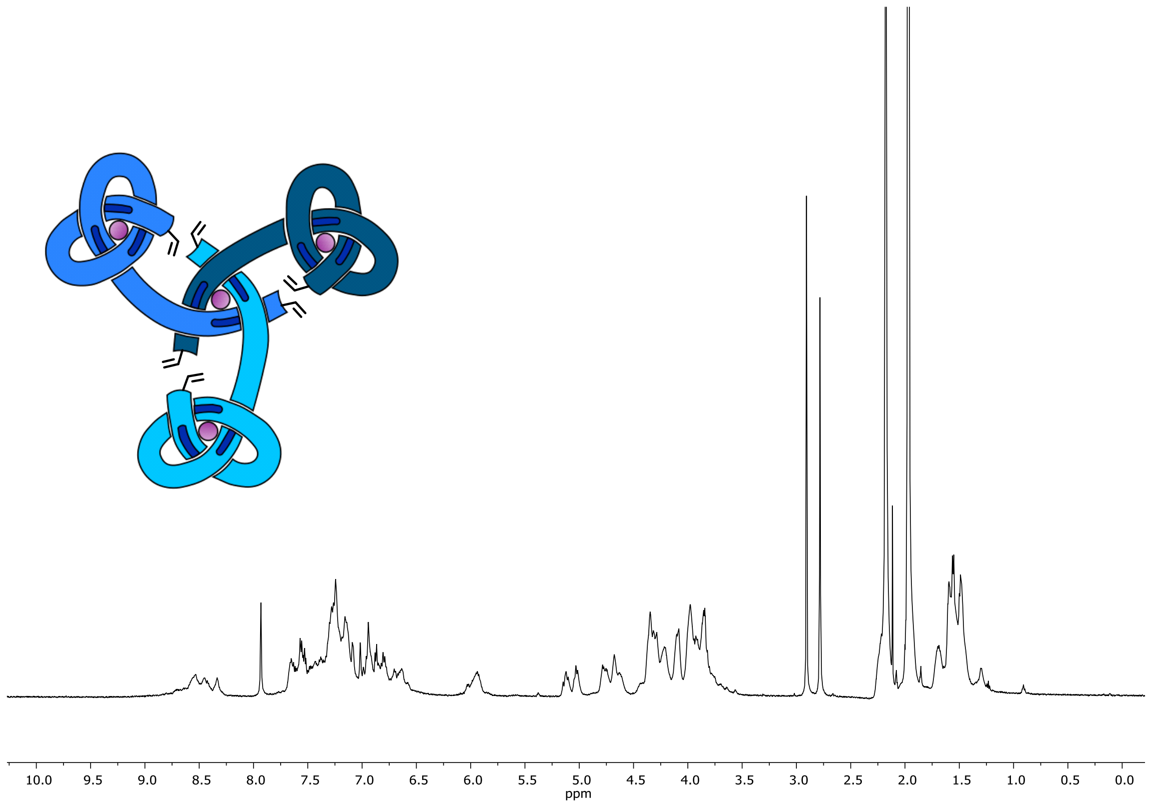

Figure S27. ^{1}H NMR spectrum (600 MHz, MeCN-d_3, 298 K) of crude reaction mixture of triskelion knot complex (Λ_3,Λ)-((*R*)$_8$-**L5**)$_3$•[Lu]$_4$ after 5 days at 80 °C.

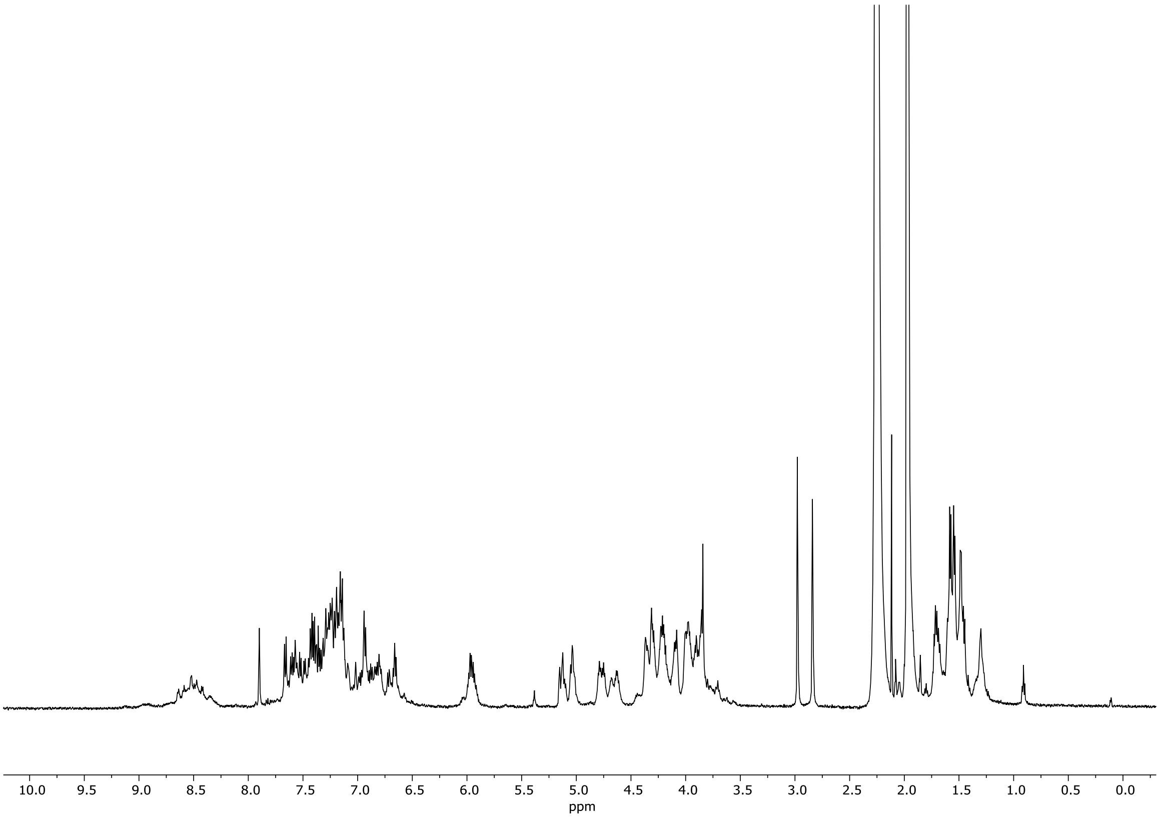

Figure S28. ^{1}H NMR spectrum (600 MHz, MeCN-d_3, 298 K) of complex mixture after 1 h at r.t.

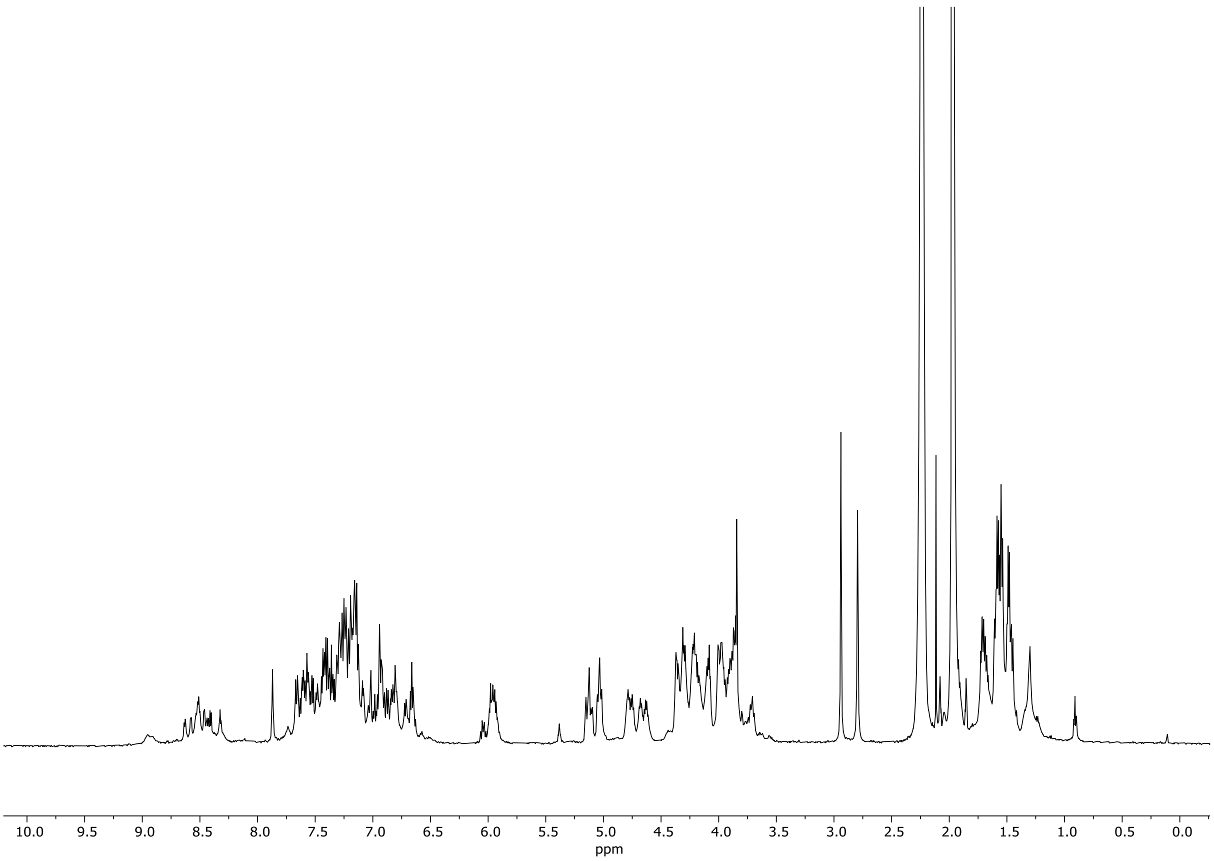

Figure S29. ^{1}H NMR spectrum (600 MHz, MeCN-d_3, 298 K) of complex mixture after 16 h at 80 °C.

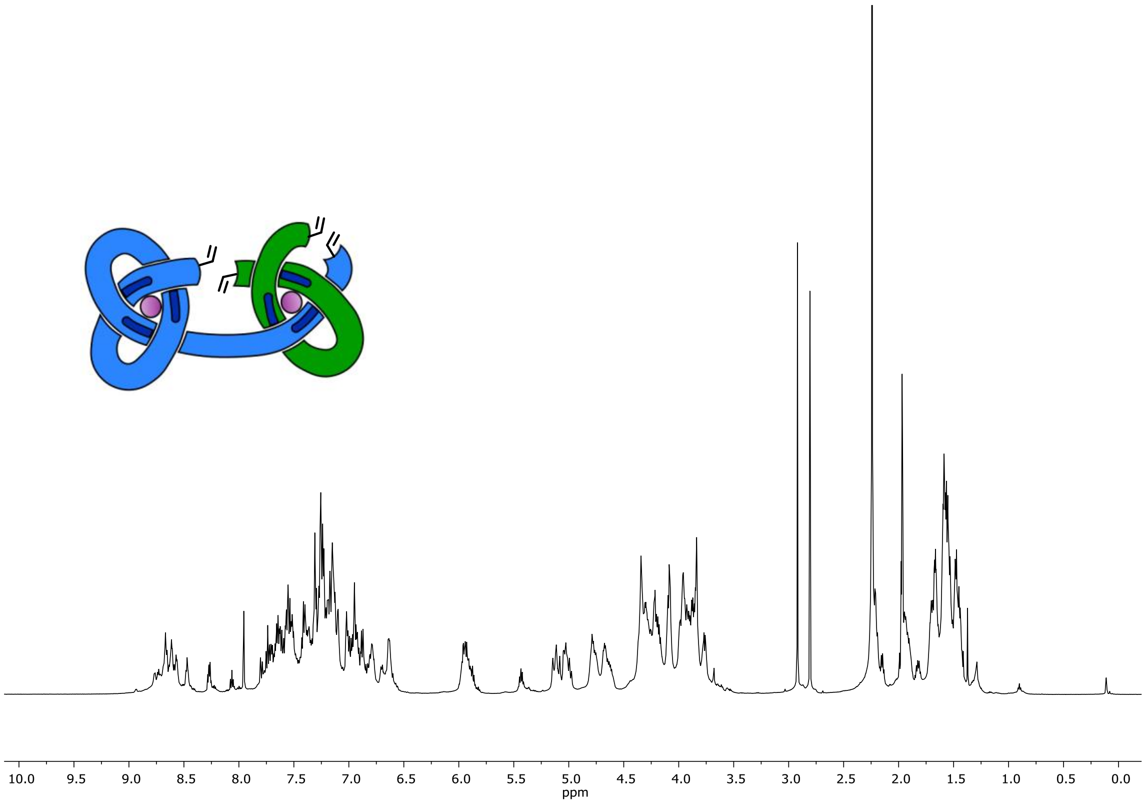

Figure S30. ^{1}H NMR spectrum (600 MHz, MeCN-d_3, 298 K) of reference open granny knot complex (Λ,Λ)-{(R)$_4$-**L4**,(R)$_8$-**L5•**[Lu]$_2$} from 1:1 ratio of ligands (R)$_4$-**L4** and (R)$_8$-**L5** after 3 days at 80 °C.

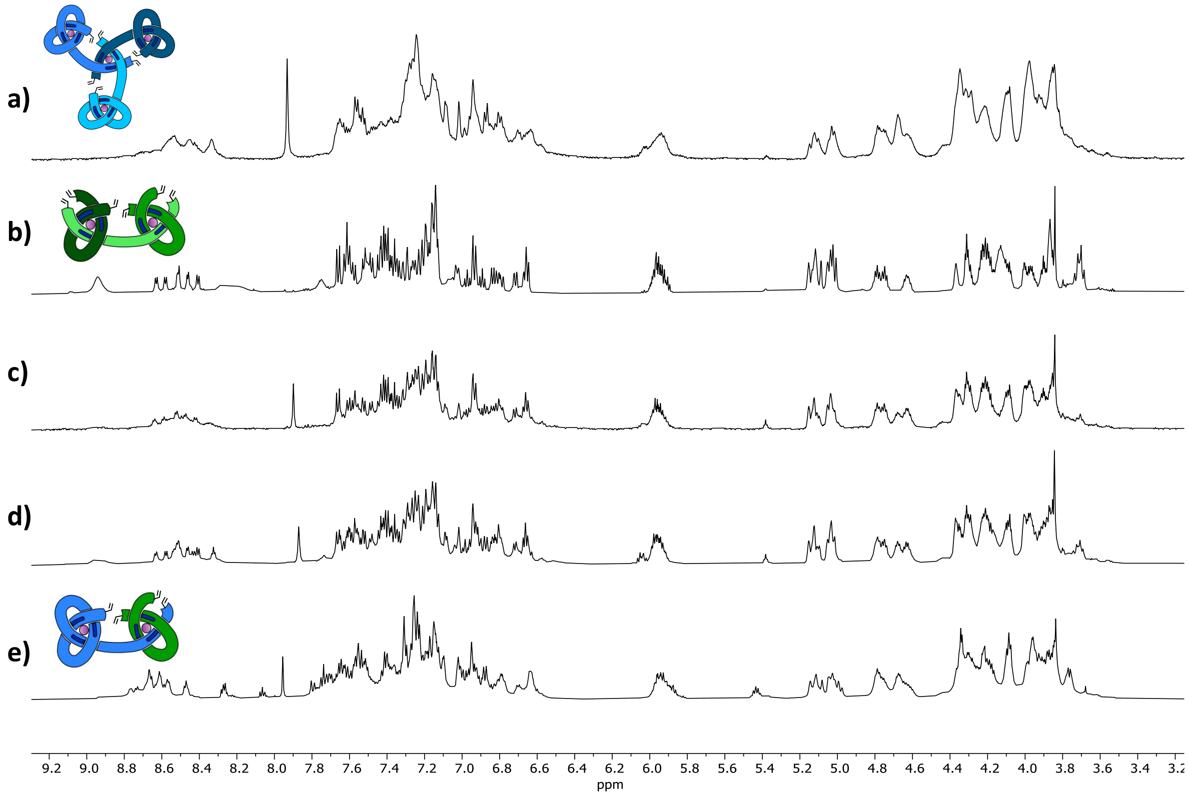

Figure S31. Partial (3.2-9.2 ppm) ^{1}H NMR spectral stack plot (600 MHz, MeCN-d_3, 298 K) showing rearrangement of homomeric knots (Λ,Λ)-((*R*)$_4$-**L4**)$_3$•[Lu]$_2$ and (Λ$_3$,Λ)-((*R*)$_8$-**L5**)$_3$•[Lu]$_4$ into heteromeric granny knot (Λ,Λ)-{(*R*)$_4$-**L4**,(*R*)$_8$-**L5**•[Lu]$_2$}: a) open triskelion knot (Λ$_3$,Λ)-((*R*)$_8$-**L5**)$_3$•[Lu]$_4$; b) open granny knot (Λ,Λ)-((*R*)$_4$-**L4**)$_3$•[Lu]$_2$; c) reaction mixture after 1 h at r.t; d) reaction mixture after 16 h at 80 °C; e) reference open granny knot (Λ,Λ)-{(*R*)$_4$-**L4**,(*R*)$_8$-**L5**•[Lu]$_2$}.

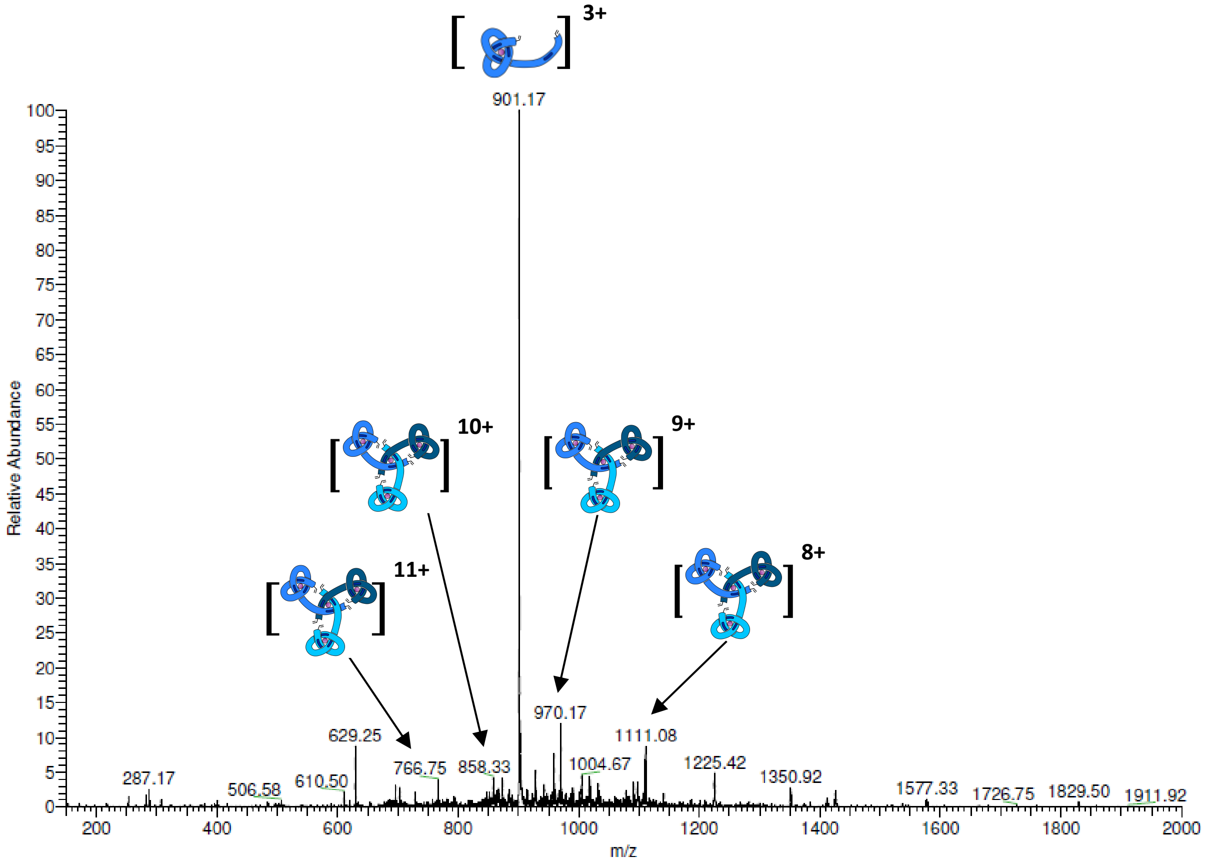

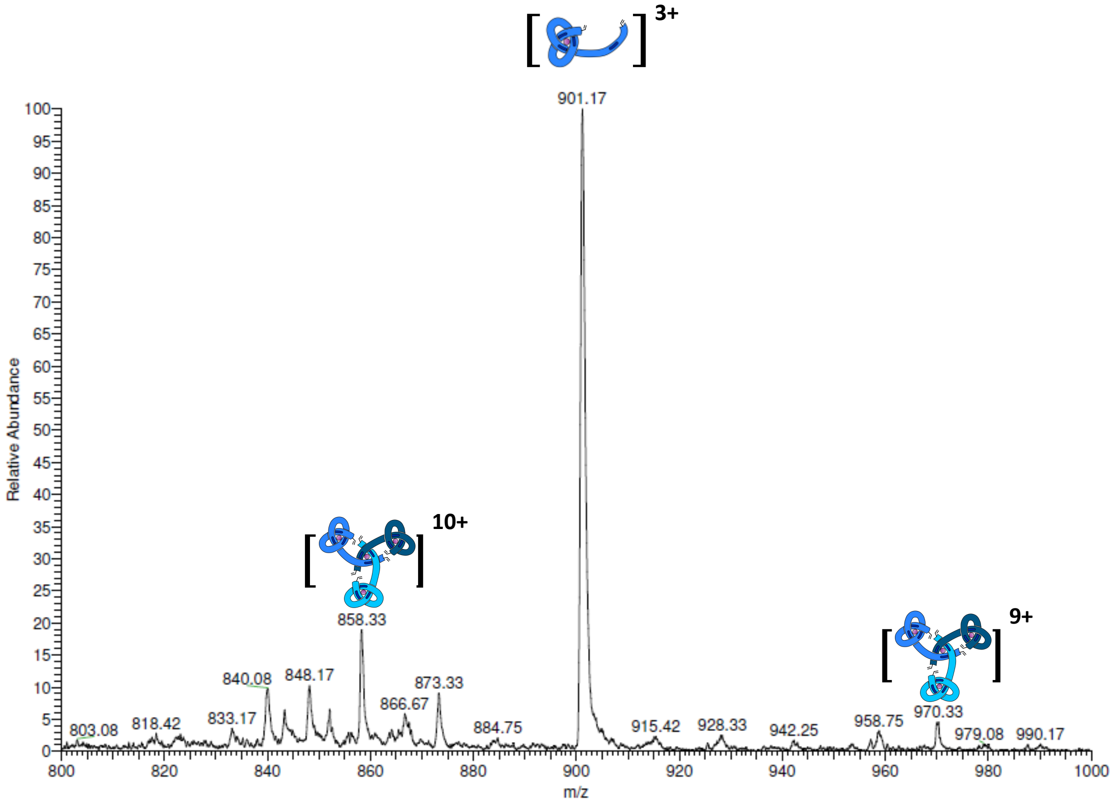

Figure S32. Low resolution ESI-MS(+) of open triskelion knot (Λ_3,Λ)-((R)$_8$-**L5**)$_3$•[Lu]$_4$ (all peaks observed as [M−n(CF_3SO_3)]$^{n+}$ adducts) after 5 days at 80 °C (top), and enhanced view of 800-1000 m/z region (bottom).

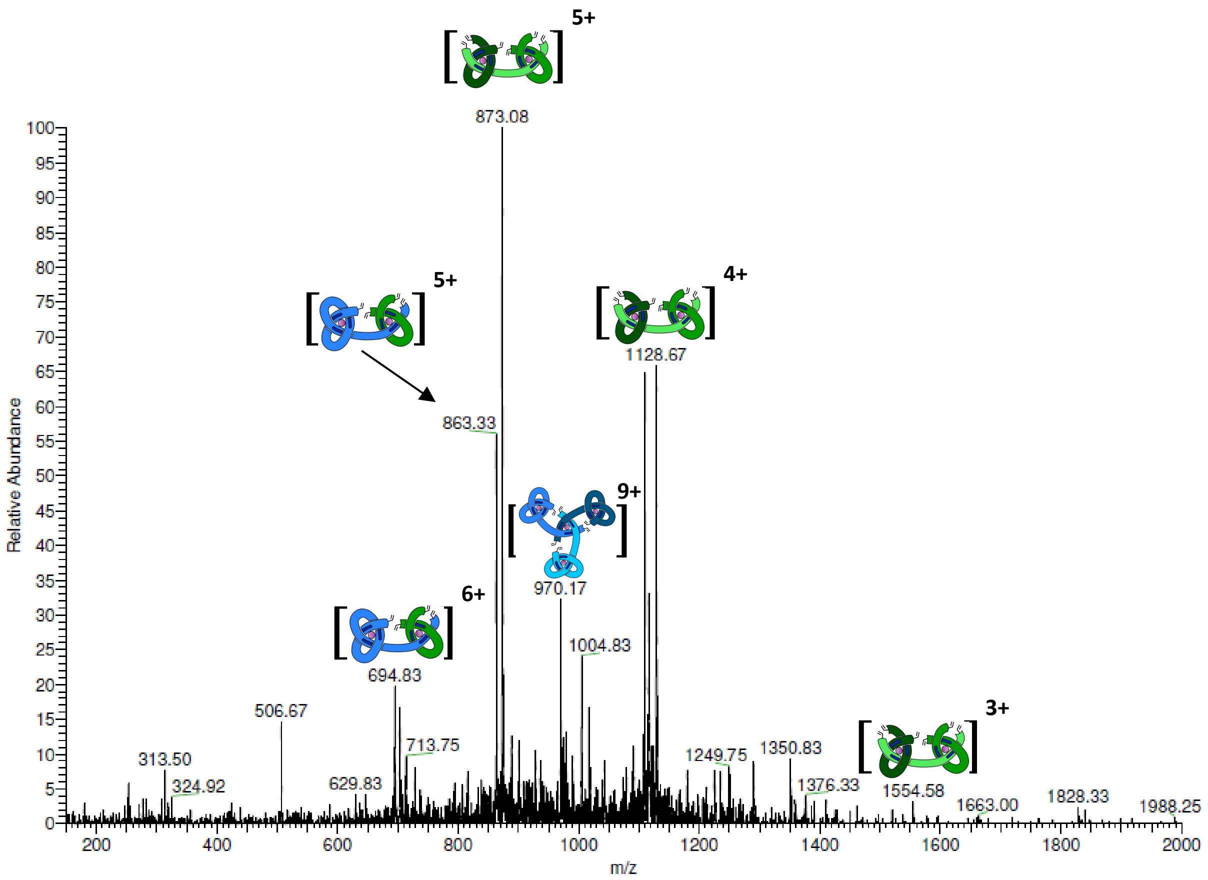

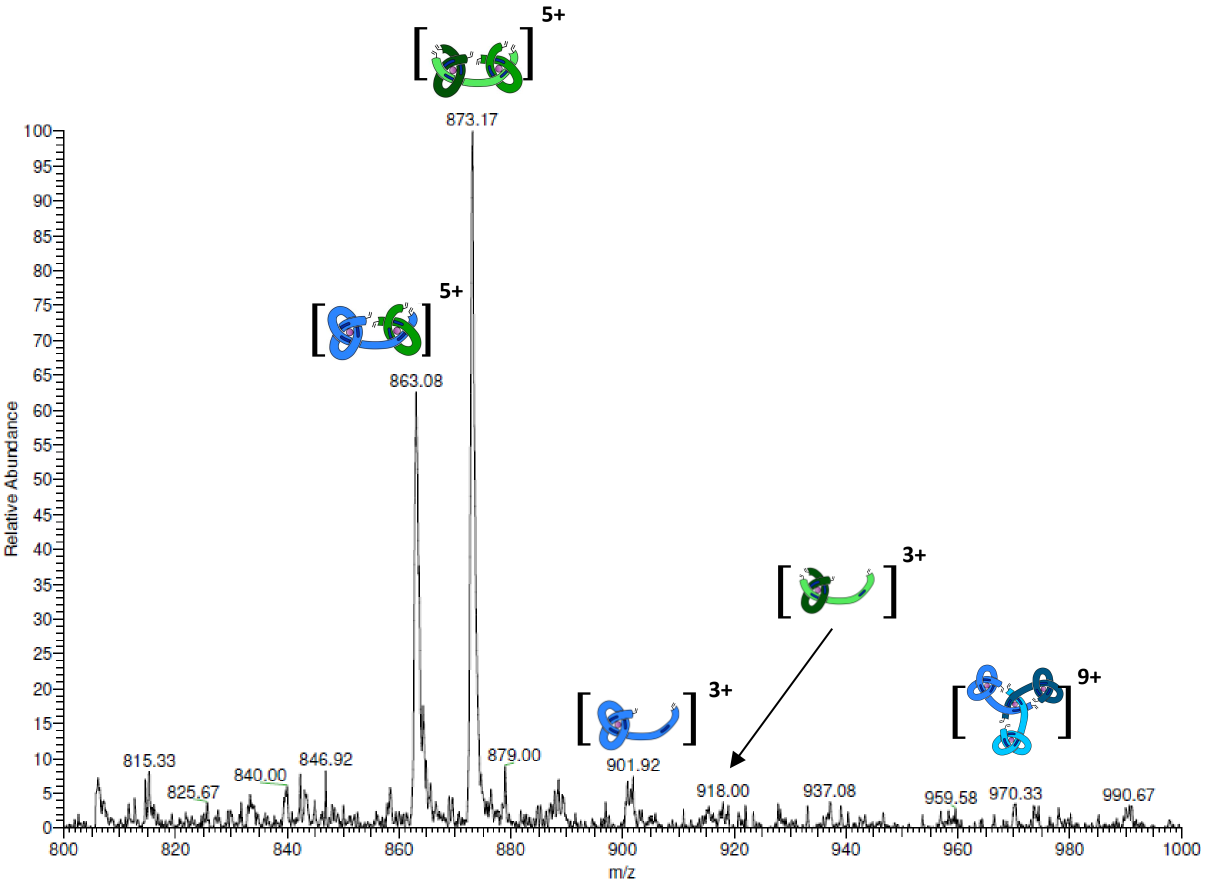

Figure S33. Low resolution ESI-MS(+) of complex mixture (all peaks observed as $[M{-}n(CF_3SO_3)]^{n+}$ adducts) after 1 h at r.t. (top), and enhanced view of 800-1000 m/z region (bottom).

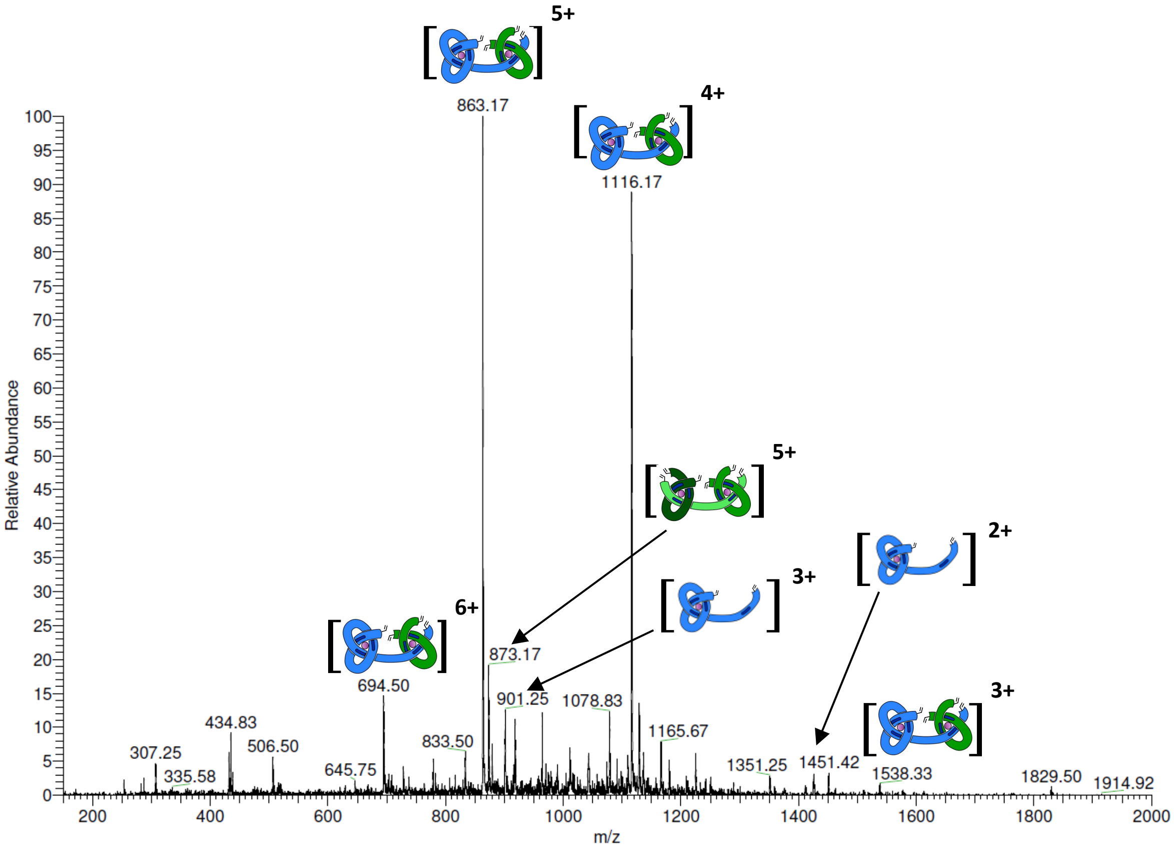

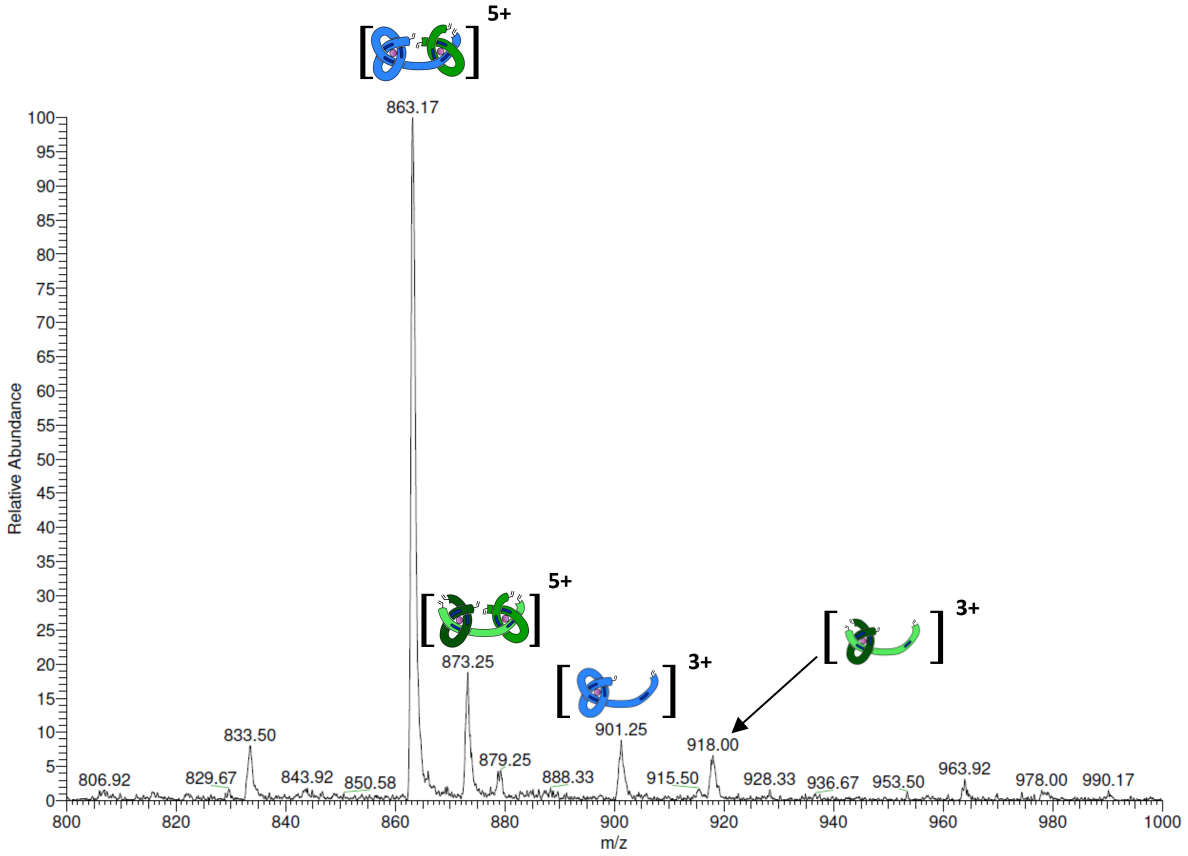

Figure S34. Low resolution ESI-MS(+) of complex mixture (all peaks observed as $[M{-}n(CF_3SO_3)]^{n+}$ adducts) after 16 h at 80 °C (top), and enhanced view of 800-1000 m/z region (bottom).

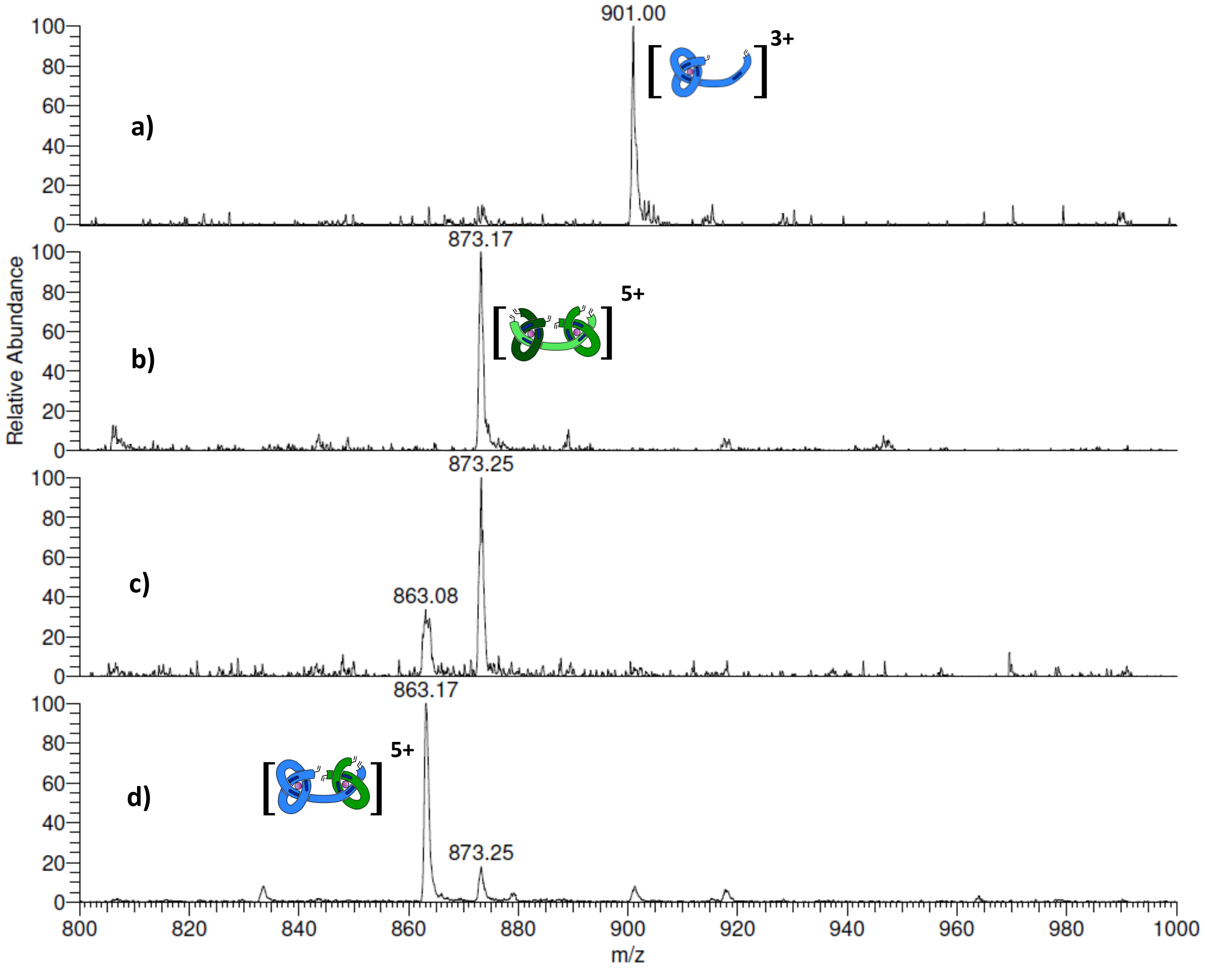

Figure S35. Partial (800-1000 m/z) mass spectral stack plot (+) showing rearrangement of homomeric open complexes (Λ,Λ)-((*R*)$_4$-**L4**)$_3$•[Lu]$_2$ and (Λ$_3$,Λ)-((*R*)$_8$-**L5**)$_3$•[Lu]$_4$ into heteromeric open granny complex (Λ,Λ)-{(*R*)$_4$-**L4**,(*R*)$_8$-**L5**•[Lu]$_2$}: a) open triskelion knot complex (Λ$_3$,Λ)-((*R*)$_8$-**L5**)$_3$•[Lu]$_4$ (complex is known to fragment under electrospray conditions)[4]; b) open granny knot complex (Λ,Λ)-((*R*)$_4$-**L4**)$_3$•[Lu]$_2$; c) reaction mixture after 1 h at r.t; d) reaction mixture after 16 h at 80 °C.

S5. NMR Spectra

S5.1 ^{1}H and ^{13}C NMR Spectra

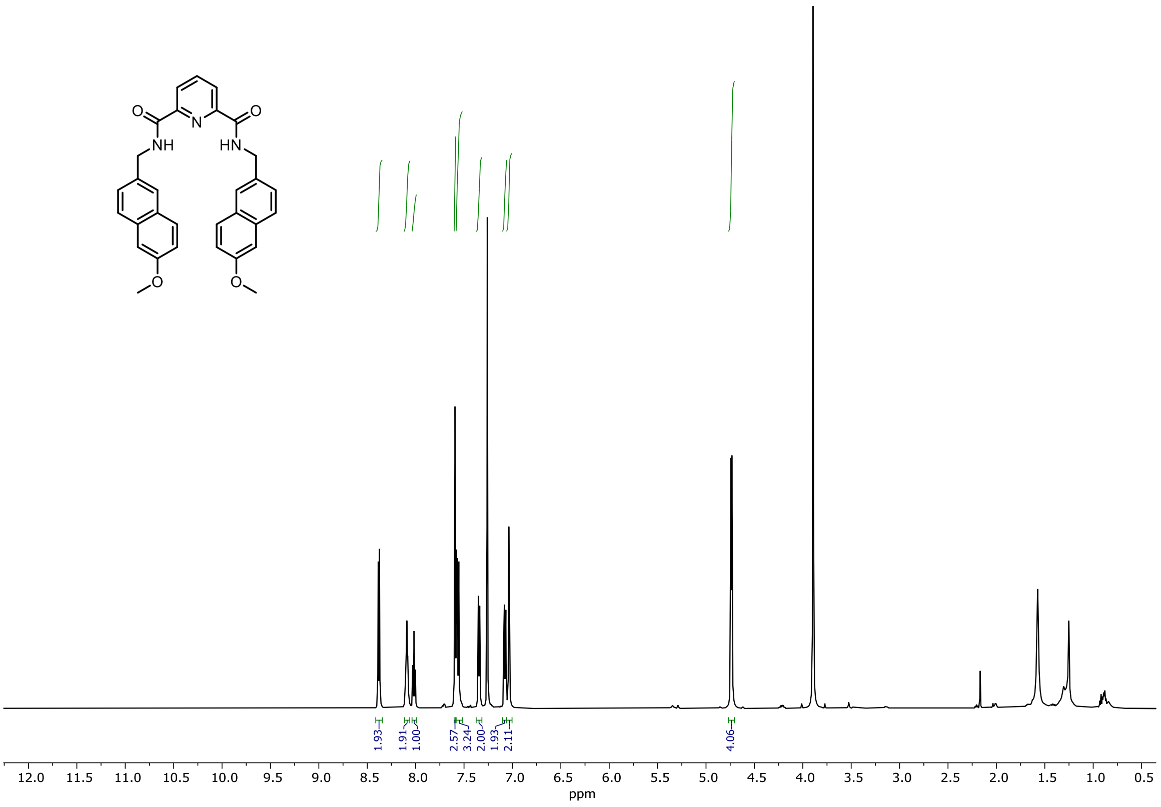

Figure S36. ^{1}H NMR (600 MHz, $CDCl_3$, 298 K) of **S2**.

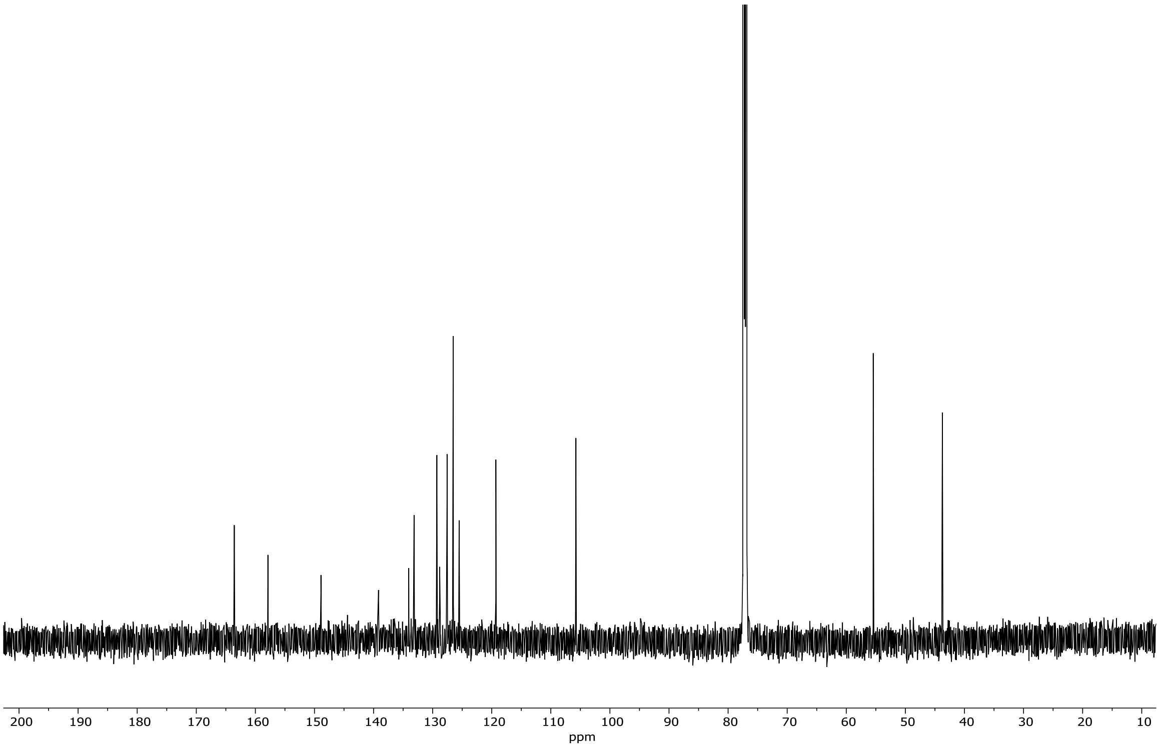

Figure S37. ^{13}C NMR (151 MHz, $CDCl_3$, 298 K) of **S2**.

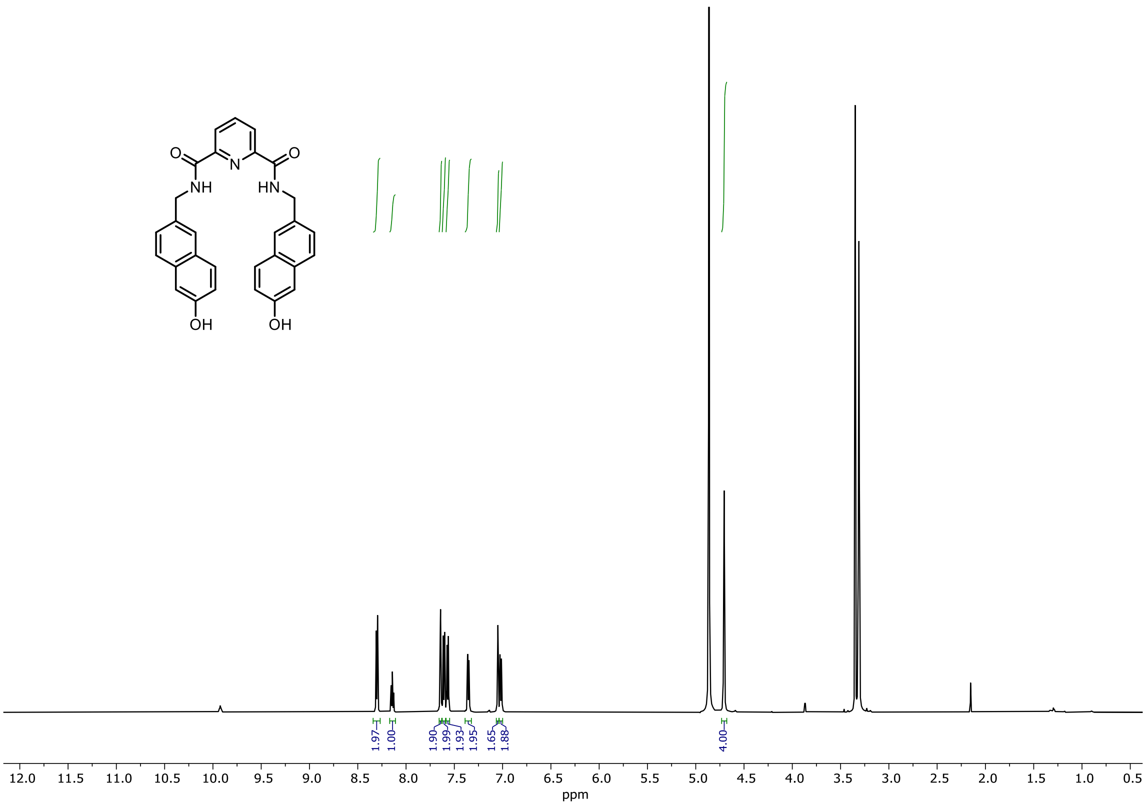

Figure S38. ^{1}H NMR (600 MHz, MeOD-d_4, 298 K) of **S3**.

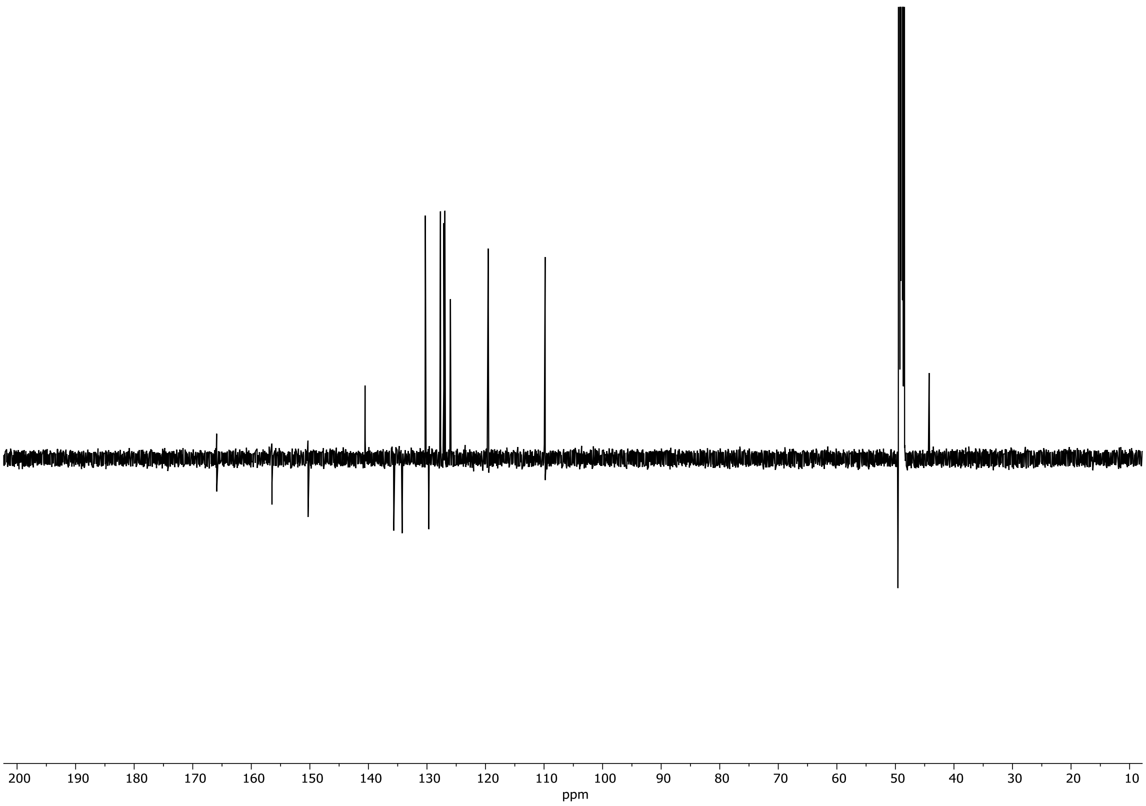

Figure S39. DEPTQ ^{13}C NMR (151 MHz, MeOD-d_4, 298 K) of **S3**.

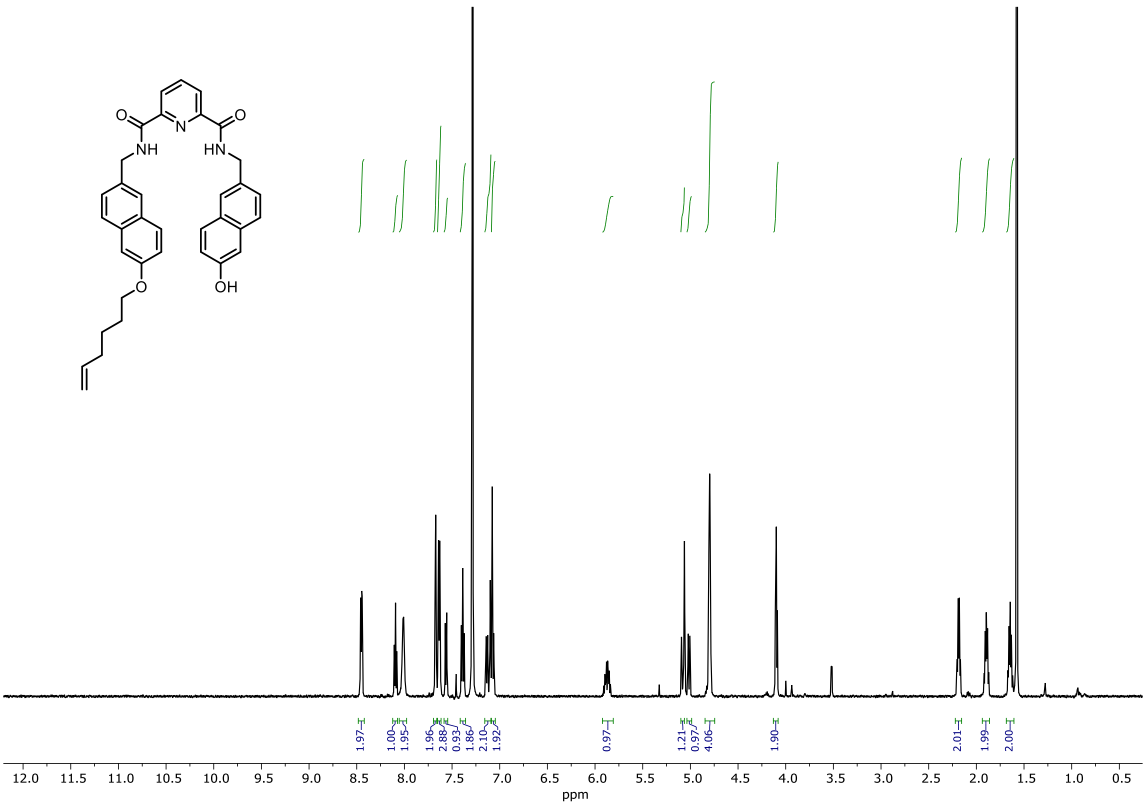

Figure S40. ^{1}H NMR (600 MHz, $CDCl_3$, 298 K) of **S4**.

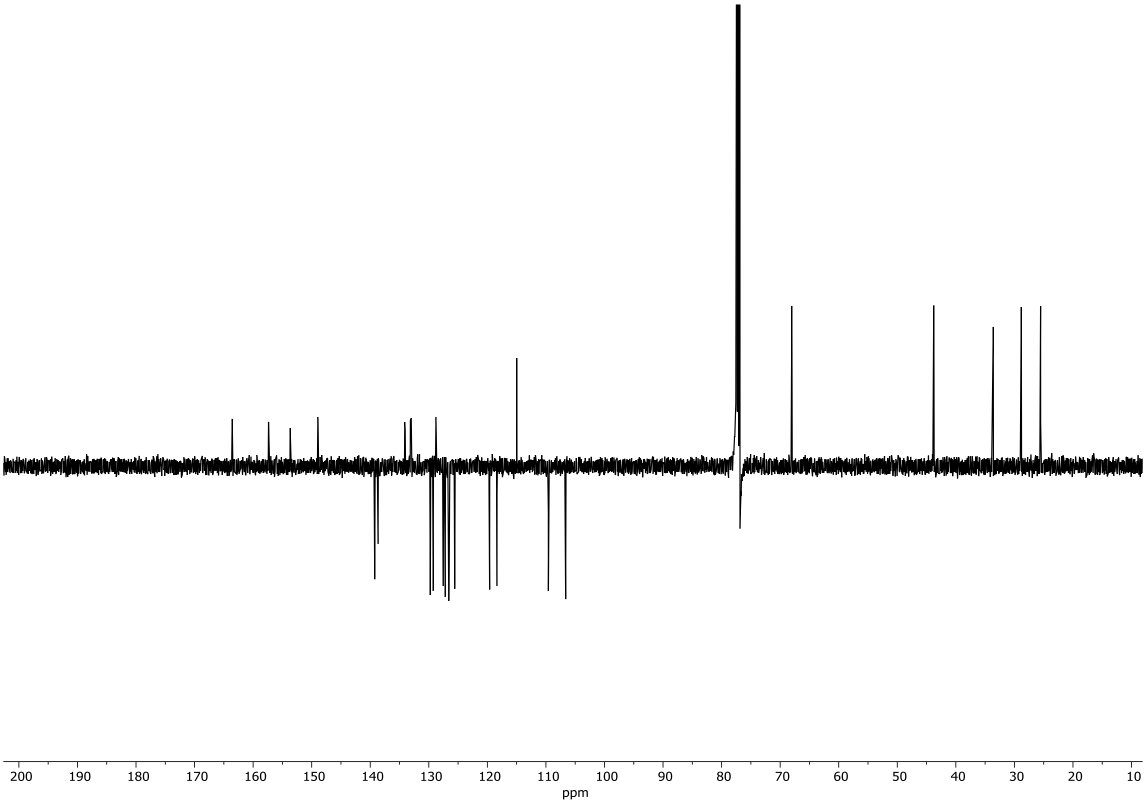

Figure S41. DEPTQ ^{13}C NMR (151 MHz, $CDCl_3$, 298 K) of **S4**.

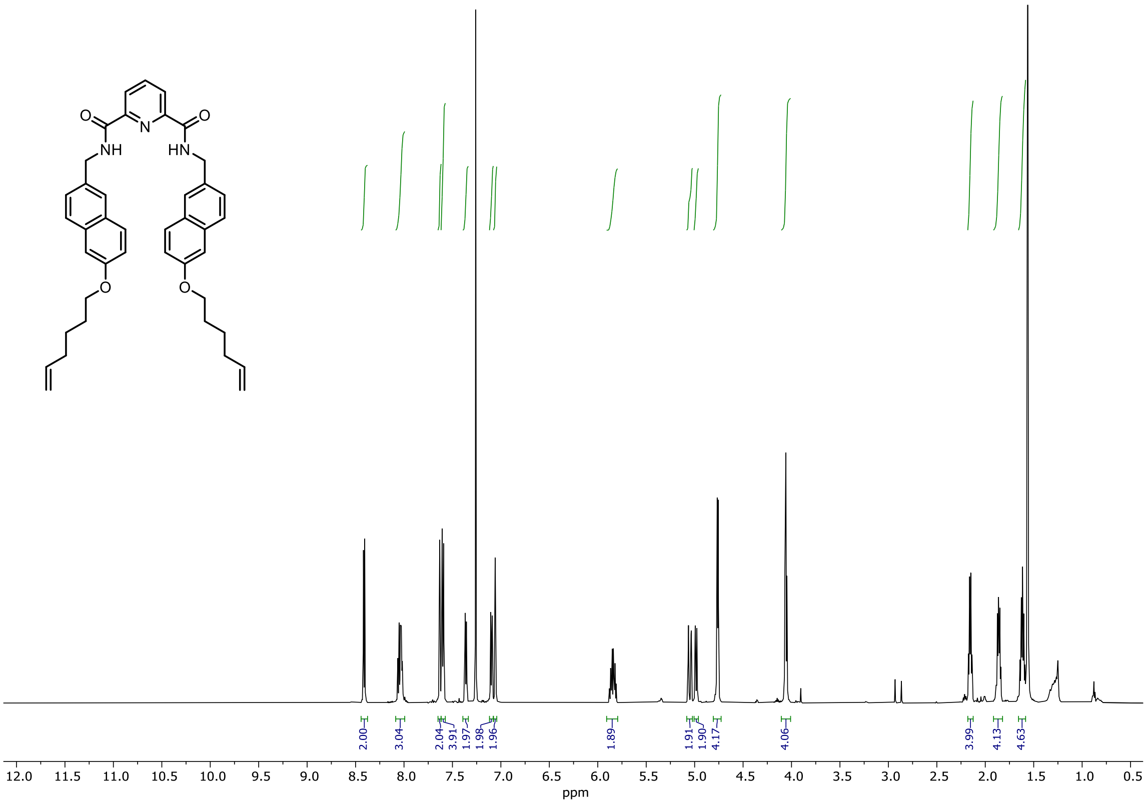

Figure S42. ^{1}H NMR (600 MHz, $CDCl_3$, 298 K) of **L1**.

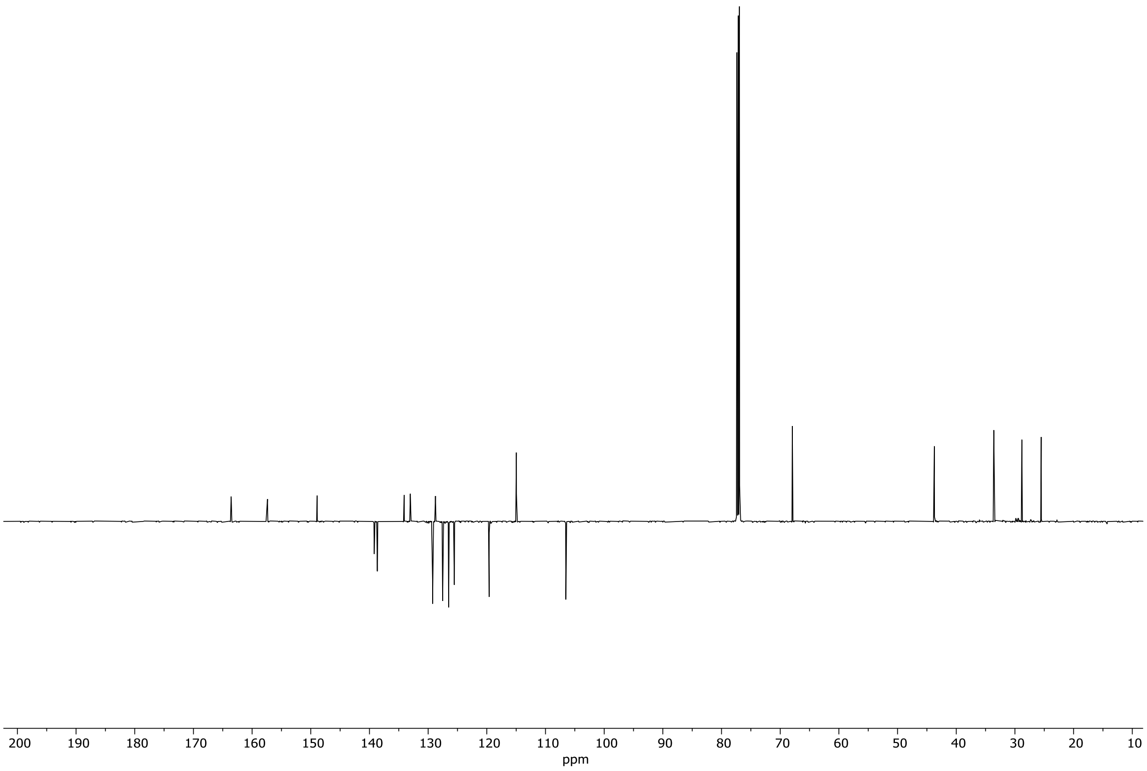

Figure S43. DEPTQ ^{13}C NMR (151 MHz, $CDCl_3$, 298 K) of **L1**.

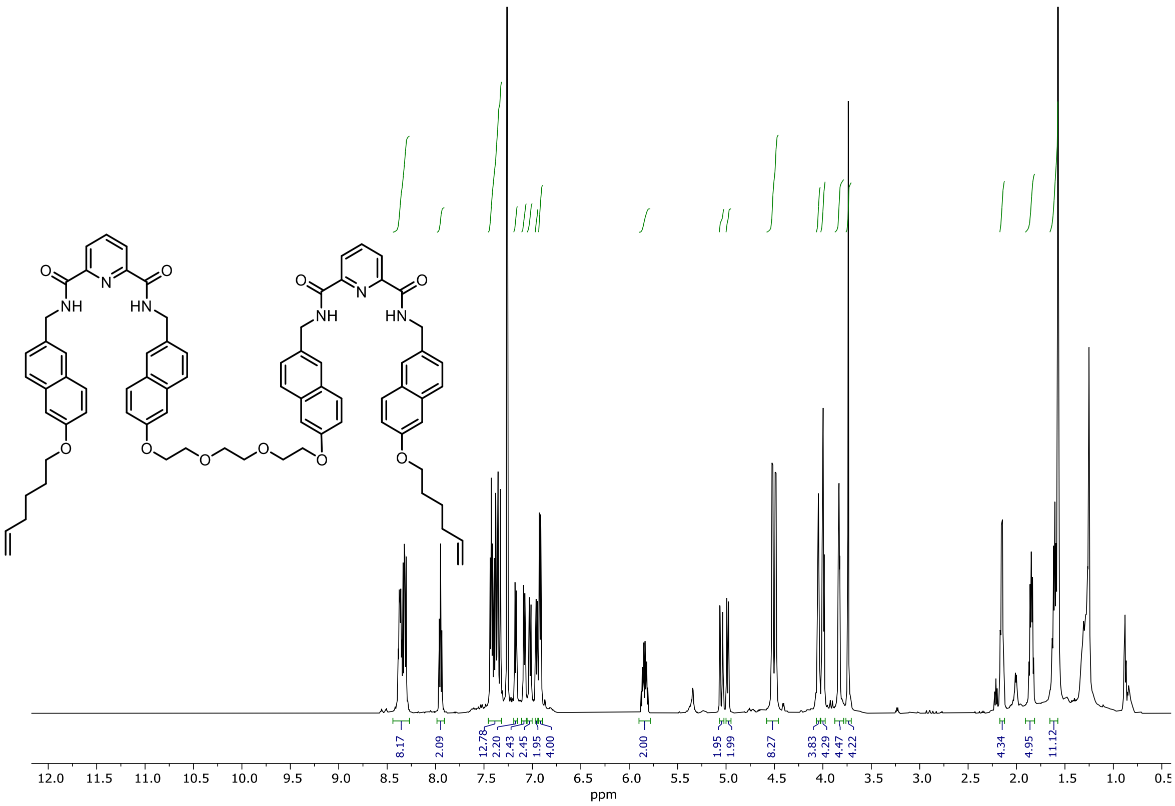

Figure S44. ^1H NMR (600 MHz, $CDCl_3$, 298 K) of **L2**.

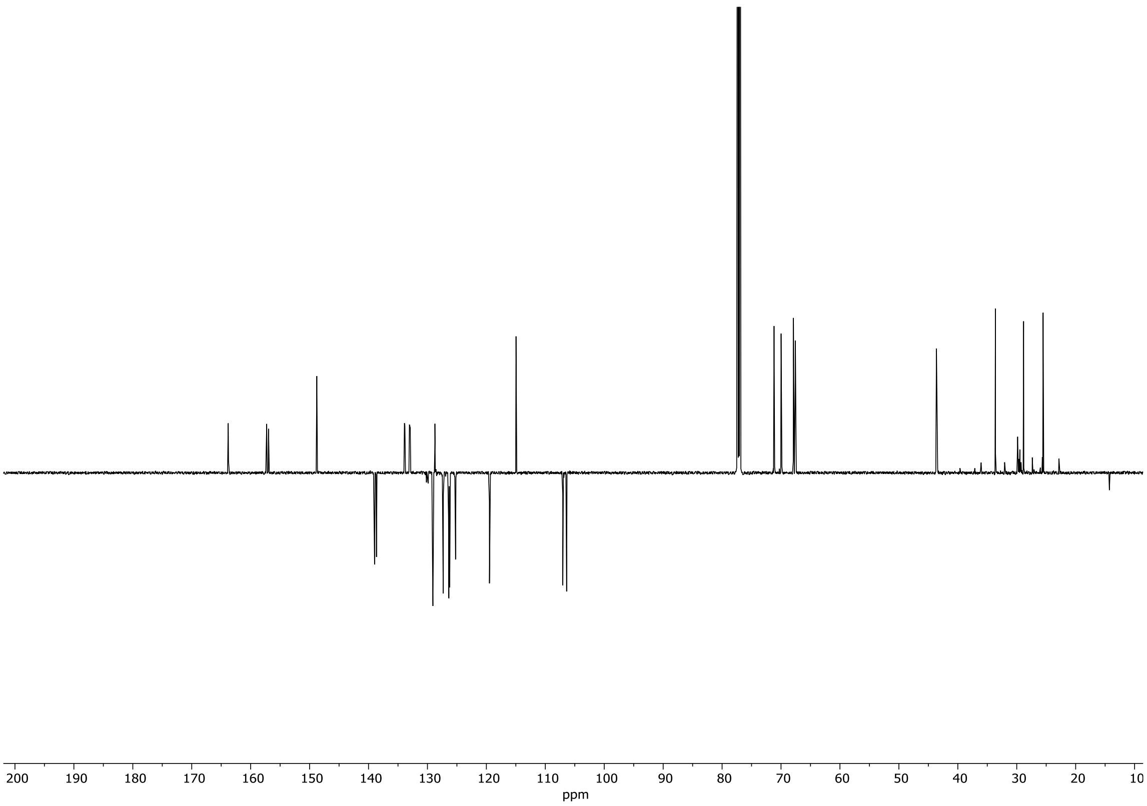

Figure S45. DEPTQ ^{13}C NMR (151 MHz, $CDCl_3$, 298 K) of **L2**.

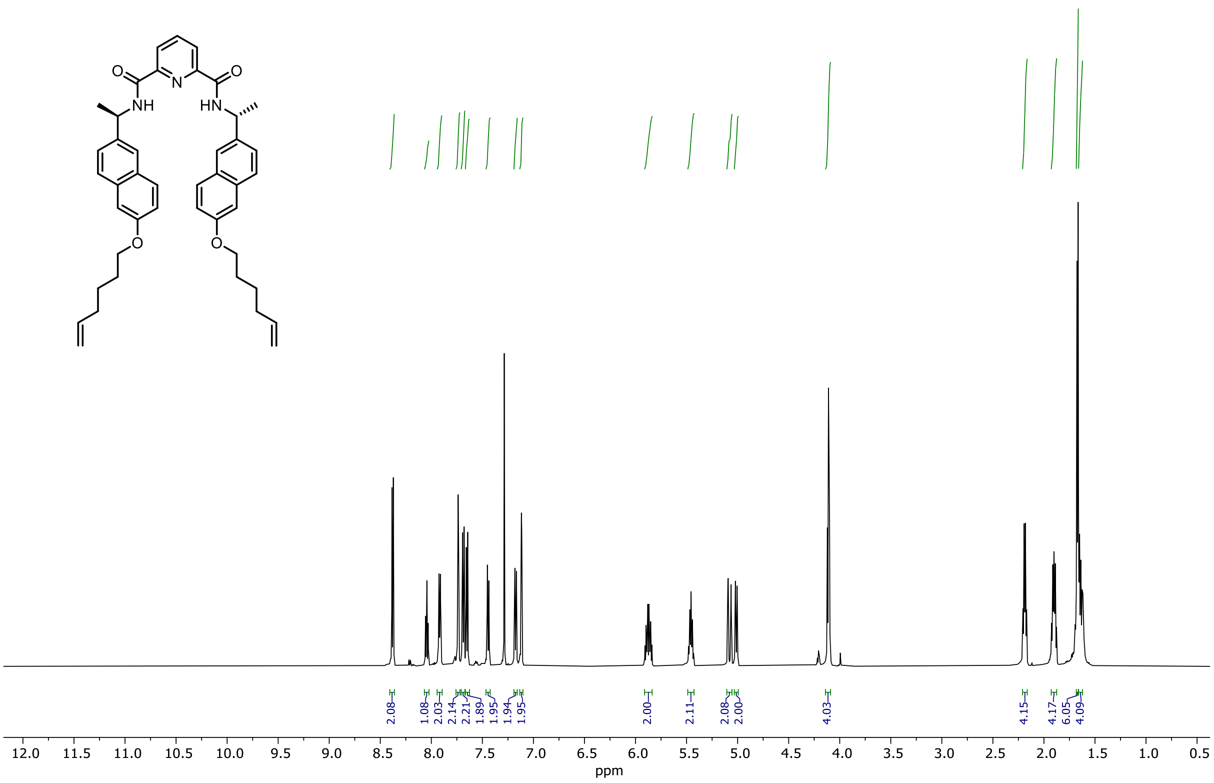

Figure S46. ^{1}H NMR (600 MHz, $CDCl_3$, 298 K) of (*R*)$_2$-**L3**.

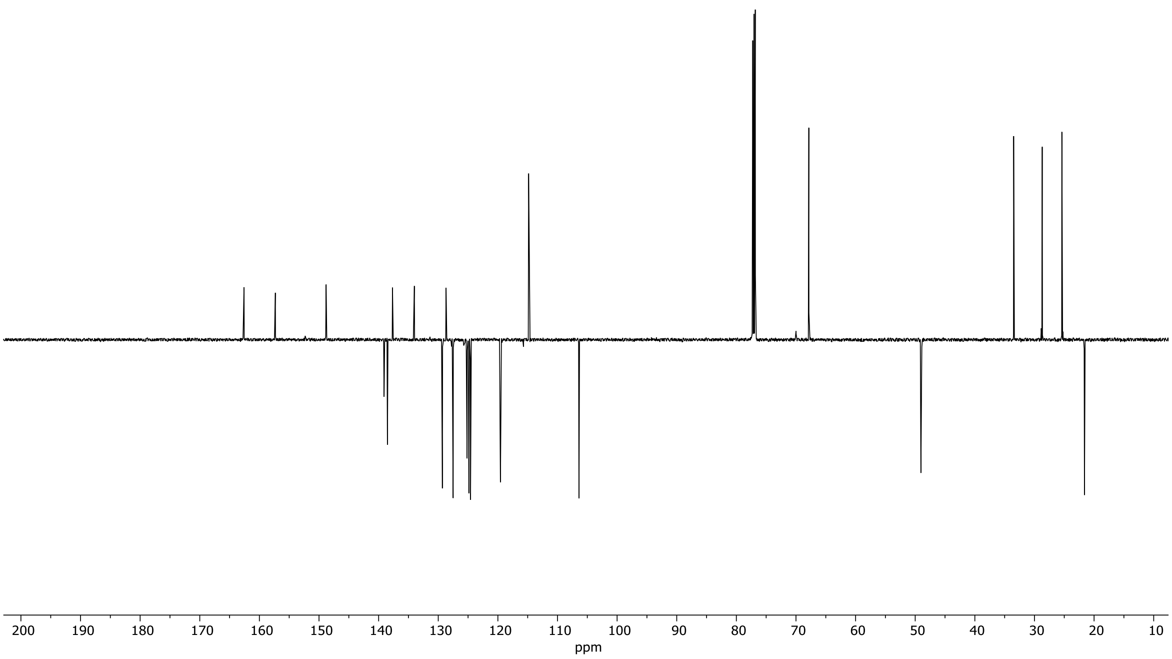

Figure S47. DEPTQ ^{13}C NMR (151 MHz, $CDCl_3$, 298 K) of (*R*)$_2$-**L3**.

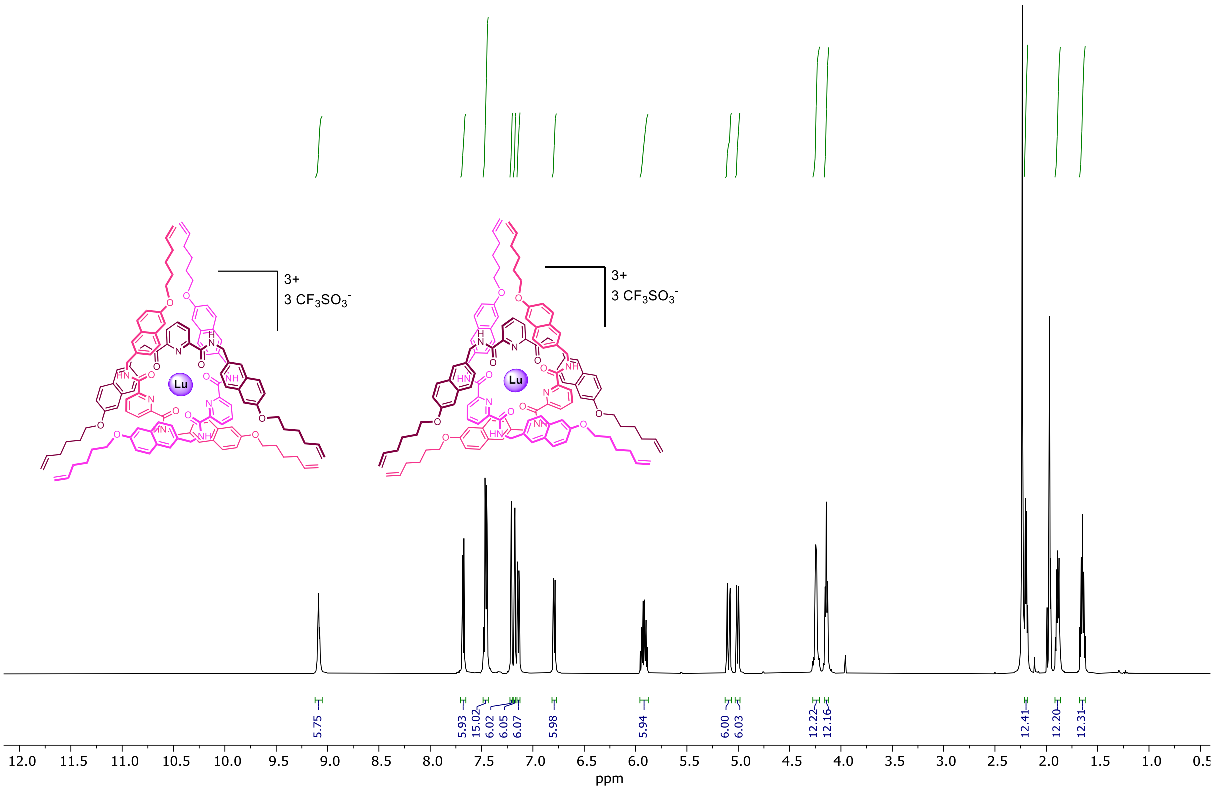

Figure S48. ^{1}H NMR (600 MHz, MeCN-d_3, 298 K) of helicate **L1**$_3$•[Lu].

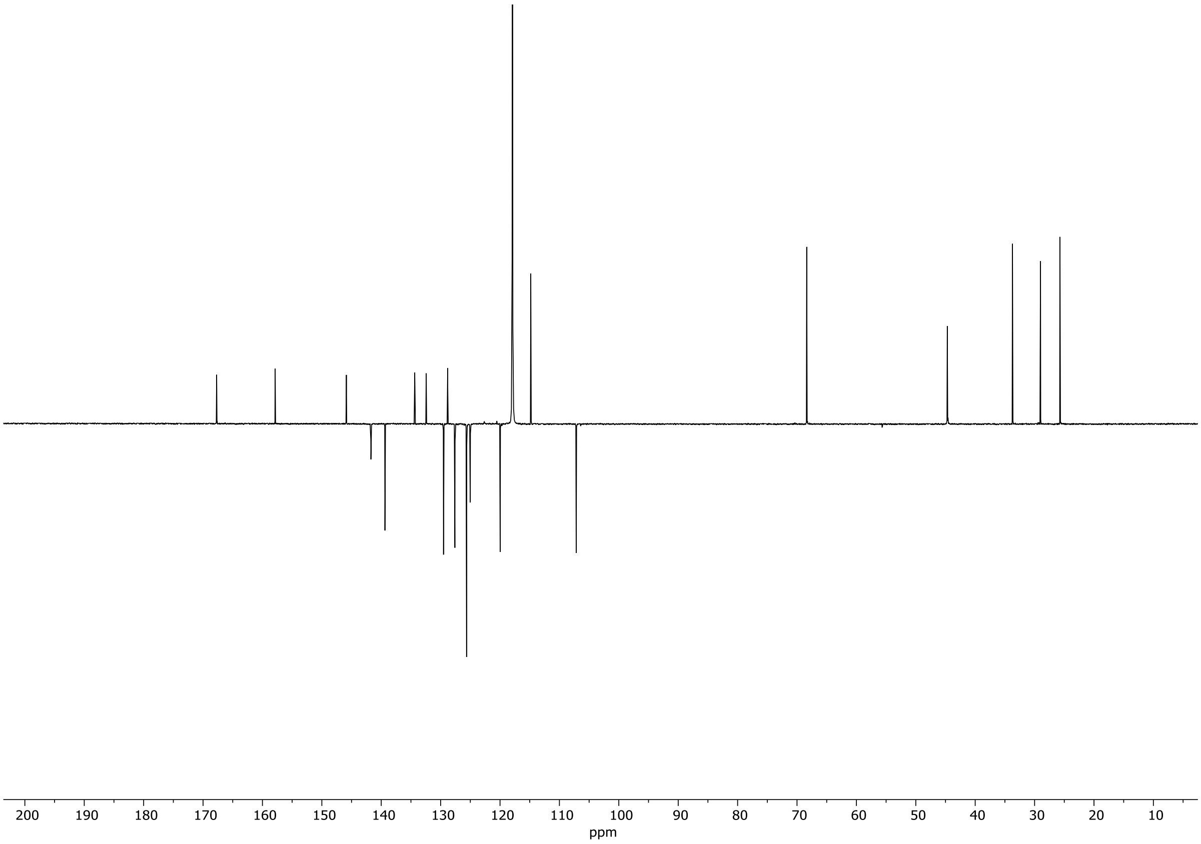

Figure S49. DEPTQ ^{13}C NMR (151 MHz, MeCN-d_3, 298 K) of helicate **L1**$_3$•[Lu].

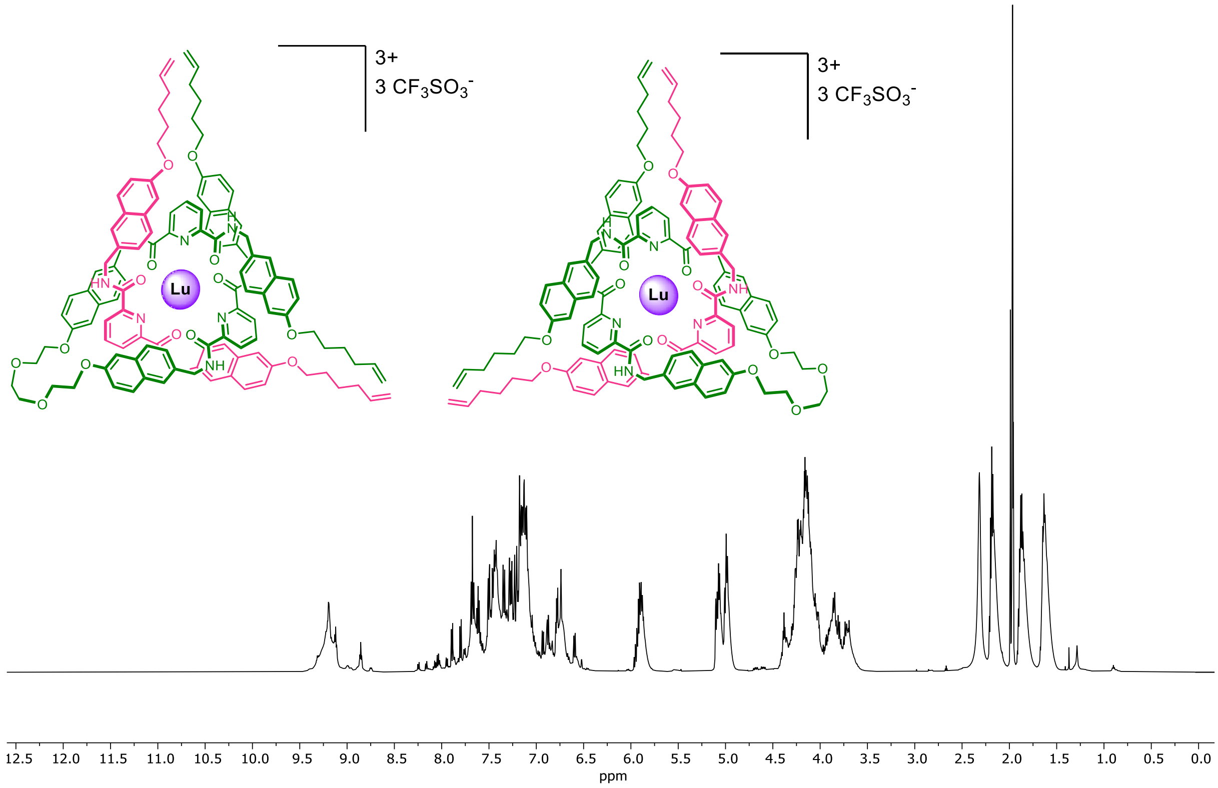

Figure S50. ^{1}H NMR (600 MHz, MeCN-d_3, 298 K) of helicate {**L1**,**L2**•[Lu]}.

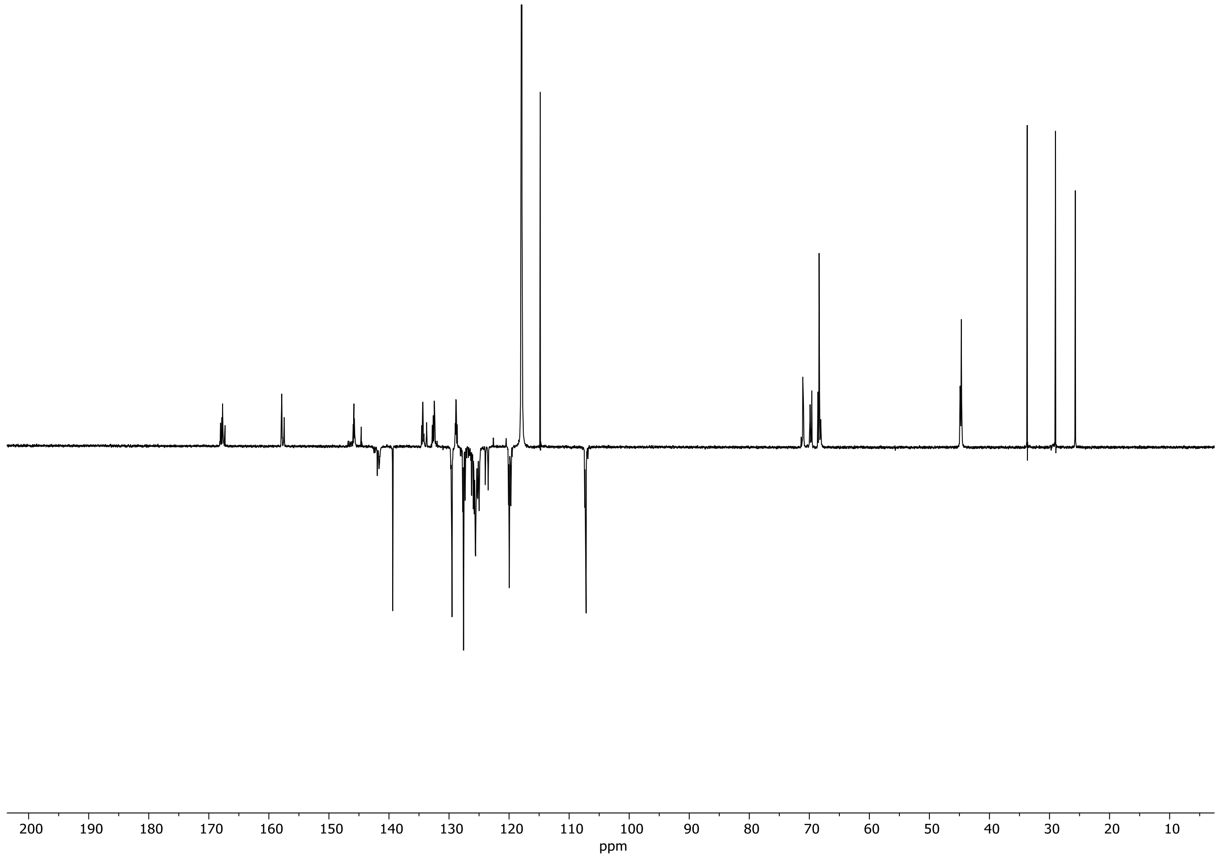

Figure S51. DEPTQ ^{13}C NMR (151 MHz, MeCN-d_3, 298 K) of helicate {**L1**,**L2**•[Lu]}.

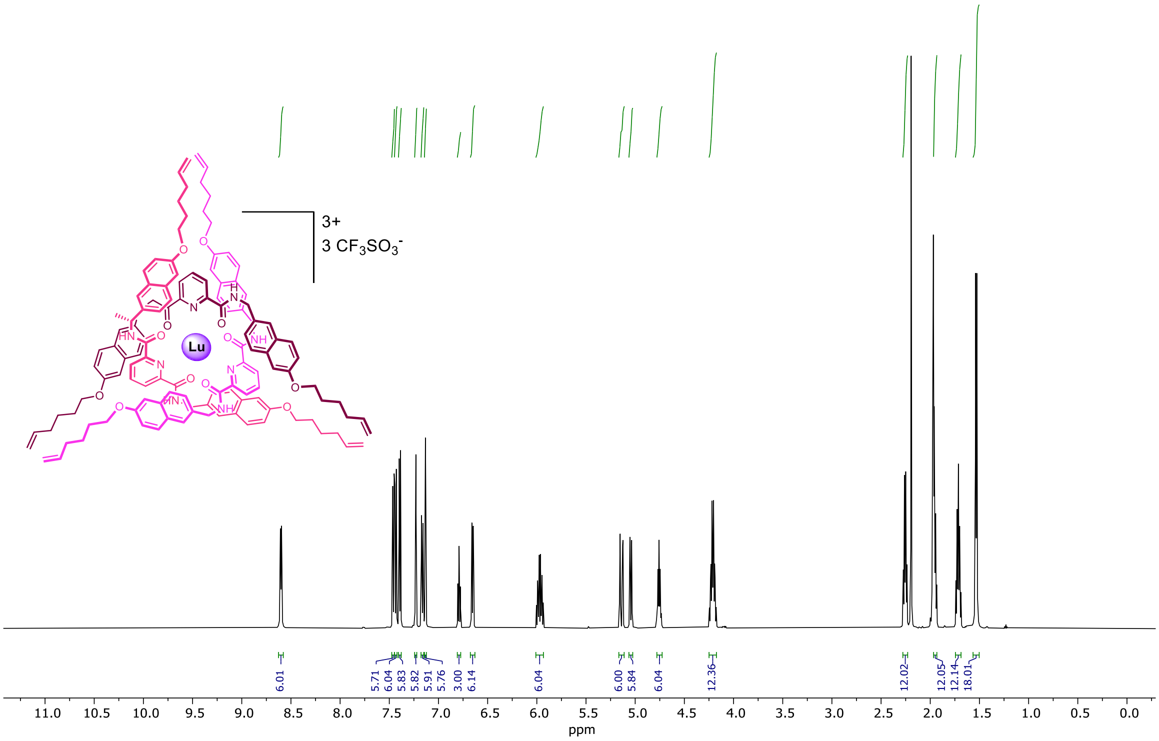

Figure S52. ^{1}H NMR (600 MHz, MeCN-d_3, 298 K) of helicate Λ-((R)$_2$-**L3**)$_3$•[Lu].

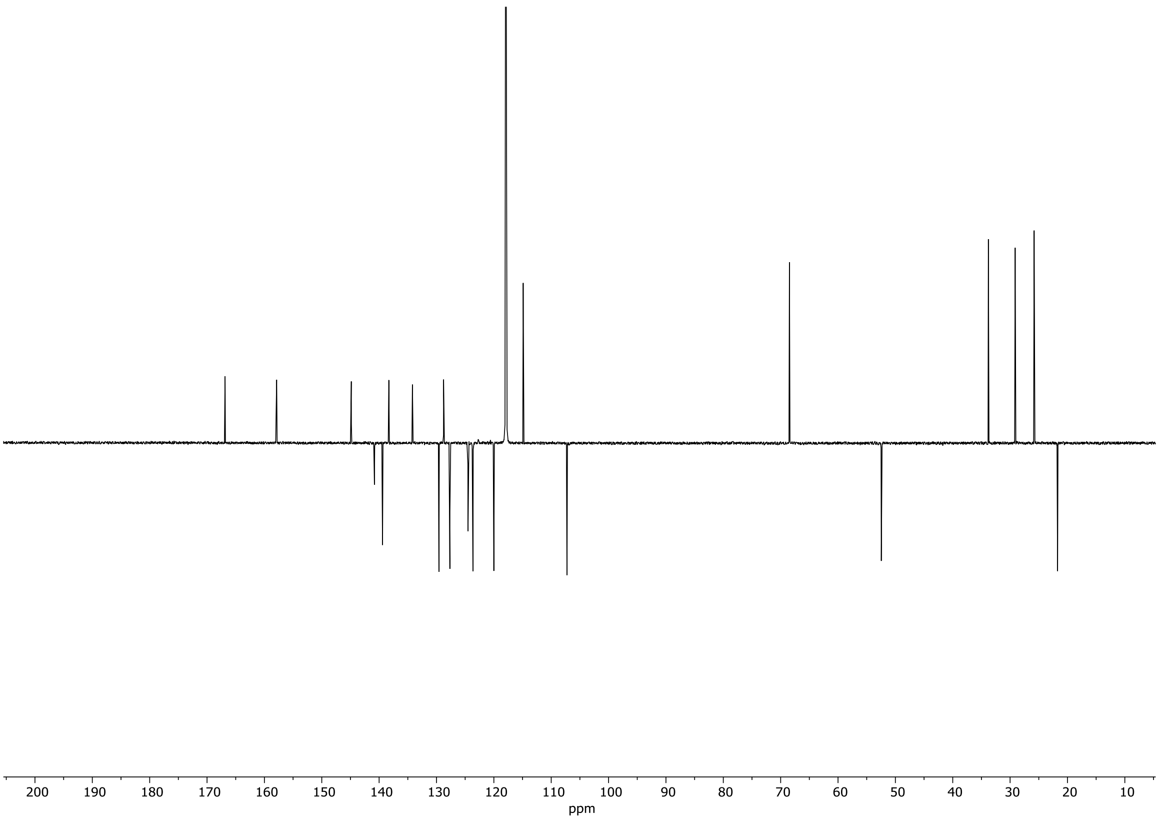

Figure S53. DEPTQ ^{13}C NMR (151 MHz, MeCN-d_3, 298 K) of helicate Λ-((R)$_2$-**L3**)$_3$•[Lu].

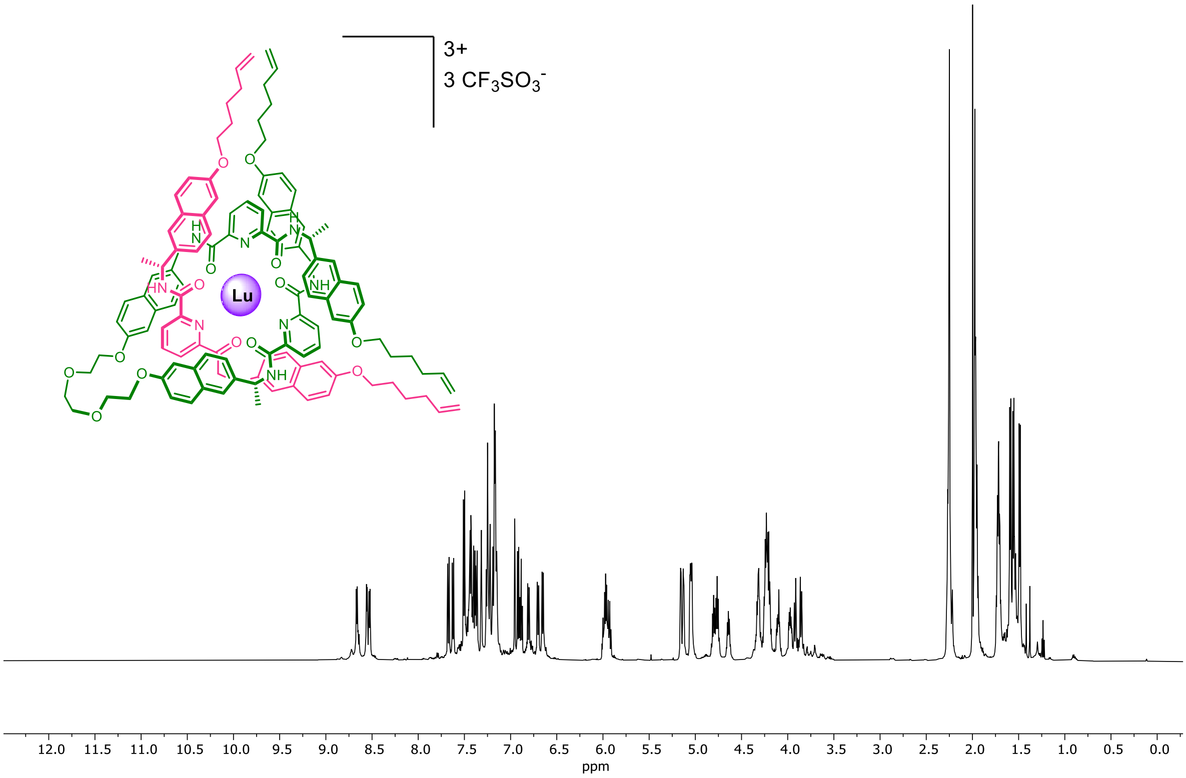

Figure S54. ^{1}H NMR (600 MHz, MeCN-d_3, 298 K) of helicate Λ-{(R)$_2$-**L3**,(R)$_4$-**L4**•[Lu]}.

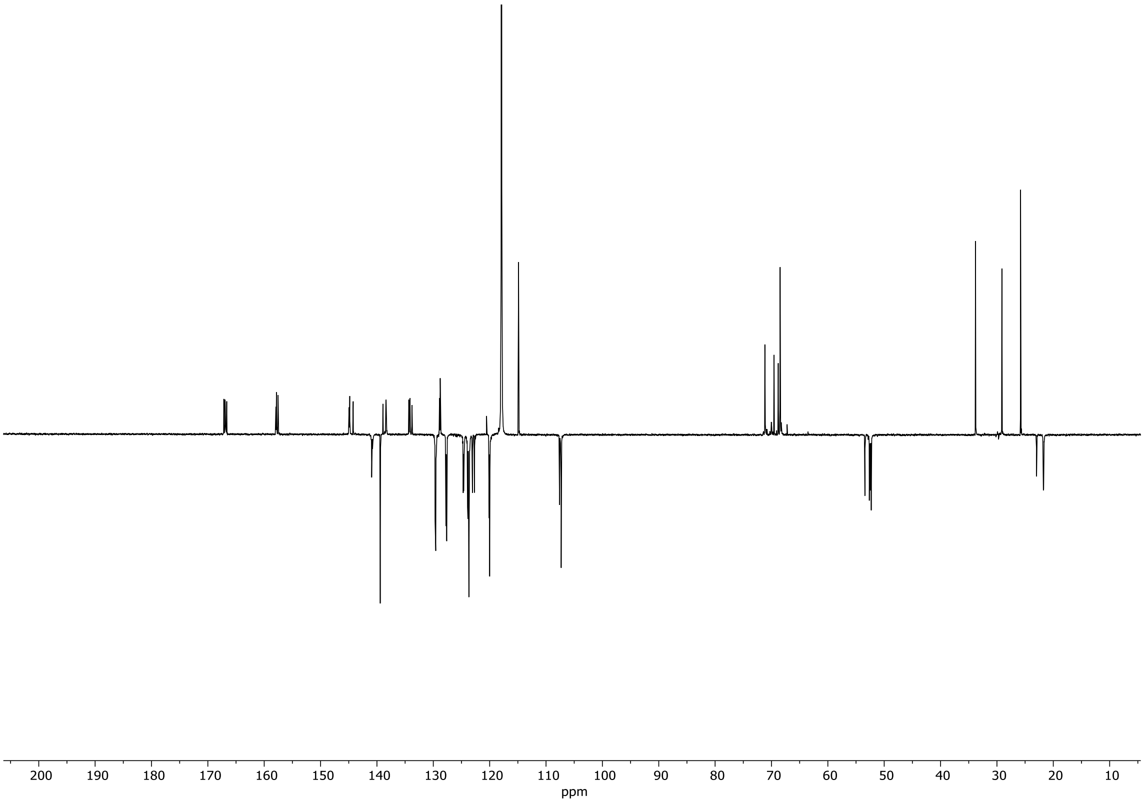

Figure S55. DEPTQ ^{13}C NMR (151 MHz, MeCN-d_3, 298 K) of helicate Λ-{(R)$_2$-**L3**,(R)$_4$-**L4**•[Lu]}.

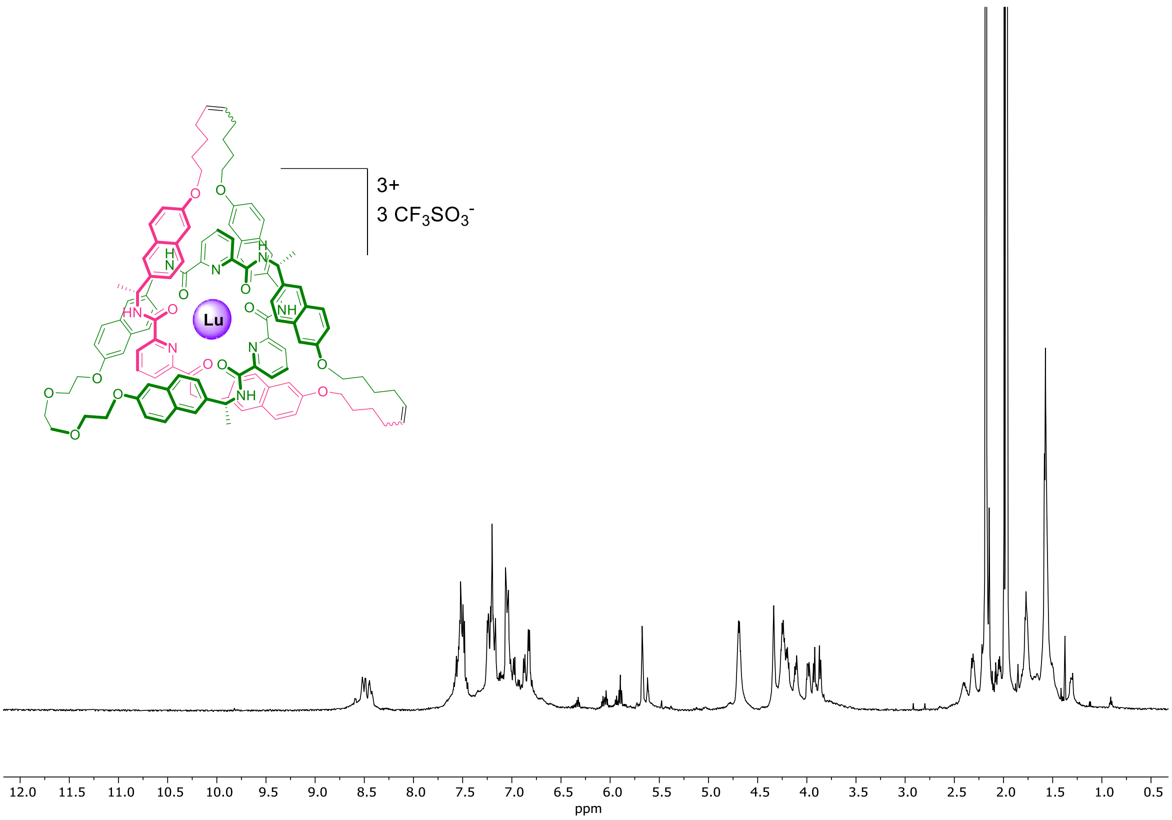

Figure S56. ^{1}H NMR (600 MHz, 298 K, MeCN-d_3, 298 K) of trefoil knot Λ-**1**•[Lu].

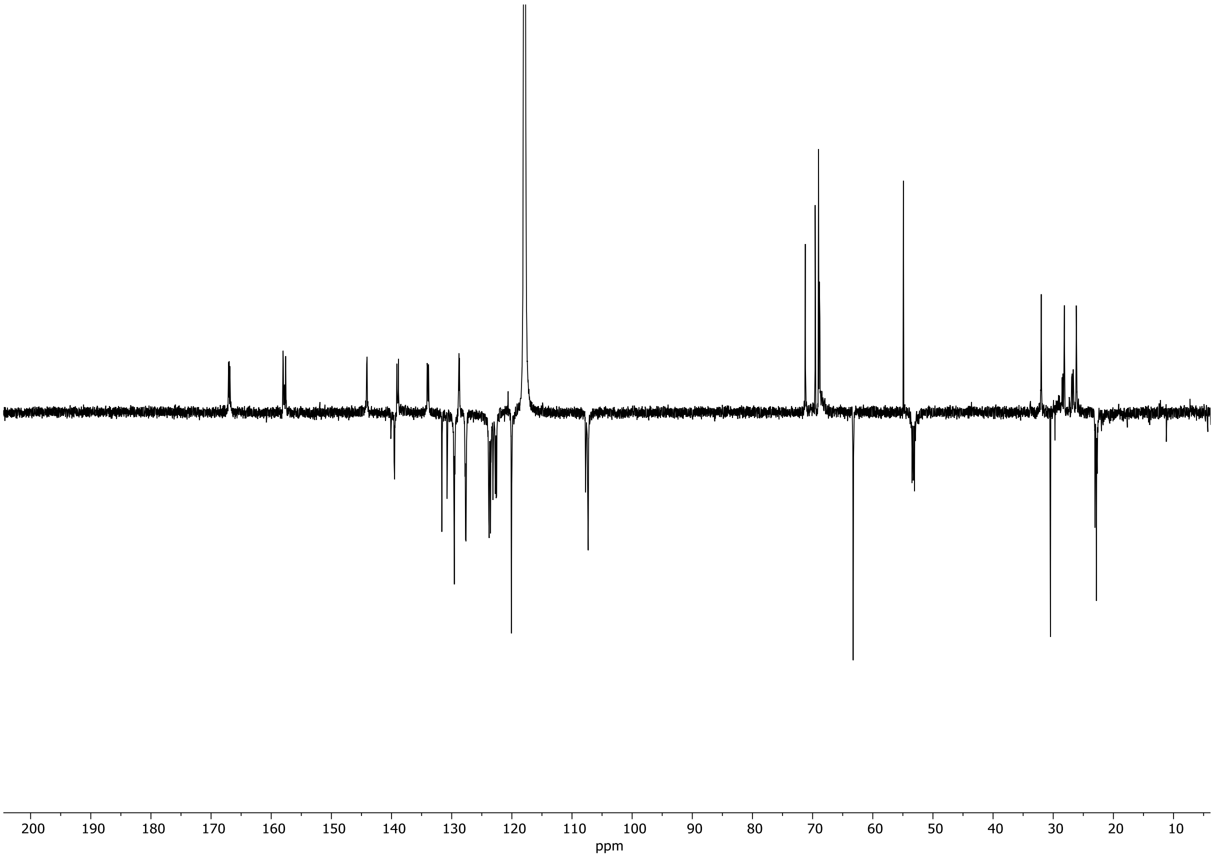

Figure S57. DEPTQ ^{13}C NMR (151 MHz, MeCN-d_3, 298 K) of trefoil knot Λ-**1**•[Lu].

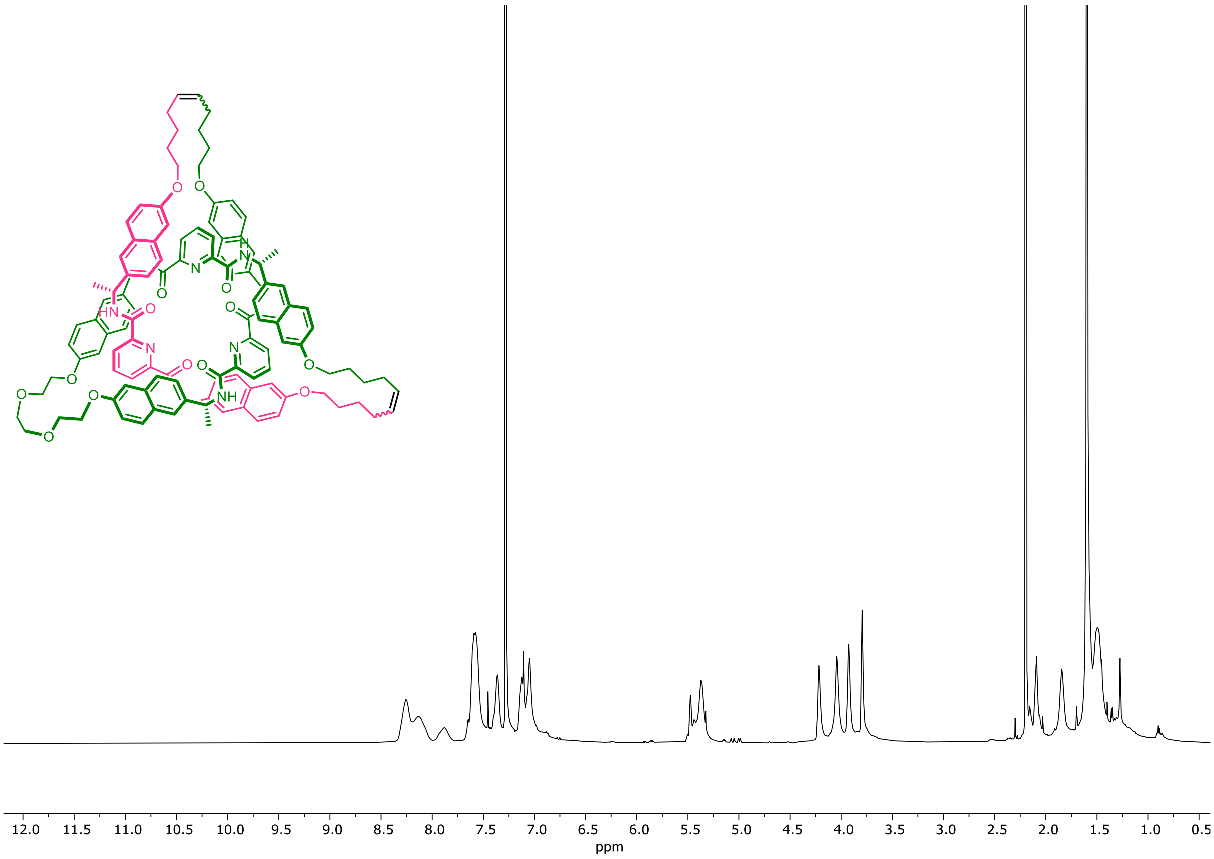

Figure S58. ^{1}H NMR (600 MHz, $CDCl_3$, 298 K) of trefoil knot Λ-**1**.

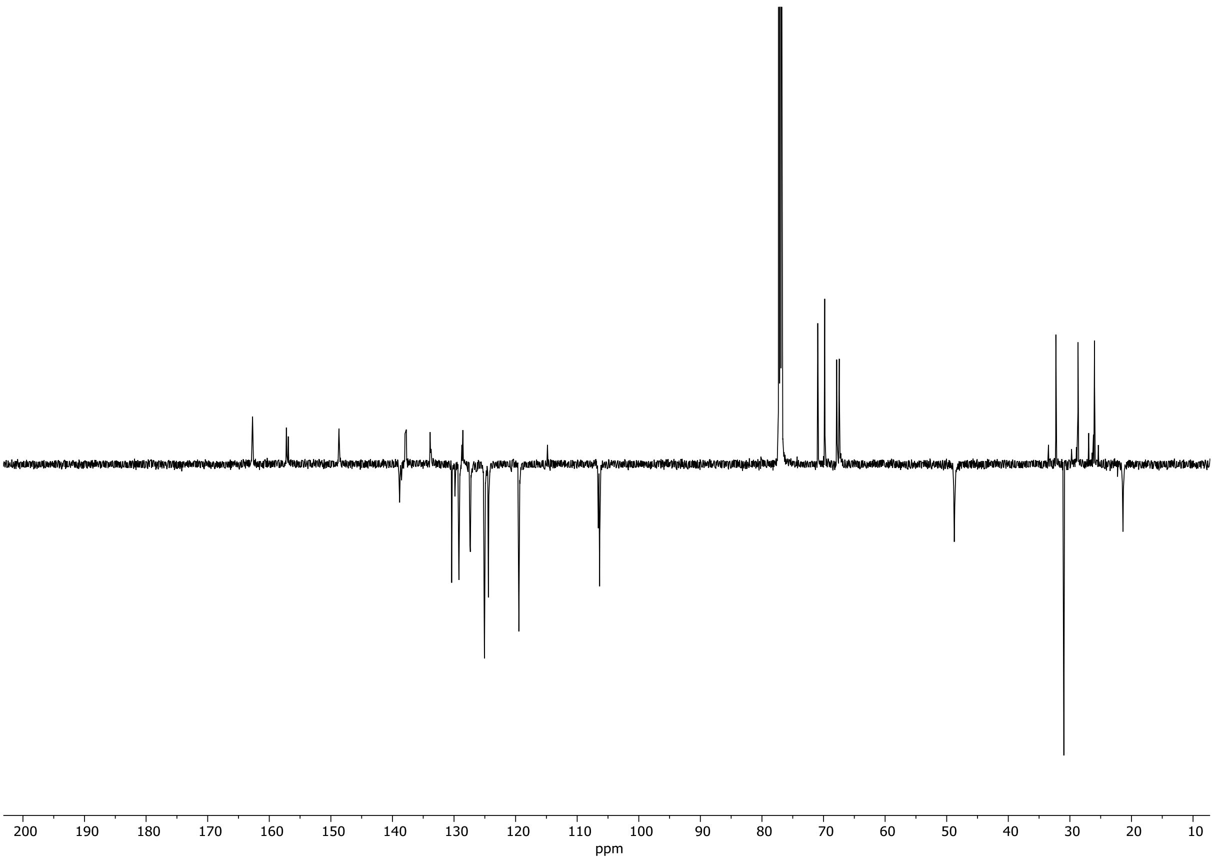

Figure S59. DEPTQ ^{13}C NMR (151 MHz, $CDCl_3$, 298 K) of trefoil knot Λ-**1**.

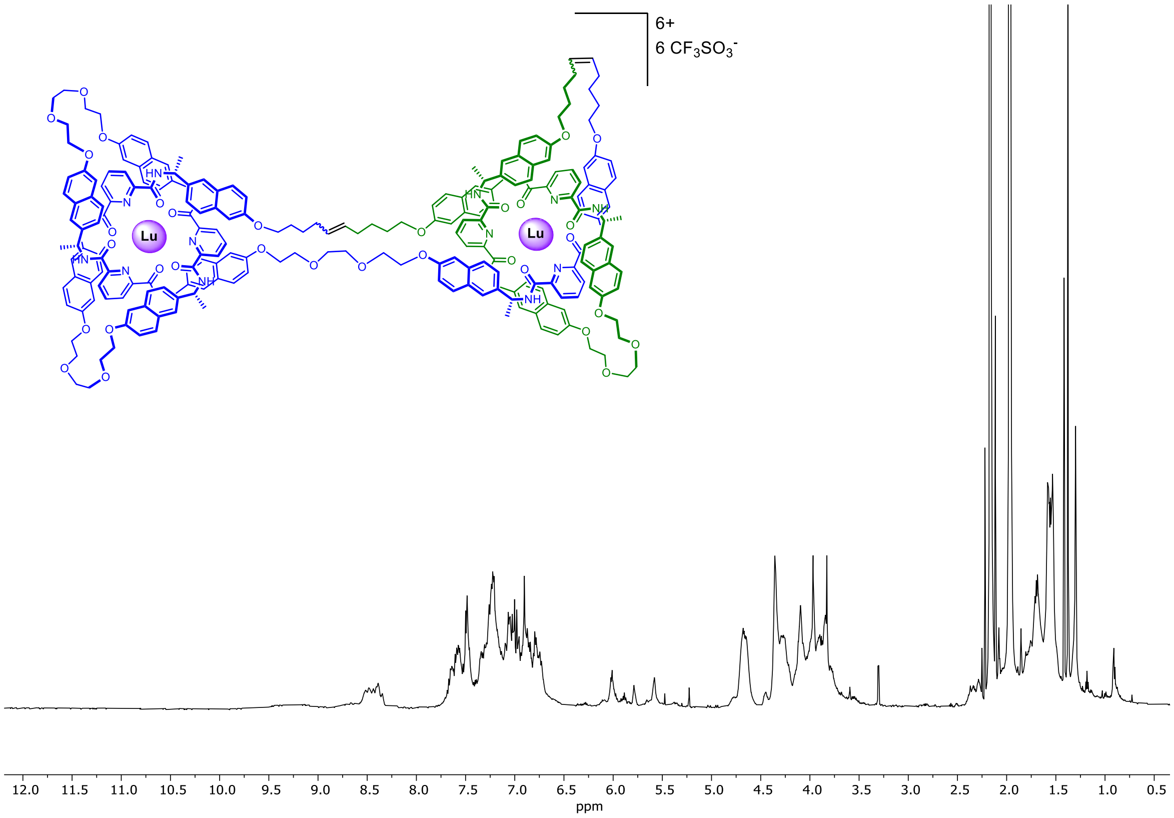

Figure S60. ^{1}H NMR (600 MHz, MeCN-d_3, 298 K) of granny knot (Λ,Λ)-**2**•[Lu]$_2$.

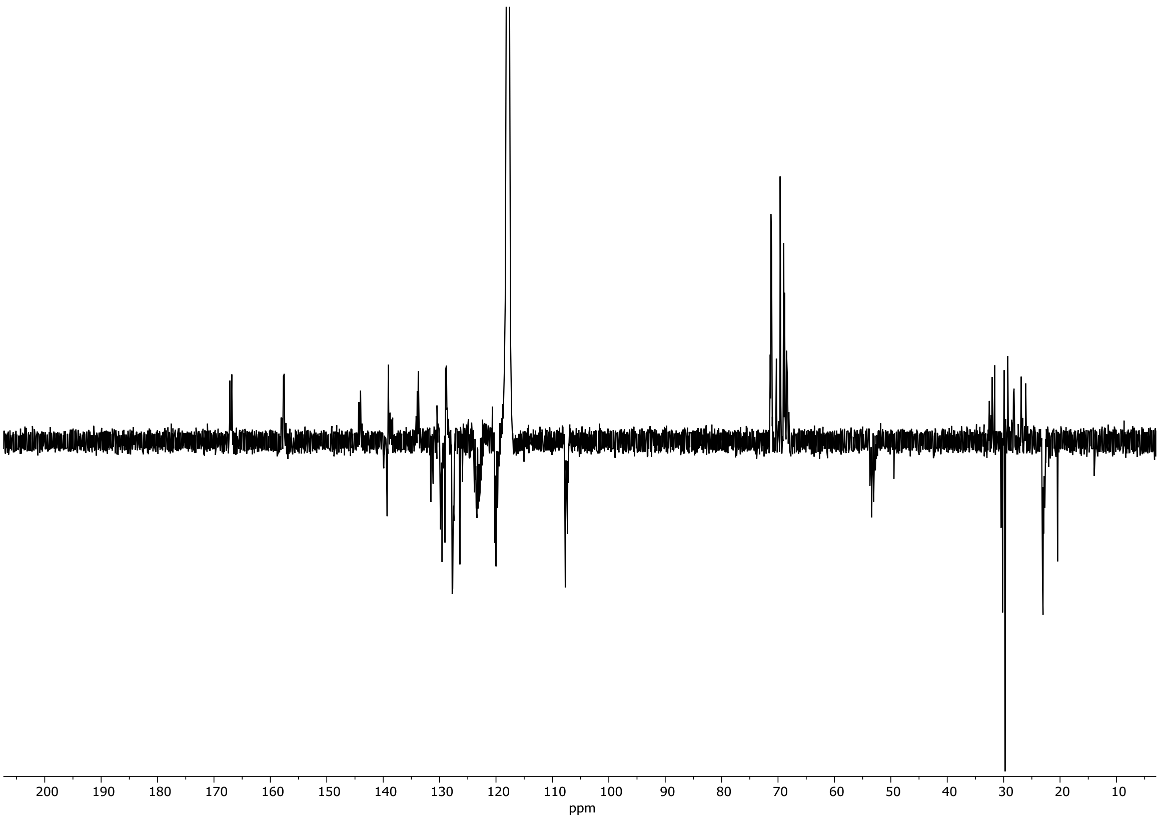

Figure S61. DEPTQ ^{13}C NMR (151 MHz, MeCN-d_3, 298 K) of granny knot (Λ,Λ)-**2**•[Lu]$_2$.

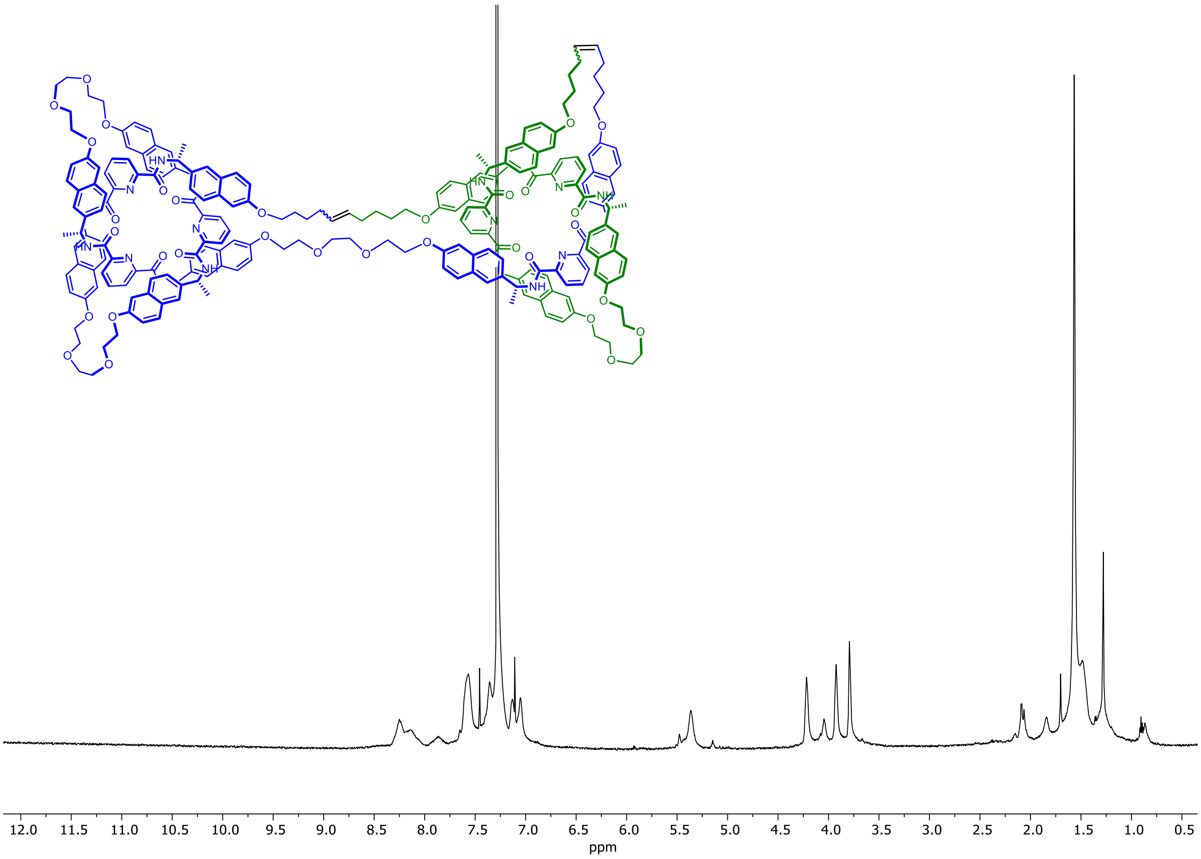

Figure S62. ^{1}H NMR (600 MHz, $CDCl_3$, 298 K) of granny knot (Λ,Λ)-**2**.

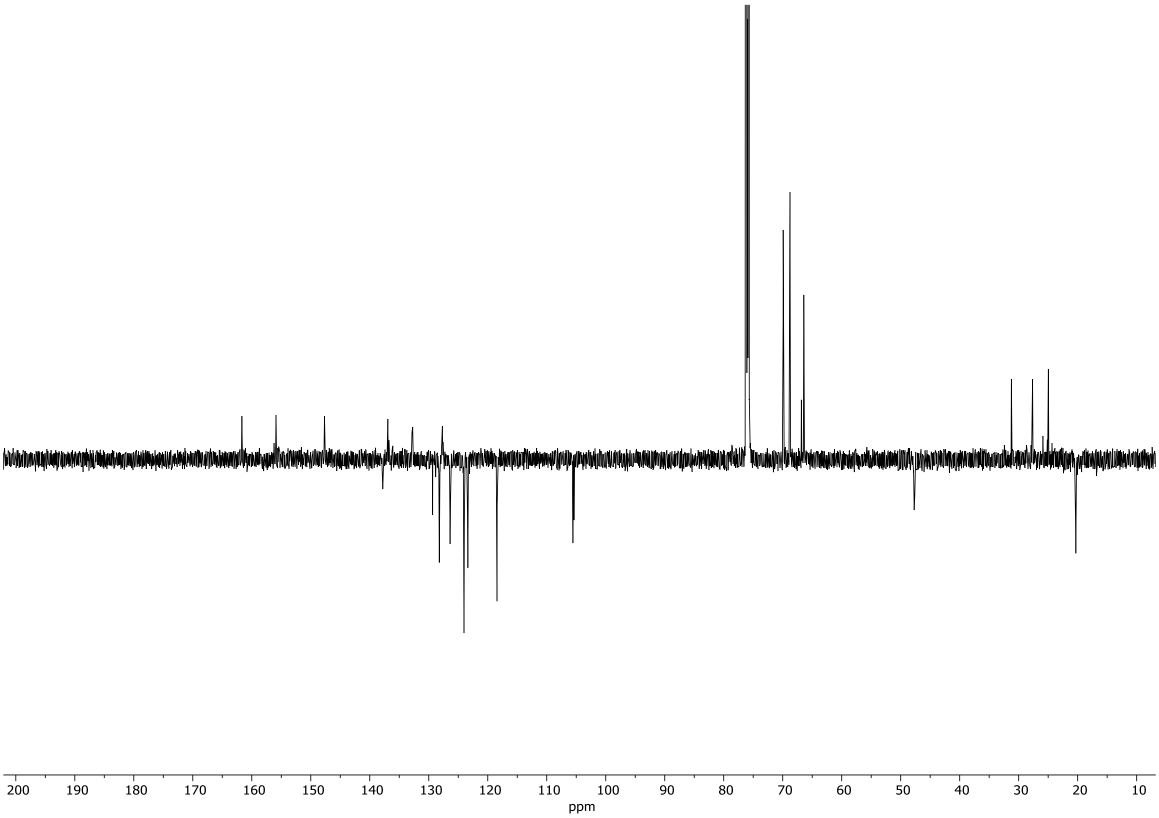

Figure S63. DEPTQ ^{13}C NMR (151 MHz, $CDCl_3$, 298 K) of granny knot (Λ,Λ)-**2**.

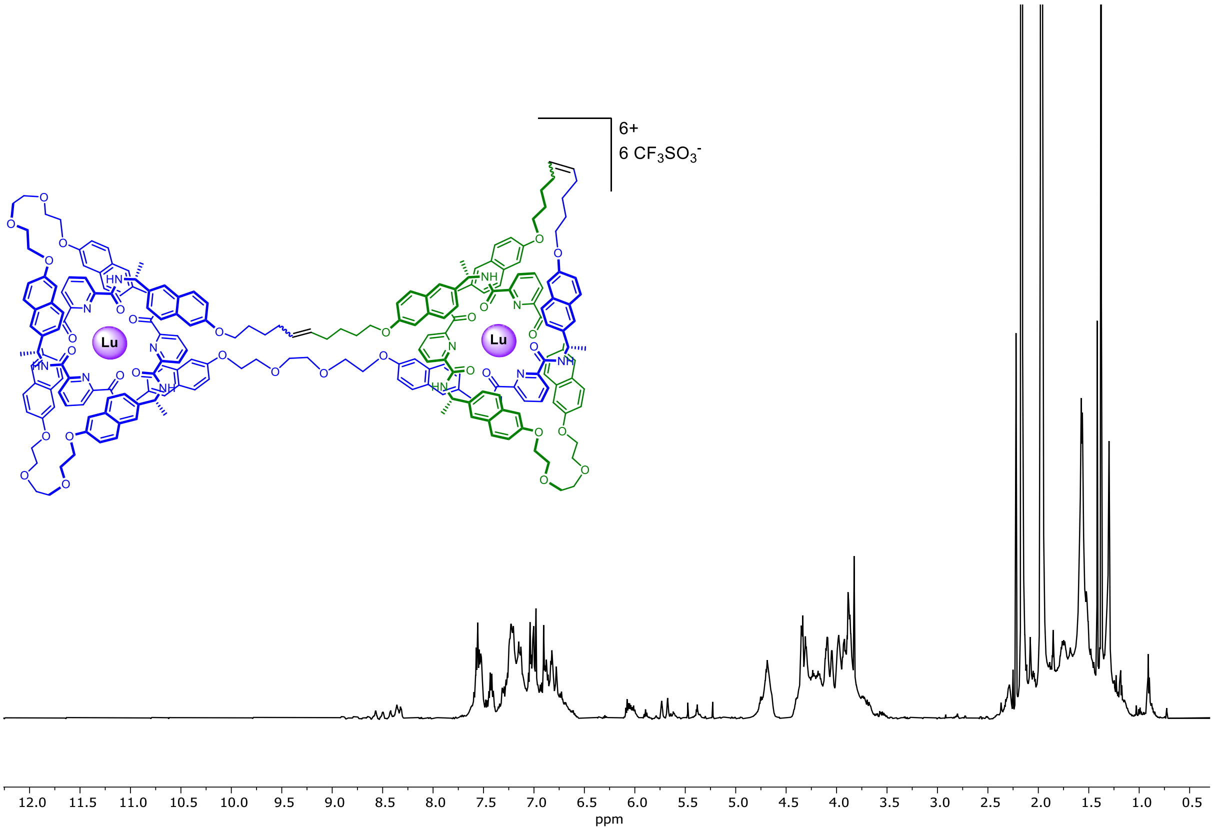

Figure S64. ^{1}H NMR (600 MHz, MeCN-d_3, 298 K) of square knot (Λ,Δ)-**2**•[Lu]$_2$.

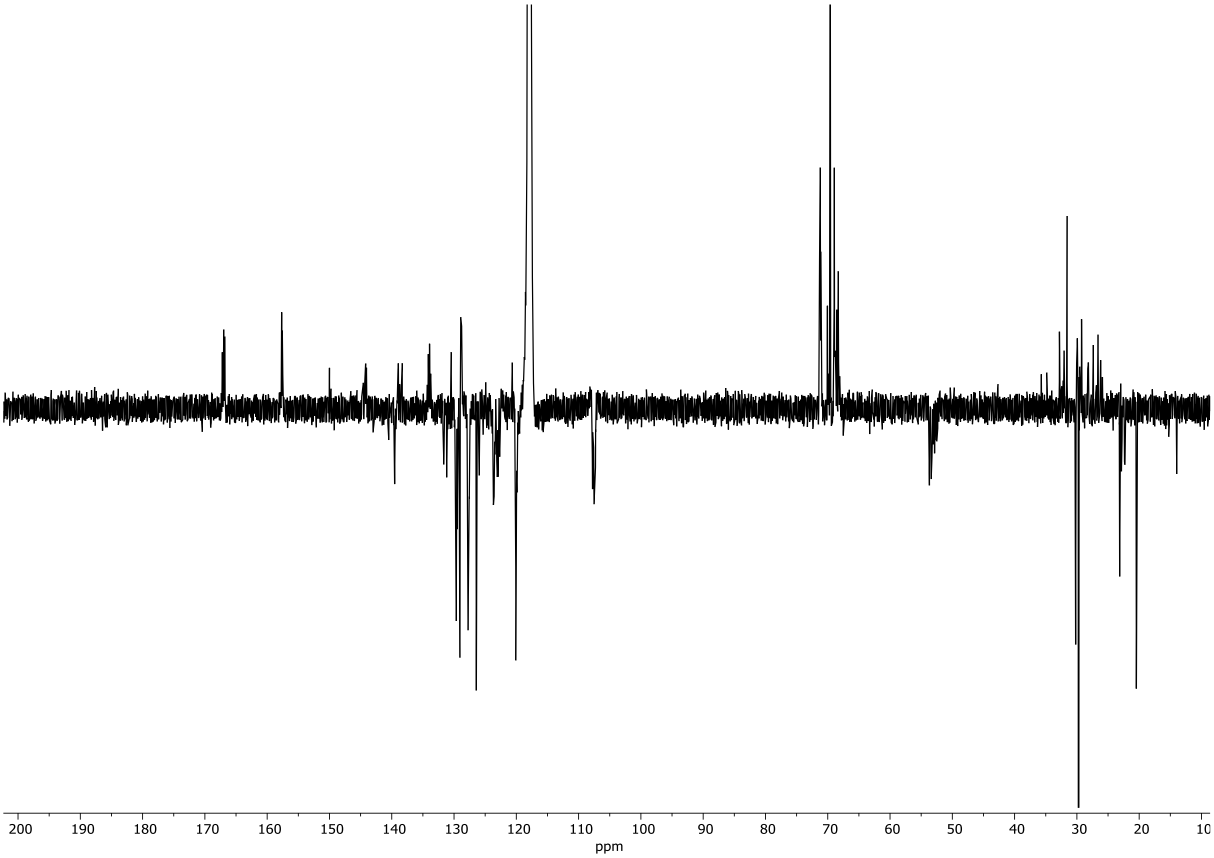

Figure S65. DEPTQ ^{13}C NMR (151 MHz, MeCN-d_3, 298 K) of square knot (Λ,Δ)-**2**•[Lu]$_2$.

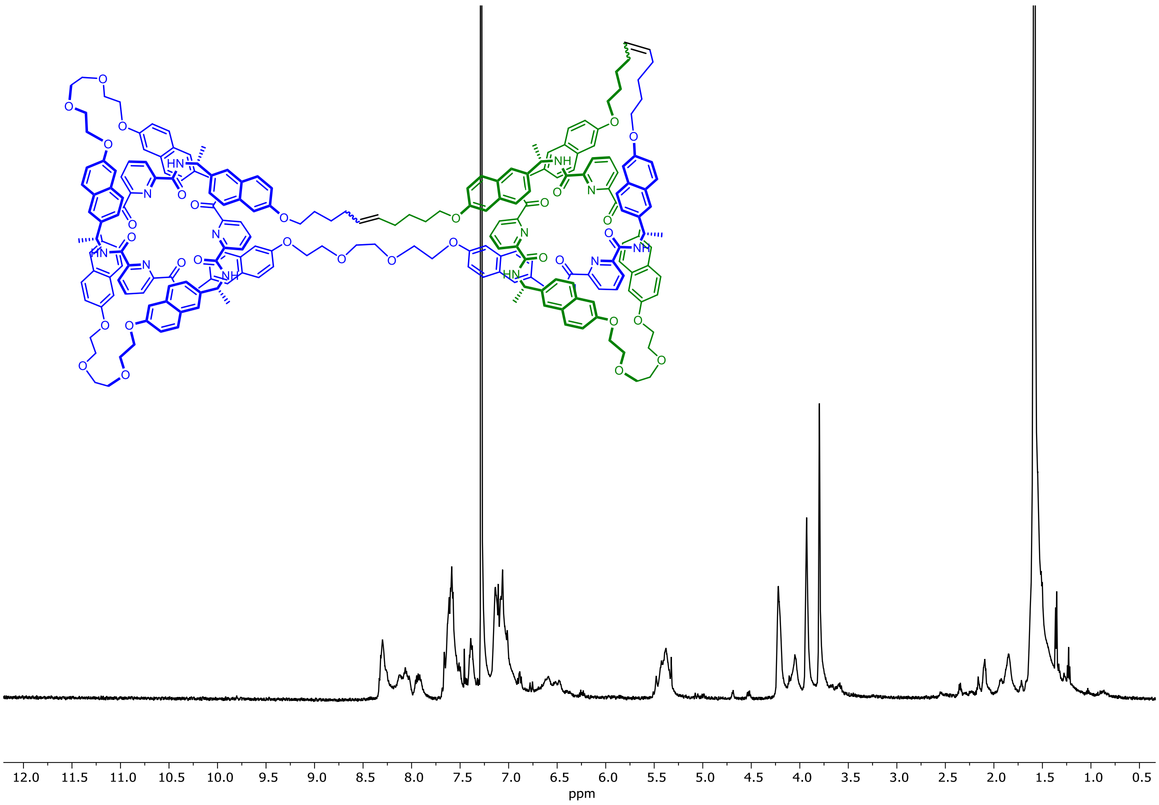

Figure S66. ^{1}H NMR (600 MHz, $CDCl_3$, 298 K) of square knot (Λ,Δ)-**2**.

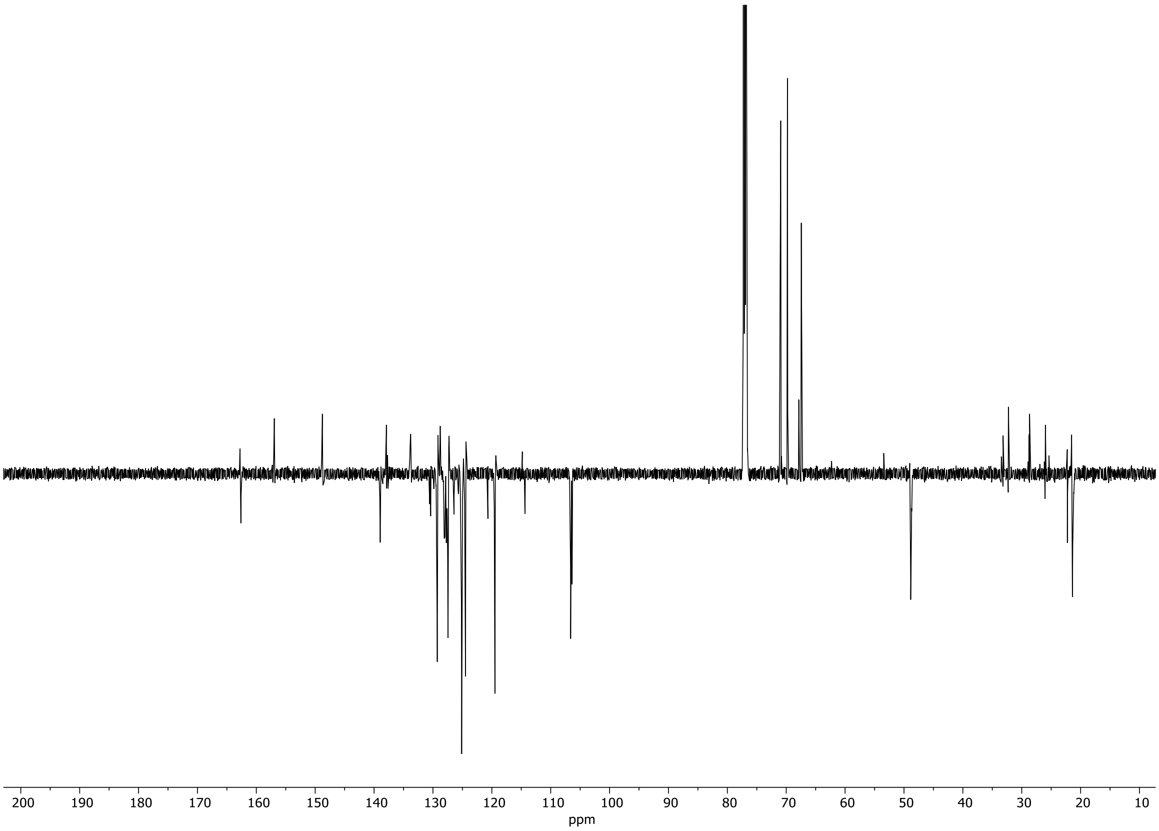

Figure S67. DEPTQ ^{13}C NMR (151 MHz, $CDCl_3$, 298 K) of square knot (Λ,Δ)-**2**.

S5.2 DOSY NMR Spectra

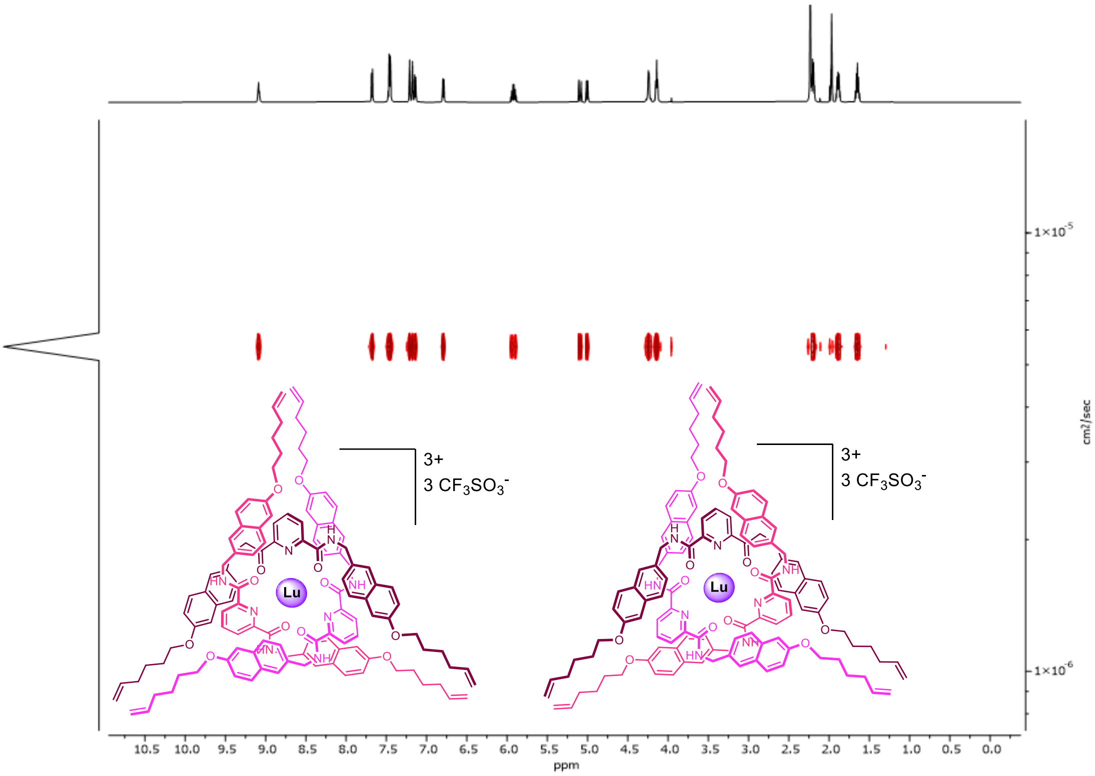

Figure S68. DOSY NMR (600 MHz, MeCN-d_3, 298 K) of **L1**$_3$•[Lu].

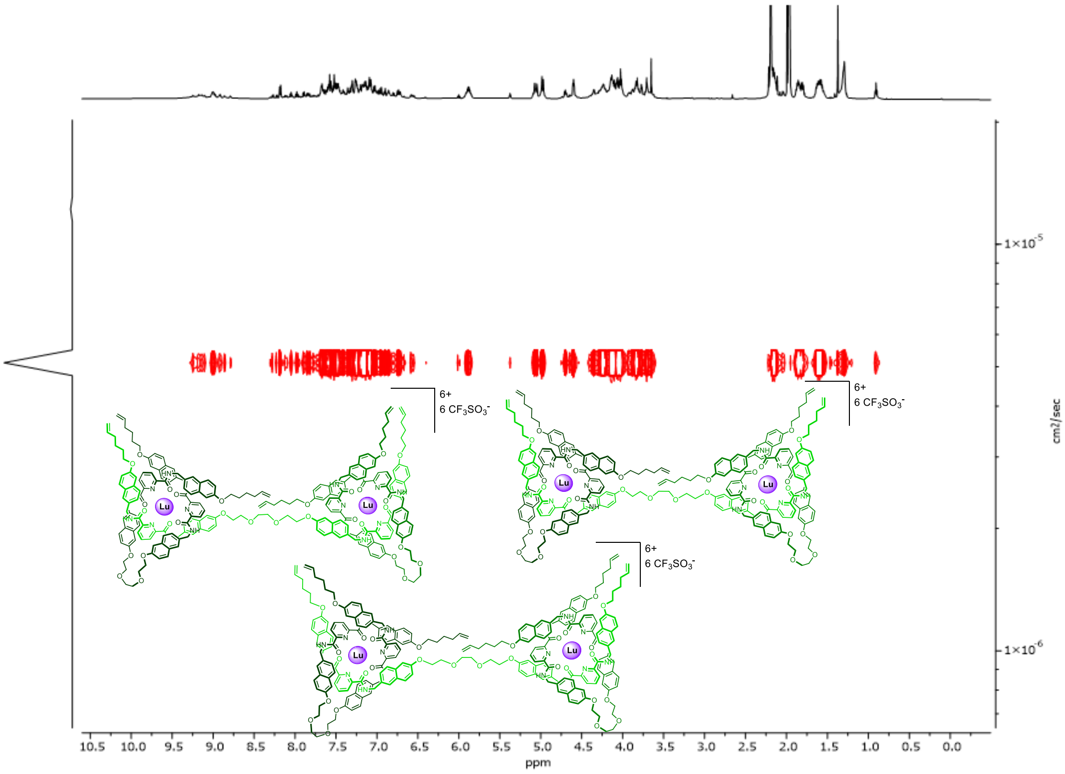

Figure S69. DOSY NMR (600 MHz, MeCN-d_3, 298 K) of **L2**$_3$•[Lu]$_2$.

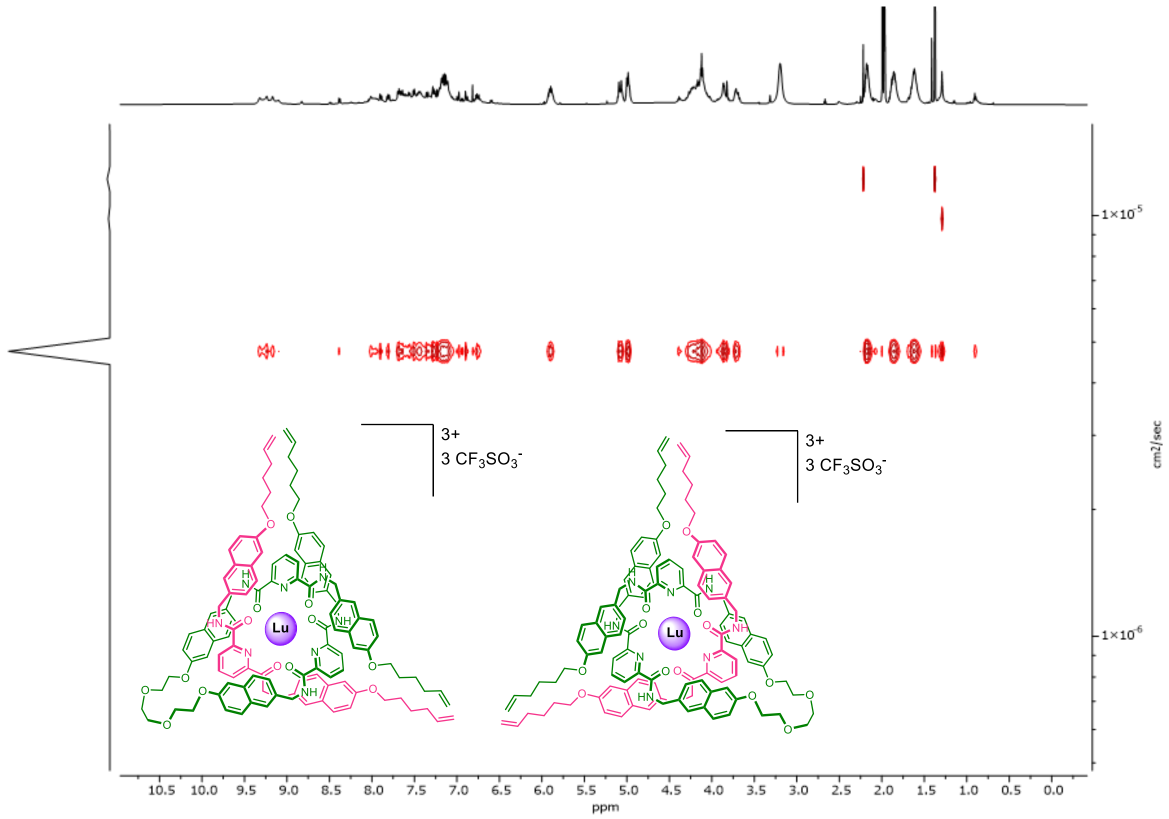

Figure S70. DOSY NMR (600 MHz, MeCN-d_3, 298 K) of {**L1**,**L2**•[Lu]}.

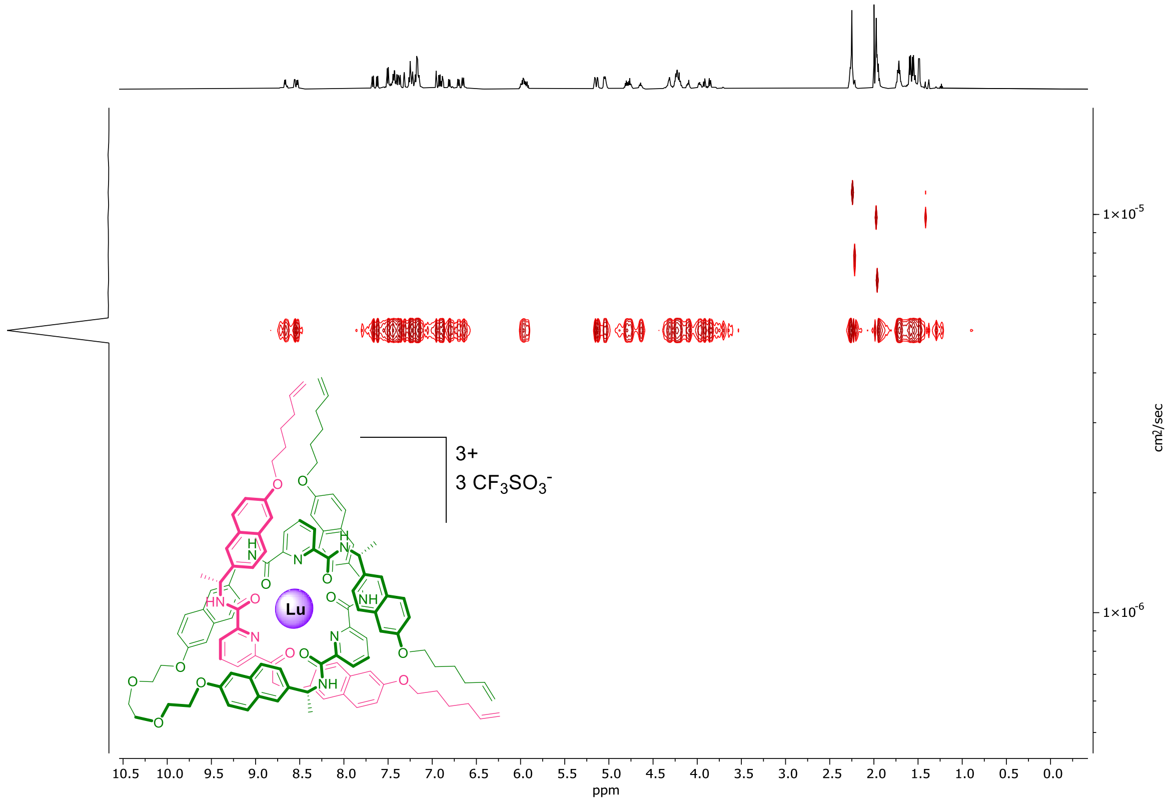

Figure S71. DOSY NMR (600 MHz, MeCN-d_3, 298 K) of Λ-{(R)$_2$-**L3**,(R)$_4$-**L4**•[Lu]}.

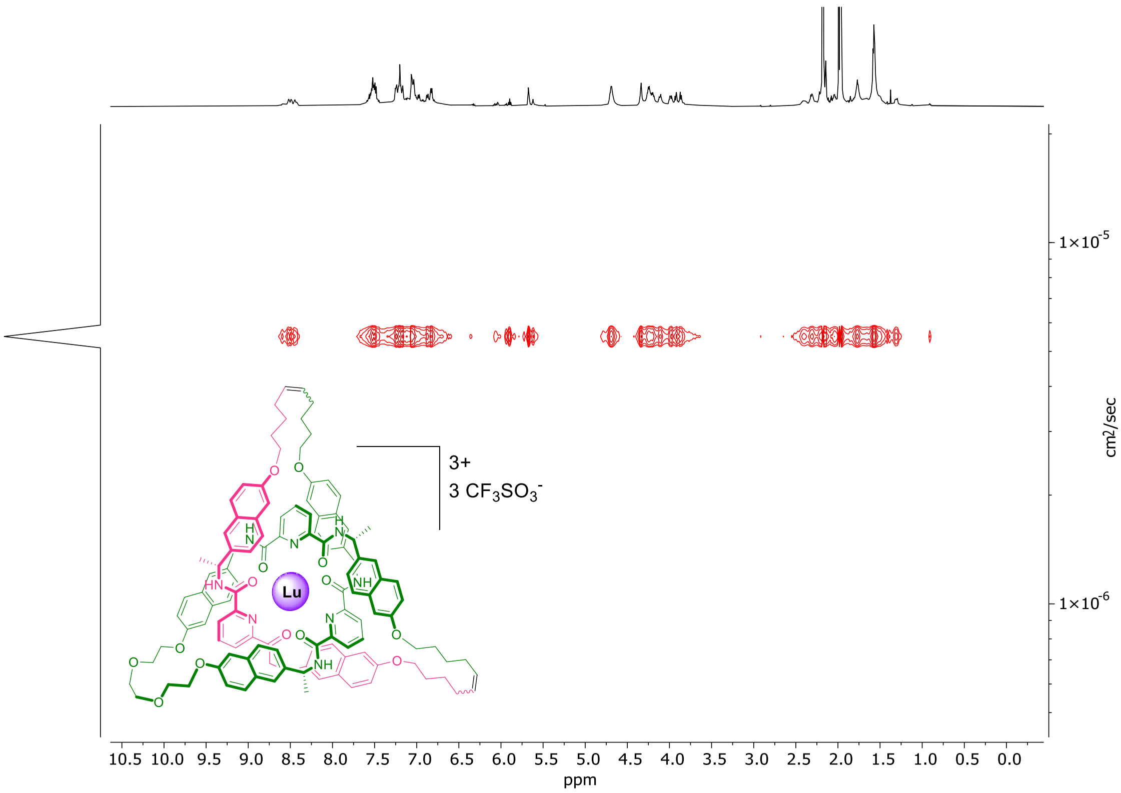

Figure S72. DOSY NMR (600 MHz, MeCN-d_3, 298 K) of Λ-**1**•[Lu].

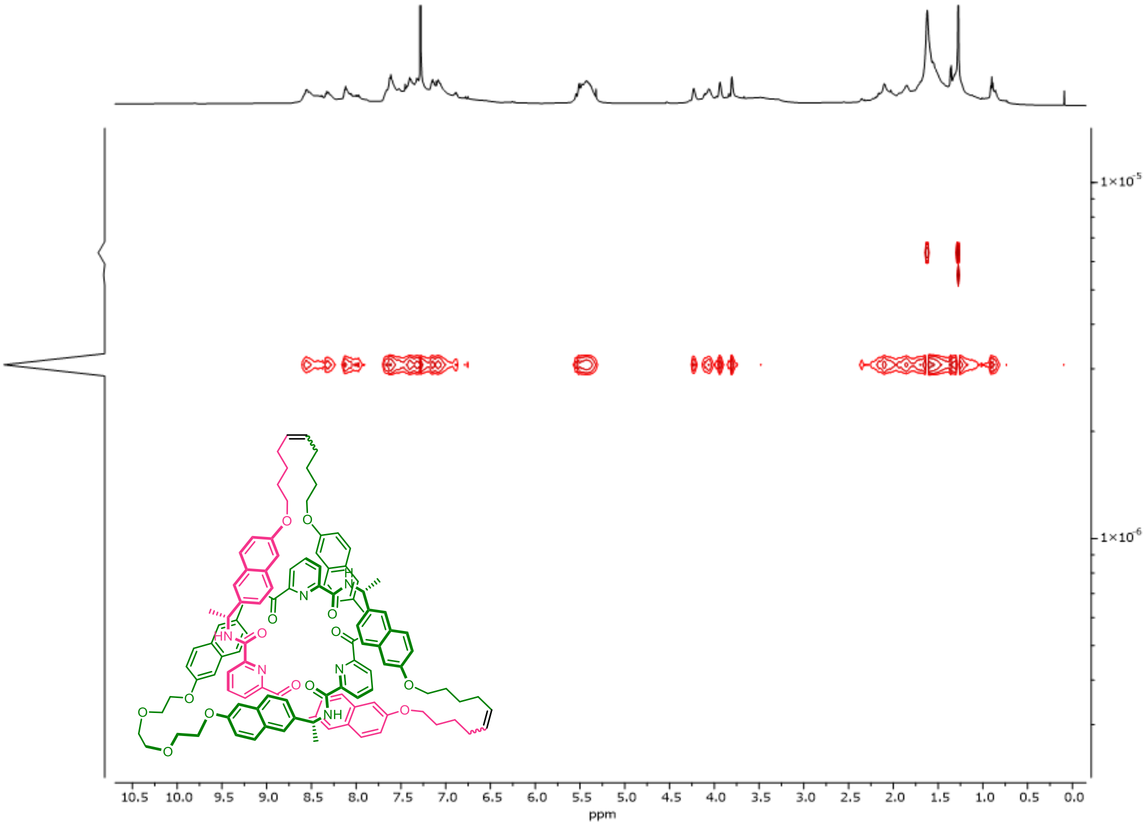

Figure S73. DOSY NMR (600 MHz, $CDCl_3$, 298 K) of Λ-**1**.

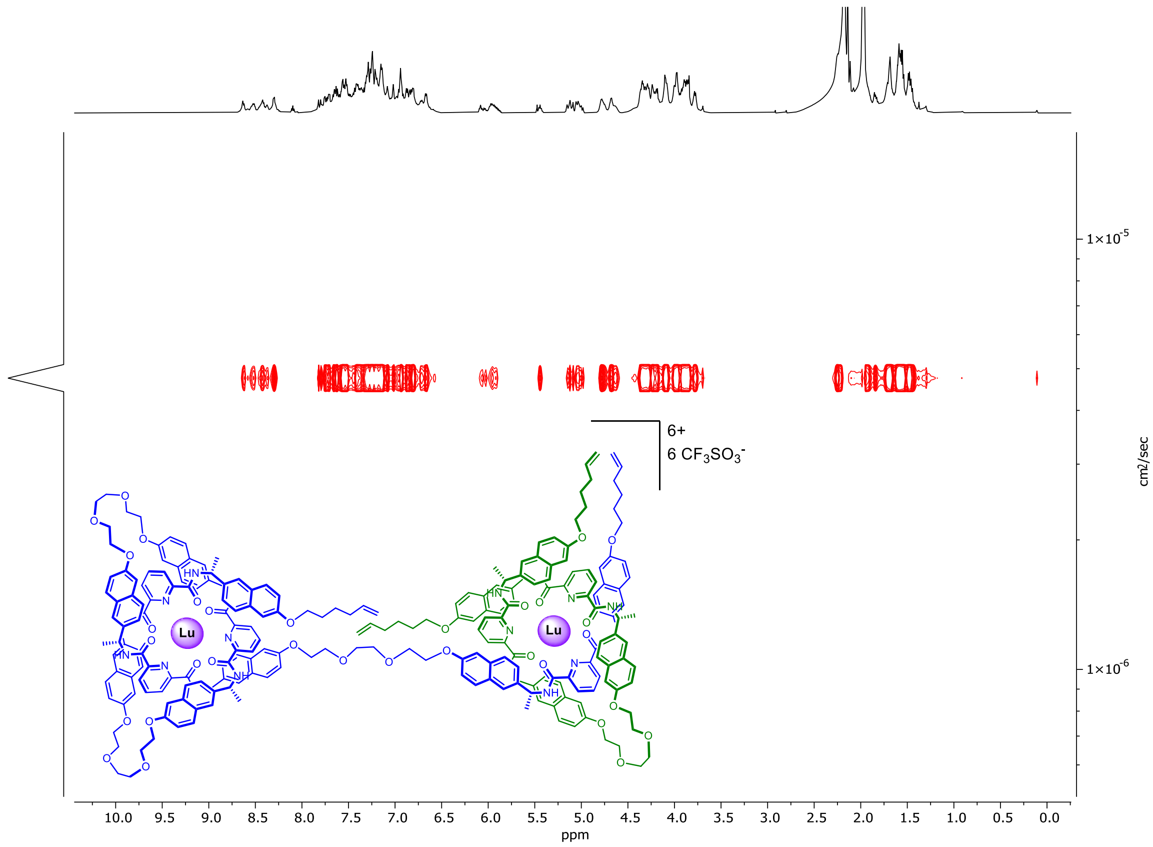

Figure S74. DOSY NMR (600 MHz, MeCN-d_3, 298 K) of (Λ,Λ)-{(R)$_4$-**L4**,(R)$_8$-**L5**•[Lu]$_2$}.

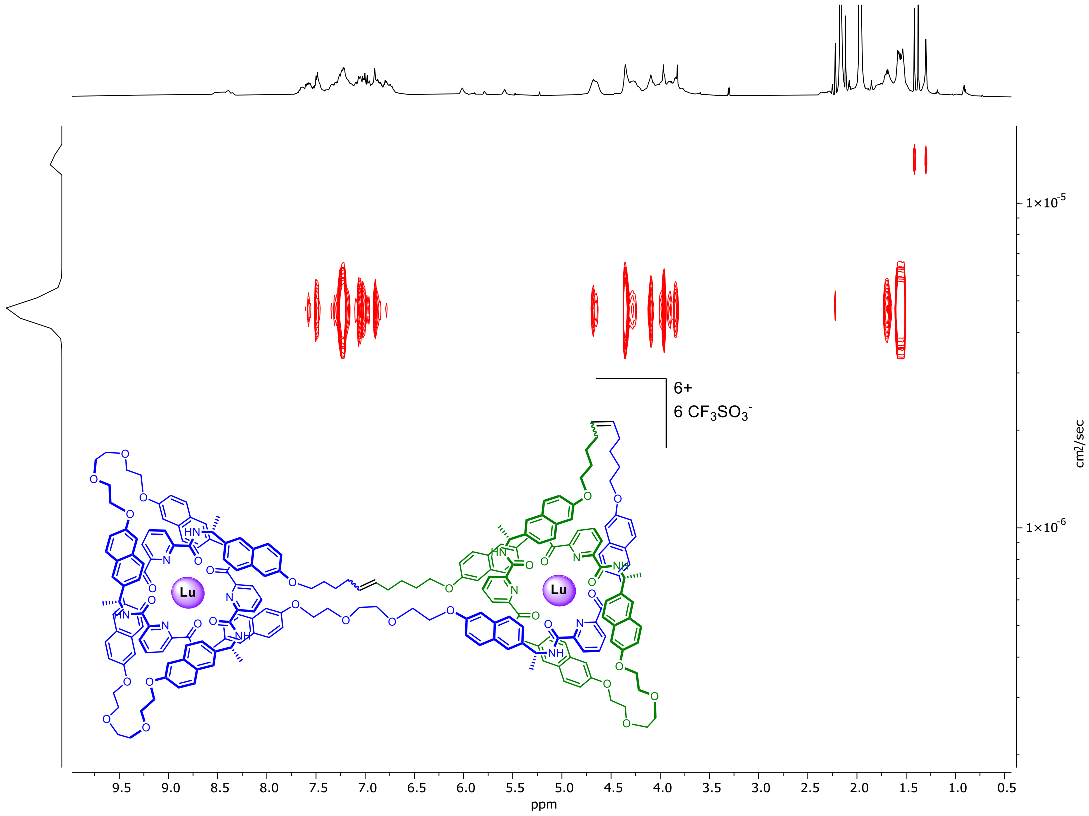

Figure S75. DOSY NMR (600 MHz, MeCN-d_3, 298 K) of (Λ,Λ)-**2**•[Lu]$_2$.

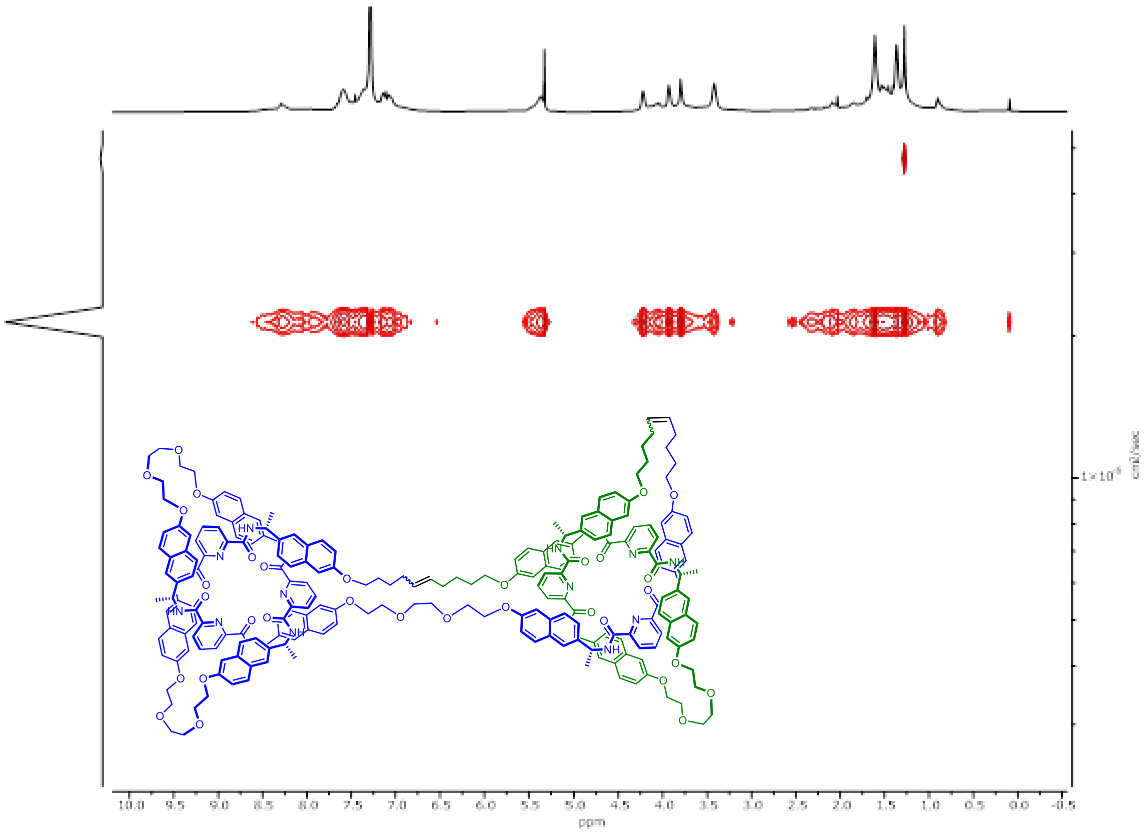

Figure S76. DOSY NMR (600 MHz, $CDCl_3$, 298 K) of (Λ,Λ)-**2**.

Table S1. Hydrodynamic radii D and approximate sizes of entangled compounds.[a]

**Compound**	**Solvent**	**D (10^{10}*m^2/s)**	**R_H (nm)**[b]
**L1**$_3$•[Lu]	MeCN-d_3	5.49	1.15
**L2**$_3$•[Lu]$_2$	MeCN-d_3	5.11	1.23
{**L1**,**L2**•[Lu]}	MeCN-d_3	4.77	1.32
Λ-{(*R*)$_2$-**L3**,(*R*)$_4$-**L4**•[Lu]}	MeCN-d_3	5.11	1.23
Λ-**1**•[Lu]	MeCN-d_3	5.49	1.15
Λ-**1**	$CDCl_3$	3.08	1.33
(Λ,Λ)-{(*R*)$_4$-**L4**,(*R*)$_8$-**L5**•[Lu]$_2$}	MeCN-d_3	4.73	1.33
(Λ,Λ)-**2**•[Lu]$_2$	MeCN-d_3	4.74	1.33
(Λ,Λ)-**2**	$CDCl_3$	2.13	1.91

a) For DOSY sampling conditions, see the general experimental information. b) Determined from the Stokes-Einstein equation assuming spherical compound shape.

S6. Mass Spectra

S6.1 LRESI and HRESI spectra

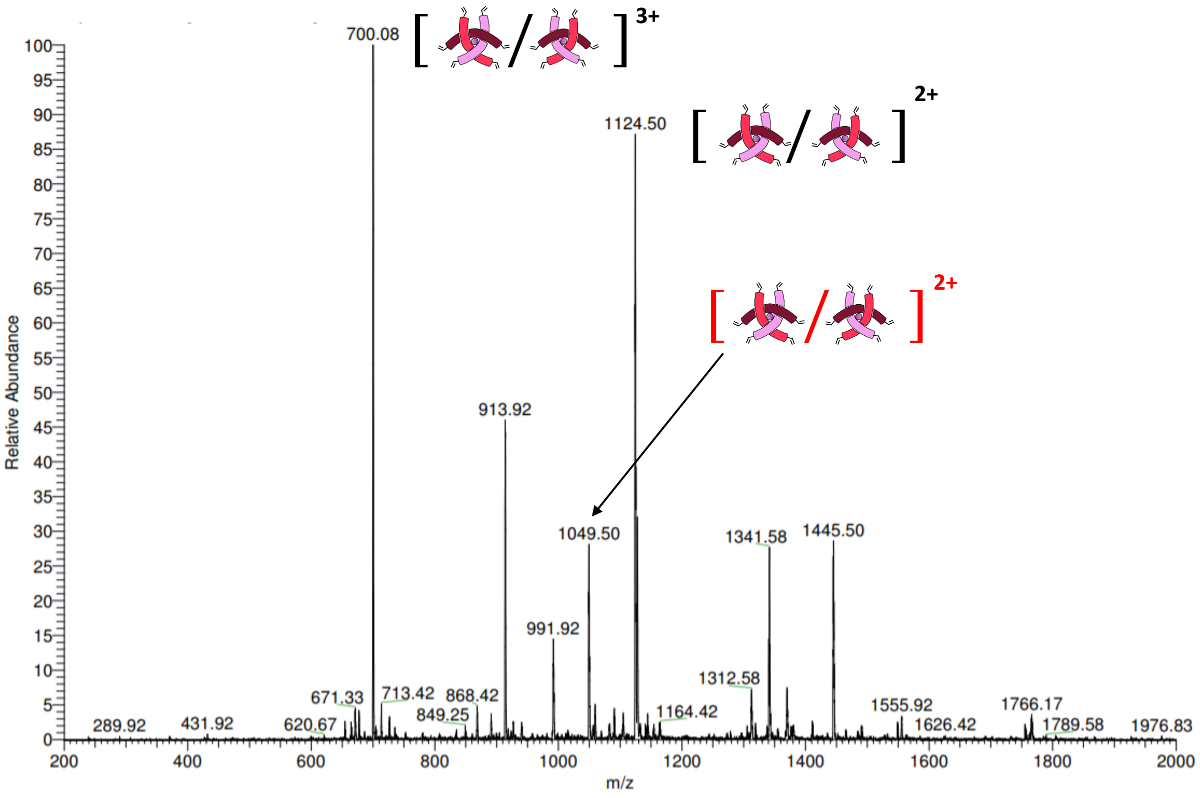

Figure S77. Low resolution ESI-MS(+) of open helicate **L1**$_3$•[Lu] (all peaks observed as $[M{-}n(CF_3SO_3)]^{n+}$ adducts (black) or $[M{-}(n+1)(CF_3SO_3)+H^{-}]^{n+}$ (red)).

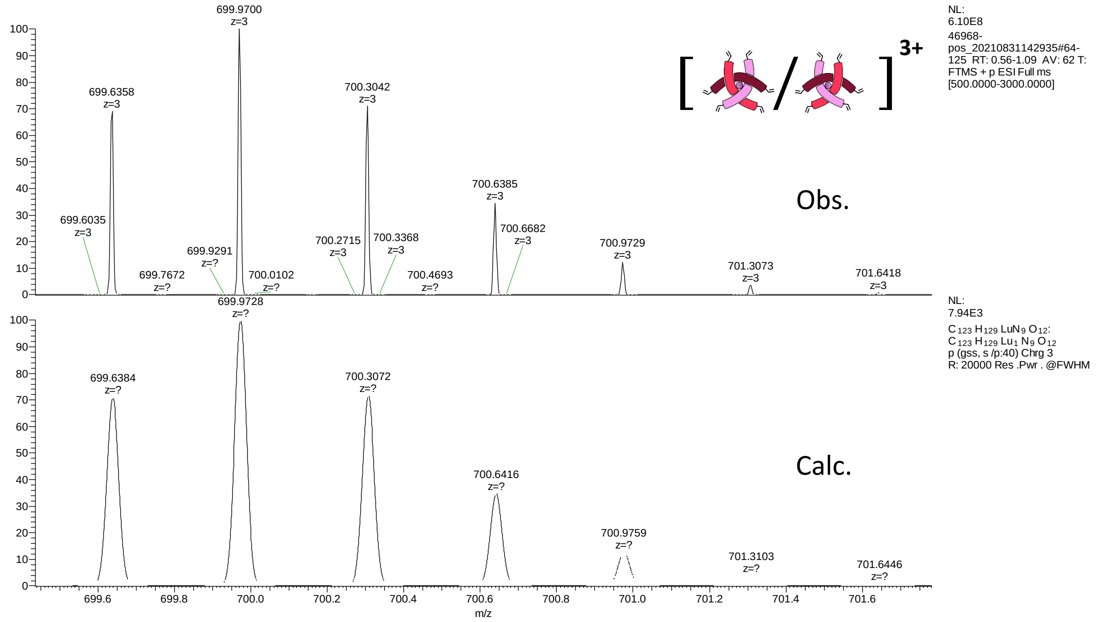

Figure S78. High resolution ESI-MS(+) of open helicate **L1**$_3$•[Lu] showing isotopic distribution of $[M{-}3(CF_3SO_3)]^{3+}$.

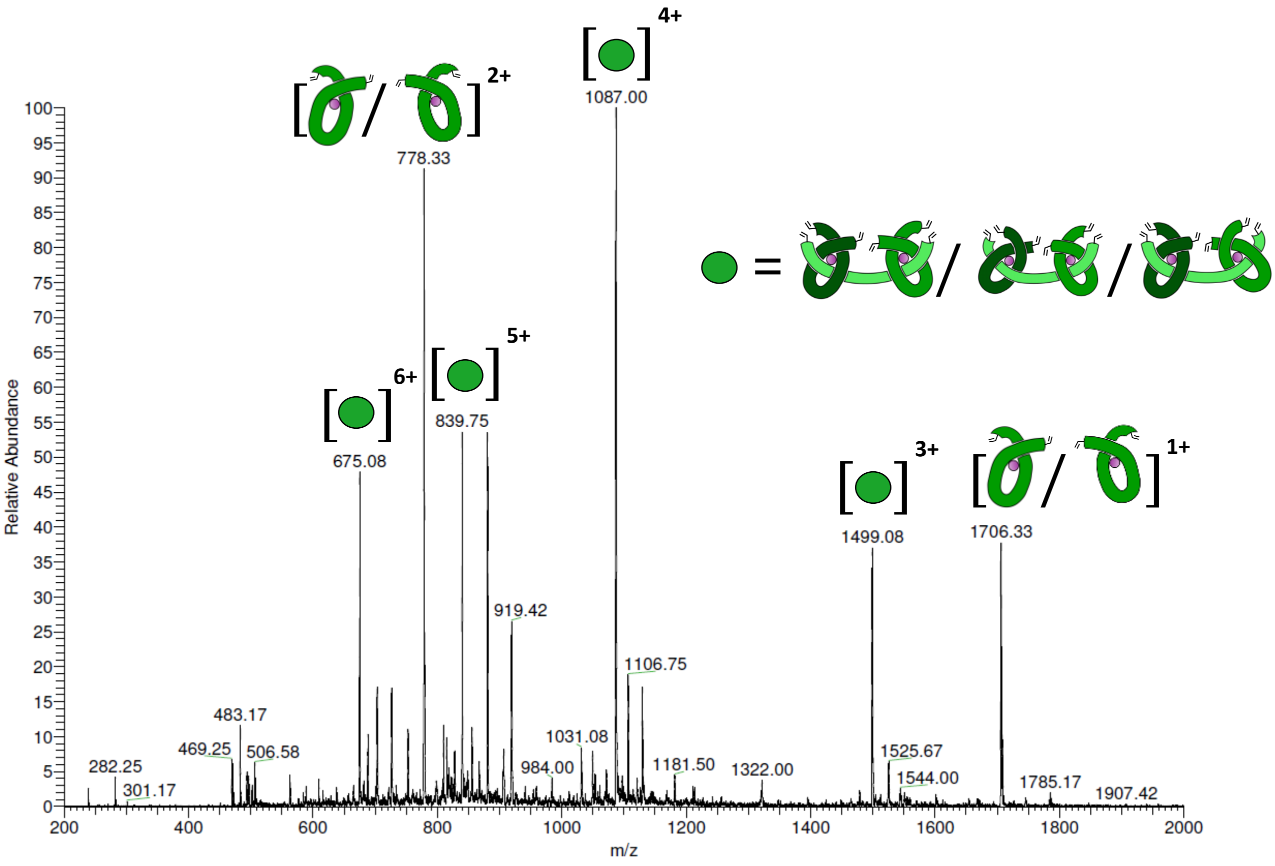

Figure S79. Low resolution ESI-MS(+) of open composite mixture **L2**$_3$•[Lu] (all peaks observed as $[M{-}n(CF_3SO_3)]^{n+}$ adducts).

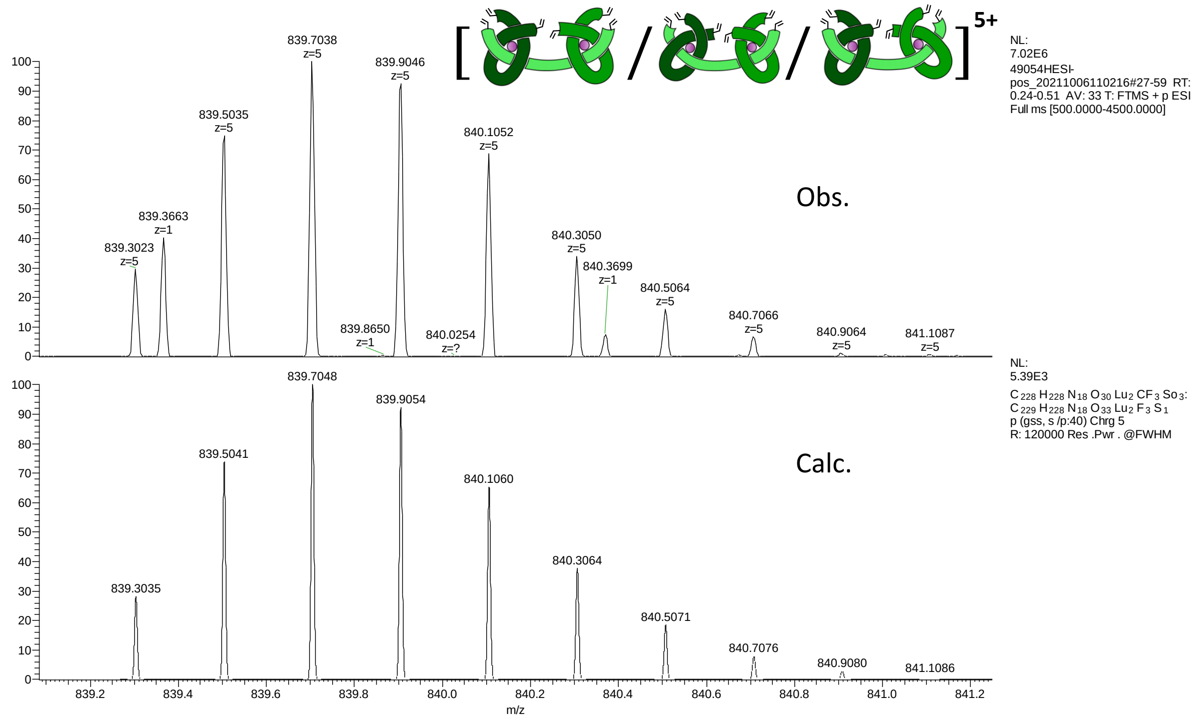

Figure S80. High resolution ESI-MS(+) of open composite mixture **L2**$_3$•[Lu] showing isotopic distribution of $[M{-}5(CF_3SO_3)]^{5+}$.

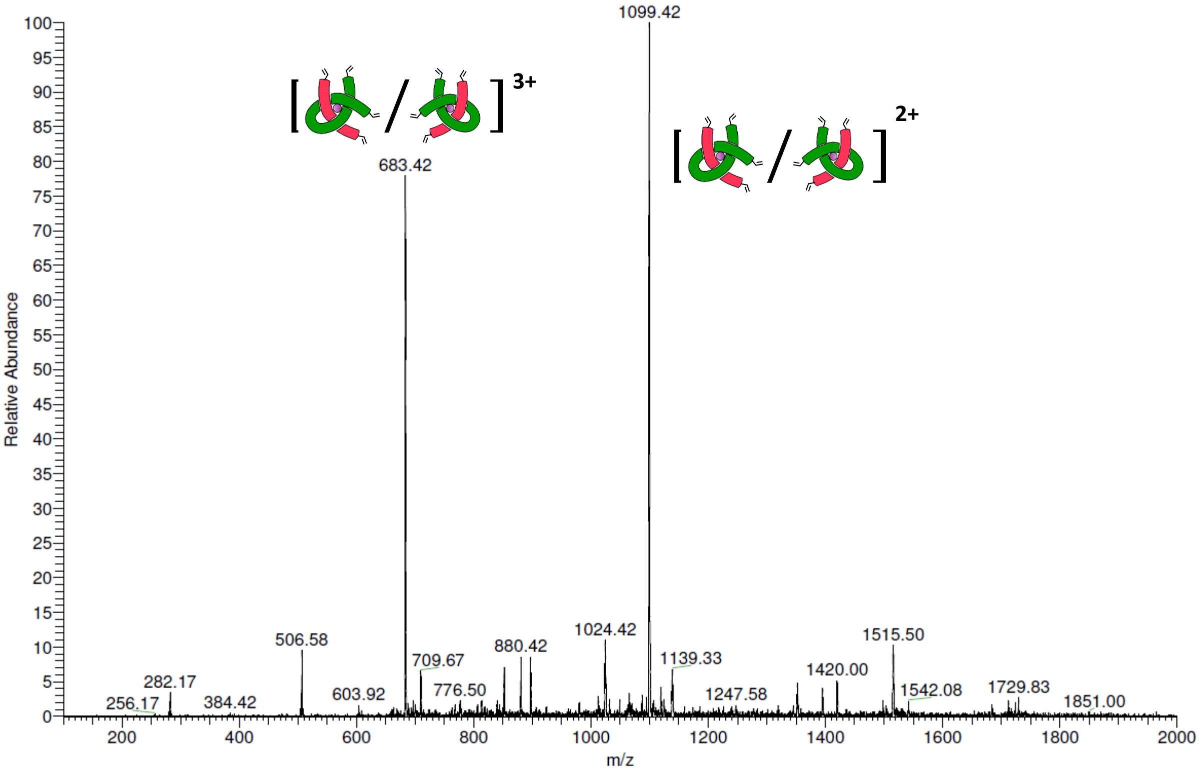

Figure S81. Low resolution ESI-MS(+) of open helicate {**L1**,**L2**•[Lu]} (all peaks observed as $[M{-}n(CF_3SO_3)]^{n+}$ adducts).

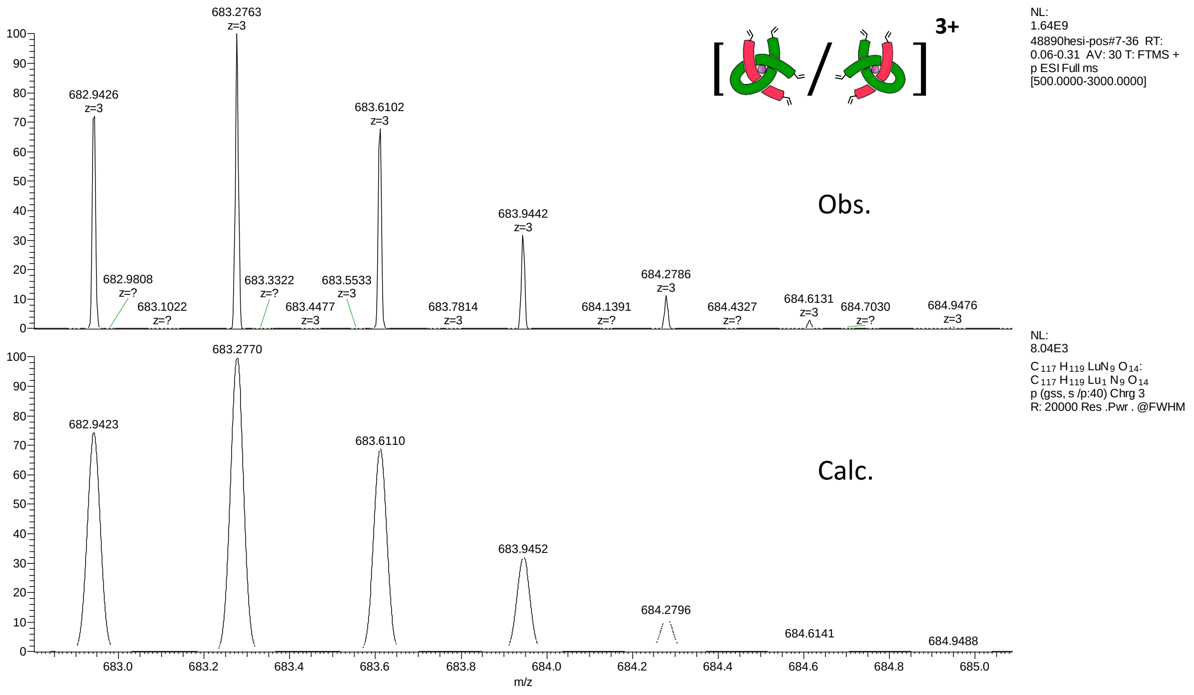

Figure S82. High resolution ESI-MS(+) of open helicate {**L1**,**L2**•[Lu]} showing isotopic distribution of $[M{-}3(CF_3SO_3)]^{3+}$.

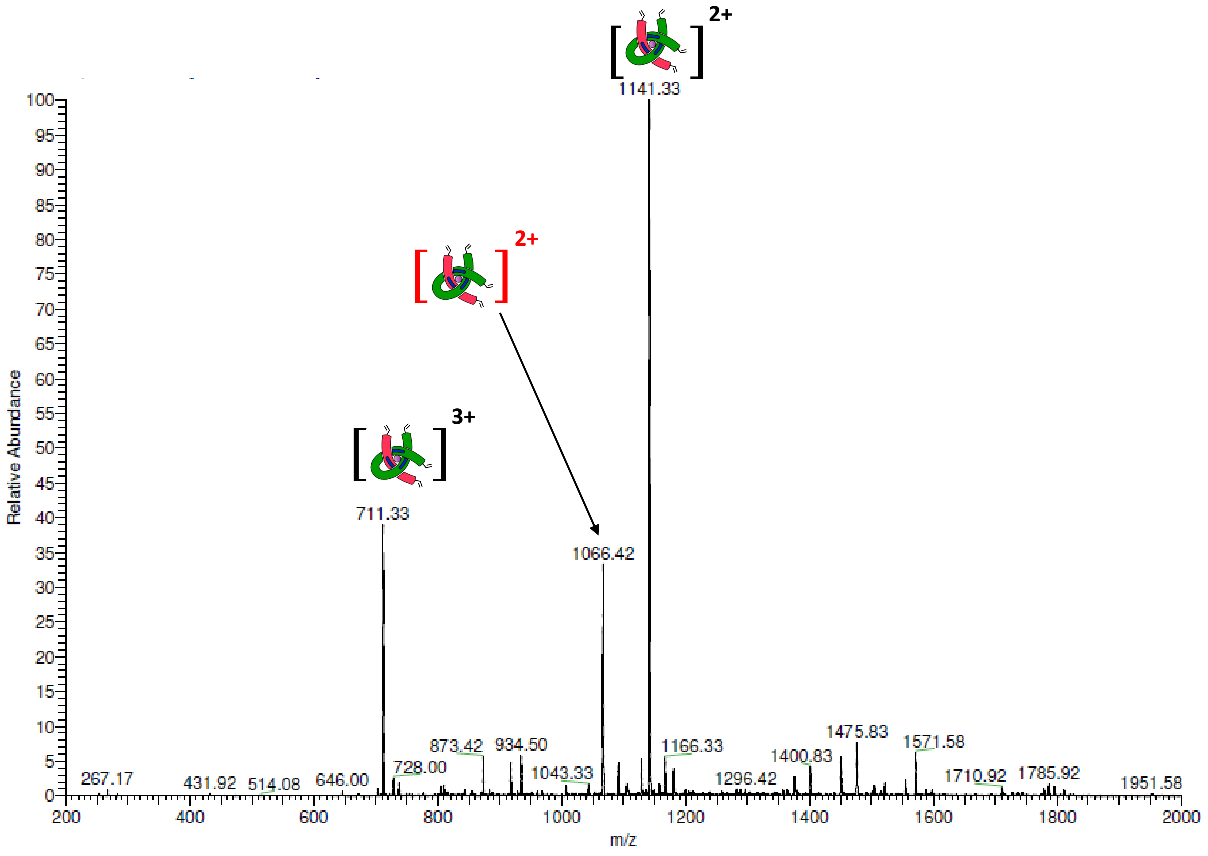

Figure S83. Low resolution ESI-MS(+) of open helicate Λ-{(*R*)$_2$-**L3**,(*R*)$_4$-**L4**•[Lu]} (all peaks observed as [M−n(CF$_3$SO$_3$)]$^{n+}$ adducts (black) or [M−(n+1)(CF$_3$SO$_3$)+H$^-$]$^{n+}$ (red)).

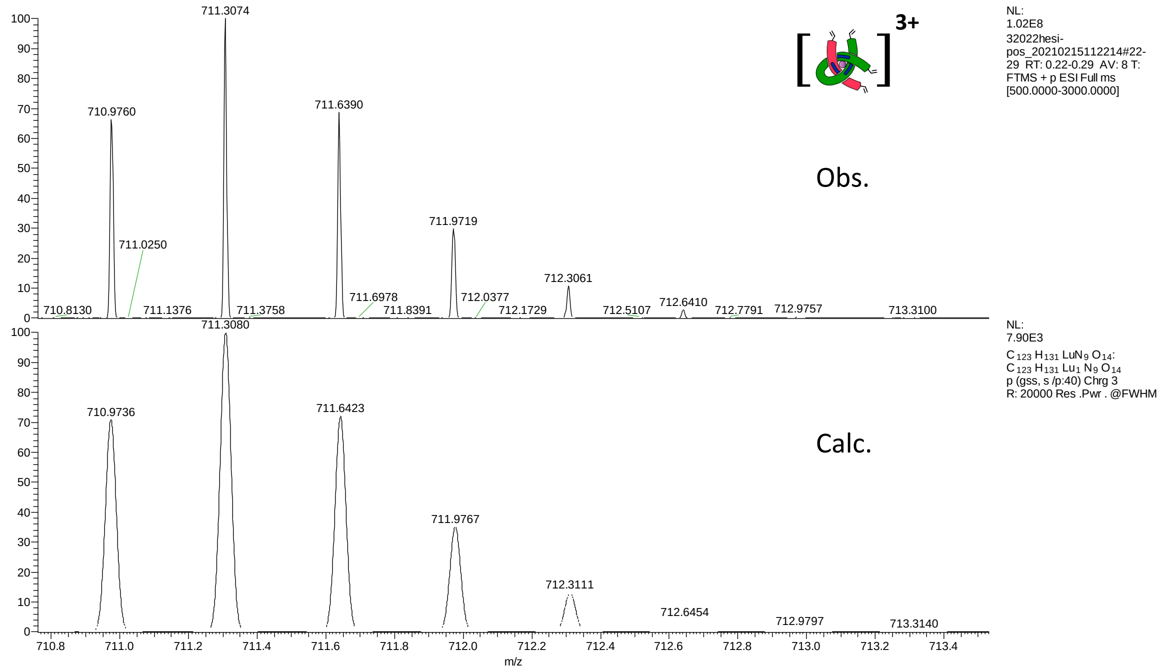

Figure S84. High resolution ESI-MS(+) of open helicate Λ-{(*R*)$_2$-**L3**,(*R*)$_4$-**L4**•[Lu]} showing isotopic distribution of [M-3(CF$_3$SO$_3$)]$^{3+}$.

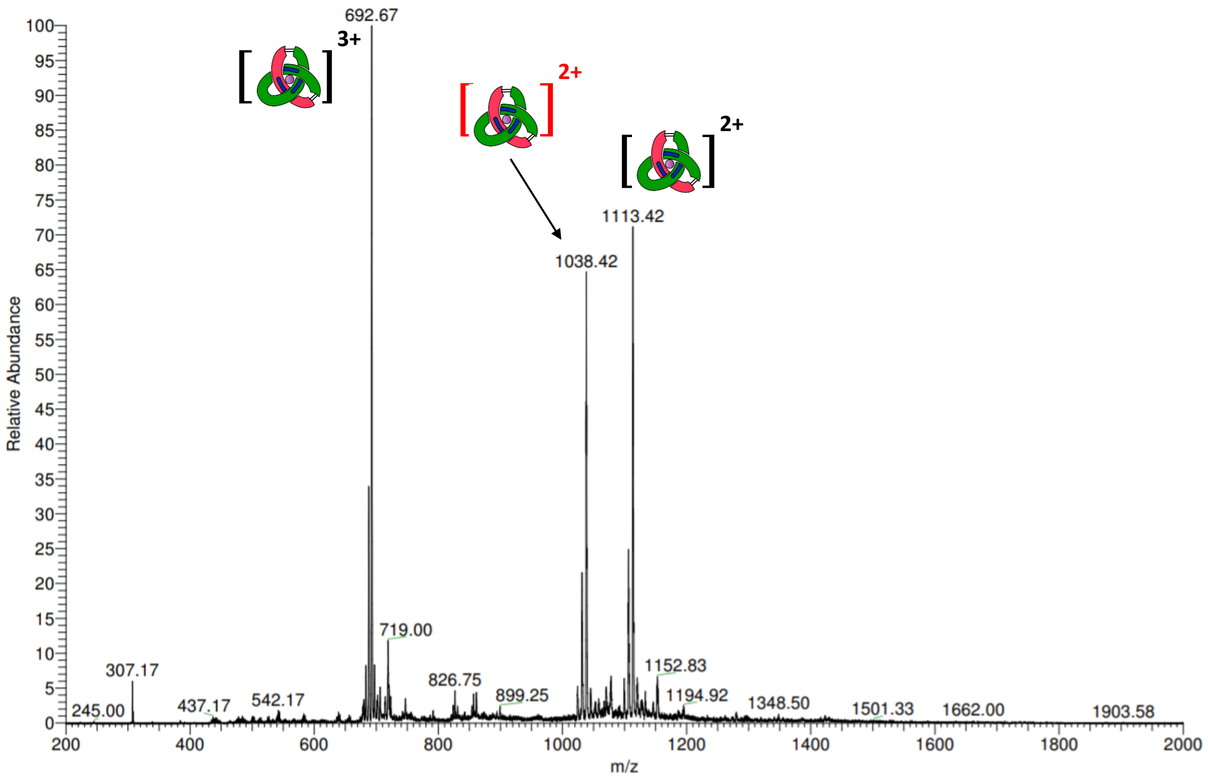

Figure S85. Low resolution ESI-MS(+) of trefoil knot Λ-**1**•[Lu] (all peaks observed as $[M{-}n(CF_3SO_3)]^{n+}$ adducts (black) or $[M{-}(n+1)(CF_3SO_3)+H^{-}]^{n+}$ (red)).

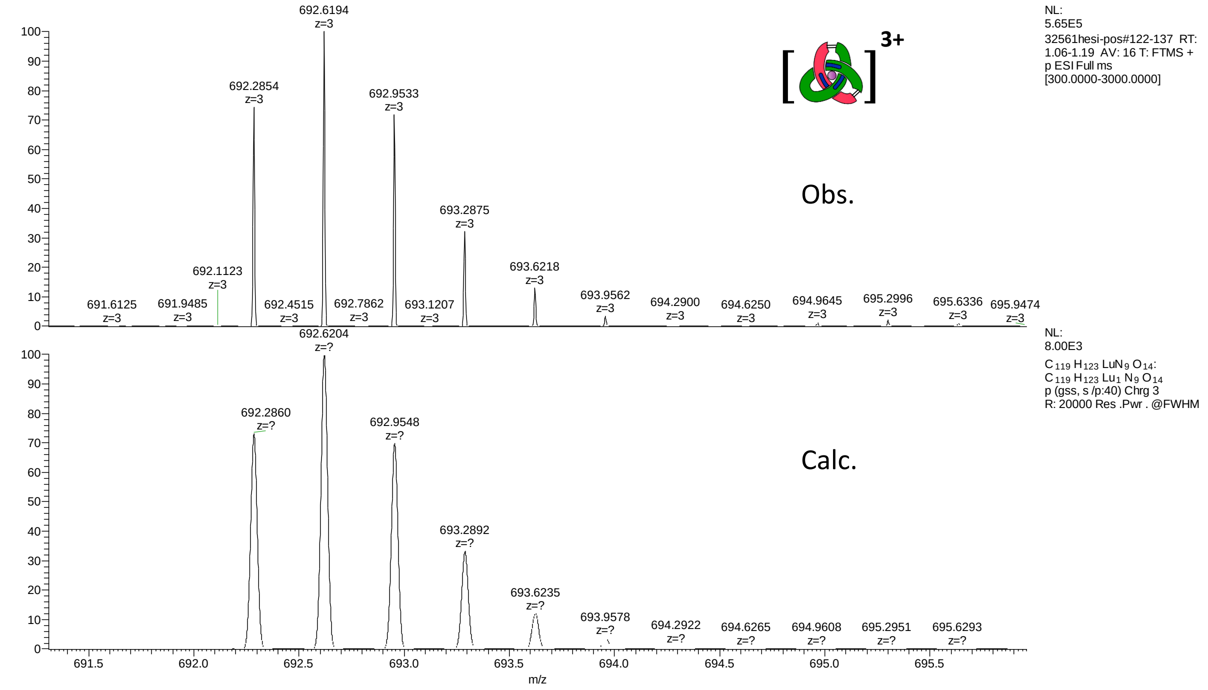

Figure S86. High resolution ESI-MS(+) of trefoil knot Λ-**1**•[Lu] showing isotopic distribution of $[M{-}3(CF_3SO_3)]^{3+}$.

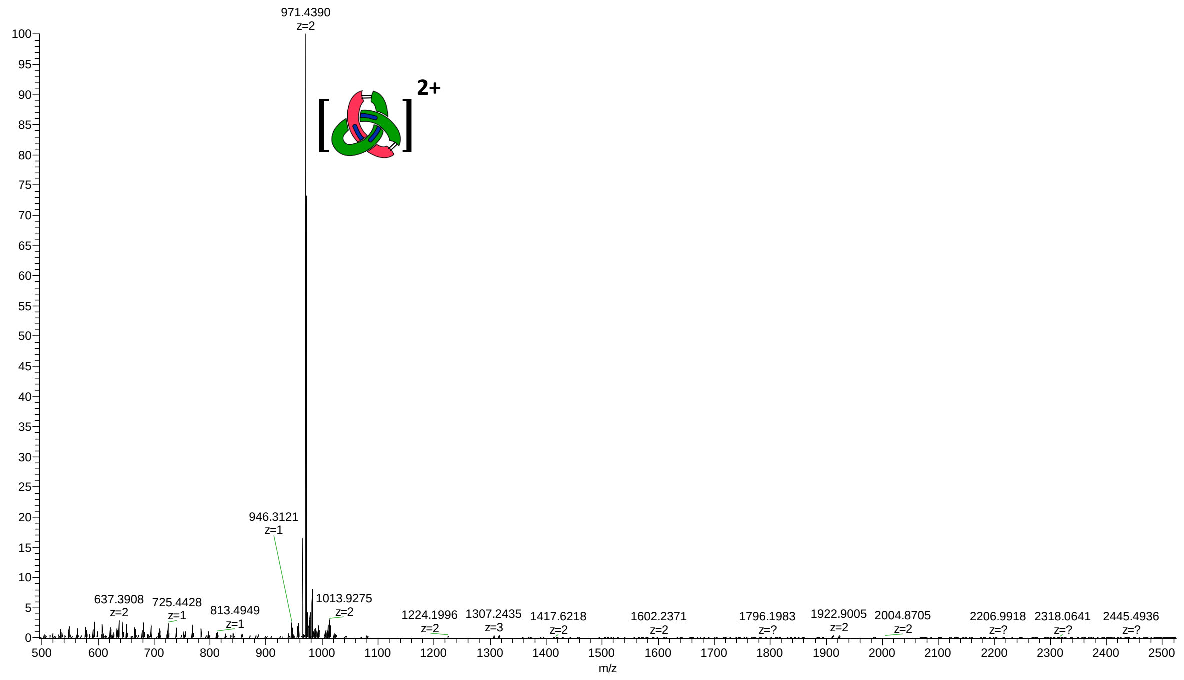

Figure S87. High resolution ESI-MS(+) of trefoil knot Λ-**1** $[M+H+K]^{2+}$.

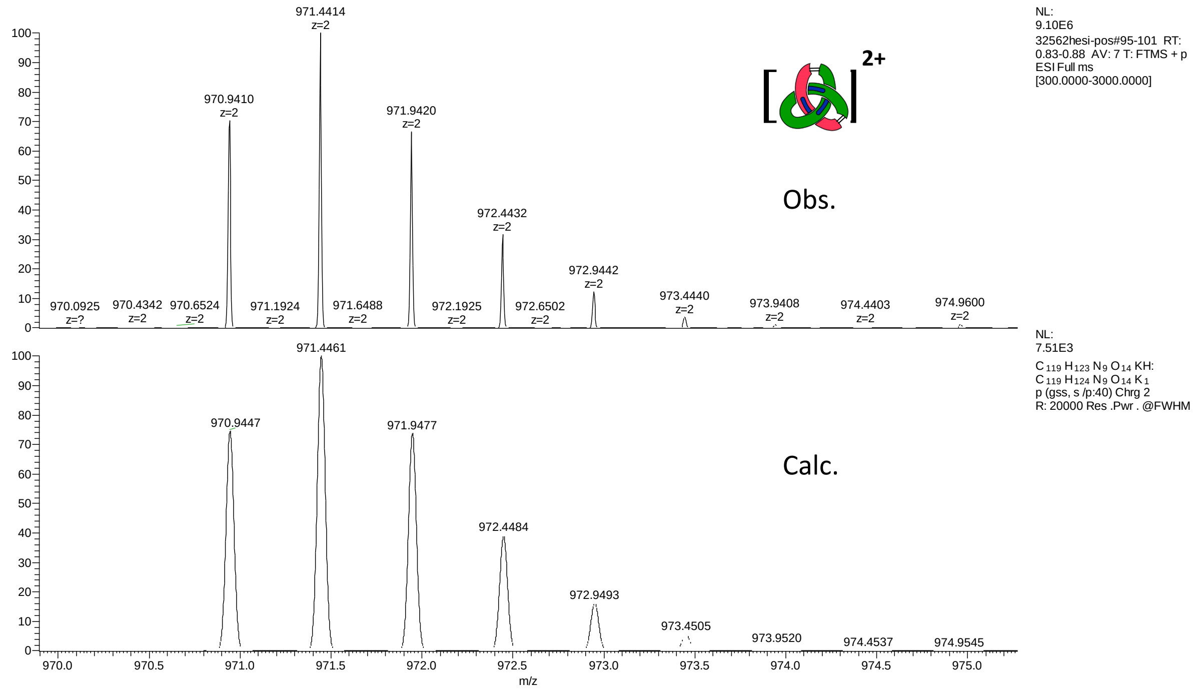

Figure S88. High resolution ESI-MS(+) of trefoil knot Λ-**1** showing isotopic distribution of $[M+H+K]^{2+}$.

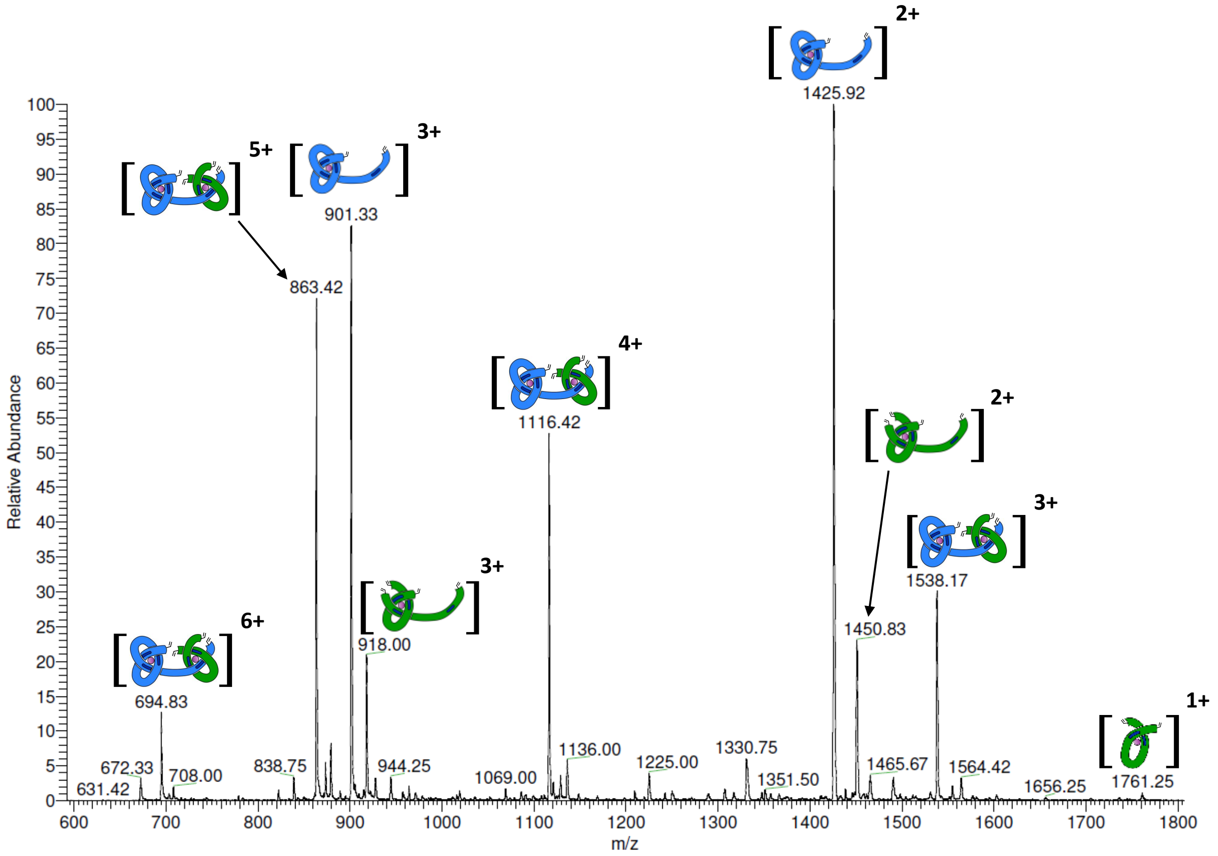

Figure S89. Low resolution ESI-MS(+) of open granny knot (Λ,Λ)-{(*R*)$_4$-**L4**,(*R*)$_8$-**L5**•[Lu]$_2$} (all peaks observed as [M−n(CF_3SO_3)]$^{n+}$ adducts) and side products.

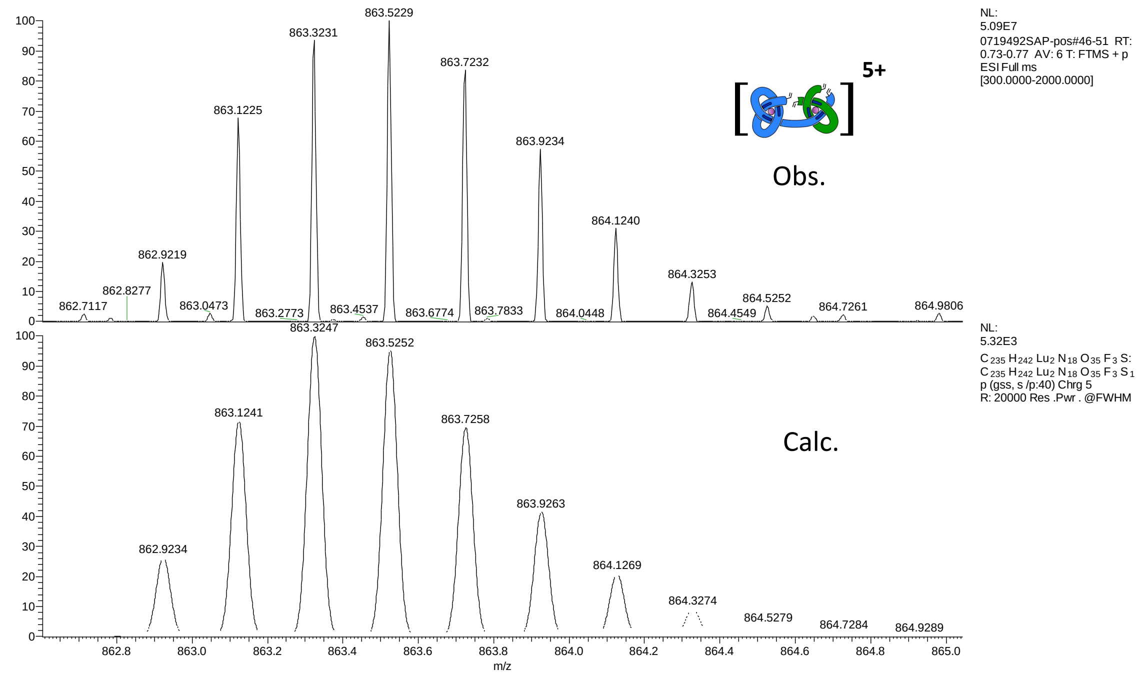

Figure S90. High resolution ESI-MS(+) of open granny knot (Λ,Λ)-{(*R*)$_4$-**L4**,(*R*)$_8$-**L5**•[Lu]$_2$} showing isotopic distribution of [M-5(CF_3SO_3)]$^{5+}$.

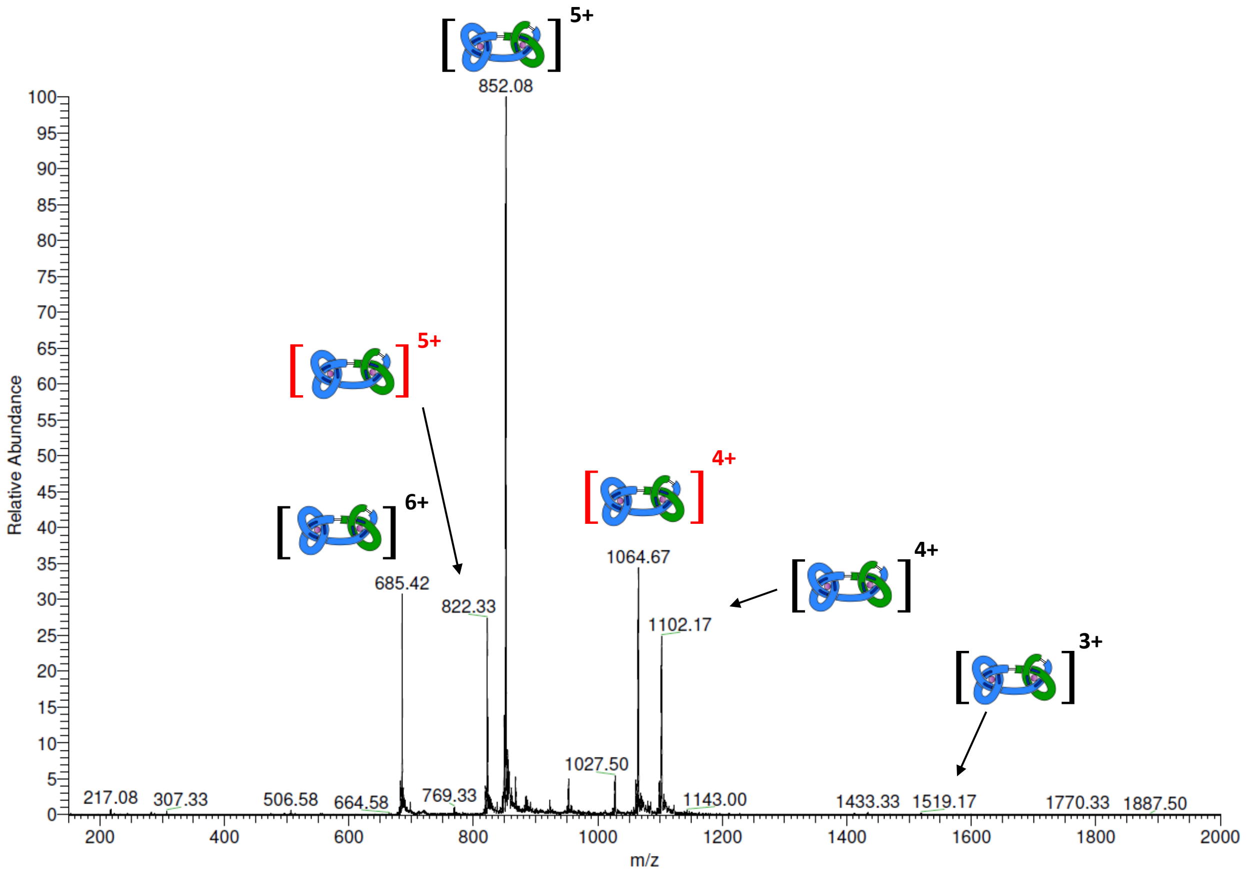

Figure S91. Low resolution ESI-MS(+) of closed granny knot (Λ,Λ)-**2**•$[Lu]_2$ (all peaks observed as $[M{-}n(CF_3SO_3)]^{n+}$ adducts (black) or $[M{-}(n{+}1)(CF_3SO_3){+}H^{-}]^{n+}$ (red)).

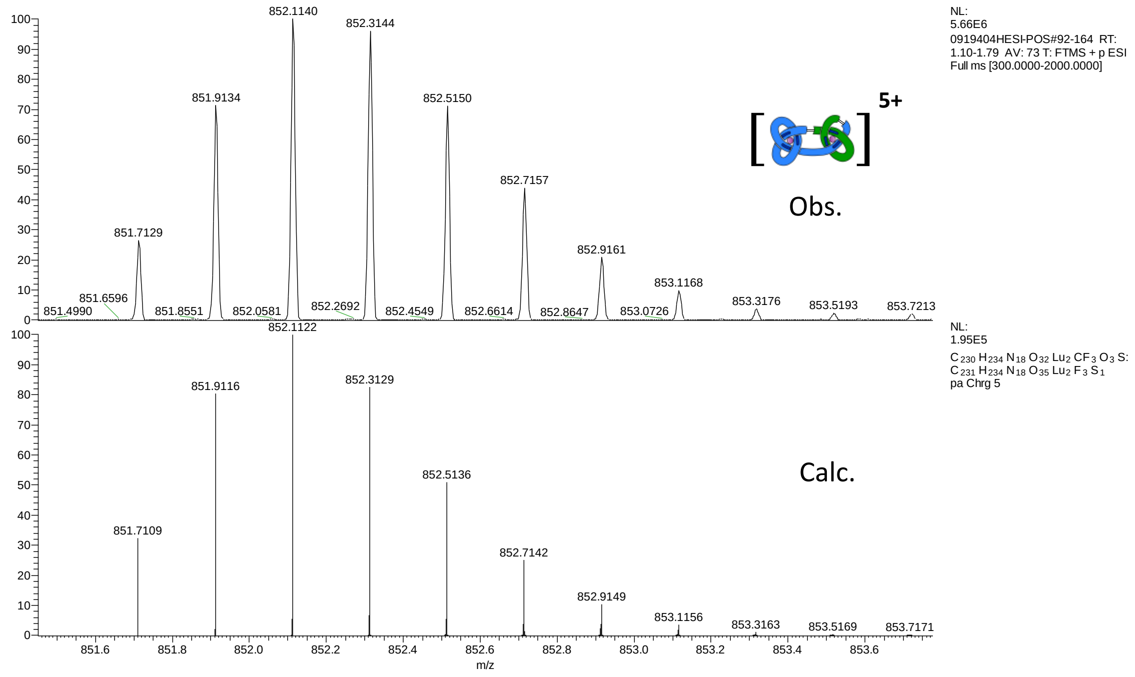

Figure S92. High resolution ESI-MS(+) of closed granny knot (Λ,Λ)-**2**•$[Lu]_2$ showing isotopic distribution of $[M\text{-}5(CF_3SO_3)]^{5+}$.

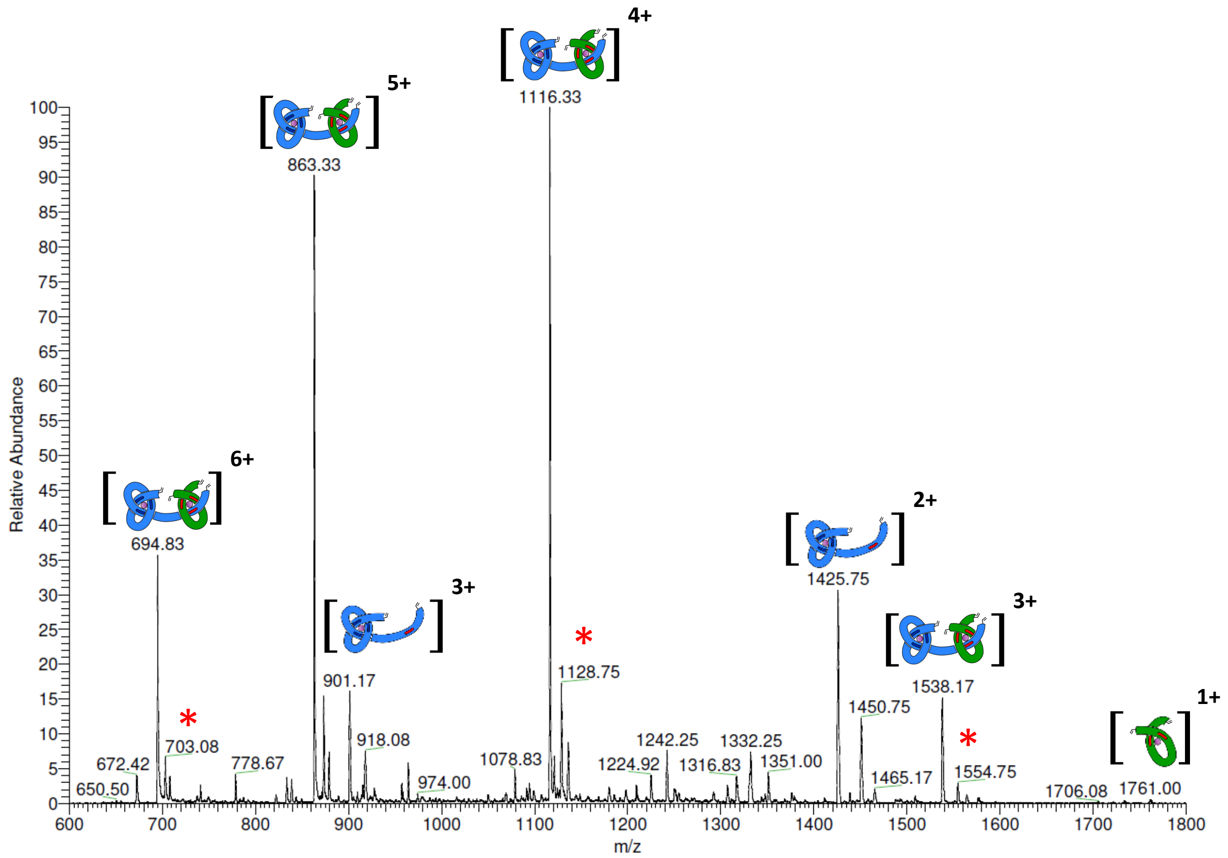

Figure S93. Low resolution ESI-MS(+) of open square knot (Λ,Δ)-{(*S*)$_4$-**L4**,(*S*)$_2$(*R*)$_6$-**L5**•[Lu]$_2$} (all peaks observed as [M−n(CF$_3$SO$_3$)]$^{n+}$ adducts) and side products. Peaks marked * show (Δ,Δ)-((*S*)$_4$-**L4**)$_3$•[Lu]$_2$ side product.

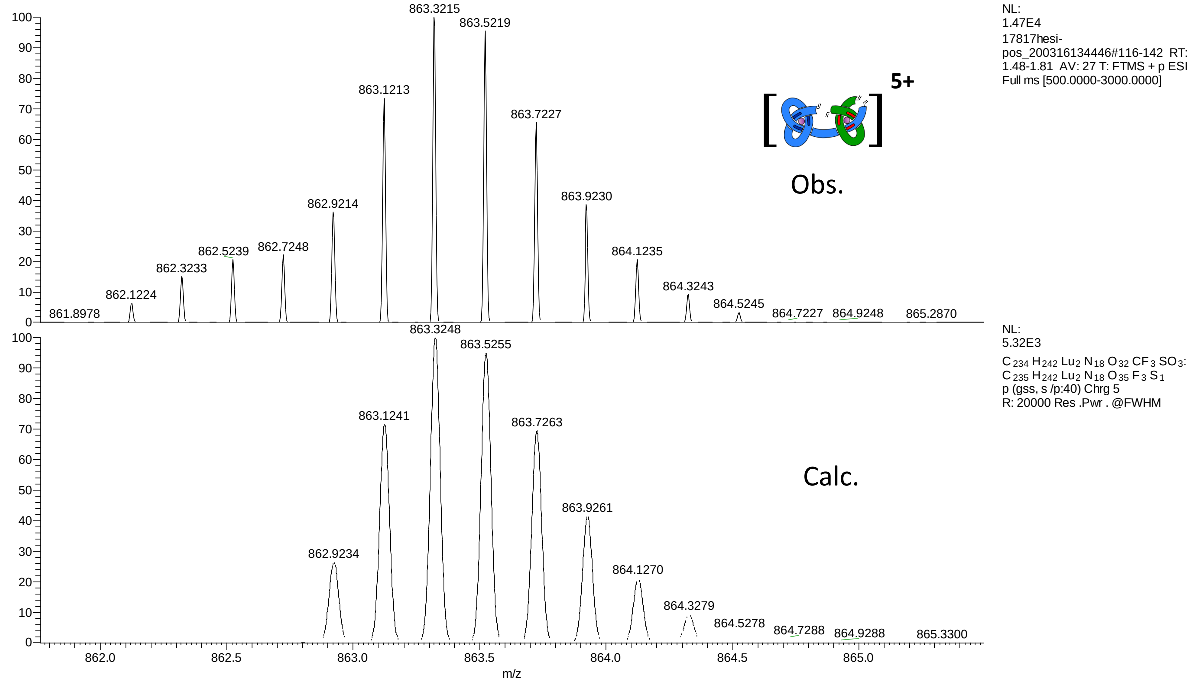

Figure S94. High resolution ESI-MS(+) of open square knot (Λ,Δ)-{(*S*)$_4$-**L4**,(*S*)$_2$(*R*)$_6$-**L5**•[Lu]$_2$} showing isotopic distribution of [M-5(CF$_3$SO$_3$)]$^{5+}$.

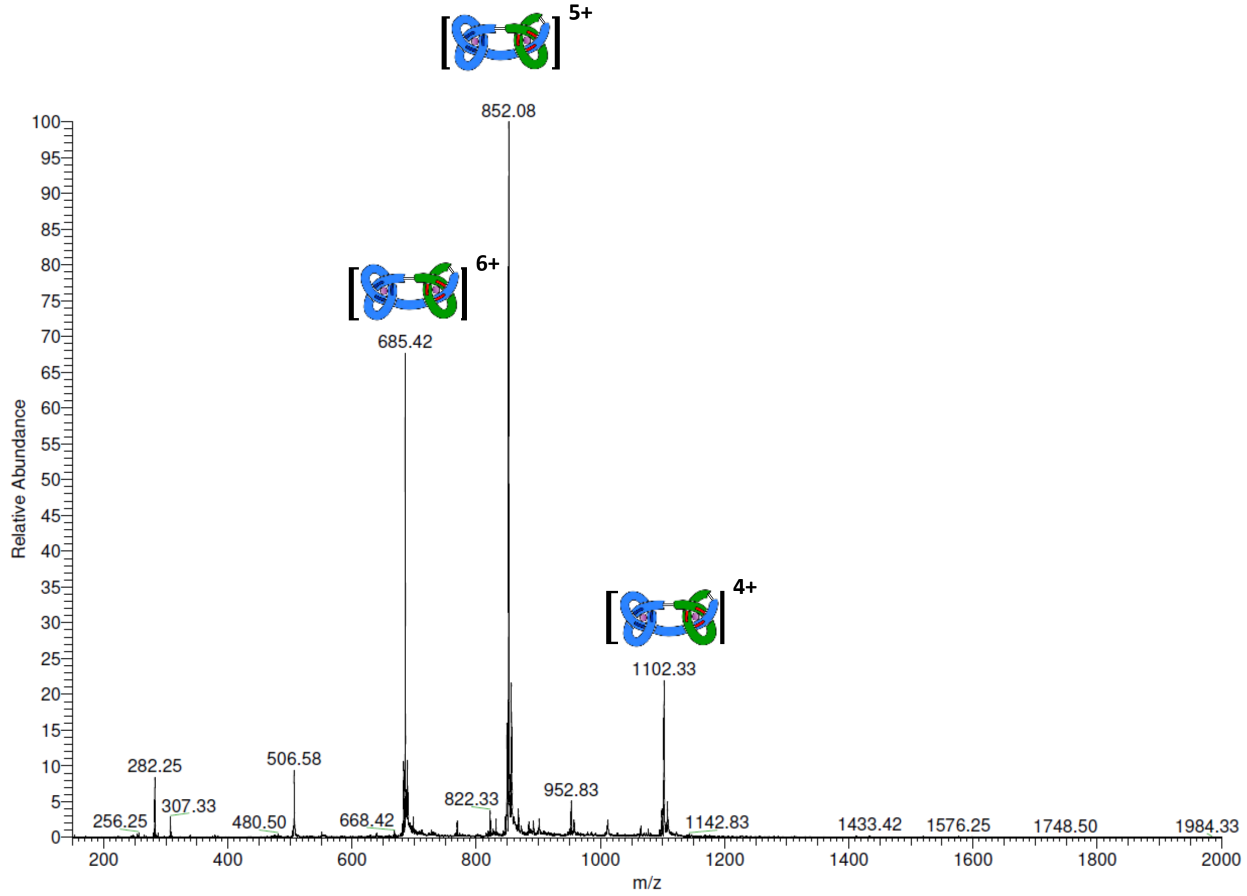

Figure S95. Low resolution ESI-MS(+) of closed square knot (Λ,Δ)-**2**•[Lu]$_2$ (all peaks observed as $[M{-}n(CF_3SO_3)]^{n+}$ adducts).

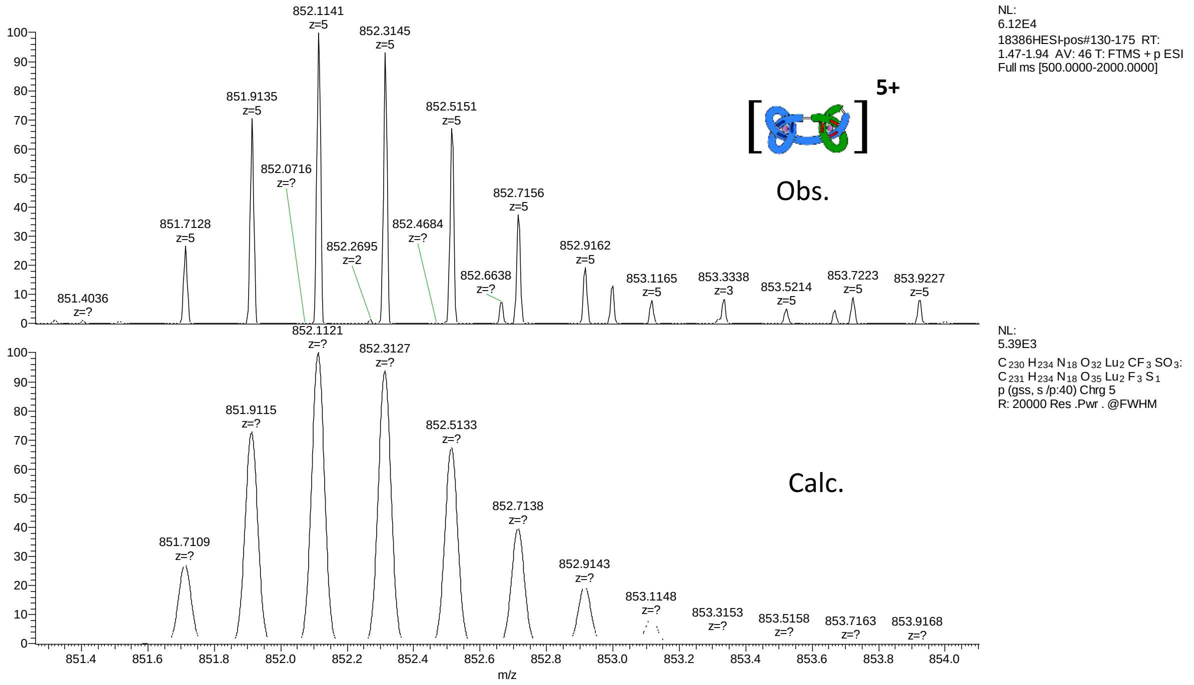

Figure S96. High resolution ESI-MS(+) of closed square knot (Λ,Δ)-**2**•[Lu]$_2$ showing isotopic distribution of $[M{-}5(CF_3SO_3)]^{5+}$.

S6.2 MALDI-TOF Spectra

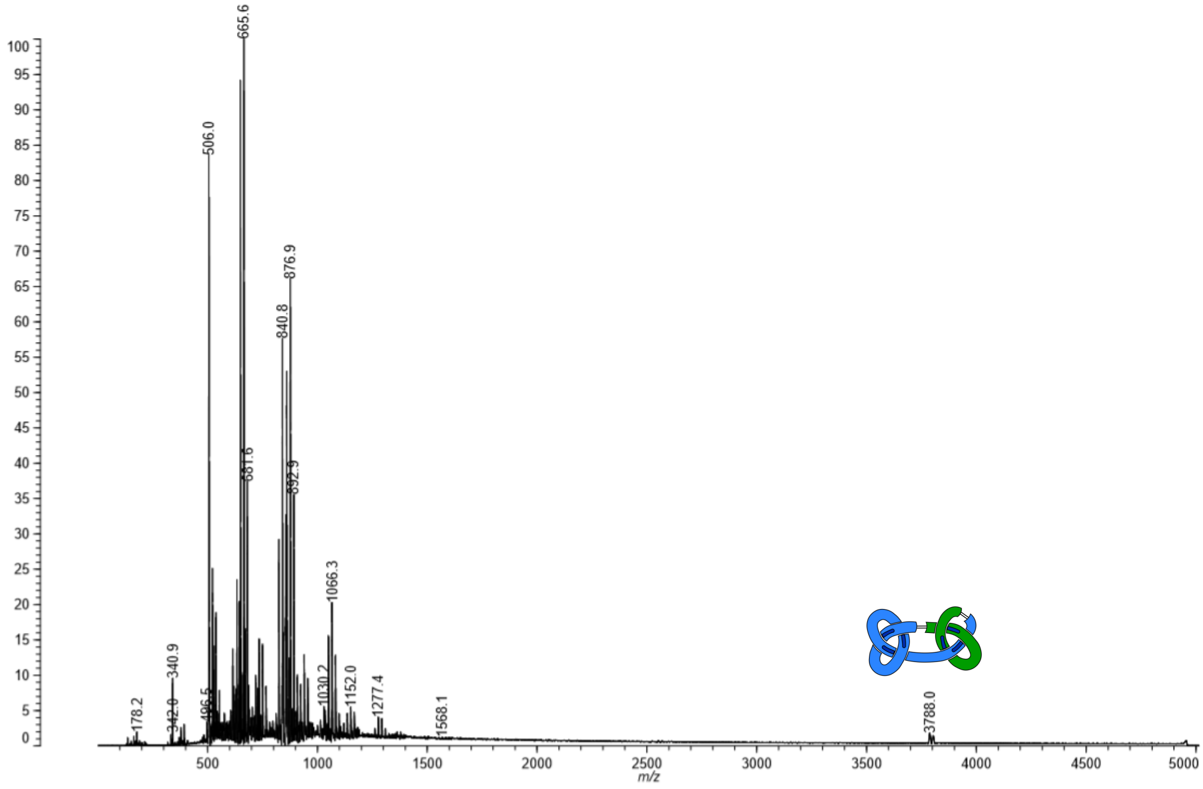

Figure S97. MALDI-TOF (MeOH, CHCA) spectrum of organic granny knot (Λ,Λ)-**2.**

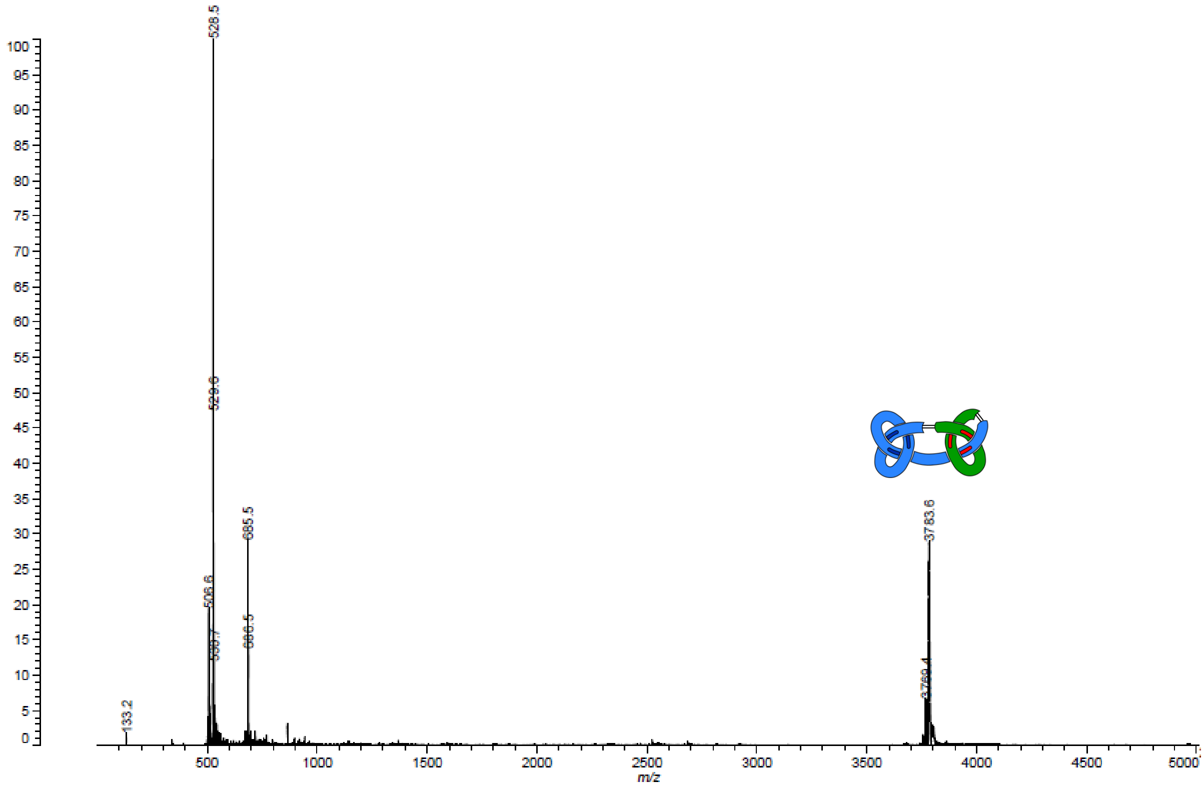

Figure S98. MALDI-TOF (MeOH, CHCA) spectrum of organic square knot (Λ,Δ)-**2.**

S7. CD and Absorbance Spectra

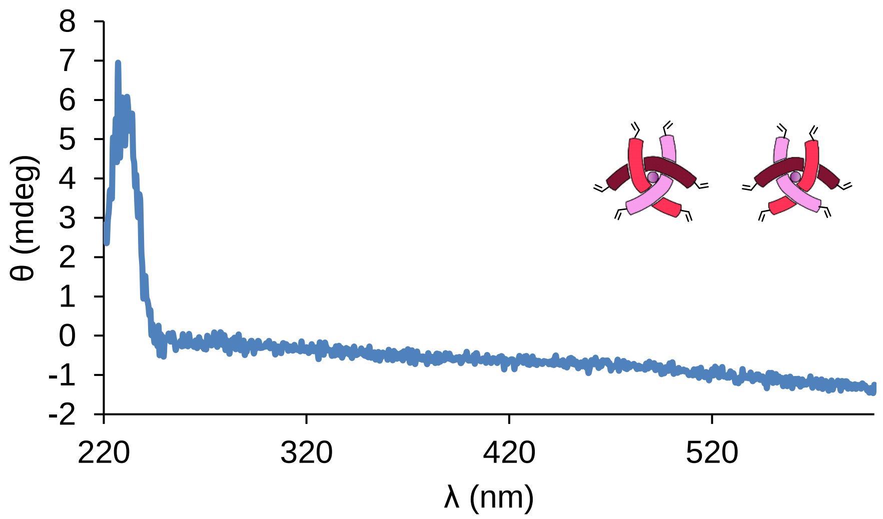

Figure S99. CD spectrum (5.0×10^{-5} M, MeCN, 298 K) of **L1**$_3$•[Lu].

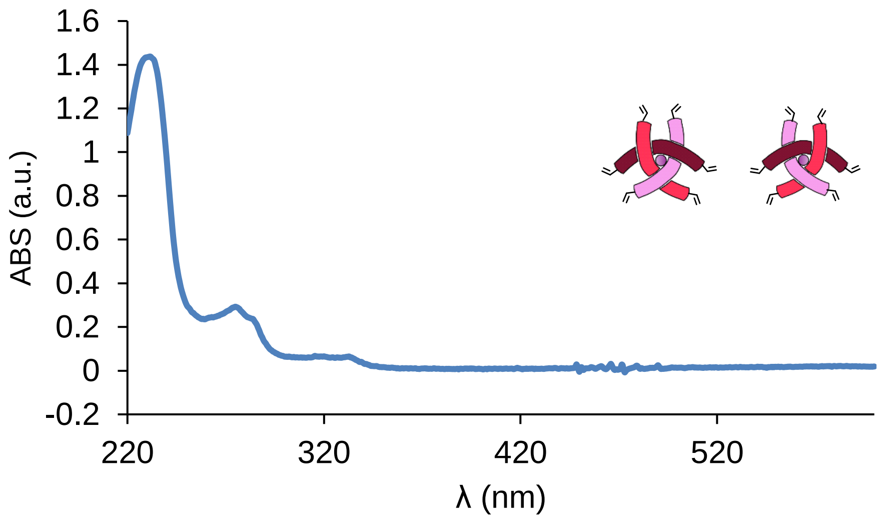

Figure S100. UV/vis spectrum (5.0×10^{-5} M, MeCN, 298 K) of **L1**$_3$•[Lu].

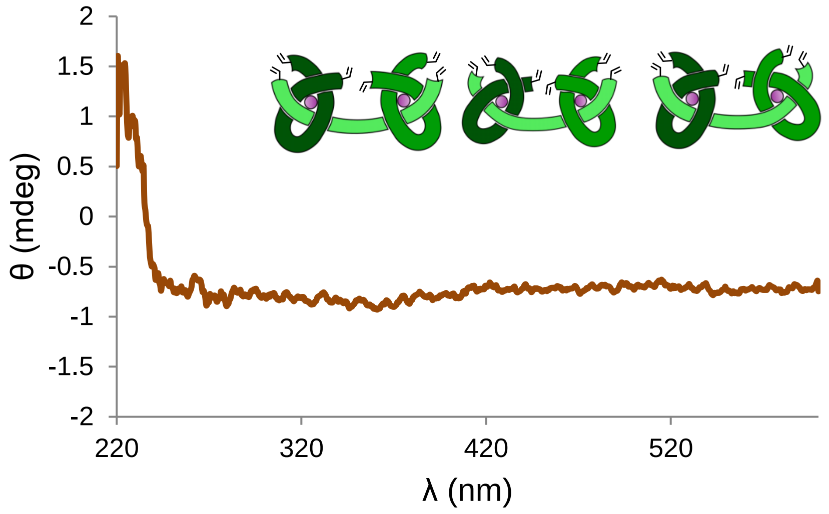

Figure S101. CD spectrum (5.0×10^{-5} M, MeCN, 298 K) of **L2**$_3$•[Lu]$_2$.

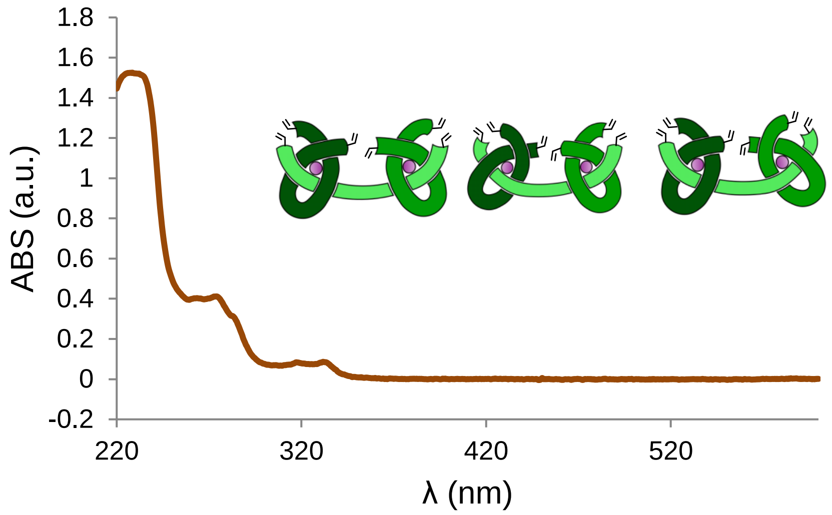

Figure S102. UV/vis spectrum (5.0×10^{-5} M, MeCN, 298 K) of **L2**$_3$•[Lu]$_2$.

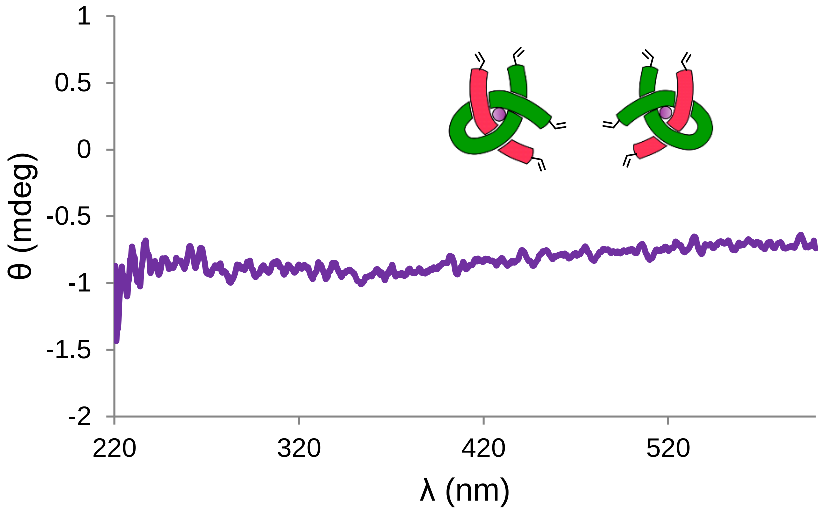

Figure S103. CD spectrum (5.0×10^{-5} M, MeCN, 298 K) of {**L1**,**L2**•[Lu]}.

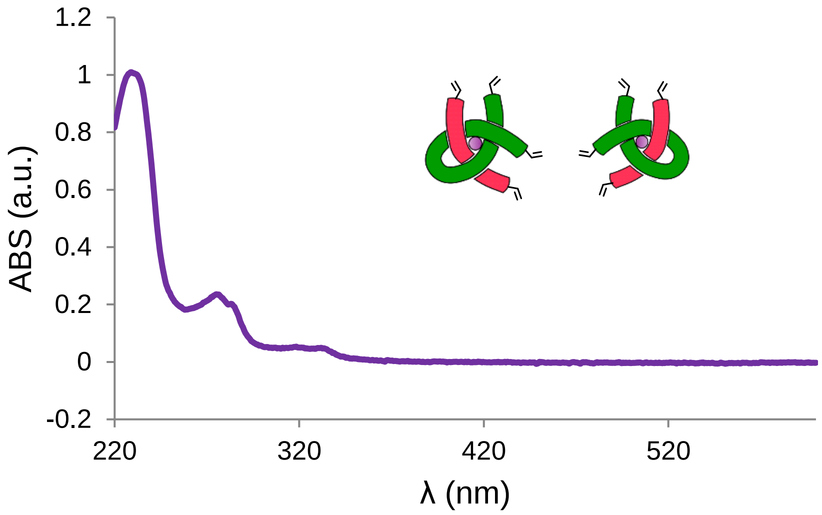

Figure S104. UV/vis spectrum (5.0×10^{-5} M, MeCN, 298 K) of {**L1**,**L2**•[Lu]}.

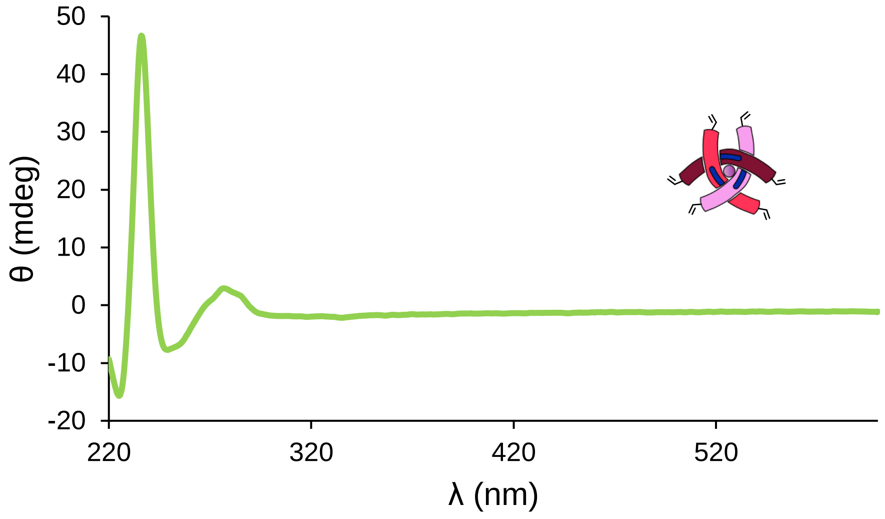

Figure S105. CD spectrum (5.0×10^{-5} M, MeCN, 298 K) of Λ-((*R*)$_2$-**L3**)$_3$•[Lu].

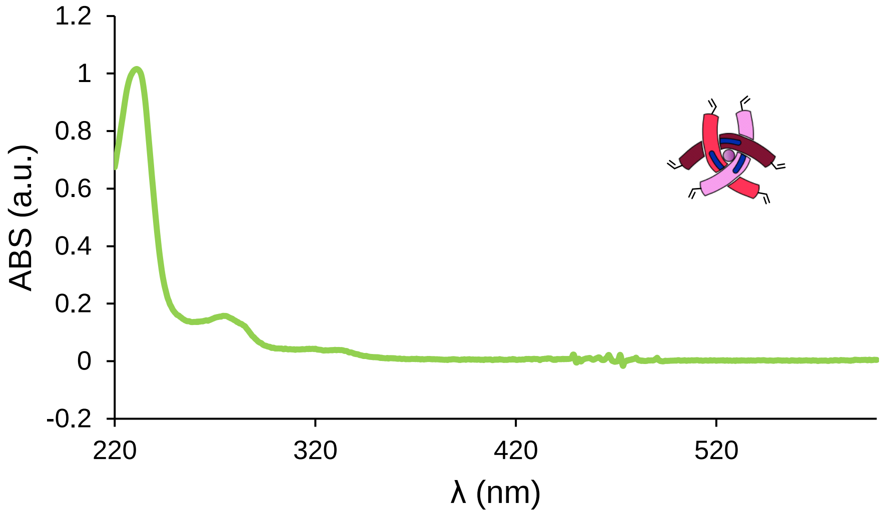

Figure S106. UV/vis spectrum (5.0×10^{-5} M, MeCN, 298 K) of Λ-((*R*)$_2$-**L3**)$_3$•[Lu].

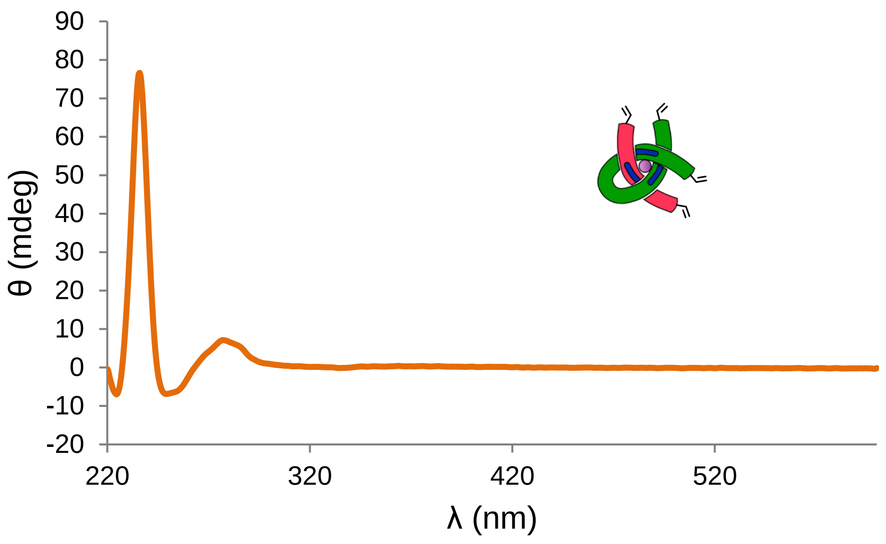

Figure S107. CD spectrum (5.0×10^{-5} M, MeCN, 298 K) of Λ-{(R)$_2$-**L3**,(R)$_4$-**L4**•[Lu]}.

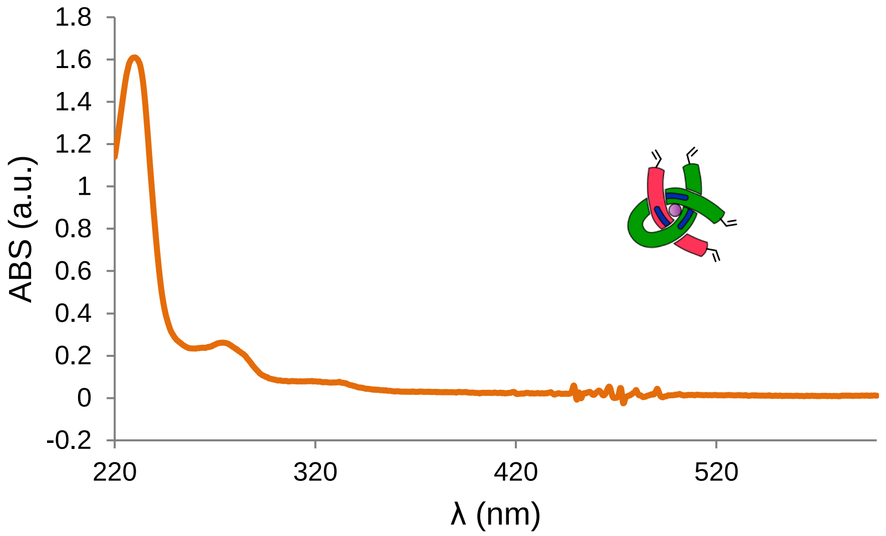

Figure S108. UV/vis spectrum (5.0×10^{-5} M, MeCN, 298 K) of Λ-{(R)$_2$-**L3**,(R)$_4$-**L4**•[Lu]}.

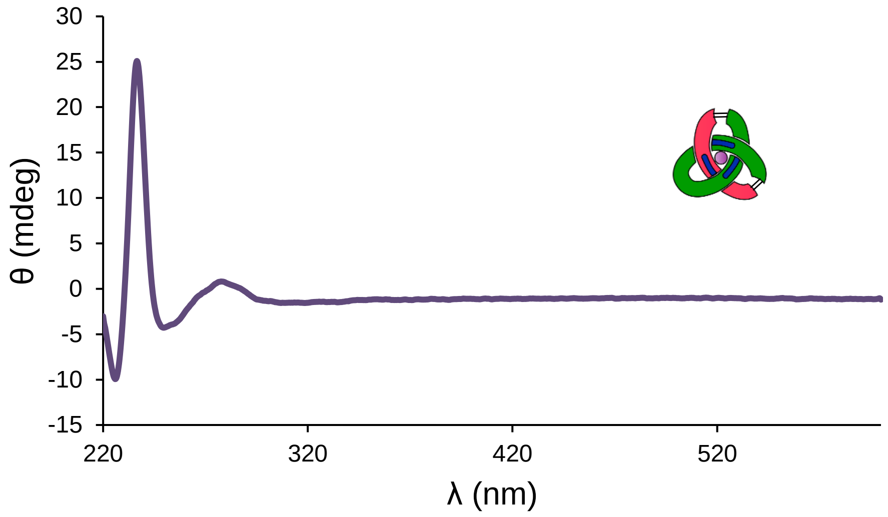

Figure S109. CD spectrum (5.0×10^{-5} M, MeCN, 298 K) of Λ-**1**•[Lu].

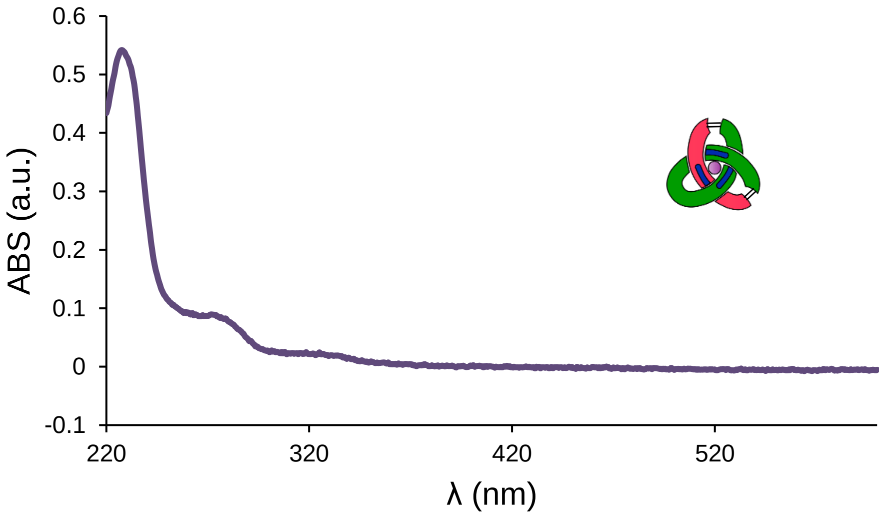

Figure S110. UV/vis spectrum (5.0×10^{-5} M, MeCN, 298 K) of Λ-**1**•[Lu].

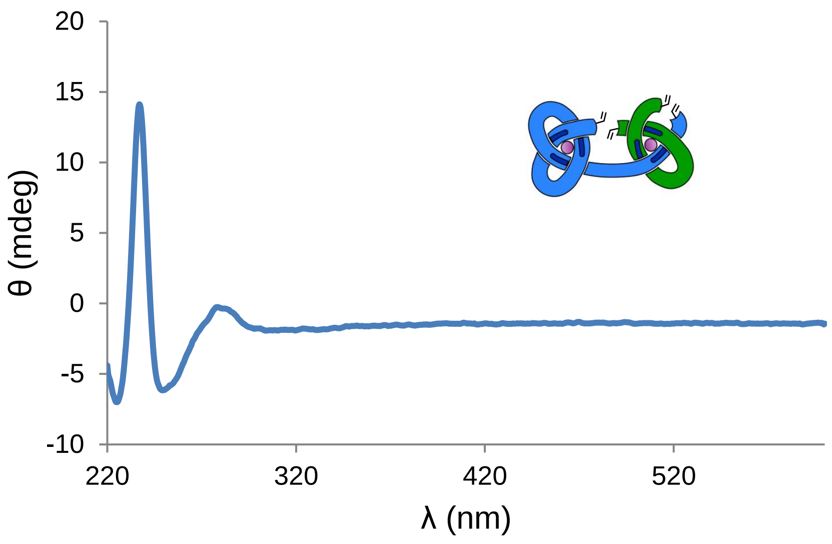

Figure S111. CD spectrum (5.0×10^{-5} M, MeCN, 298 K) of (Λ,Λ)-{$(R)_4$-**L4**,$(R)_8$-**L5**•$[Lu]_2$}.

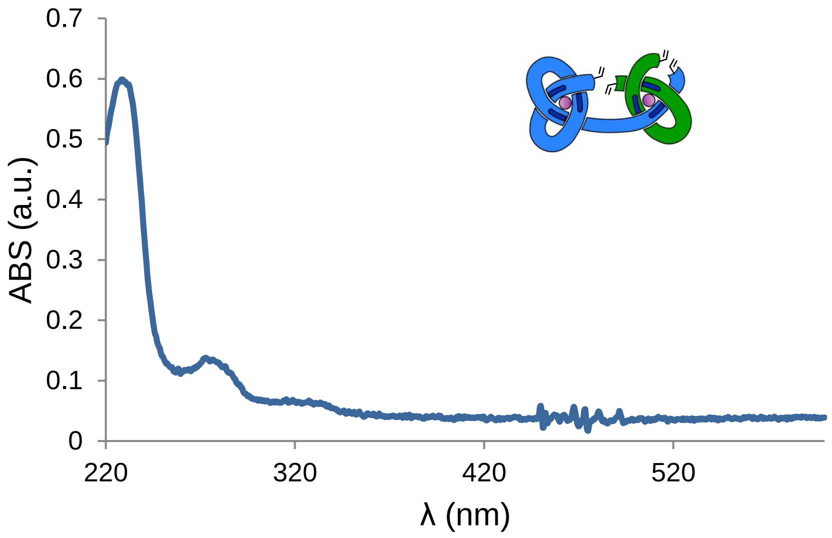

Figure S112. UV/vis spectrum (5.0×10^{-5} M, MeCN, 298 K) of (Λ,Λ)-{$(R)_4$-**L4**,$(R)_8$-**L5**•$[Lu]_2$}.

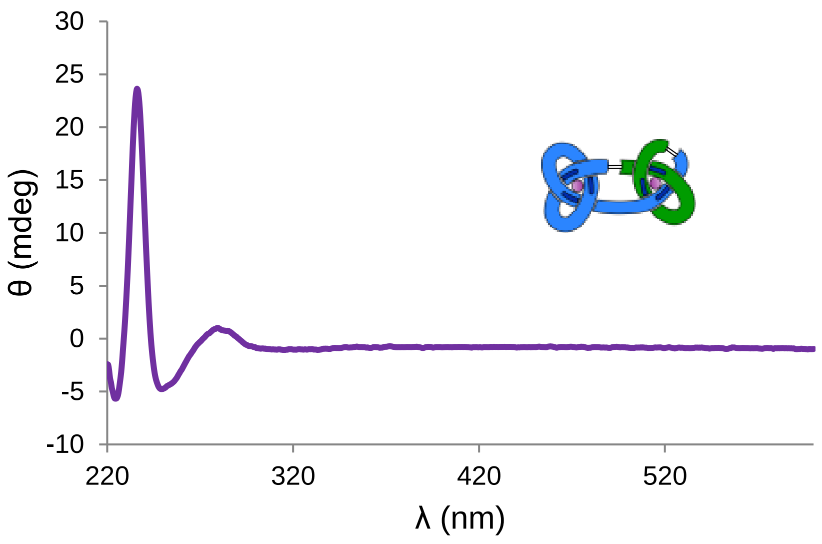

Figure S113. CD spectrum (5.0×10^{-5} M, MeCN, 298 K) of (Λ,Λ)-**2**•[Lu]$_2$.

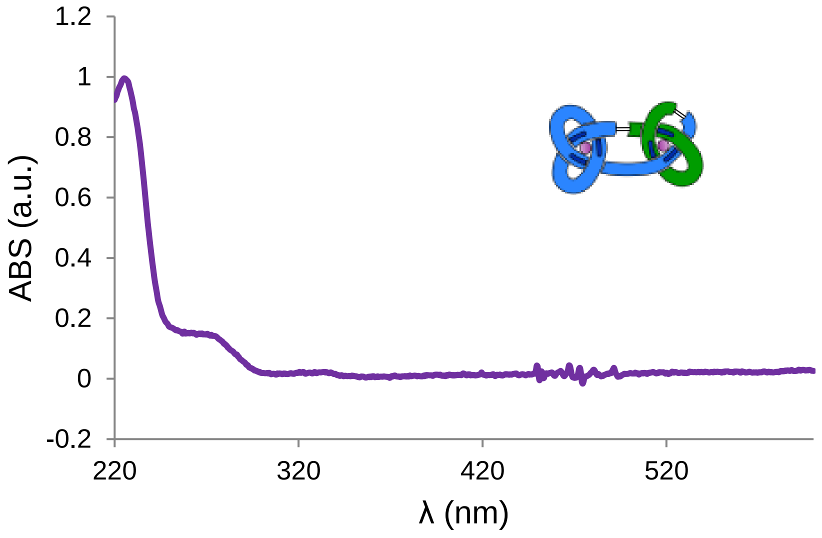

Figure S114. UV/vis spectrum (5.0×10^{-5} M, MeCN, 298 K) of (Λ,Λ)-**2**•[Lu]$_2$.

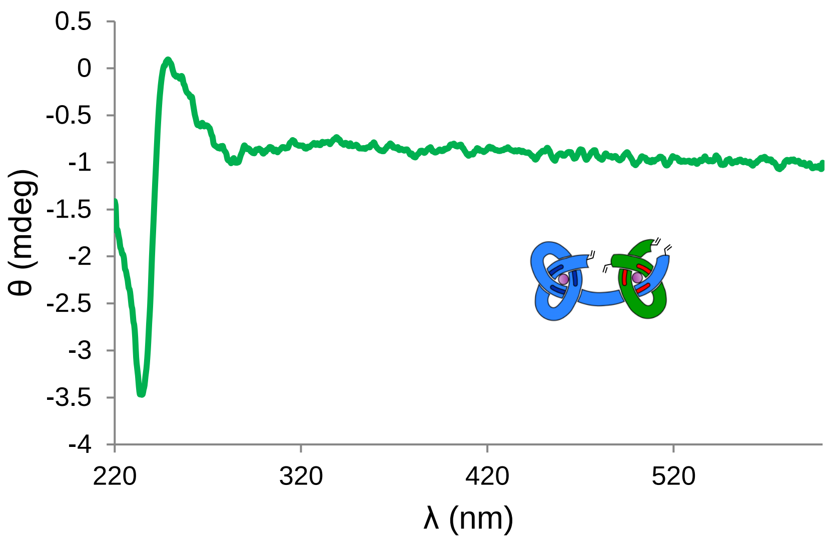

Figure S115. CD spectrum (5.0×10^{-5} M, MeCN, 298 K) of (Λ,Δ)-{(*S*)$_4$-**L4**,(*S*)$_2$(*R*)$_6$-**L5**•[Lu]$_2$}.

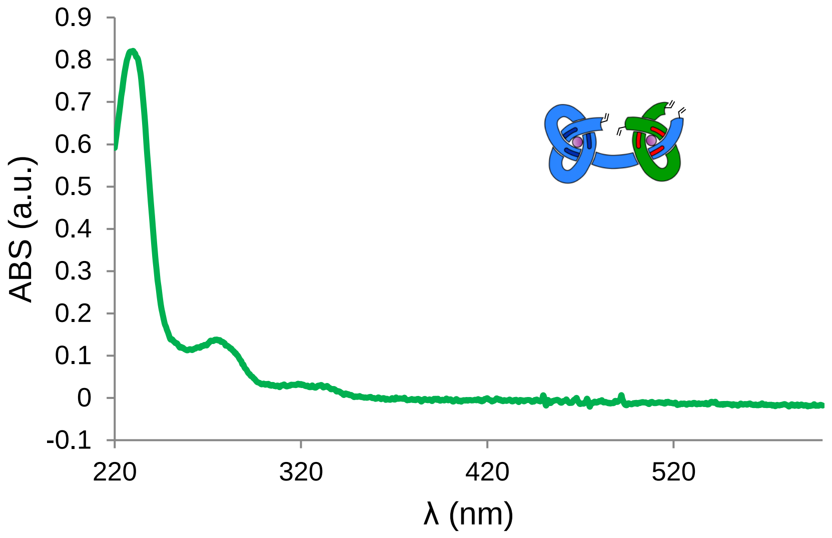

Figure S116. UV/vis spectrum (5.0×10^{-5} M, MeCN, 298 K) of (Λ,Δ)-{(*S*)$_4$-**L4**,(*S*)$_2$(*R*)$_6$-**L5**•[Lu]$_2$}.

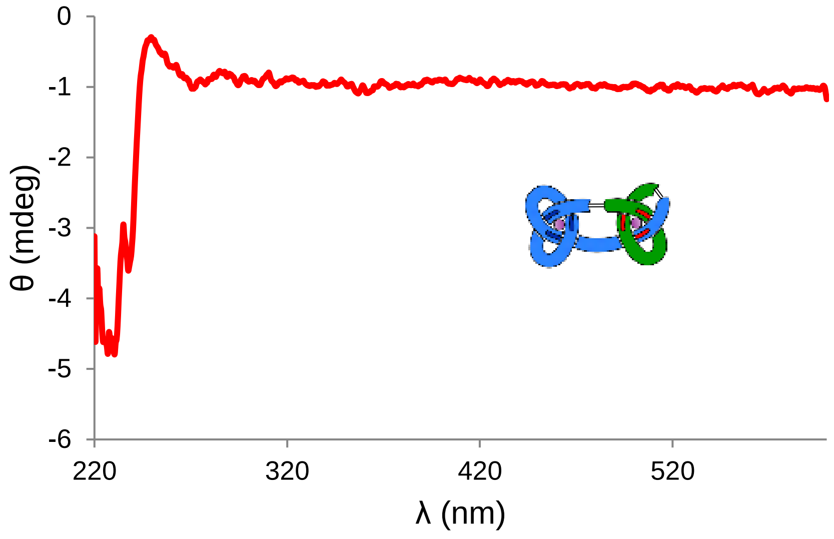

Figure S117. CD spectrum (5.0×10^{-5} M, MeCN, 298 K) of (Λ,Δ)-**2**•[Lu]$_2$.

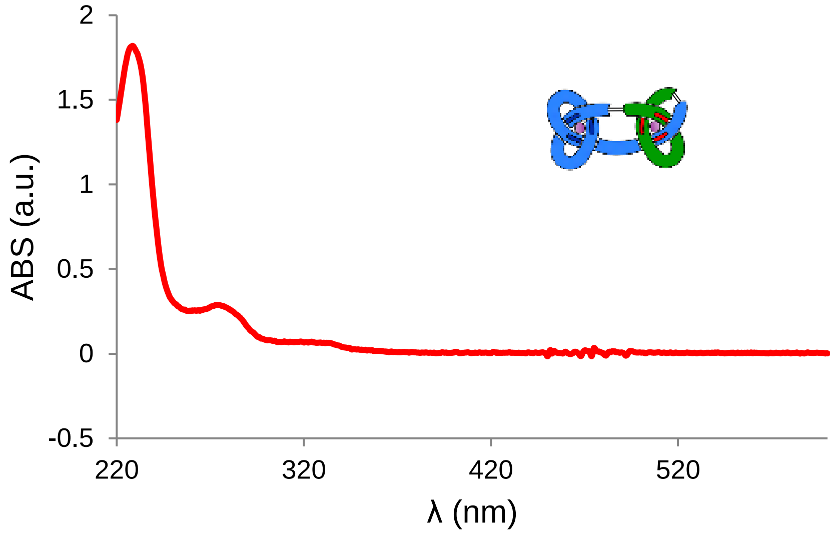

Figure S118. UV/vis spectrum (5.0×10^{-5} M, MeCN, 298 K) of (Λ,Δ)-**2**•[Lu]$_2$.

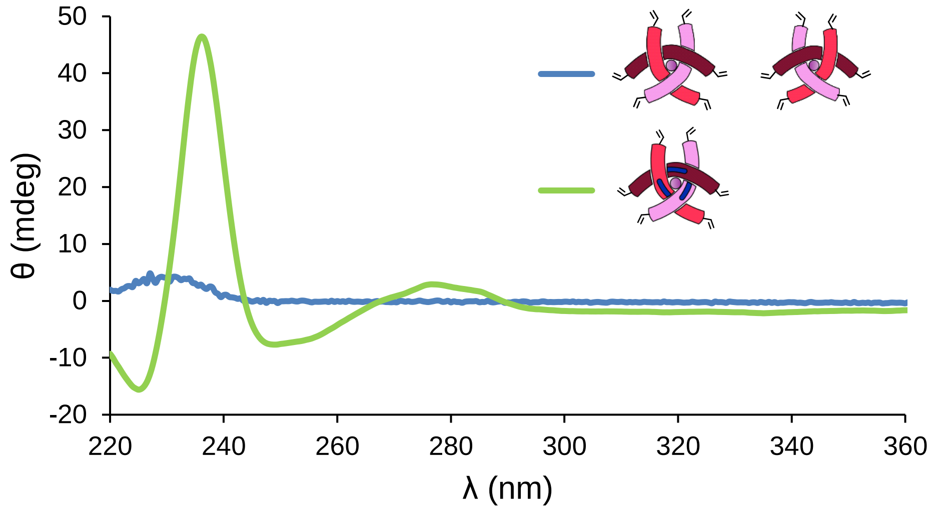

Figure S119. CD spectral stack plot (5.0×10^{-5} M, MeCN, 298 K) showing comparison of Λ-((*R*)$_2$-**L3**)•[Lu] (green) and **L1**$_3$•[Lu] (blue), normalized for absorbance.

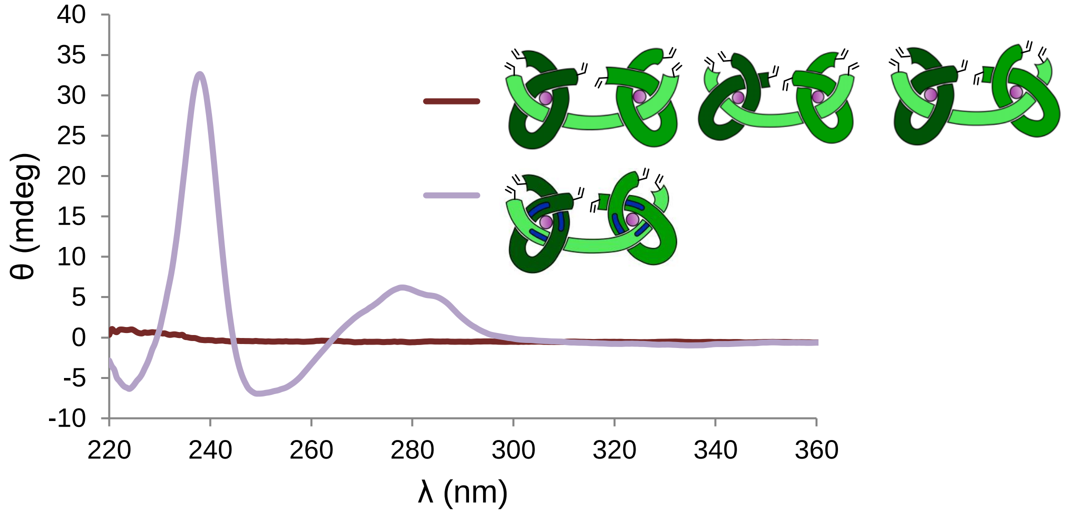

Figure S120. CD spectral stack plot (5.0×10^{-5} M, MeCN, 298 K) showing comparison of **L2**$_3$•[Lu]$_2$ (brown) and (Λ,Λ)-((*R*)$_4$-**L4**)$_3$•[Lu]$_2$ (purple), normalized for absorbance.

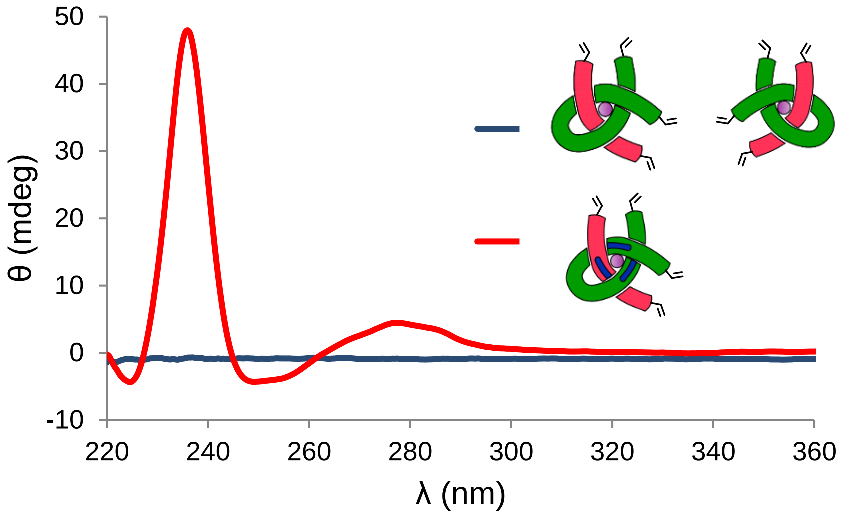

Figure S121. CD spectral stack plot (5.0×10^{-5} M, MeCN, 298 K) showing comparison of {**L1**,**L2**•[Lu]} (blue) and Λ-{(*R*)$_2$-**L3**,(*R*)$_4$-**L4**•[Lu]} (red), normalized for absorbance.

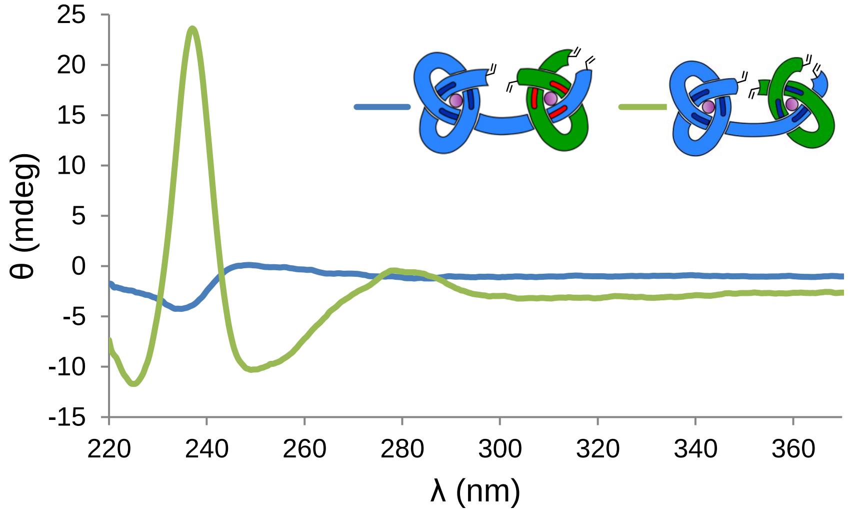

Figure S122. CD spectral stack plot (5.0×10^{-5} M, MeCN, 298 K) showing comparison of (Λ,Λ)-{(*R*)$_4$-**L4**,(*R*)$_8$-**L5**•[Lu]$_2$} (green) and (Λ,Δ)-{(*S*)$_4$-**L4**,(*S*)$_2$(*R*)$_6$-**L5**•[Lu]$_2$} (blue), normalized for absorbance.

S8. Further 2D NMR Spectroscopic Characterisation

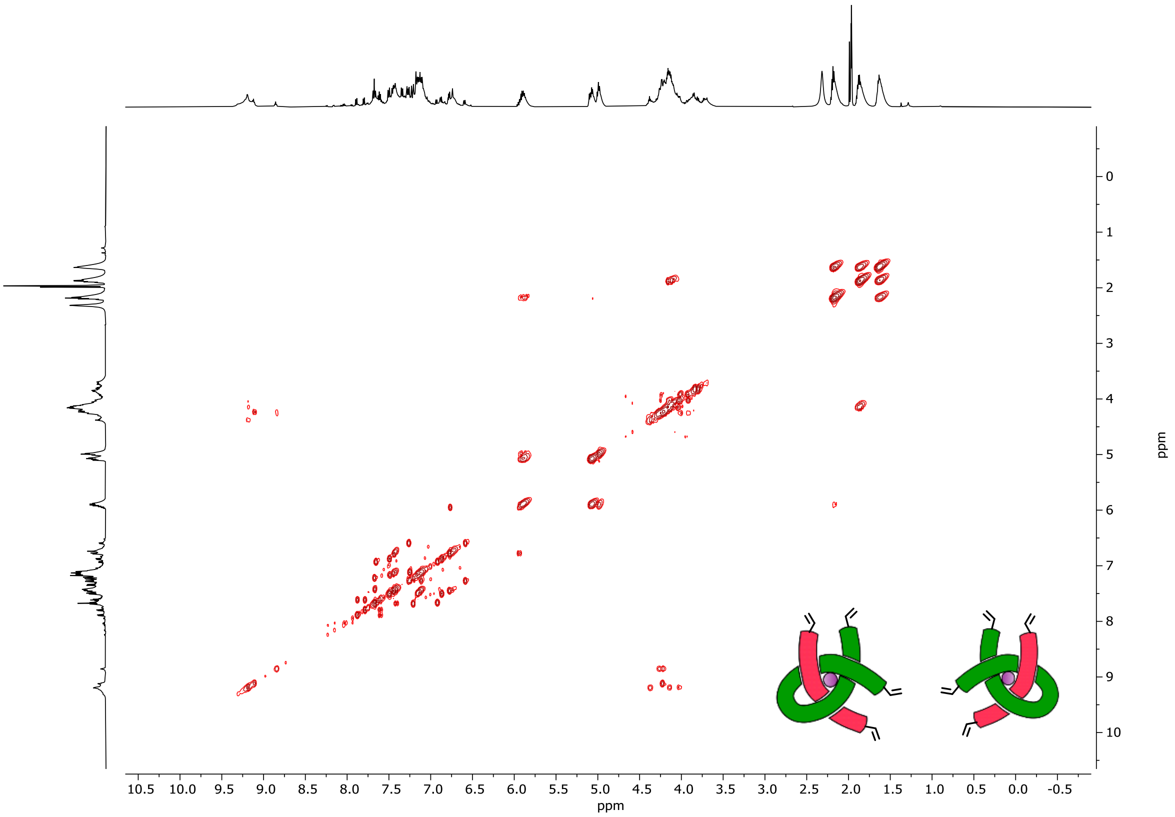

Figure S123. ^{1}H-^{1}H COSY NMR spectrum (600 MHz, MeCN-d_3, 298 K) of {**L1**,**L2**•[Lu]}.

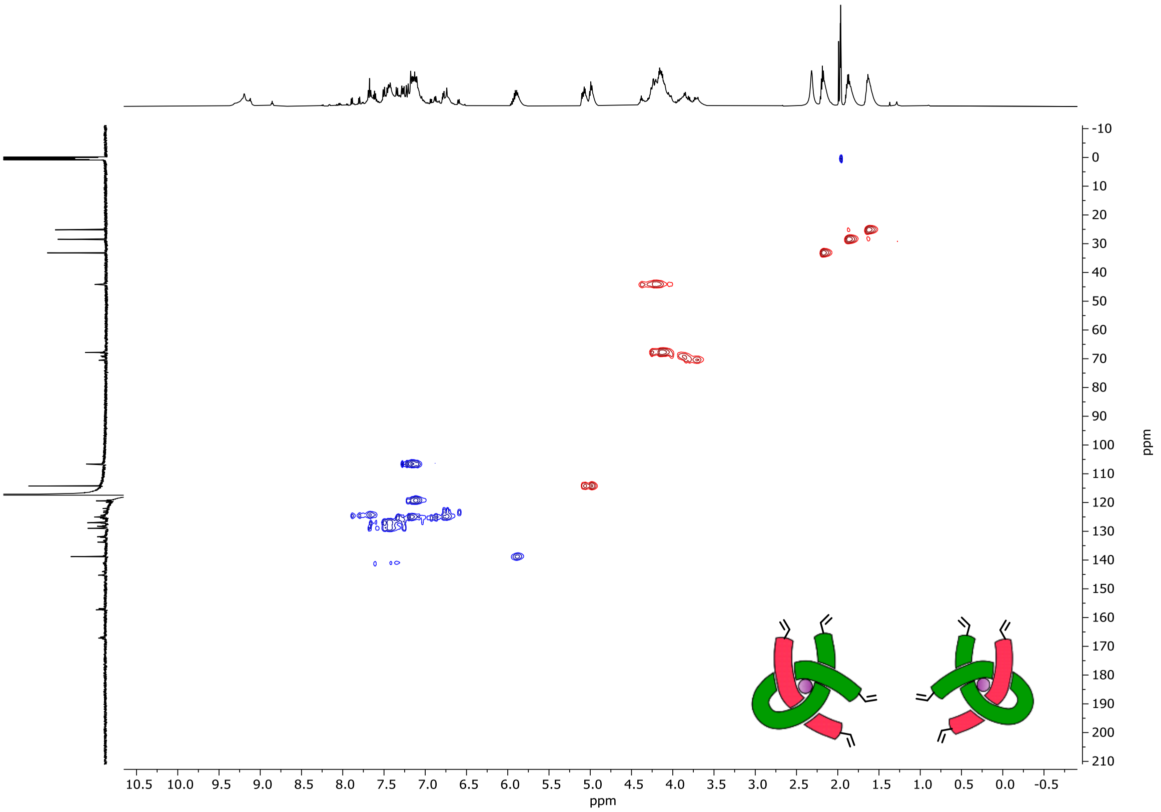

Figure S124. ^{1}H-^{13}C HSQC NMR spectrum (600 MHz, MeCN-d_3, 298 K) of {**L1**,**L2**•[Lu]}.

S9. References

1. Liang, C.; Cerf, C.; Mislow, K. Specification of chirality for links and knots. *J. Math. Chem.* **1996**, *19*, 241–263.
2. a) Cai, W.; Wu, J.; Liu, W.; Xie, Y.; Liu, Y.; Zhang, S.; Xu, W.; Tang, L.; Wang, J.; Zhao, G. Systematic structure-activity relationship (SAR) exploration of diarylmethane backbone and discovery of a highly potent novel uric acid transporter 1 (URAT1) inhibitor. *Molecules* **2018**, *23*, 252–290; b) Cody, W. L.; Holsworth, D. D.; Powell, N. A.; Jalaie, M.; Zhang, E.; Wang, W.; Samas, B.; Bryant, J.; Ostroski, R.; Ryan, M. J.; Edmunds, J. J. The discovery and preparation of disubstituted novel amino-aryl-piperidine-based renin inhibitors. *Bioorg. Med. Chem*. **2005**, *13*, 59–68.
3. Zhang, G.; Gil-Ramírez, G.; Markevicius, A.; Browne, C.; Vitorica-Yrezabal, I. J.; Leigh, D. A. Lanthanide template synthesis of trefoil knots of single handedness. *J. Am. Chem. Soc.* **2015**, *137*, 10437−10442.
4. Ashbridge, Z.; Kreidt, E.; Pirvu, L.; Schaufelberger, F.; Halldin Stenlid, J.; Abild-Pedersen, F.; Leigh, D. A. Vernier template synthesis of very large molecular knots. Submitted for publication.
